# Alterations in the LRRK2-Rab pathway in urinary extracellular vesicles as Parkinson’s disease and pharmacodynamic biomarkers

**DOI:** 10.1038/s41531-023-00445-9

**Published:** 2023-02-07

**Authors:** Jean-Marc Taymans, Eugénie Mutez, William Sibran, Laurine Vandewynckel, Claire Deldycke, Séverine Bleuse, Antoine Marchand, Alessia Sarchione, Coline Leghay, Alexandre Kreisler, Clémence Simonin, James Koprich, Guillaume Baille, Luc Defebvre, Kathy Dujardin, Alain Destée, Marie-Christine Chartier-Harlin

**Affiliations:** 1 Univ. Lille, Inserm, CHU Lille, UMR-S 1172 - LilNCog - Lille Neuroscience & Cognition, F-59000 Lille, France; 2 CHU Lille, Movement Disorders Unit, F-59000 Lille, France; 3 Atuka Inc., Toronto, ON Canada

**Keywords:** Diagnostic markers, Parkinson's disease, Parkinson's disease

## Abstract

Expression or phosphorylation levels of leucine-rich repeat kinase 2 (LRRK2) and its Rab substrates have strong potential as disease or pharmacodynamic biomarkers. The main objective of this study is therefore to assess the LRRK2-Rab pathway for use as biomarkers in human, non-human primate (NHP) and rat urine. With urine collected from human subjects and animals, we applied an ultracentrifugation based fractionation protocol to isolate small urinary extracellular vesicles (uEVs). We used western blot with antibodies directed against total and phosphorylated LRRK2, Rab8, and Rab10 to measure these LRRK2 and Rab epitopes in uEVs. We confirm the presence of LRRK2 and Rab8/10 in human and NHP uEVs, including total LRRK2 as well as phospho-LRRK2, phospho-Rab8 and phospho-Rab10. We also confirm LRRK2 and Rab expression in rodent uEVs. We quantified LRRK2 and Rab epitopes in human cohorts and found in a first cohort that pS1292-LRRK2 levels were elevated in individuals carrying the LRRK2 G2019S mutation, without significant differences between healthy and PD groups, whether for LRRK2 G2019S carriers or not. In a second cohort, we found that PD was associated to increased Rab8 levels and decreased pS910-LRRK2 and pS935-LRRK2. In animals, acute treatment with LRRK2 kinase inhibitors led to decreased pT73-Rab10. The identification of changes in Rab8 and LRRK2 phosphorylation at S910 and S935 heterologous phosphosites in uEVs of PD patients and pT73-Rab10 in inhibitor-dosed animals further reinforces the potential of the LRRK2-Rab pathway as a source of PD and pharmacodynamic biomarkers in uEVs.

## Introduction

One of the key challenges in Parkinson’s disease research today is to identify biomarkers for the early diagnosis of Parkinson’s disease (PD), for the monitoring of the progression of PD pathology and for the assessment of therapeutic interventions. Indeed, biological biomarkers for PD are currently unavailable in routine clinical practice. Much hope in developing PD biomarkers is vested in exploiting findings from biological studies of genetic determinants of PD. These same genetic determinants are also the basis for a growing number of novel therapeutic strategies with disease-modifying potential. Some examples of therapies based on PD genetic determinants that have been developed in preclinical models and are currently in clinical trials include strategies to counter the aggregation of alpha-synuclein, encoded by the *SNCA* gene^1^, or to inhibit the kinase activity of leucine-rich repeat kinase 2 (LRRK2)^2^. These approaches are expected to block the further development of disease, and it is therefore beneficial to detect the disease in the earliest stage possible. Unfortunately, current diagnostic approaches are only capable of detecting relatively late stages of PD, *i.e*. when motor symptoms begin to appear, which corresponds to a stage where at least half of the dopaminergic neurons have been lost. This constitutes an additional argument reinforcing the pressing need to develop PD biomarkers, as current tools are weak in identifying early stage disease and in assessing target engagement and the therapeutic effect of novel therapies. The genetic determinants of PD constitute an interesting starting point to identify candidate biomarkers for PD, including *LRRK2*, which is the focus of the present study.

*LRRK2* encodes a large protein of 2527 amino acids, harboring multiple domains including a GTPase domain, a kinase domain and other domains with a role in protein-protein interactions. Linkage studies have identified several mutations in the coding sequence that segregate with autosomal dominant PD, while genome-wide association studies (GWAS) have revealed that genomic variation at the LRRK2 locus is associated with sporadic PD, demonstrating that LRRK2 is implicated in a large proportion of PD cases. Research efforts of recent years have elucidated several aspects of LRRK2 function and signaling that can be measured as a marker of LRRK2 activity or status. For instance, LRRK2 is a kinase that autophosphorylates itself at multiple serines and threonines in or near its ROC domain, including at Ser1292 whose phosphorylation is considered a marker for LRRK2 autophosphorylation. LRRK2 also displays other phosphorylation sites resulting from heterologous phosphorylation, specifically at sites S910-S935-S955-S973 in the ANK-LRR interdomain region. These sites mediate interaction with 14-3-3 proteins and may constitute an activation or inactivation step in LRRK2 cellular activity. Downstream of LRRK2, several of the Rab family small GTPases involved in membrane trafficking are robust substrates of LRRK2, including Rab8A and Rab10.

Several pieces of evidence point to the potential of measuring LRRK2 pathway activity to monitor disease or target engagement, including total or phosphorylated levels of LRRK2 and total or phosphorylated levels of LRRK2’s Rab substrates (reviewed in Rideout et al.^3^). LRRK2 is expressed in the central nervous system, both in brain^4,5^ as well as in exosome-rich isolates from CSF and urine^6^. Reports show increases in LRRK2 protein levels in the prefrontal cortex of PD patients relative to controls^7^, suggesting that increase in total LRRK2 protein expression is correlated with disease. LRRK2 phosphorylation is modulated in a majority of disease variants. For the S910-S935-S955-S973 phosphosites, levels are reduced for most mutants^8^, while for phospho-S1292, levels are increased for most mutants^9^ (reviewed in ref. ^10^). Also, all of these five sites are rapidly dephosphorylated upon LRRK2 kinase inhibitor treatment^11–13^, considered potential therapeutics. Analogous to the LRRK2 autophosphorylation, phosphorylation of LRRK2’s Rab substrates is increased in the presence of disease mutant forms of LRRK2 and decreased upon kinase inhibition of LRRK2. Therefore, the LRRK2-Rab pathway is a promising source of markers for both disease as well as pharmacodynamic response.

In biomarker development, one key aspect is the sample type that will be measured. The sample type tested in the present study, urine, has the particularity that relatively large quantities can be collected quickly, with minimal equipment and with low discomfort to the patient. Specifically related to assessing the LRRK2-Rab pathway for PD or pharmacodynamic biomarkers, urine ticks several boxes. Both LRRK2 and Rab proteins are known components of urinary extracellular vesicles (uEVs), specifically EVs of small size (30-150 nm) composed of microvesicles and exosomes^6,14^. Lipidomics studies have shown that bis(monoacylglycero)phosphate (BMP), a phospholipid that can be found in exosomes, is decreased in urine of LRRK2 kinase inhibitor treated cynomolgous monkeys and in LRRK2 KO mouse urine, while diBMP is elevated in urine of patients harboring LRRK2 mutations^15^. In addition, proteomics analysis of urine collected from sporadic and LRRK2-G2019S-PD showed that patients display different urine proteome profiles compared to healthy controls^16^. Previous work also reported that pS1292-LRRK2 levels were elevated in groups of PD patients carrying the LRRK2 G2019S mutation as well as in groups of sporadic PD patients^17,18^. However, it is unclear what the status is of LRRK2 heterologous phosphorylation sites and Rab proteins in urinary EVs of PD patients or of rodents after LRRK2 inhibitor treatment.

In the present study, we analyzed extracellular vesicles isolated from urine samples to assess the biomarker potential of measures of the LRRK2-Rab pathway in idiopathic and LRRK2 linked PD as well as in inhibitor-dosed rodents and non-human primates. Our results show modifications in the LRRK2-Rab pathway in uEVs, both in disease and after LRRK2 kinase inhibitor treatment.

## Results

**Fig. 1:**
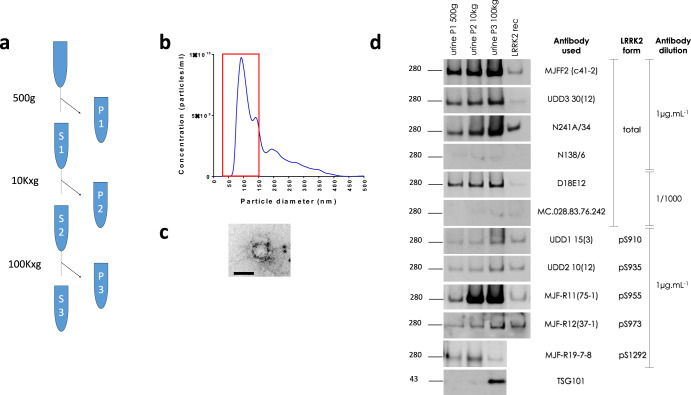
Detection and characterization of LRRK2 and Rab epitopes in urinary exosome enriched extracellular vesicles (uEVs). **a** Flow diagram of the differential centrifugation protocol employed to isolate uEVs. The full method is described in detail in materials and methods. **b** Nanoparticle tracking analysis of extracellular vesicles isolated from urine according to the method schematized in (**a**) (sample P3). **c** LRRK2 detection via immunogold labeling in negative stain transmission electron microscopy (TEM). The panel shows a TEM image of uEVs and dual immunogold labeling with anti-LRRK2 antibodies (RabMab UDD3 and Neuromab N241, revealed by large and small gold particles, respectively) illustrating LRRK2 labeling in uEVs. Scale bar, 100 µm. **d** Western blot detection of total and phospho-epitopes of LRRK2 with multiple different antibodies, using different fractions of urine (pellet fractions P1, P2, and P3 as schematized in A) as well as recombinant LRRK2. The presence of the exosome marker TSG101 in P3 indicates that this fraction is enriched in exosomes.

In the first step, we isolated exosome-enriched human urinary EVs using a differential centrifugation protocol (Fig. 1a) with control urine samples, and characterized these preparations using nanoparticle tracking analysis (NTA), immunogold labeling followed by transmission electron microscopy (TEM) and western blot analysis. Particle size distribution determined by NTA shows that samples are primarily composed of vesicles of 30–150 nm diameter, consistent with the size of exosomes (Fig. 1b). TEM imaging of immunogold labeled samples showed vesicles around 100 nm in diameter that are immunopositive for 2 different anti-LRRK2 antibodies (Fig. 1c). For western blot analysis, we collected fractions at the progressive steps in the sequential centrifugation protocol, including after low speed, medium speed, and ultracentrifugation. We assessed these samples and a recombinant protein standard for the detection of total and phosphorylated LRRK2 epitopes with 11 different antibodies, including 6 antibodies detecting total LRRK2 and 5 detecting phospho-LRRK2 (at sites S910, S935, S955, S973, and S1292), as well as an antibody detecting TSG101, a marker for exosomes (Fig. 1d). All antibodies detected LRRK2, with two total LRRK2 antibodies showing weak detection (N138/6 and MC.028.83.76.242), in line with comparatively lower sensitivity of detection previously reported for these antibodies^19^. As shown from the TSG101 profile, the exosome-positive EVs were enriched in the pellet of the ultracentrifugation step, while LRRK2 protein was also detected in the pellets after low-speed and medium-speed centrifugation, corresponding to fractions with larger vesicles or cells. In order to characterize the average protein content of the EV isolates (P3), we determined the total protein content for 17 urine samples selected at random from the Lille University Hospital cohort and found that the total protein concentration of the samples is on average 1856 µg/ml (±544 s.e.m.). Prior to analyzing patients samples, we generated calibration standards to be able to compare measures between runs, including full length and truncated recombinant phosphorylated LRRK2 as well as a pool of EV isolates generated by pooling a small volume of each sample of EV isolate included in the study (Supplementary Fig. 1). We verified the linearity of the quantification of signal intensity across different protein concentrations ranging from 200 ng/ml to 5 ng/ml and found linear correlations between signal intensity and protein concentrations with *R*² values all above 0.97 (Supplementary Fig. 3).

**Fig. 2:**
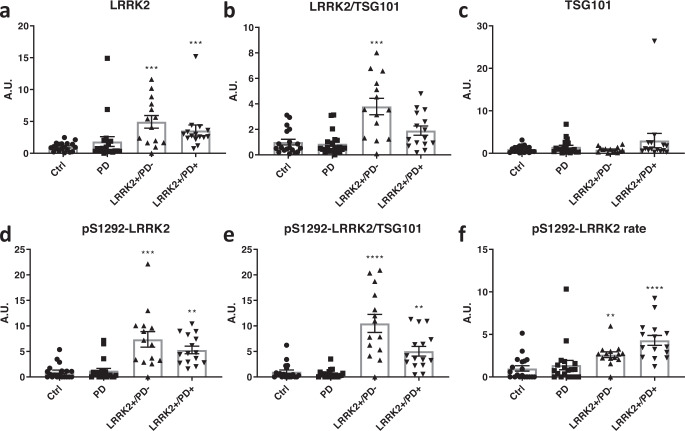
LRRK2 and pS1292-LRRK2 in urinary EVs of iPD subjects and PD-manifesting and non-PD-manifesting LRRK2 carriers compared to healthy controls. Urine samples for this study were obtained from the LRRK2 cohort consortium as described in materials and methods, and the clinical characteristics of this cohort are given in Table 1. Urinary EVs were isolated for subjects’ urine samples as described in materials and methods and total-LRRK2 (**a**), total LRRK2 normalized to TSG101 (**b**), TSG101 (**c**), pS1292-LRRK2 (**d**), pS1292-LRRK2 normalized to TSG101 (**e**) and the rate of LRRK2 phosphorylation at S1292 (**f**) were determined using western blotting (quadruplicate measures per sample). Direct values and ratios of values were normalized to the ‘pool’ calibrator (Supplementary Fig. 1). Note that the values of phosphorylation ‘rate’ are indicative of the proportion of protein that is phosphorylated at the given site, see materials and methods. Results are depicted here in the form of histograms. Significant differences were tested via the Kruskal-Wallis test, using the healthy control group as control. Error bars represent standard error of the mean (s.e.m.). *P < 0,05; **P < 0,01, ***P < 0,001, ***P < 0,0001.

We next set out to perform a first study on patient samples obtained from the LRRK2 cohort consortium (LCC), from all male subjects divided into four groups: idiopathic PD, LRRK2 G2019S carriers manifesting or not manifesting PD as well as healthy controls (Clinical parameters of the subjects are summarized in Table 1). EVs were isolated using the differential centrifugation method and EV pellets were prepared for Western blot analysis as described in “Materials and methods”. Measures of total LRRK2, pS1292-LRRK2 and the exosome marker TSG101 were made using densitometric quantification of Western blot signals as described in Materials and Methods. As intensity values of each epitope were normalized to the three different calibration standards we verified the correlations of individual values for each calibrator and found significant correlations between values from different calibrators (Supplementary Fig. 4). We therefore present here data using the ‘pool’ calibration standard while relative values derived from the other two calibrators are comparable. After Kruskal-Wallis comparisons, significantly higher values compared to the healthy control group were observed in LRRK2 carriers (both manifesting and non-manifesting) for total-LRRK2, LRRK2 phosphorylation rate at S1292 as well as raw pS1292-LRRK2 values or the pS1292-LRRK2/TSG101 ratio. The ratio total LRRK2/TSG101 is significantly higher in non-manifesting LRRK2 carriers compared to healthy controls. No significant differences were observed between the healthy control and idiopathic PD groups, neither were any significant differences observed between non-manifesting and manifesting LRRK2 carriers (Fig. 2). These data were submitted to a Pearson’s correlation analysis to identify correlations between the LRRK2 measures and clinical parameters (summarized in supplementary Table 1) and we found a significant correlation for total LRRK2 with overall cognition as assessed by the score at the Montreal Cognitive assessment (MoCA), TSG101 with total levodopa equivalent dose and Schwab and England consensus rating, raw pS1292-LRRK2 values with age and age at onset, as well as S1292-LRRK2 phosphorylation rate with Hoehn & Yahr stage and Schwab and England consensus rating.

**Table 1:** Overview of values for key clinical parameters of samples from the LRRK2 cohort consortium: age at sampling, age at onset, age at diagnosis, disease duration, familial history (y/n), Levodopa Equivalent Daily Dose (LEDD), Unified Parkinson’s disease rating scale III (UPDRS III), modified Hoehn and Yahr Stage, Modified Schwab and England Consensus Rating, Montreal Cognitive Assessment (MoCA).

Descriptor	LRRK2-/PD- *n* = 19	LRRK2−/PD+ *n* = 20	LRRK2+/PD− *n* = 14	LRRK2+/PD+ *n* = 15	Statistical summary^a^
Age at sampling (years), mean ± SD (median)	61.3 ± 10.9 (61)	62.6 ± 8.4 (62)	60.3 ± 12.9 (59.5)	66.1 ± 11.5 (71)	n.s.^b^
Age at onset (years), mean ± SD (median)	NA	56.1 ± 24.4 (55)	NA	55.1 ± 12.6 (58)	n.s.
Age at diagnosis (years), mean ± SD (median)	NA	58 ± 20 (58)	NA	57.2 ± 19.5 (61)	n.s.
Disease duration (years), mean ± SD (median)	NA	5.9 ± 3.1 (6)	NA	11.1 ± 5.6 (10)	***0.0018***
Familial History of PD, n= total no/total yes	4/15	12/8	1/13	5/10	0.118^c^
LEDD mg, mean ± SD (median)	NA	566 ± 406 (550)	NA	1060 ± 725 (835)	0.0861
UPDRS III, mean ± SD (median)	NA	25.8 ± 10.5 (25)	NA	22.6 ± 8.9 (24)	n.s.
Modified Hoehn and Yahr score, mean ± SD (median)	NA	2 ± 0.3 (2)	NA	2.5 ± 0.8 (2)	***0.0206***
Schwab and England, mean ± SD (median)	NA	90.3 ± 7 (90)	NA	71.4 ± 19 (80)	***0.0008***
MoCA, mean ± SD (median), out of 30	26.4 ± 2.5 (27)	25.9 ± 3.2 (27)	25.6 ± 3.6 (26.5)	22.4 ± 4.3 (23)	***0.0145*** for LRRK2+/PD+, n.s. for LRRK2−/PD+ and LRRK2+/PD−^b^

^a^*P*-values > 0.3 are indicated as n.s. (not significant). *P*-values < 0.05 are bold italics. Statistical comparisons are carried out by the use of the 2-tailed Mann–Whitney test for the LRRK2−/PD+ group compared to the LRRK2+/PD+ group, except for comparisons indicated by ^b^where all groups are compared by use of the Kruskal–Wallis test followed by the Dunn’s multiple comparison test using the LRRK2−/PD− group as the reference group and for comparisons indicated by ^c^where a Chi² test is applied.

Because these data only partially replicated previously published data, we next analyzed an additional cohort of samples collected at the Lille University Hospital from both male and female subjects. These included 53 healthy controls as well as 60 idiopathic PD patients and 7 PD patients carrying the G2019S LRRK2 mutation. Clinical parameters of the subjects are summarized in Table 2 (and in Supplementary Table 2 for the breakdown of parameters between men and women). We included in the analysis the quantification of total and pS1292-LRRK2 as well as additional measures, including pS910-LRRK2, pS935-LRRK2, LRRK2’s substrate Rab8A and its LRRK2-dependent phospho-epitope, pT72-Rab8a. As shown above for the detection of LRRK2, we optimized the conditions of detection of Rab8a and pT72-Rab8a and tested the linearity of quantification using a recombinant protein preparation phosphorylated in vitro by recombinant LRRK2 (Supplementary Figs. 5, 6). We found that Rab8a could be detected in human urinary EVs, with a detection limit of recombinant Rab8a close to 20 ng/ml (Supplementary Fig. 5). A representative image of the blots that were used for quantification, illustrating the multiple epitopes detected on the same samples, is given in Supplementary Fig. 7. Statistical significance was first tested for the full groups of healthy controls (HC) compared to idiopathic PD patients and subsequently the same comparisons were performed separately for males and females.

**Table 2:** Overview of clinical parameters from clinical cohort 2 (cohort from Lille University Hospital).

				Statistical summary^a^	
Descriptor	Controls (*n* = 53)	Idiopathic PD (*n* = 60)	G2019S LRRK2 PD (*n* = 7)	Control vs iPD	iPD vs LRRK2-PD
Gender, M/F n	22/31	36/24	3/4	***0.0497***^b^	n.s.
Weight (kg), mean ± SD (median)	74.1 ± 14.2 (74)	78.6 ± 17.7 (77)	80.7 ± 18.1 (80)	0.2598	n.s.
Height (cm), mean ± SD (median)	169.4 ± 9.7 (168)	171 ± 9.3 (172)	165.1 ± 8 (164)	0.2976	0.1189
Body Mass Index, mean ± SD (median)	25.6 ± 5.3 (25)	26.8 ± 4.6 (26)	29.7 ± 5.4 (31)	0.1633	0.1222
Age at sampling (years), mean ± SD (median)	64,3 ± 9.6 (63)	61.6 ± 11.2 (63)	51 ± 10.6 (47)	0.2720	***0.0311***
Age at onset (years), mean ± SD (median)	NA	55.3 ± 12.3 (57)	44.7 ± 11.1 (44)	NA	***0.0294***
Age at diagnosis (years), mean ± SD (median)	NA	56.4 ± 12.1 (57)	45.7 ± 11.6 (45)	NA	***0.0270***
Disease duration (years), mean ± SD (median)	NA	5.3 ± 5.9 (4)	5.3 ± 4.3 (6)	NA	n.s.
de novo, total (%)	NA	13 (22%)	1 (14%)	NA	n.s.
Familial History of PD, n= total no/total yes	NA	40/20	4/3	NA	n.s.
LEDD mg, mean ± SD (median)	NA	618 ± 570 (462)	678 ± 389 (580)	NA	n.s.
UPDRS III, mean ± SD (median)	NA	20.3 ± 11.4 (20)	15.6 ± 6.6 (15)	NA	n.s.
Modified Hoehn and Yahr score, mean ± SD (median)	NA	2.35 ± 0.8 (2)	2 ± 0 (2)	NA	0.1012
Schwab and England, mean ± SD (median)	NA	80 ± 15 (90)	85 ± 34 (90)	NA	n.s.
MoCA, mean ± SD (median), out of 30	NA	25.0 ± 3 (25)	25.1 ± 2.9 (26)	NA	n.s.
MMSE, mean ± SD (median)	28.4 ± 1.4 (29)	27.5 ± 2 (28)	27.9 ± 1.5 (27)	***0.0225***	n.s.
Cognitive Impairment, n	NA	MCI *n* = 21, dementia *n* = 5,	MCI *n* = 4, dementia *n* = 0,	NA	0.14
		no cognitive impairment *n* = 24, unknown *n* = 10	no cognitive impairment *n* = 3, unknown *n* = 0		

See Supplementary Table 2 for the breakdown of these parameters for men and women.

^a^*P*-values > 0.3 are indicated as n.s. (not significant). *P*-values < 0.05 are bold italics. Statistical comparisons are carried out for controls compared to idiopathic PD and for idiopathic PD compared to LRRK2 PD by use of the 2-tailed Mann–Whitney test. Statistical comparisons indicated by ^b^are determined via a Chi² test.

*LEDD* L-dopa equivalent daily dosage, *MMSE* Mini-Mental State Examination, *MoCA* The Montreal Cognitive Assessment, *PD* Parkinson disease, *UPDRS* Unified Parkinson’s Disease Rating Scale, *NA* not applicable.

**Fig. 3:**
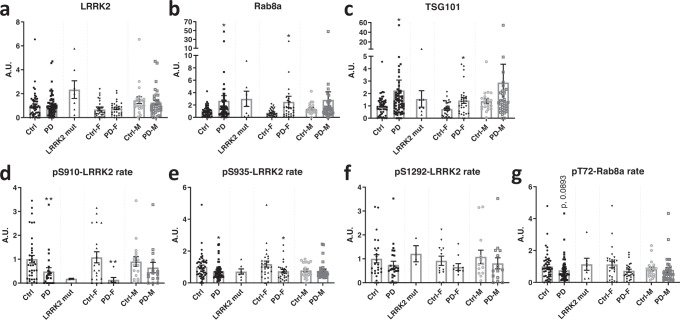
Analysis of LRRK2 and Rab levels in urinary EVs of PD patients compared to healthy controls. Urine samples of PD vs control groups were collected at Lille University Hospital. Genotype and diagnosis confirmed for all subjects. In total, this cohort included 77 PD patients (41 men and 33 women, with 7 patients carrying the G2019S mutation) as well as 53 control subjects (22 men and 31 women). An overview of the clinical characteristics of the different groups is given in Table 2. EVs were isolated from the urine samples and total levels and phosphorylation rates of LRRK2 and Rab8 were measured together with TSG101 levels via western blotting (quadruplicate measures per sample). Results were expressed relative to a calibrator and represented as a histogram. Values of phosphorylation ‘rate’ are indicative of the proportion of protein that is phosphorylated at the given site, see materials and methods. Shown here are values for total LRRK2 (**a**), total Rab8a (**b**), TSG101 (**c**), and phosphorylation rates for pS910-LRRK2 (**d**), pS935-LRRK2 (**e**), pS1292-LRRK2 (**f**) and pT72-Rab8a (**g**). Note that histograms each show first 2 bars corresponding to the values obtained from the groups of healthy controls and idiopathic PD patients, then a third bar with values of PD patients carrying the G2019S mutation, the last 4 bars correspond to the same values as the healthy controls vs iPD, now separated out per gender. Significant differences are tested using the two-tailed Mann–Whitney test. Note that the values of the LRRK2-G2019S group are shown for information, but are not included in the statistical analysis due to the smaller group size (*N* = 7). Error bars represent standard error of the mean (s.e.m.). *P < 0,05; **P < 0,01.

We observed that there is a significant difference between the healthy control compared to idiopathic PD groups for total Rab8 and total TSG101 (upregulation) and for the S910-LRRK2 and S935-LRRK2 phosphorylation rates (downregulation) (Fig. 3). Receiver operator characteristic analysis for these 4 parameters yielded areas under the curve ranging from 0,6047 to 0,6925 (Supplementary Fig. 8). We noted 2 subjects displaying strong outlying values for Rab8 and 1 of these 2 also displaying a strong outlier for TSG101 levels (Fig. 3B, C). Upon review of the clinical characteristics of these 2 subjects, we did not find a specific explanation for this, as we observed typical PD without red flags and without specific kidney or inflammatory conditions, suggesting that these outliers are an aspect of normal variability of these. Note also that the exclusion of these 2 values did not alter the statistical conclusion of the significant increase of Rab8 and TSG101 in PD. For markers related to LRRK2 kinase activity, including S1292-LRRK2 phosphorylation rate and T72-Rab8 phosphorylation rate, no significant differences were observed between HC and iPD groups. It is also noteworthy that group averages of these measures were not higher in the iPD group compared to the HC group.

When measures were broken down per gender, we found that observations made in the full groups were confirmed in females but not in males. The lack of difference for the male HCs compared to iPDs may be explained by variability in the data, for instance for Rab8 and TSG101 where differences in group averages are comparable for males and females. However, this is not the case for S910-LRRK2 and S935-LRRK2 phosphorylation rates where group average differences are very low in males compared to females. To complete these results, the values of a small group (*n* = 7) of PD patients that were found in the Lille cohort to be carrying the G2019S mutation were also included in the graphs of Fig. 3, without including these in the statistical analysis due to the small sample size. In our correlation analysis (Table 3), we found that total LRRK2 and Rab8 both correlated with weight and BMI, while pT72-Rab8 correlated with age at onset and age at diagnosis. Also, in order to explore these measures in function of specific clinical parameters, we compared PD patients with and without mild cognitive impairment or de novo compared to non de novo or treated (a subset of non de novo patients) (Supplementary Fig. 9). We found that TSG101 was significantly increased in non de novo patients compared to de novo patients and there was a non-significant tendency for an increase in Rab8 and decrease in S910-LRRK2 phosphorylation rate for the same comparison. Interestingly, we also observed a decrease in T72-Rab8 phosphorylation rate in non de novo and treated compared to de novo patients (Supplementary Fig. 9).

**Table 3:** Correlation of clinical data with measures in EVs from clinical cohort 2 (cohort from Lille University Hospital).

		WEIGHT	HEIGHT	BMI	AGE at Sampling	AGE at ONSET	AGE at DIAG	DIAG DURATION	de novo	LEDD	UPDRS III	H&Y	Schwab & England	MMS	MoCA	DEMENTIA
Total LRRK2	Pearson’s r	**0.267**	0.1763	**0.1942**	0.06265	−0.08305	−0.08339	0.08399	−0.1826	0.07935	0.2749	0.1295	−0.04577	−0.06448	−0.05424	−0.048
	P (two-tailed)	**0.0043**	0.0629	**0.0402**	0.5098	0.5281	0.5264	0.5234	0.1626	0.5468	0.1215	0.3241	0.7284	0.4975	0.6806	0.7433
	*P*-value summary	**	ns	*	ns	ns	ns	ns	ns	ns	ns	ns	ns	ns	ns	ns
Rab8	Pearson’s r	**0.1884**	0.05904	**0.1974**	−0.09921	−0.1681	−0.1725	0.1346	−0.1862	0.17	0.1265	−0.08077	−0.07776	0.01325	0.03446	0.08787
	*P* (two-tailed)	**0.0457**	0.5363	**0.037**	0.2958	0.1993	0.1875	0.3051	0.1543	0.1941	0.4831	0.5396	0.5548	0.8892	0.7938	0.5482
	*P*-value summary	*	ns	*	ns	ns	ns	ns	ns	ns	ns	ns	ns	ns	ns	ns
TSG101	Pearson’s r	0.2167	0.1109	0.1927	−0.1093	−0.1546	−0.1552	0.1067	−0.1573	0.1965	0.1662	−0.0634	−0.09658	0.02681	0.003682	0.0833
	P (two-tailed)	0.0212	0.2444	0.0418	0.249	0.2382	0.2364	0.4173	0.23	0.1323	0.3552	0.6303	0.4629	0.778	0.9777	0.5693
	*P*-value summary	*	ns	*	ns	ns	ns	ns	ns	ns	ns	ns	ns	ns	ns	ns
pT72-Rab8	Pearson’s r	−0.01749	−0.02261	−0.02519	0.05429	**0.3094**	**0.2963**	−0.181	−0.05387	0.05604	0.06888	0.129	−0.1306	−0.1074	0.1131	0.1226
	P (two-tailed)	0.8621	0.8233	0.8035	0.5897	**0.0228**	**0.0296**	0.1903	0.6989	0.6873	0.7226	0.3527	0.3464	0.2851	0.4156	0.4277
	*P*-value summary	ns	ns	ns	ns	*	*	ns	ns	ns	ns	ns	ns	ns	ns	ns
pS910-LRRK2	Pearson’s r	0.1914	0.1253	0.07958	-0.07421	-0.4123	-0.4261	-0.2204	0.2038	-0.3836	-0.5919	-0.2628	0.1582	0.1351	0.408	−0.3649
	P (two-tailed)	0.1537	0.3574	0.5599	0.5832	0.0794	0.0689	0.3646	0.4027	0.1049	0.1222	0.277	0.5178	0.3162	0.0829	0.1499
	*P*-value summary	ns	ns	ns	ns	ns	ns	ns	ns	ns	ns	ns	ns	ns	ns	ns
pS935-LRRK2	Pearson’s r	0.007961	−0.02278	0.04586	0.04341	−0.01878	−0.03096	0.1223	−0.1131	0.1884	0.08682	−0.05449	−0.1752	0.1656	0.1049	−0.003233
	P (two-tailed)	0.9364	0.8202	0.6472	0.6633	0.8918	0.8225	0.3739	0.411	0.1684	0.6605	0.6927	0.2007	0.0945	0.3911	0.983
	*P*-value summary	ns	ns	ns	ns	ns	ns	ns	ns	ns	ns	ns	ns	ns	ns	ns
pS1292-LRRK2	Pearson’s r	−0.01756	−0.1706	0.1271	0.1708	0.1754	0.1435	0.06422	−0.3625	0.002997	0.2859	−0.1077	0.1275	0.09997	0.03103	0.03205
	*P* (two-tailed)	0.9037	0.2412	0.3842	0.2356	0.3914	0.4845	0.7553	0.0688	0.9884	0.4232	0.6005	0.5347	0.4897	0.8804	0.8846
	*P* value summary	ns	ns	ns	ns	ns	ns	ns	ns	ns	ns	ns	ns	ns	ns	ns

*LEDD* L-dopa equivalent daily dosage, *MMSE* Mini-Mental State Examination, *MoCA* The Montreal Cognitive Assessment, *UPDRS* Unified Parkinson’s Disease Rating Scale. *P < 0,05; *ns* not significant.

**Fig. 4:**
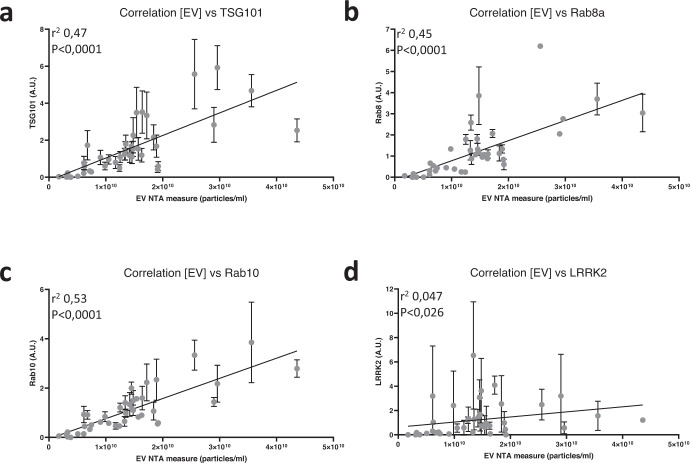
Analysis of EV concentration using nanoparticle tracking analysis and correlation to LRRK2, Rab8, Rab10 and TSG101 levels in urinary EVs isolated from PD patients and healthy controls. Extracellular vesicles were isolated from urine samples collected at the Lille University Hospital from patients and healthy controls (20 samples per group, each group consisting of 10 males and 10 females). Data EV number determined by nanoparticle tracking analysis and relative protein level for either LRRK2 (**a**), Rab8a (**b**), Rab10 (**c**), or TSG101 (**d**) determined by western blotting (triplicate measures per sample) were plotted for each individual and a Pearson’s correlation analysis performed. The resulting trendline is given as well as the *r*² and *P*-value of the Pearson’s correlation. Error bars represent standard error of the mean (s.e.m.). [EV] EV concentration, NTA nanoparticle tracking analysis.

In light of the observation that total Rab8a and TSG101 values are higher in iPD subjects compared to HCs, we reasoned that this could be due to either increased release of EVs in urine of iPD subjects or to an increased Rab8 and TSG101 content in urinary EVs. To verify these 2 scenarios, we decided to quantify urinary EV concentrations via nanoparticle tracking analysis (NTA) and correlate this to measures of TSG101, Rab8a and LRRK2 from a subset of urine samples (20 HCs and 20 iPDs, 10 male and 10 female each). We also included in the analysis another LRRK2 substrate, Rab10 (Supplementary Figs. 10, 11 show the optimization of detection conditions and linearity of quantification). Using a Pearson’s correlation analysis, we found a significant correlation of the EV number with TSG101, Rab8a and Rab10 with *r*² values above 0.45 and *p*-values below 0.0001, while correlation of EV numbers with LRRK2 was markedly lower with *r*² value of 0.047 and *p* values 0.026 (Fig. 4). An accessory result of this experiment is that we measured additional EV markers in these samples, including Alix and the tetraspanin CD9 and assessed their correlation to the TSG101 values (Supplementary Fig. 12). As can be appreciated from this figure, the correlation between TSG101 and Alix as well as the correlation between TSG101 and CD9 is good, with Pearson’s coefficients of 0.8477 and 0.6383, respectively, and *p*-values below 0.0001. This result further justifies the choice of TSG101 as the the primary uEV loading control used throughout this study.

**Fig. 5:**
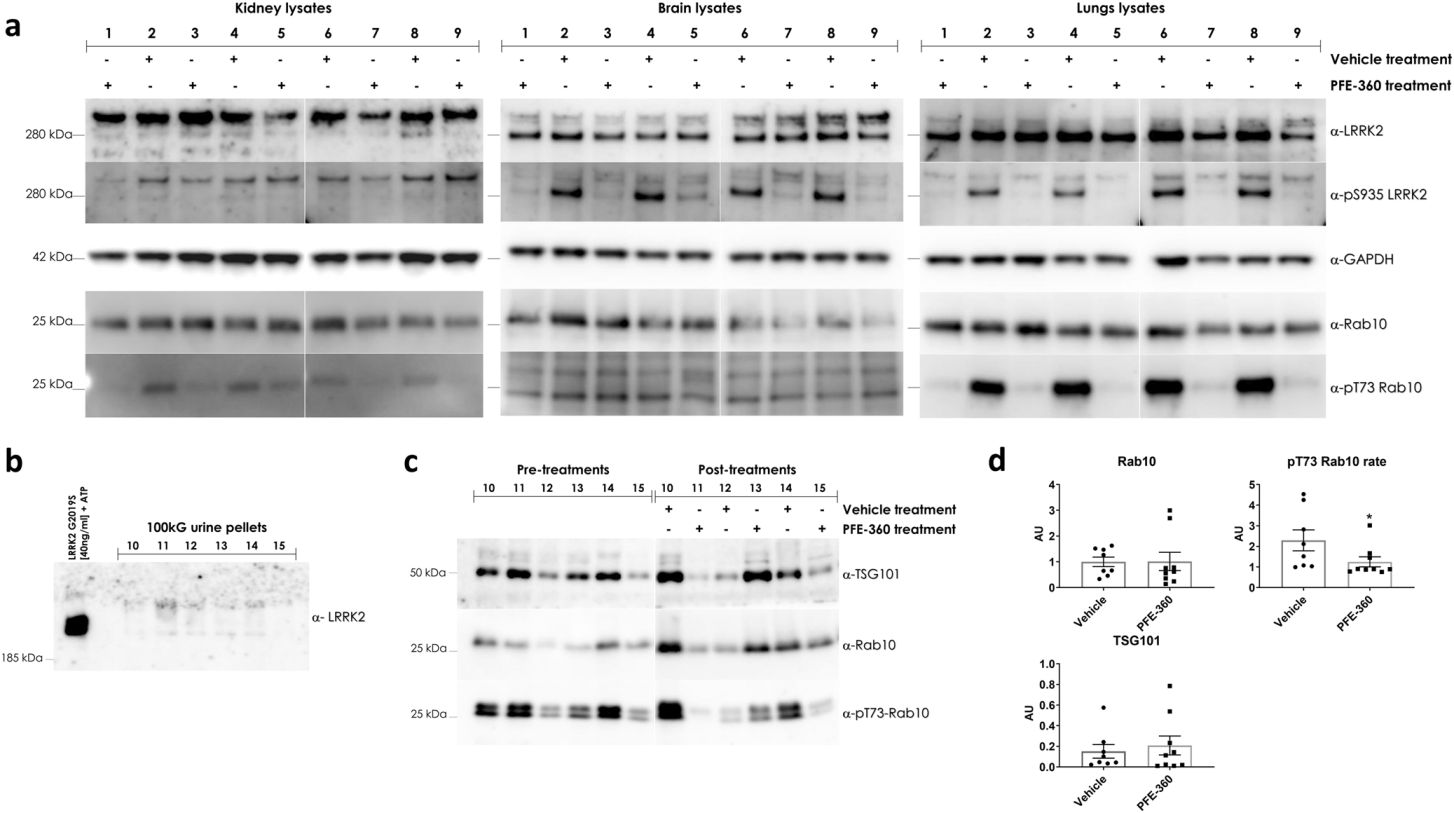
Detection of LRRK2 & Rab10 in rat organ lysates and in urinary EVs after solvent and LRRK2 kinase inhibitor treatment. **a** Western blot detection of total LRRK2, pS935-LRRK2, total Rab10, pT73-Rab10 and TSG101 in kidney, brain and lung lysates of rats treated with PF360 or solvent (7.5 mg/kg, 6 h). Note that the blots in kidney yield a non-specific band at a higher molecular weight. **b** Detection of total LRRK2 in normal rat uEVs, showing very low LRRK2 levels. **c** Western blot detection of total total Rab10, pT73-Rab10 and TSG101 in rat uEVs, both pre-treatment and after administration of PF360 or solvent (7.5 mg/kg, 6 h) (triplicate measures per sample). **d** quantification of (**c**). The values of phosphorylation ‘rate’ are indicative of the proportion of protein that is phosphorylated at the given site, see materials and methods. Statistical differences were tested using a two-tailed Mann–Whitney test. Error bars represent standard error of the mean (s.e.m.). *P < 0.05 (*N* = 8–9).

**Fig. 6:**
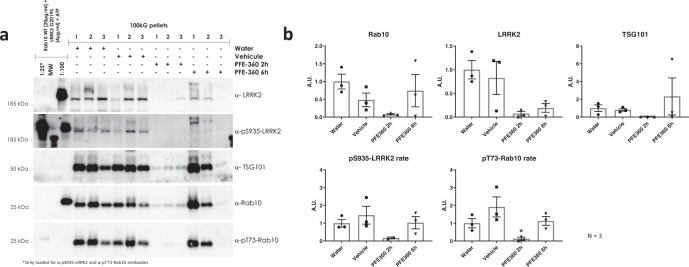
Effect of acute LRRK2 kinase inhibition on phospho-LRRK2 and phospho-Rab in urinary EVs of macaques. Urine was collected from macaques after different treatments and urinary EVs were isolated as described in materials and methods. **a** Western blot detection of total LRRK2, pS935-LRRK2, total Rab10, pT73-Rab10, and TSG101 in urinary EVs of NHPs treated with PF360 or solvent (5 mg/kg p.o., 2 and 6 h) (quadruplicate measures per sample). Note that two different dilutions of the calibration standard were used and these are separated by a molecular weight marker containing a band that is detected by the anti-pS935-LRRK2 antibody. **b** Quantification of (**a**). The values of phosphorylation ‘rate’ are indicative of the proportion of protein that is phosphorylated at the given site, see materials and methods. Statistical differences were tested using a Kruskal-Wallis test followed by a Dunn’s post-hoc test using the vehicle group as control. Note that values for pS935-LRRK2 were below the linear range of quantification at the 2 h time point and statistical anlaysis was not possible. Error bars represent standard error of the mean (s.e.m.). *P < 0,05 (*N* = 3). MW molecular weight marker.

Besides the potential for measures of LRRK2 and its Rab substrates as biomarkers in PD, the phosphorylation of LRRK2 and Rab proteins are also proposed biomarkers of pharmacological response to treatment with LRRK2 kinase inhibitors. To prepare testing for this, we first verified the detection of the concerned epitopes in uEVs of mice, rats and non-human primates (NHP, rhesus macaques). Using the sensitive western blot approach described in materials and methods, we found that detection of both total LRRK2 and pS935-LRRK2 is close to or under the detection limit in rodent urinary EVs. Nevertheless, using LRRK2 KO rats, we were able to show that the band detected in rat urine with anti-LRRK2 antibodies is indeed the LRRK2 band (Supplementary Fig. 13). By contrast, we show that Rab and phospho-Rab levels are readily detected (Supplementary Fig. 13). In uEVs of NHPs, detection of total LRRK2, pS935-LRRK2, and Rabs and phospho-Rabs was confirmed (Supplementary Fig. 14). We therefore decided to test for the effect of acute LRRK2 kinase inhibition on Rab phosphorylation in urinary EVs from rodents and on LRRK2 and Rab phosphorylation in urinary EVs from NHPs. Upon testing, we found a significant reduction in phospho-Rab in rat urinary EVs of treated animals (for urine collected for 6 h after inhibitor injection (cumulative collection method), Fig. 5). For NHPs, proposed pharmacodynamic biomarkers for LRRK2 inhibition were comparisons of baseline levels (vehicle or water) to those levels measured after drug treatment. It should be noted that collection of the urine samples from the NHPs was performed by emptying the bladder using a catheter at the start of the experiment and collecting again at the given time point. For this reason, overall EV levels were low at the 2 h time point, due to the short time frame for EVs to accumulate in the urine. Also, pS935-LRRK2 was very low at the 2 h time point, outside of the linear range, and statistics could not be performed for this measure. Another caveat to mention is that due the reduction and refinement principle of animal experimentation, these experiments were carried out with a relatively low number of individuals (*N* = 3) Nevertheless, Rab and phospho-Rab detection was within the linear range of detection for all time points and we found significantly reduced Rab phosphorylation rate at 2 h post-administration (PFE-360 5 mg/kg p.o.), not at 6-h post-injection (Fig. 6).

## Discussion

The first focus of the present study was to evaluate LRRK2 and related epitopes as a potential biomarker of PD, via the analysis of patient urinary EVs. The first hypothesis in this study was that LRRK2 phosphorylation rates at S1292 would be increased in patients, as an indicator that PD patients would display a gain of kinase function. Indeed, S1292 is an autophosphorylation site of LRRK2 and the phosphorylation at this site is considered to be a marker of steady state LRRK2 kinase activity in tissues^9^. Phospho-Ser1292-LRRK2 is also increased in pathogenic mutant variants of LRRK2^20^, although there are some exceptions such as for the lower penetrance variant G2385R^21^. In addition, Fraser and colleagues have reported that pS1292-LRRK2 levels are increased in PD patients compared to healthy controls, both for idiopathic PD and for G2019S carriers^17,18^.

### Analysis of LRRK2/Rab in uEVs of PD patients

Our first experiment was performed to compare results with the previous report by Fraser *et al* ^17^, *i.e*. using similar sample groups (male individuals, including healthy controls, idiopathic PD patients, healthy LRRK2 G2019S carriers and LRRK2 G2019S carriers with PD) and with similar modes of sample processing and signal detections/quantification. In line with the report by Fraser *et al* ^17^, pS1292-LRRK2 is elevated in EVs of LRRK2 G2019S carriers, both with PD and non-manifesting carriers. Also in line with Fraser *et al*. is our observation that total LRRK2 is elevated in EVs isolated from LRRK2 G2019S carriers, both with PD and non-manifesting carriers. By contrast, we observed no significant increase in PD samples of the pS1292-LRRK2, neither in raw phosphorylation levels, nor in phosphorylation rates relative to total LRRK2 or to the EV marker TSG101. While these measures are all indicative of phosphorylation of LRRK2 at S1292, we opted to include each of these specific values as each represents something different: volume normalized pS1292-LRRK2 representing raw phosphorylation values, exosome marker normalized pS1292-LRRK2 for the ratio with TSG101 and phosphorylation rate for the ratio with total LRRK2. Note that in an independent group of samples collected at the Lille University Hospital Center, which included 7 G2019S carriers with PD, we also observed the increase of total-LRRK2 levels in G2019S carriers relative to non-carriers (Fig. 3), lending inherent validity to our observations. Therefore, our results do not confirm the hypothesis that pS1292-LRRK2 level in urinary EVs is predictive of the parkinsonian phenotype, neither for idiopathic PD nor for LRRK2-G2019S-PD. Rather, urinary EV levels of both total LRRK2 and pS1292-LRRK2 are increased in G2019S carriers compared to non-carriers. Interestingly, in our correlation analysis of this experiment we find that S1292-LRRK2 phosphorylation rates correlate to the Hoehn & Yahr stage as well as to the Schwab and England consensus rating, suggesting that this parameter is related to disease severity. However, as a caveat it should be noted that the LRRK2-PD group showed a longer disease duration and higher Hoehn & Yahr score. We also observed that raw pS1292-LRRK2 levels correlate to age and age at onset, while TSG101 correlates to total levodopa equivalent dose and the Schwab and England consensus rating and total LRRK2 correlates to MoCA.

A second hypothesis that we wanted to test, was that pT72-Rab8a levels may be increased in patient urinary EVs. Again, this is based on the idea of a gain of kinase function in PD given that Rab8a is a LRRK2 kinase activity dependent phosphorylation target^22^. With the Lille cohort samples, we observed no significant increase in pT72-Rab8a in PD compared to healthy controls, in line with our observations made with pS1292-LRRK2 in both tested cohorts. Note that the pT72-Rab8a data is obtained with an antibody raised against a phospho-Rab8a sequence, however the antibody nevertheless cross-reacts with other phospho-Rabs at their respective homologous phospho-sites, therefore it should be considered as a pan-phospho-Rab signal^23,24^. The lack of an increase in phospho-Rab8 phosphorylation rate in PD uEVs is intriguing as phosphorylation of Rabs has been assessed in other sample types such as patient derived neutrophils, where an increase in Rab10 phosphorylation rate was observed in patients carrying the LRRK2 G2019S or VPS35 D620N mutations compared to healthy controls^25^. In addition, analysis of post-mortem brain showed an increase in T73-Rab10 phosphorylation in nigrostriatal dopaminergic neurons of iPD patients compared to age-matched controls^26^. Our findings therefore suggest that Rab phosphorylation is regulated differently in urinary EVs compared to neutrophils and brain.

After the hypotheses of increased S1292-LRRK2 or Rab phosphorylation, a third hypothesis can be formulated based on the S910 and S935 phosphosites of LRRK2. Indeed, the S910 and S935 sites are well established as being dephosphorylated in the presence of several disease mutant forms of LRRK2 (with notable exceptions, including the G2019S mutant)^8^ as well as in PD brain^27^. Our data suggest that this hypothesis is confirmed, with reductions observed for S910-LRRK2 and S935-LRRK2 phosphorylation rates in urinary EVs of PD patients compared to healthy controls.

Outside of pre-existing hypotheses, other parameters measured allow us to derive some conclusions from our data. For instance, we have observed in our second cohort that the EV marker TSG101 is increased in PD uEVs. Related to this, we observe as well that total Rab8a levels are significantly increased in urinary EVs of idiopathic PD. These are intriguing findings suggesting either that higher EV concentrations are found in PD urine or that urinary EVs in PD patients express higher levels of these 2 markers. A correlation analysis for these markers and EV concentrations determined via nanoparticle tracking analysis on a subset of samples (Fig. 4) points to the former hypothesis as samples with higher EV concentrations display higher TSG101, Rab8a and Rab10 levels. Interestingly, while the correlation between EV concentration and LRRK2 levels was statistically significant, the P-value was higher than for the other values and the slope flatter, suggesting that there is some flexibility for LRRK2 levels to fluctuate within EVs.

With these findings in mind, one important aspect is to determine how such measures could be implemented in the clinical setting. One of the ways of assessing this is to perform a receiver operator characteristic (ROC) analysis. For the four measures that are modified between PD and healthy controls, the areas under the ROC curve are significantly different from the random hypothesis and they range from 0,6047 to 0,6925 (Supplementary Fig. 7). Arguably, these values are on the low side for implementation in clinical trials; however, it should be noted that while our western blot approach shows good quantitative linearity, higher throughput assays would need to be developed for routine applications and these may be expected to be of greater precision. Also, by looking at subsets of PD patients, our data suggest that some of the parameters tested such as Rab8a, TSG101, pS910-LRRK2, pT72-Rab8a are altered in specific patients groups such as de novo compare to non de novo or patients with MCI compared to those without, indicating a potential to stratify different patients groups. Potential additional parameters to explore in future studies are alternative normalization methods. Our study used volume normalization to analyze samples (i.e. samples were tested at equal volumes corresponding to equal volumes of the originally processed urine) and EV loading was quantified primarily using the EV marker TSG101. While we found a good correlation between TSG101 and 2 additional EV markers (Alix and CD9) in a subset of our samples (Supplementary Fig. 12), and given that TSG101 is a robustly validated EV marker^28,29^, it is distinctly possible that the use of alternative EV markers or the use of alternative analysis conditions (for instance protein content normalization rather than volume normalization) may reveal differences between healthy controls and patient groups with greater sensitivity or precision. Further work on LRRK2 and Rab release mechanisms in uEVs, taking into account additional normalization methods and testing of samples from additional clinical cohorts would be useful in future studies.

While it is difficult to pinpoint exactly what the mechanism may be that leads to differences in the LRRK2 measures in female compared to male study participants, we can underline that gender differences are documented in idiopathic PD as well as in LRRK2-PD. For instance, men have a twofold higher risk of developing disease. However, mortality rate is higher and disease progression faster in women^30^. Interestingly, there is a higher female prevalence for the LRRK2 G2019S mutation, although no gender difference is observed for the G2385R variant^31^. By contrast, another study finds that diversity of phenotype between men and women is lower for LRRK2 G2019S mutation carriers than for idiopathic PD patients^31^. Further work will be necessary to better understand the differences in LRRK2 markers in uEVs of men compared to women.

### Analysis of LRRK2/Rab in uEVs in rats and non-human primates treated with LRRK2 kinase inhibitors

In a final set of experiments, we aimed to assess pharmacodynamic markers of LRRK2 kinase inhibition in rodent urine. This section of the study yielded some technical challenges. Indeed, we demonstrate that the detection of LRRK2 in rat urinary EVs is more complicated compared to LRRK2 detection in human urinary EVs, with low detection of total LRRK2 and virtually undetectable pS935-LRRK2, precluding the possibility to quantify these levels. Therefore, the use of LRRK2 as a pharmacodynamic marker in rat urine may be possible in the future on condition of significantly concentrating EV volumes (at least 5-fold) and/or increasing detection sensitivity. We also tested Rab10 as pharmacodynamic markers and found a reduction in pT73-Rab10 in animals treated with the LRRK2 kinase inhibitor PFE-360 that was shown to be active (shown by the dephosphorylation of LRRK2 at S935 in kidney, lung, and brain). In NHPs, LRRK2 S935 phosphorylation levels in urinary EVs have a tendency to reduce after PFE-360 LRRK2 inhibitor treatment, as do total LRRK2 levels, while Rab10 phosphorylation rates are significantly reduced at 2 h after acute LRRK2 kinase inhibitor treatment. It should be noted that the method of collecting urine directly from the bladder via a catheter (done here at 2 and 6 h post-administration) allow us to observe that the changes in LRRK2 or Rab10 occur very rapidly. Further work looking at the dynamics of these changes would be most useful to begin to define best practices for using LRRK2-Rab measures in clinical trials for LRRK2 kinase inhibitors. Overall, these data are consistent with the potential of pT73-Rab10 in urinary EVs as a pharmacodynamic marker of LRRK2 kinase inhibitors.

The observations made during this study confirm the potential use of LRRK2 or its Rab substrates as biomarkers for PD or for pharmacodynamic response to LRRK2 kinase inhibitors. In particular, our study reveals that Rab8a is increased and that phosphorylation rates of LRRK2 at S910 and S935 are reduced in urinary EVs of PD patients. Interestingly, when analyzing male and female groups of our cohort, these changes were found to be driven primarily by the female groups. Using rodents and non-human primates, we find that dosing animals with LRRK2 kinase inhibitors leads to reduced Rab10 T73 phosphorylation, found in urinary EVs of NHP and rodents. Further work is now warranted to assess additional activity markers within the LRRK2-Rab pathway and to develop high throughput multiplex assays for these measures in order to begin to apply these in clinical settings.

## Materials and methods

### Ethics

The study of urinary EVs in PD was conducted in using samples from two different cohorts.

The first cohort of samples was obtained from the Michael J. Fox Foundation (MJFF) LRRK2 Cohort Consortium (LCC) biobank. The LCC study was established in 2009, when the MJFF LCC brought together investigators from North America, Europe, North Africa, and Asia to study individuals with mutations in the LRRK2 gene. Ethical review and approval of the LCC biobank were not required for the de-identified sample analysis in accordance with the local legislation and institutional requirements. The patients/participants provided their written informed consent to participate in this study. All participants included are male: 36 G2019S LRRK2 mutation carriers (20 with PD and 16 without PD) and 39 non carriers (20 with PD and 19 without PD). These participants were selected to match with our second cohort (*cfr. infra*) as closely as possible.

The second cohort followed at the Lille University Hospital Movement Disorders Department included 120 participants (67 diagnosed with PD and 53 healthy controls) followed at the Lille University Movement Disorders Department, and enrolled between June 2015 and October 2017. Each subject provided written consent to participate prior to inclusion. The protocol (reference CONVERGENCE cohort CPP 2008-A00219-42) was approved by the independent ethics committee for northwestern France (Comité de Protection des Personnes Nord-Ouest IV).

### Collection and storage of human urine samples

All PD patients fulfilled the Movement Disorders Society (MDS) diagnostic criteria of clinically established PD^32^. Fourteen PD patients were at de novo stage (dopaminergic treatment-naive and less than 3 years of disease duration) and their medical records were verified for 5 years to ensure the accuracy of the diagnosis. Inclusion criteria for healthy controls were: age > 18 years, lack of familial history of PD, lack of parkinsonian syndrome at inclusion examination and lack of significant cognitive impairment objectified by a score > 25/30 at the Mini-Mental State Examination (MMSE). None of the control subjects were diagnosed with PD to date. All the participants were genotyped for the G2019S mutation of the LRRK2 gene using standard genotyping assays^33,34^ and 7 PD patients were carrying the mutation. At inclusion, demographic data, medical and PD familial history, PD characteristics, and medications were collected for each participant. Levodopa equivalent daily dose (LEDD) was calculated as described^35^.

Demographic and relevant clinical data for the LCC and Lille cohorts are presented in Tables 1 and 3, respectively, of the results section.

Human urine samples collected during the visit of subjects to the Lille University Hospital were completed with 5% of Tris-EGTA (1M-40mM) upon collection, sampled into 40 mL aliquots and kept at 4 °C until freezing (all samples were frozen at less than 6 h after collection). All urine samples were stored at −80 °C until processing.

### Antibodies

Information on the antibodies used this study, including detection epitope, clone reference, species and supplier are provided in Table 4. Please note that the pT72-Rab8a antibodies are reported to cross react with other LRRK2 phosphorylated Rab proteins and the signal obtained represents therefore a cumulation of multiple Rab phosphorylations^23^. Also, TSG101 was selected as the loading control for the uEV samples for the majority of the analyses of the present paper, as it is a protein known to be present in exosomes/EVs that has also been shown to be closely correlated to extracellular vesicle numbers^28^. In general, the selection of a protein present in uEVs as a loading control shows strong rationale as suggested by the recently published position paper on uEVs^29^. Besides the commonly used TSG101 protein, other protein EV markers exist such as the protein Alix or proteins of the tetraspanin family such as CD9, CD63. See “Results” section for comparisons between different markers.

**Table 4:** List of antibodies used in the study, including clone information, species in which the antibody was raised and supplier.

Detection epitope	Clone	Species	Supplier
Total LRRK2	MJFF2 (C41-2)	Rabbit	Abcam (ab133474)
Total LRRK2	UDD3 30(12)	Rabbit	Abcam (ab133518)
Total LRRK2	N241A/34	Mouse	UC Davis/NIH NeuroMab Facility (75-253)
Total LRRK2	D18E12	Rabbit	Ozyme (#13046)
Total LRRK2	MC.028.83.76.242	Mouse	BioLegend (SIG-39840-100)
pS910-LRRK2	UDD1 15(3)	Rabbit	Abcam (ab133449)
pS935-LRRK2	UDD2 10(12)	Rabbit	Abcam (ab133450)
pS955-LRRK2	MJF-R11 (75-1)	Rabbit	Abcam (ab169521)
pS973-LRRK2	MJF-R12(37-1)	Rabbit	Abcam (ab181364)
pS1292-LRRK2	MJF-R19-7-8	Rabbit	Abcam (ab203181)
Total Rab8a	D22D8	Rabbit	Ozyme (#6975)
pT72-Rab8a*	MJF-R20	Rabbit	Abcam (ab230260)
pT72-Rab8a*	Polyclonal	Rabbit	MRC PPU (E8263)
Total Rab10	D36C4	Rabbit	Ozyme (#8127)
pT73-Rab10	MJF-R21	Rabbit	Abcam (ab230261)
TSG101	4A10	Mouse	Life Technology (MA1-23296)
Alix	3A9	Mouse	Ozyme (#2171S)
CD9	EPR2949	Rabbit	Abcam (ab92726)
CD63	H-193	Rabbit	Santa Cruz (sc-15363)
GAPDH	Polyclonal	Rabbit	Sigma-Aldrich (G9545)
Beta-actin	AC-15	Mouse	Sigma-Aldrich (A5441)

### Housing, handling and pharmacological treatment of rats and non-human primates

Rodents Housing and handling of rodents were done in compliance with national and international guidelines. Sprague Dawley rats (Janvier labs, Le Genest-Saint-Isle, France) were used in the pharmacological treatment experiments. LRRK2 KO rats (HsdSage: LE-Lrrk^tm1sage^)^36^ and wild type Long Evans hooded rats were also used in experiments to confirm LRRK2 detection in rat uEVs and were ordered from Horizon Discovery (currently, Envigo, Lafayette, CO, USA). All animal procedures were approved by the Ethical Committee of the French Ministry of National Education, Higher Education and Research (reference #4271-2015102712365542). Pharmacological treatment of rats was done via intraperitoneal injection. Compound injected is PFE-06685360 (PFE-360 for short, generous gift from Dr. T. Lanz, Pfizer, USA). Injected dose of PFE-360 in the rats is 7.5 mg/kg.

Non-human primates: All husbandry, housing, and experimental procedures were conducted at Suzhou Xishan Zhongke Drug R&D Co., Ltd. (Suzhou, PRC) and under an IACUC animal use protocol specific for this study and approved by Suzhou Xishan Zhongke Drug R&D Co., Ltd. (IACUC number, IP171125PD11). All methods were performed in accordance with the relevant guidelines and regulations. Female cynomolgus macaques averaged 10 years of age and weighed an average of 4.45 kg at the start of the study.

#### Administration of PFE-360

Administration of PFE-360: Animals were administered PFE-360 (5 mg/kg), vehicle or sterile water by oral gavage using a dose volume of 10 ml/kg. Seven days were allowed between single administrations to allow for a complete washout. The vehicle was composed of 1.25% (w/v) hydroxypropyl cellulose / 0.05% (w/v) docusate Sodium in sterile water.

### Collection and storage of urine samples from rats and non-human primates

#### Rats

Rat urines where collected over a 7–8 h period in metabolic cages. In these cages, urine produced by the rodents is sorted into urine collection vessels that are prepared to contain 100 µL of Tris-EGTA buffer (1 M Tris pH7.4, 40 mM EGTA) at the beginning of collection. The urine collection vessels are placed in a cooling module for the duration of the collection to ensure that collected urine is maintained cool. At the end of the collection period, urines are complemented with Tris-EGTA to a final concentration of 5% and sampled into 1.5 mL aliquots. Urine is then stored at −80 °C until processing.

#### Non-human primates

Under anesthesia (Zoletil/atropine (6/0.04 mg kg^−1^, IM)), urine (via acute catheterization to the bladder via the urethra) was collected from the animals before treatment (baseline), and again 2 and 6 h after dosing with PFE-360. Urine samples were aliquoted into Eppendorf tubes and immediately buried in dry ice and subsequently stored at −80 °C.

### Isolation of urinary extracellular vesicles via differential centrifugation

Protocols for processing of urine are based on previously published reports^6,14,37–39^ and depicted schematically in Fig. 1a. In brief, urine samples were stored at −80 °C. In order to analyze the samples, either ‘neat’ (i.e. without further processing) or fractionated, samples were submitted to a rapid thawing step by placing samples in a water bath at 42 °C with regular mixing. In order to obtain specific fractions, urine is submitted to sequential centrifugation steps. Low-speed centrifugation steps are to remove large particles and aggregates of biomolecules (‘clearing’ step at 500 g) or to remove large vesicular structures (10.000 g step). A fraction of small extracellular vesicles (EVs) enriched in exosomes are sedimented in an ultracentrifugation step at 100.000 g for 2 h. EV pellets are then rinsed and resuspended in PBS and repelleted in an ultracentrifugation step at 100.000 g for 2 h. Finally, EVs were resuspended in 15 µL of Tris-Triton buffer (20 mM Tris-HCl, 150 mM NaCl, 1 mM EDTA pH8, 10% Glycerol, 1% Triton) for western blot analysis or 15 µL PBS for nanoparticle tracking analysis per 1 mL of initial urine volume. All sample buffers contained protease (cOmplete™ Protease Inhibitor Cocktail, Roche®) and phosphatase (PhosSTOP™, Roche®) inhibitor cocktails.

### Western blot analysis

#### Protein calibration standards

First, calibration standards to be included in each SDS-PAGE gel alongside regular EV samples were generated and validated. These standards included (i) pool of urinary EV samples, generated from the samples of the study, (ii) a truncated recombinant LRRK2 (Life technologies) that had been submitted to autophosphorylation^40^ and spiked into the pool of urinary EVs (at 100 pg/10 µl) and (iii) full length recombinant LRRK2 (Life technologies), both unmodified and autophosphorylated (100 pg/10 µl). The validation of these calibration standards is given in Supplementary Fig. 1.

### Tissue lysates

Rats were anaesthetized with ketamine (100 mg/kg)/xylazine (10 mg/kg) and then transcardially perfused with HBSS. Tissues including brain, kidney, lungs were rapidly dissected out and snap frozen using liquid nitrogen. Tissues were stored at −80 °C until use.

To make homogenates for use in western immunoblotting, tissues were ground with mortar and pestle in liquid nitrogen and homogenized in 5 volumes of buffer medium (10 mM Tris-HCl, 1 mM EDTA, and 0.25 M sucrose pH 7.4), containing a Complete® protease inhibitor cocktail (Roche Molecular Biochemicals, Indianapolis, IN, USA), using a dounce homogenizer. An aliquot of the resultant homogenates was stored at −80 °C.

### Western blotting and quantification

The protein content of cell lysates was determined using the bicinchoninic acid (BCA) protein determination assay (Pierce Biotechnology) or the Bradford method (Thermo Scientific) with bovine serum albumin (BSA) as the standard. After addition of LDS sample buffer (containing sample reducing agent) and boiling, equal volumes of samples (10 µl) were resolved by electrophoresis on NuPAGE 3-8% Tris-Acetate gradient gels, 4–12% Bis–Tris gradient gels, 4–20% Tris-Glycine gradient gels or 12.5% SDS gels (LifeTechnologies). The majority of samples were tested in quadruplicate, although some samples were tested in triplicate as indicated in the figure legends. 15-well gels were used, with 11-12 wells reserved for samples, one well for a molecular weight marker and 2–3 wells reserved for calibrators. Separated proteins were transferred to PVDF (Bio-Rad) or nitrocellulose (Amersham) membranes, and non-specific binding sites were blocked for 60 minutes in Tris-buffered saline containing 0.05% Tween-20 (TNT) and 5% non-fat milk or 5% BSA. After overnight incubation at 4 °C with the appropriate antibodies, blots were washed four times with TNT. After incubation with the secondary antibodies, blots were washed again. Bands were visualized using enhanced chemiluminescence (Amersham ECL Prime, Cytiva) that was acquired using the image analyzer Imager 600 (GE Healthcare Bio-Sciences). All blots were processed in parallel and derive from the same experiment.

Specific attention was taken for those samples where different epitopes of the same protein were detected in the same samples, specifically for detection of total and phosphorylated forms of LRRK2, Rab8, or Rab10. We opted to detect the total and phospho-epitopes in separate blots, rather than analyze both on the same blot using a stripping technique between detections or using fluorescence detection in different detection channels. The advantage of this repeated blots approach is that we could benefit from a sensitive detection in a linear range and also that the samples are not altered between the detection of the phospho- and total epitope. Indeed, we have previously successfully used multiplex detection with separate fluorescent secondary antibodies for the total and phospho-signal^41,42^, however this was for cell lysates with high exogenous expression of LRRK2. When we tested this technique on uEV samples, most were in the low to no detection range (data not shown) and it was therefore not applicable. We also opted not to use the blot stripping technique as it has the major disadvantage that there is a treatment of the blot between detecting both signals that can lead to important biases including residual signal in the case of understripping or antigen reduction in the case of overstripping, as discussed in a recent report on technical considerations for western blotting^43^. We validated our repeated blots approach by verifying the linear ranges of detection of the different epitopes. Importantly, to reduce the variability of the quantification, we also took into account the TSG101 loading control signal (an EV marker) from the same blots as those detecting the corresponding total or phospho-epitope of LRRK2 or Rab proteins (see below). Taken together, this repeated blots approach allowed us to achieve sensitive and quantifiable detection in a linear range (see results section for linearity testing). The number of gels run for the study are 53 gels for the LRRK2 cohort consortium study, 156 gels for the study with the samples from the Lille cohort, 55 for the rat study, 8 for the non-human primate study and 18 for the correlation analysis of the LRRK2 and Rab measures with the nanoparticle tracking analysis. This is a total of 290 gels.

Densitometric analysis of the bands on the blot images was performed using Imagequant or ImageJ software. Values representing abundance levels of proteins or protein phosphorylation rates were obtained by a series of calculations (note that the values of phosphorylation ‘rates’ are indicative of the proportion of protein that is phosphorylated at the given site, see below). First, for each sample and for each calibration standard, average values for the replicate values of each detected epitope were calculated. Replicates where detection was under the detection limit were not included in this averaging. Values are given of single epitopes when appropriate such as for total LRRK2, total Rab, total TSG101; these values are considered volume normalized as equal volumes of sample are loaded in each lane (10 µl), corresponding to equivalent amounts of originally processed urine. When LRRK2 or Rab epitopes are expressed relative to a marker or loading control such as TSG101 in the EV samples and GAPDH in tissue samples, a ratio is calculated of the LRRK2 or Rab values over the marker or loading control. To obtain phosphorylation rates for LRRK2 or Rab, the ratio of the phosphorylated epitope to the marker/loading control is divided by the ratio of the total epitope to the marker/loading control (for example, for a LRRK2 phosphorylation rate in EVs = (phospho-LRRK2/TSG101)/(total-LRRK2/TSG101). This last calculation takes into account potential differences in loading between the phospho and total epitopes as these are loaded onto separate gels. Values thus obtained are then expressed relative to the protein calibration standards (see above). All values are normalized to the control group whose average is set to 1. A flow chart with the overview of the steps of sample processing, sample western blot analysis and quantification is given in Supplementary Fig. 2.

### Transmission electron microscopy

EV isolates were then processed for electron microscopy adapted from a previous protocol^44^. Briefly, the EV pellet was resuspended and fixed in a solution of 2% paraformaldehyde in 0.1 M phosphate buffer, pH 7.4, overnight at 4 °C. To detect LRRK2 immunoreactivity, samples collected on carbon film coated 400 mesh nickel grids (EMS, Fort Washington, PA, USA) were treated using an immunogold procedure described previously^44^. Briefly, after a preliminary treatment with PBS + glycine 50 mM and a blocking step in PBS containing 1% bovine serum albumin (10 min at RT), the grids were floated on a drop of the following reagents and washing solutions: (1) rabbit anti-LRRK2 UDD3 30(12) (1:100, Abcam, Paris, France) and mouse anti-LRRK2 N241A/34 (1:50, Antibodies Incorporated, Davis, CA, USA) in PBS + 1% bovine serum albumin for 60 h at 4 °C, (2) PBS + 0,1% Bovine serum albumin to remove excess antibodies (three times for 10 min), (3) colloidal gold (18 nm)-labeled goat anti-rabbit immunoglobulins (Jackson ImmunoResearch) 1:20 and colloidal gold (6 nm)-labeled goat anti-mouse immunoglobulins in TBS for 90 min at RT, (4) PBS (three times for 10 min) and (5) PBS + 1% glutaraldehyde. The sections were then counterstained with uranyl acetate and methylcellulose before observation. The specificity of the LRRK2 antibodies used has been discussed previously^19^. Samples were examined with a Zeiss transmission electron microscope 902 (Leo, Rueil-Malmaison, France) and images were acquired using a Gatan Orius SC1000 CCD camera (Gatan France, Grandchamp, France).

### Nanoparticle tracking assay (NTA)

Analysis in NTA of urine purified EVs were always performed directly after isolation via differential centrifugations. UCF pellets resuspended in PBS were diluted once more in PBS to attain a concentration range between 10^7^ and 10^9^ particles/mL and injected under controlled flow in the microfluidic chamber of a NanoSight NS300 (Malvern Panalytical, Palaiseau, France). Each sample was captured 5 times for 60 s with a Camera level set at 15, and analyzed with a Detection Threshold at 4.

### Statistics

The number of independent experiments and sample size are indicated in the figure legends, data are expressed as mean±s.e.m. Statistical analyses were conducted using the GraphPad Prism 7 software (GraphPad Software, San Diego CA, USA). Tests carried out include the Mann–Whitney test (when comparing 2 groups) or the Kruskal–Wallis test followed by a Dunn’s post hoc test (when testing 3 or more groups). To test correlations between measures in EVs and clinical parameters, Pearson’s correlations were carried out using GraphPad Prism 7 software. Significance levels in the figures are denoted as ns, *, **, ***, **** corresponding to not significant (*P* > 0.05), *P* < 0.05; *P* < 0.01; *P* < 0.001 and *P* < 0.0001, respectively.

### Reporting summary

Further information on research design is available in the Nature Research Reporting Summary linked to this article.

## Supplementary information

Supplemental material

Uncropped western blot images

Reporting Summary

## Data Availability

Data used in the preparation of this article were obtained from the MJFF-sponsored LRRK2 Cohort Consortium (LCC). For up-to-date information on the study, visit www.michaeljfox.org/lcc). For the data presented in graphs of this paper, excel files with individual values of each tested sample can be provided upon request. Requests should be directed to Dr. Jean-Marc Taymans jean-marc.taymans@inserm.fr and Dr. Marie-Christine Chartier-Harlin marie-christine.chartier-harlin@inserm.fr.
