# Supplementary material for: Alterations in the LRRK2-Rab pathway in urinary extracellular vesicles as Parkinson’s disease and pharmacodynamic biomarkers
Source: NPJ Parkinsons Dis. 2023 Feb 7;9:21. doi: 10.1038/s41531-023-00445-9 (PMC9905493; doi:10.1038/s41531-023-00445-9)
Supplement: Supplementary file 1 — Supplemental material [file 41531_2023_445_MOESM1_ESM.docx]

**Supplemental data for the manuscript:**

**Alterations in the LRRK2-Rab pathway in urinary extracellular vesicles as Parkinson’s disease and pharmacodynamic biomarkers**

*Author list:*

Jean-Marc Taymans^1,*^, Eugénie Mutez^1,2^, William Sibran^1^, Laurine Vandewynckel^1^, Claire Deldycke^1^, Séverine Bleuse^2^, Antoine Marchand^1^, Alessia Sarchione^1^, Coline Leghay^1^, Alexandre Kreisler^2^, Clémence Simonin^1,2^, James Koprich^3^, Guillaume Baille^1^, Luc Defebvre^1,2^, Kathy Dujardin^1,2^, Alain Destée^1,2^, Marie-Christine Chartier-Harlin^1,*^

Supplementary Figures 1-14

Supplementary Tables 1 and 2


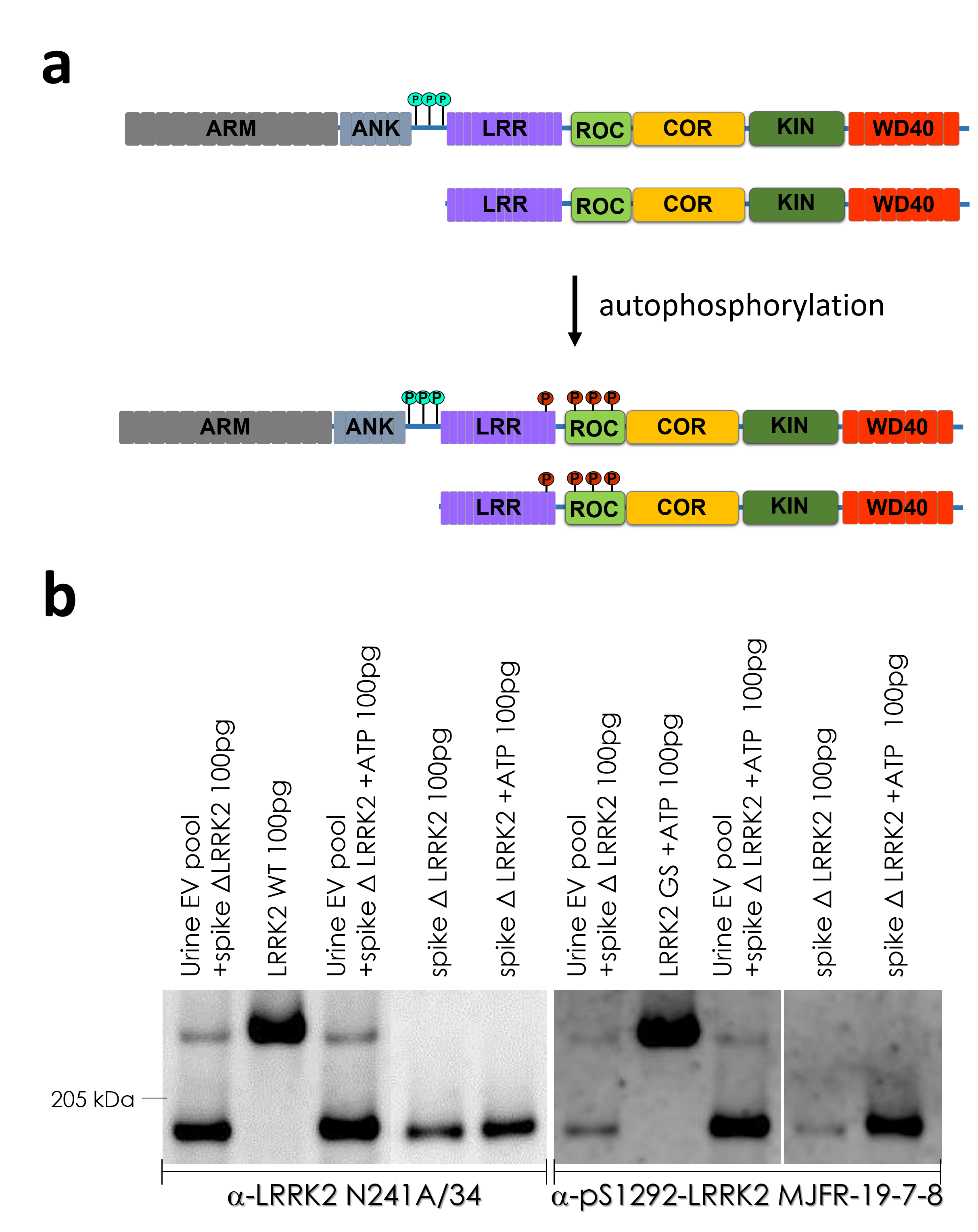


**Supplementary Figure 1**. Preparation of LRRK2 calibration controls.

Three different calibration controls for LRRK2 were prepared, for use as detection and quantification controls on western blots. The ‘pool’ calibrator consists of a pool of all of the EV isolates in the study. It therefore provides a signal approximately equivalent to the average of all samples. It also contains the other EV proteins besides LRRK2. The ‘Rec’ calibrator corresponds to recombinant full length LRRK2. For total-LRRK2 measures, this is the WT version, for the pS1292-LRRK2 epitope, this is the LRRK2 G2019S recombinant protein submitted to autophosphorylation. The ‘spike’ calibrator is recombinant, truncated LRRK2 (residues 970-2527) submitted to autophosphorylation and spiked into the pool of EVs. The autophosphorylation step is described in materials and methods and is schematically represented in (A). Panel (B) shows the the detection of these different calibrators, both for the total LRRK2 detection with N241A/34 anti-LRRK2 antibody and for detection of the autophosphorylation epitope pS1292-LRRK2.


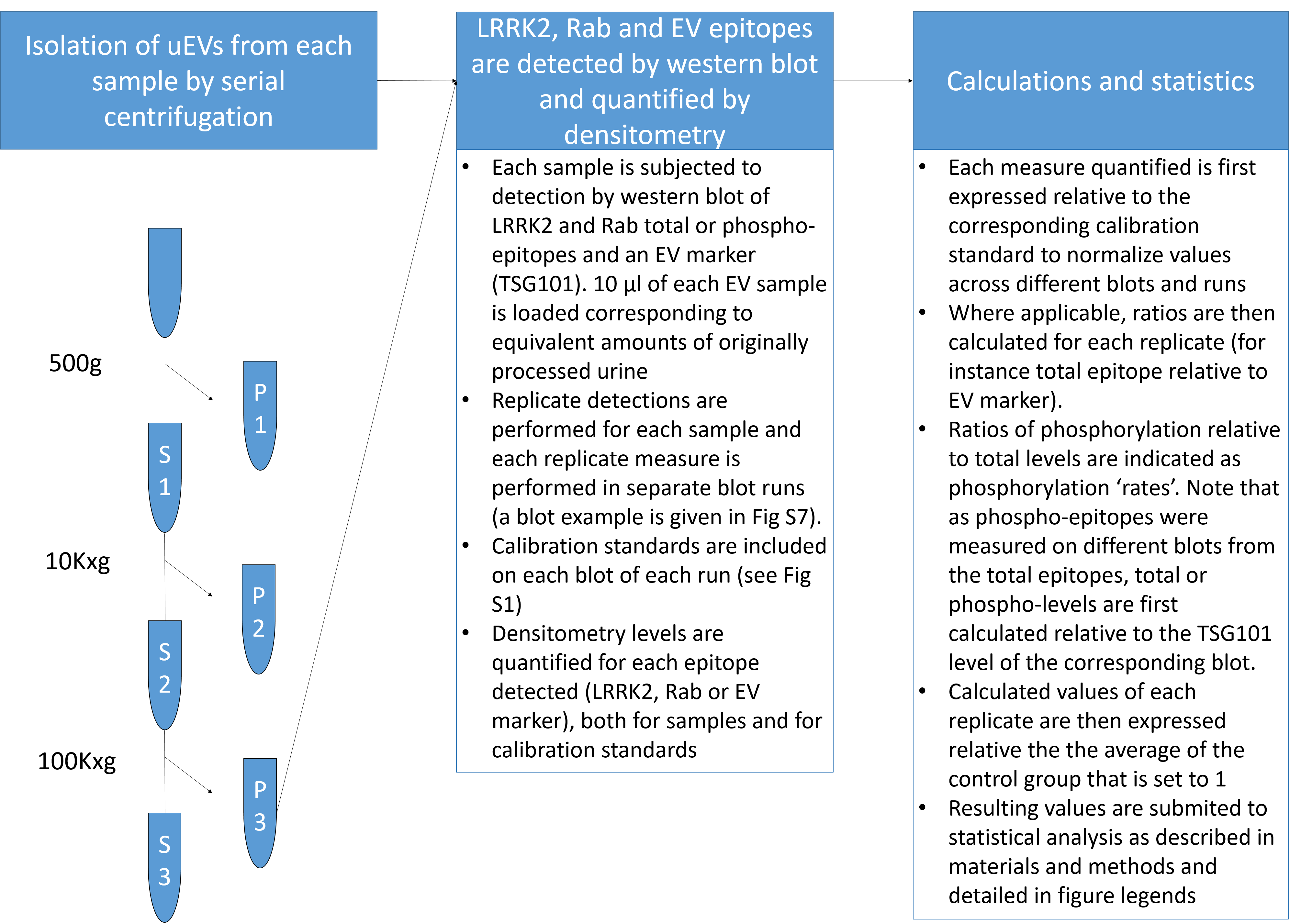


**Supplementary Figure 2**. Flow chart of steps from sample processing to determination of LRRK2, Rab and EV values. The flow chart indicates 3 major steps: i) sample processing, ii) detection of the different epitopes by western blotting and their quantification, and iii) the calculations to reach the presented values. Full details of each step can be found in materials and methods.


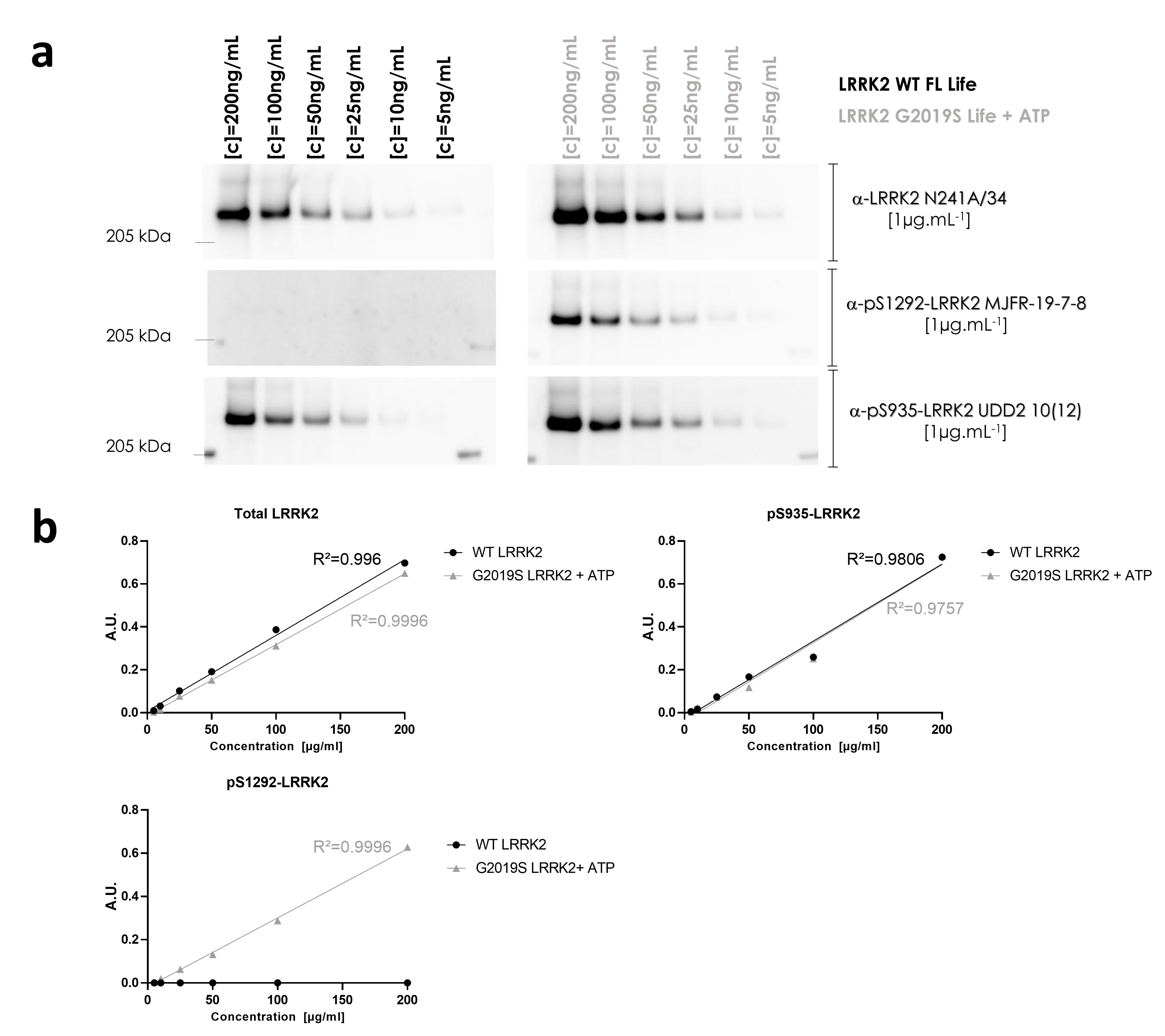


**Supplementary Figure 3**. Validation of the signal linearity of western blot detection of LRRK2, for total-LRRK2, pS935-LRRK2 and pS1292-LRRK2.

(A). Serial dilutions of recombinant LRRK2 WT and recombinant autophosphorylated LRRK2 G2019S LRRK2, ranging from 5 ng/ml to 200 ng/ml, were analyzed by western blot for epitopes of total LRRK2, pS1292-LRRK2 and pS935-LRRK2.

(B) Blots in A were quantified as described in materials and methods and plots of western blot signals at different calibrator concentrations were submitted to linear regression. Data in blue is from the LRRK2 WT calibrator and in red from the autophosphorylated LRRK2 G2019S. Values for the square of the correlation coefficient (R²) all surpass 0,97 indicative of a very good linearity in the quantification of detection over this range of protein concentrations.


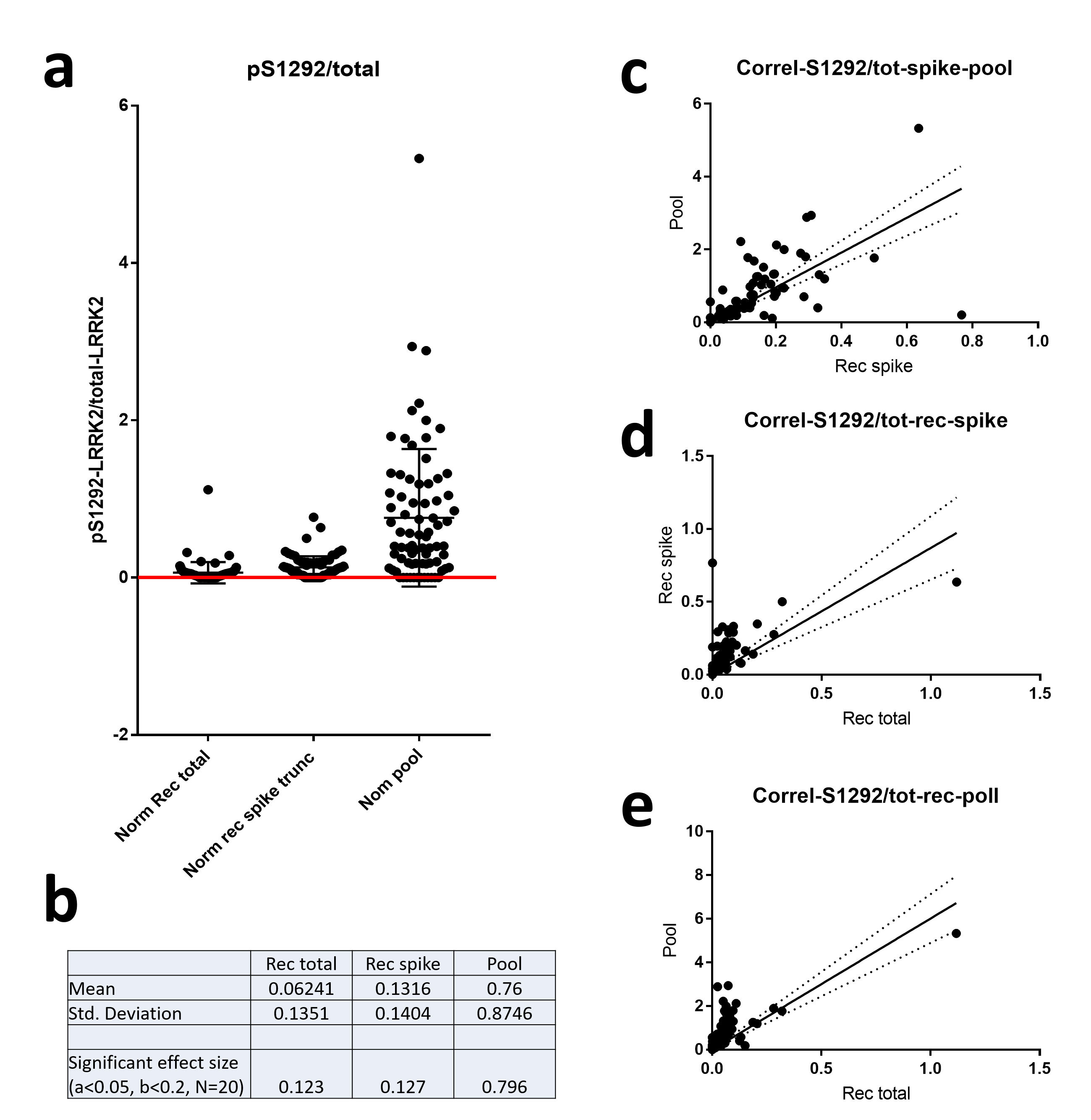


**Supplementary Figure 4**. Comparison of quantifications of LRRK2 epitopes obtained from three different calibrators. The measure of the ratio of pS1292-LRRK2 with total-LRRK2 was determined from the same samples using three different calibrators (as described in supplementary Figure 1). Panel (A) illustrates of the spread of experimental values obtained using different calibrators and (B) shows a table with means, standard deviations and significant effect sizes for values derived from each calibrator. Panels (C), (D) and (E) show correlation graphs for all 3 combinations of 2 calibrators, indicating a good correlation between the different calibrators. In the paper, the pool calibrator was used in priority as its signal corresponds to an average signal of the samples tested and this calibrator also contained other proteins such as the exosome marker TSG101.


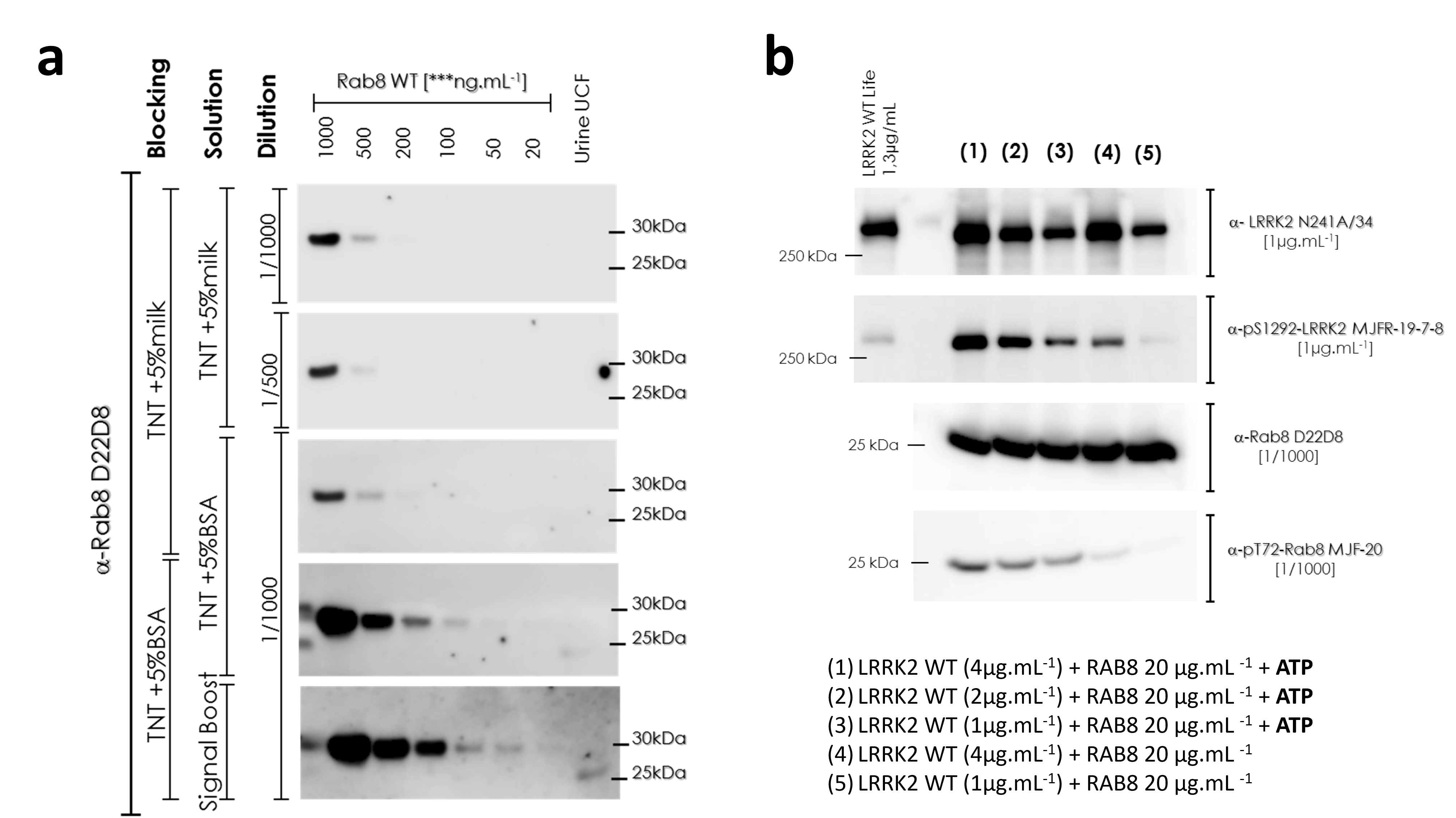


**Supplementary Figure 5**. Recombinant standards and optimization of detection and quantification for Rab8. (A) Western blot images obtained for a range of recombinant Rab8 WT dilutions (from 20 to 1000 ng/ml) with several different blocking solutions and several concentrations of the anti-Rab8 D22D8 antibody. (B) Western blot detection of LRRK2 (total and pS1292-LRRK2) and Rab8 (total and pT72-Rab8) for different conditions of concentrations of LRRK2 and incubation with and without ATP. The 5 conditions are listed below the blot.


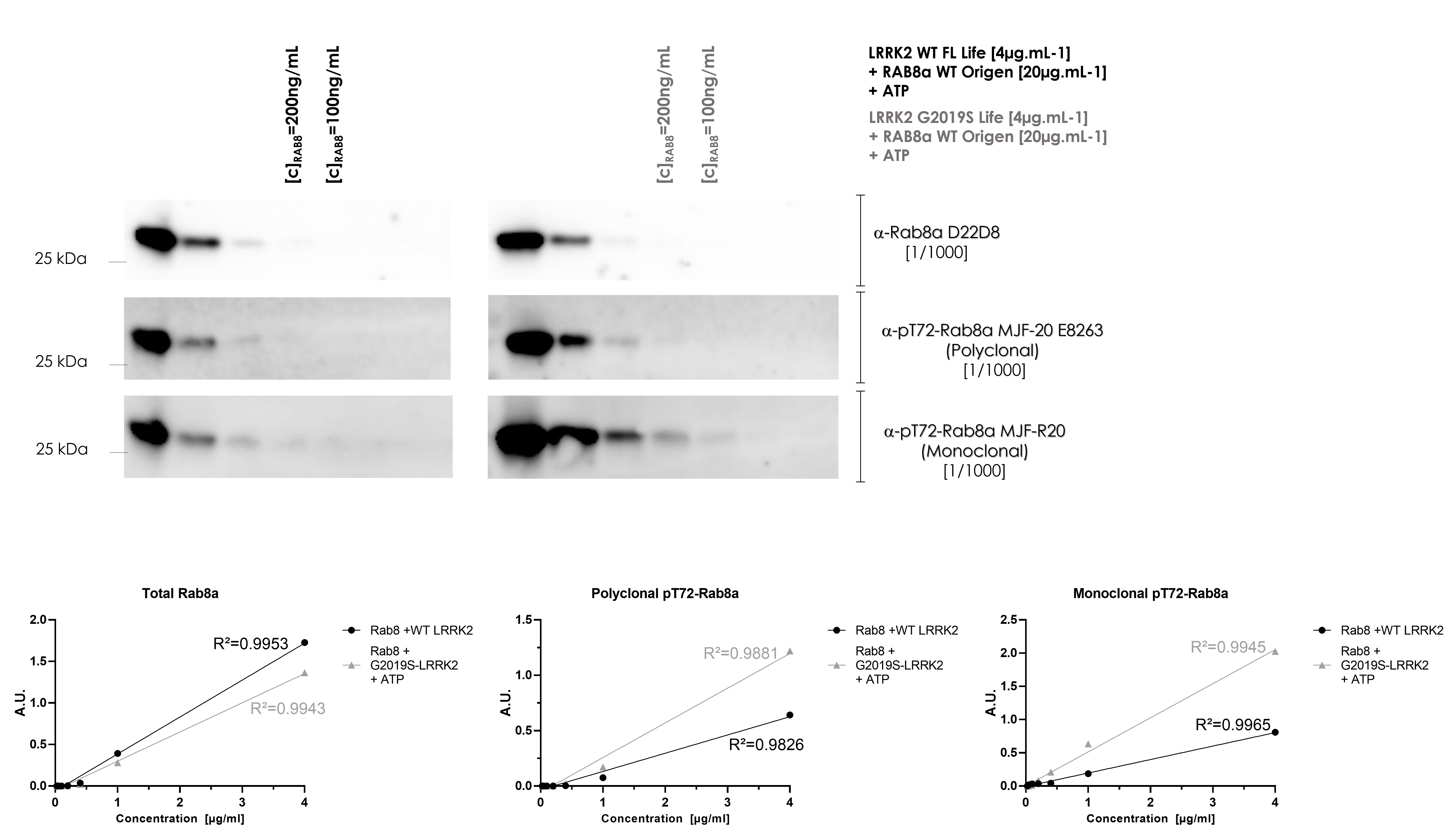


**Supplementary Figure 6**. Validation of the signal linearity of western blot detection for RAB8 calibrators. Recombinant RAB8 was incubated with recombinant LRRK2 (WT or G2019S) and ATP to phosphorylate RAB8 at Thr72, as depicted also in supplementary Figure 4. (A) The recombinant phosphorylated protein was serially diluted from 4 µg/ml to 25 ng/ml and analyzed by western blot for detection of total and phosphorylated RAB8. (B) Blots in A were quantified as described in materials and methods and plots of western blot signals at different calibrator concentrations were submitted to linear regression. Values for the square of the correlation coefficient (R²) all surpass 0,98 indicative of a good linearity in the quantification of detection over this range of protein concentrations.


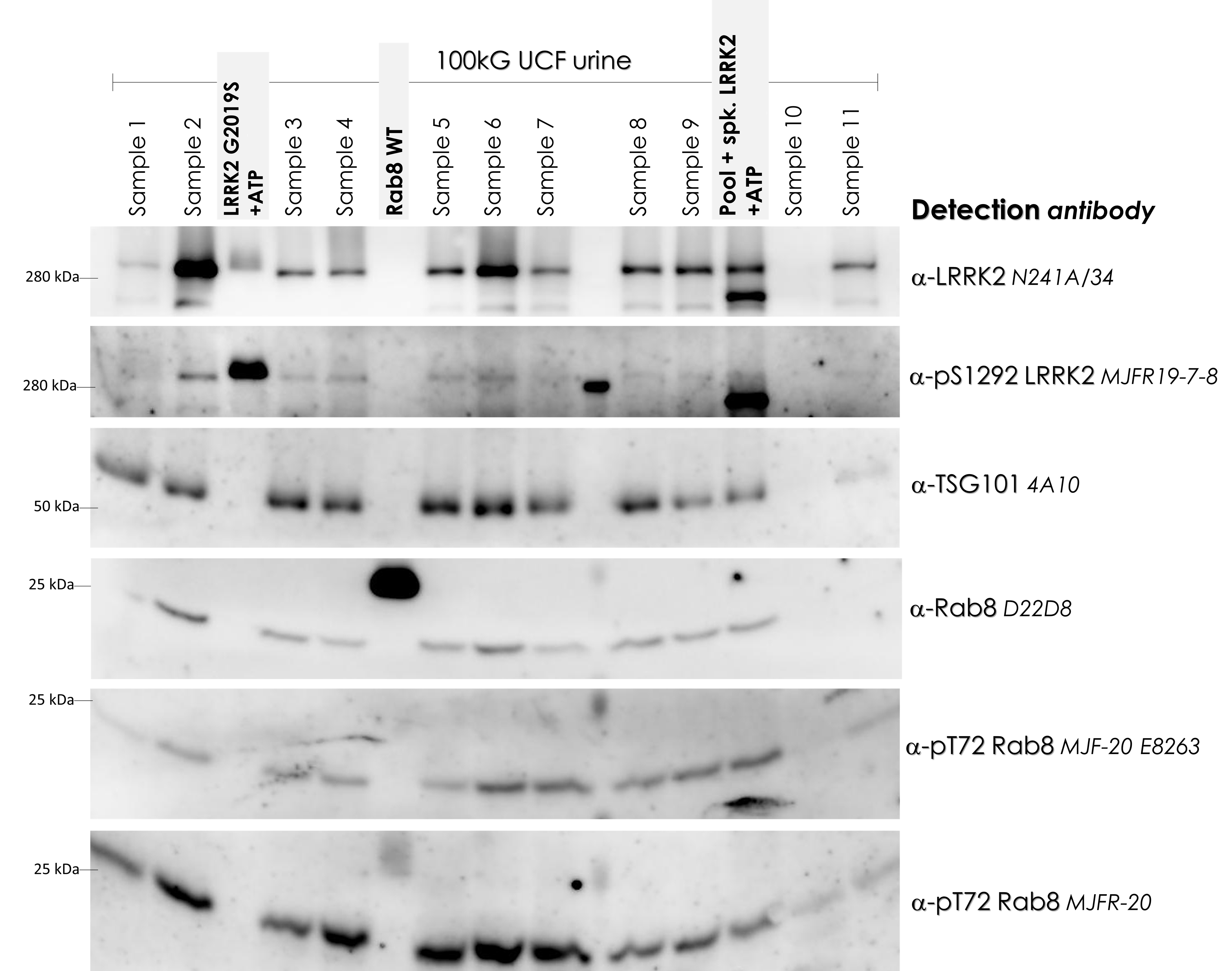


**Supplementary Figure 7**. Illustration of the combined detection of LRRK2, Rab8 and TSG101 epitopes in urinary exosomes. Representative western blot images obtained for detection in human urinary exosome samples of total-LRRK2, pS935-LRRK2 and pS1292-LRRK2 (in separate blots), total-Rab8 and pT72-Rab8 (in separate blots), along with TSG101 associated with each LRRK2 and Rab8 detection. These detections performed in the tested samples as well as calibration standards are subsequently quantified to obtain values for different clinical or experimental groups of the different LRRK2 and Rab measures as presented throughout this paper.


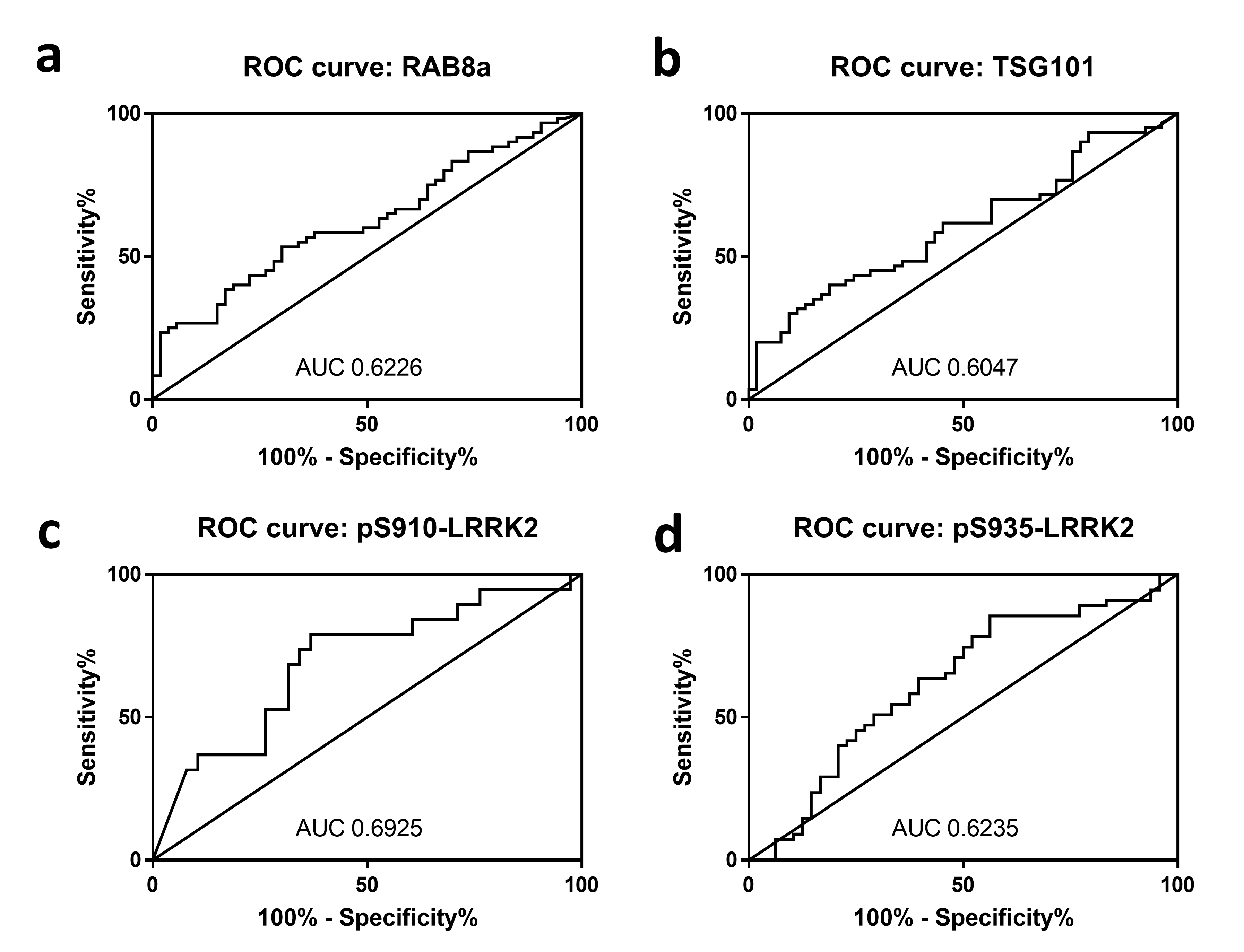


**Supplementary Figure 8**. Receiver operator characteristic (ROC) curve analysis for those measures that showed significant differences between samples from healthy controls and idiopathic PD patients in the study from the Lille University Hospital (Figure 3). (A) ROC curve for RAB8 with an area under the curve of 0,6226 and a P-value of 0,0248. (B) ROC curve for TSG101 with an area under the curve of 0,6047 and a P-value of 0,0554. (C) ROC curve for pS910-LRRK2 with an area under the curve of 0,6925 and a P-value of 0,0186. (D) ROC curve for pS935-LRRK2 with an area under the curve of 0,6235 and a P-value of 0,0311. AUC, area under the curve.


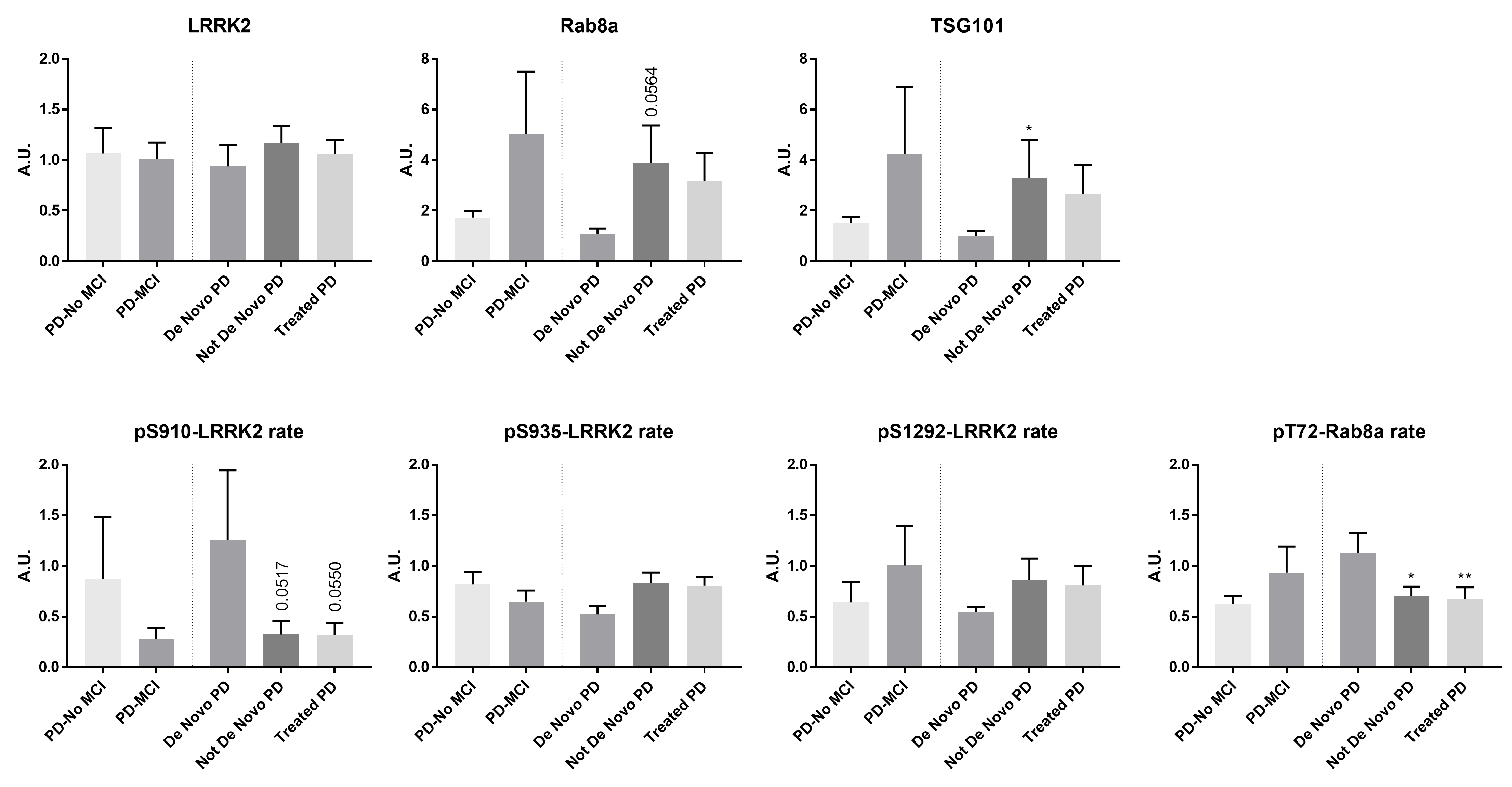


**Supplementary Figure 9**. Comparison of LRRK2 and Rab markers in urinary EVs of PD patients with or without mild cognitive impairment (MCI) or for *de novo* patients compared to non *de novo* or treated patients. These are values derived from the study presented in figure 3 with samples collected at the Lille University Hospital. Each graph shows first 2 bars of values from PD patients without MCI and values from PD patients with MCI. The following 3 bars correspond to values of PD patients that are *de novo*, not *de novo* and treated, respectively. Values represented are total LRRK2, total Rab8a, TSG101 in the top panels and pS910-LRRK2, pS935-LRRK2, pS1292-LRRK2 and pT72-Rab8 phosphorylation rates in the bottom panels.

Statistical differences were tested using using the two-tailed Mann-Whitney test for the 2 MCI groups and via the Kruskal-Wallis test followed by a Dunn’s multiple comparison test using the *de novo* PD group as control for the 3 *de novo* groups. Error bars represent standard error of the mean (s.e.m.). *P<0,05; **P<0,01.


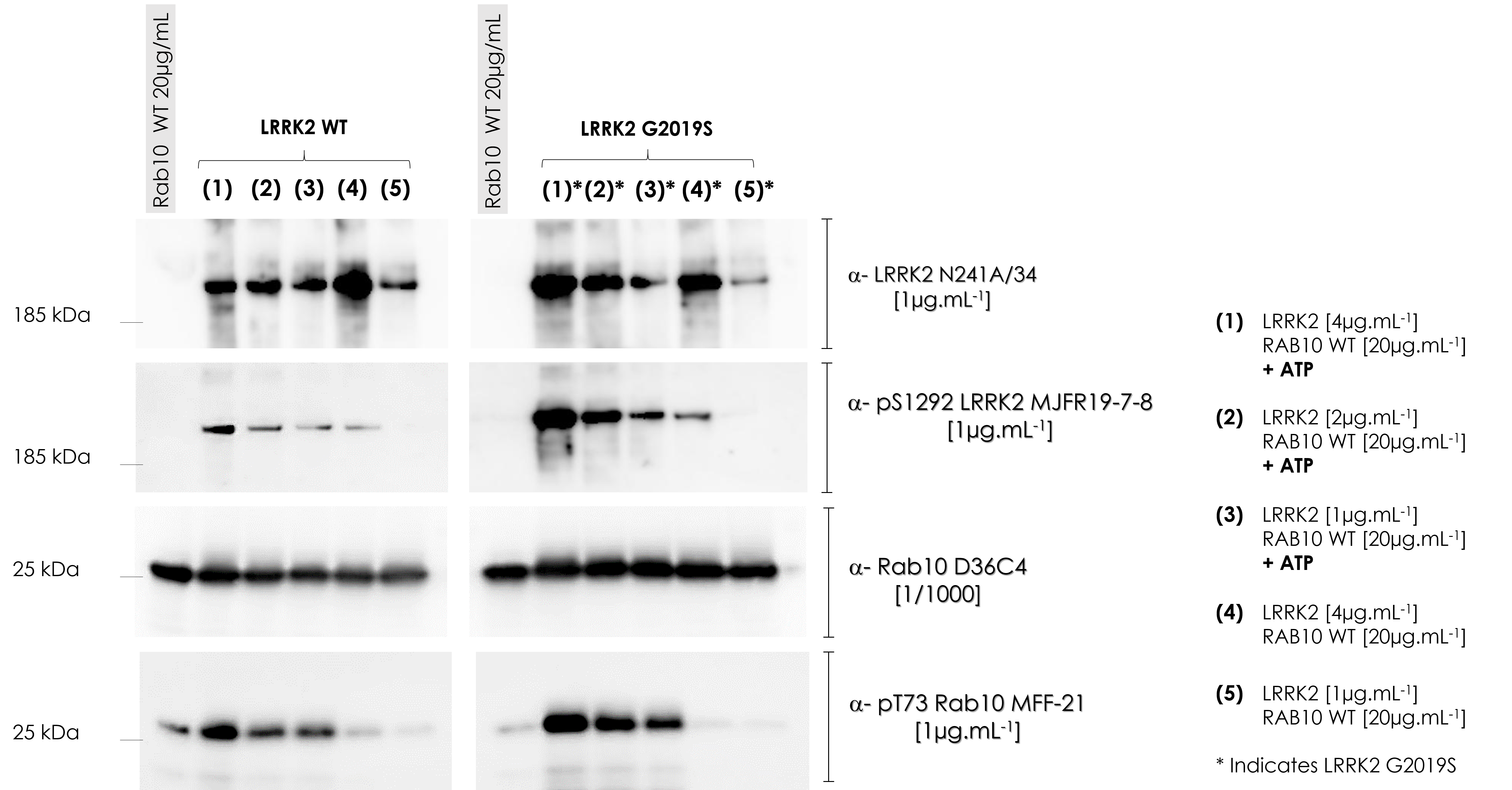


**Supplementary Figure 10**. Recombinant standards for Rab10. Different conditions of recombinant Rab10 protein incubated with recombinant LRRK2. The left panel of blots shows conditions of recombinant Rab10 protein incubated with different amounts of recombinant LRRK2 WT protein and ATP. The left panel shows the same conditions, replacing the LRRK2 WT with the LRRK2 G2019S recombinant protein. Note that the phospho-epitopes for LRRK2 autophosphorylation (pS1292-LRRK2) and Rab10 phosphorylation (pT73-Rab10) are increased when proteins are incubated with ATP and the increases are higher with the LRRK2 G2019S protein.


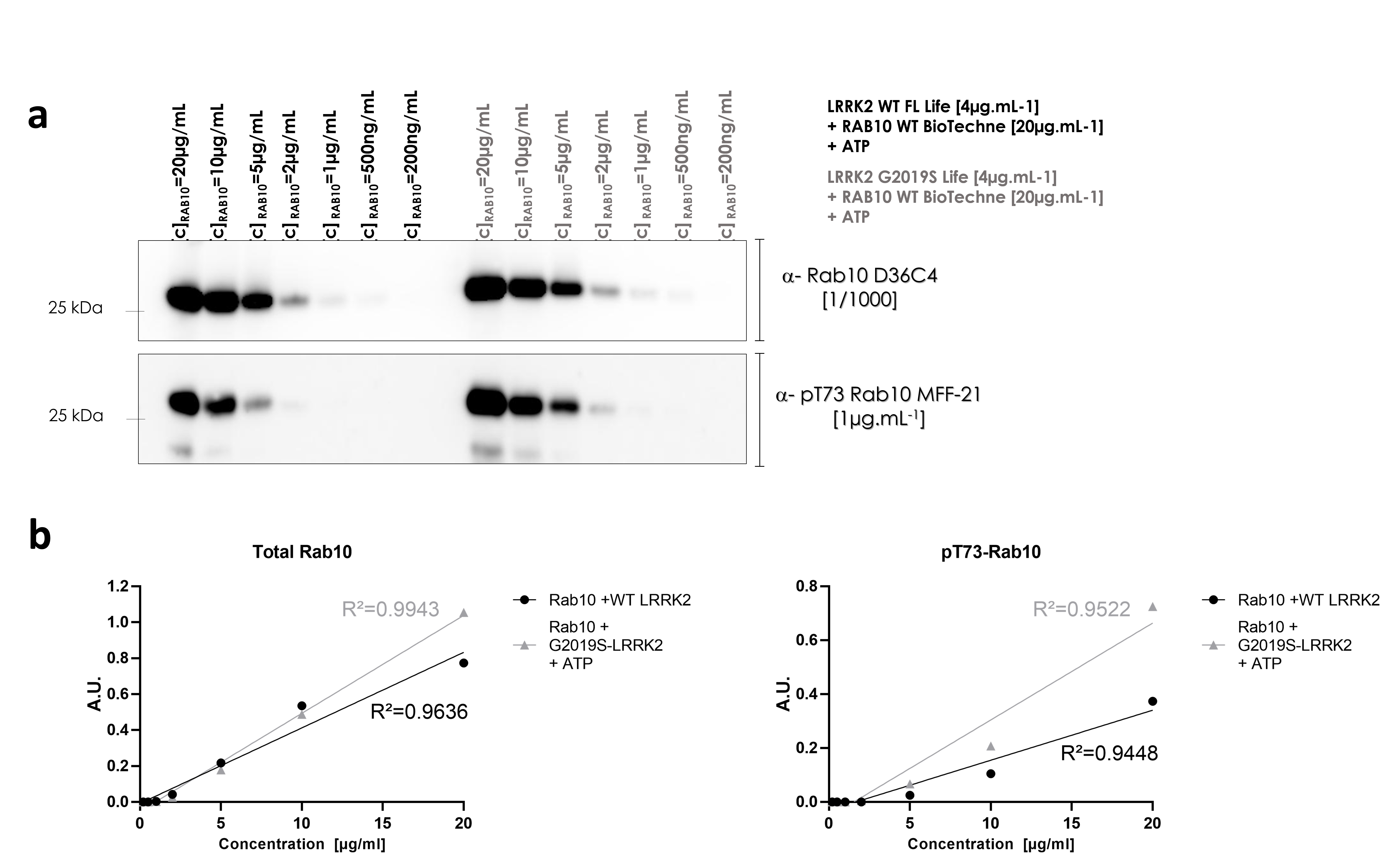


**Supplementary Figure 11**. Validation of the signal linearity of western blot detection for RAB10 calibrators. Recombinant RAB10 was incubated with recombinant LRRK2 (WT or G2019S) and ATP to phosphorylate RAB10 at Thr73, as depicted also in supplementary Figure 5. (A) The recombinant phosphorylated protein was serially diluted from 20 µg/ml to 200 ng/ml and analyzed by western blot for detection of total and phosphorylated RAB10. (B) Blots in A were quantified as described in materials and methods and plots of western blot signals at different calibrator concentrations were submitted to linear regression. Values for the square of the correlation coefficient (R²) all surpass 0,94 indicative of a good linearity in the quantification of detection over this range of protein concentrations.


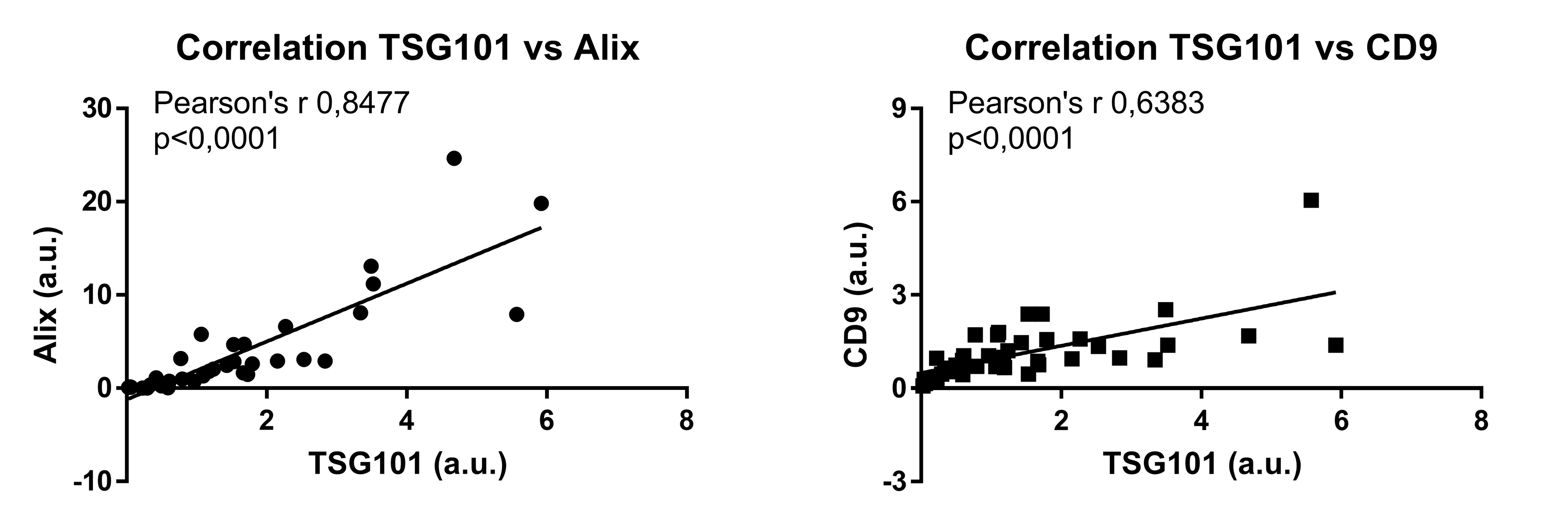


**Supplementary Figure 12**. Correlation of TSG101, Alix and CD9 in human uEVs. In order to compare different EV markers in our experimental conditions, we quantified the levels of three proteins commonly used as markers of extracellular vesicles, including TSG101, Alix and the tetraspanin CD9 for a subset of 40 samples (the same samples used for the analysis presented in Figure 4) and submitted the values to correlation analysis. The 2 graphs depicted show that the correlations of TSG101 with Alix and TSG101 with CD9 are both significant.


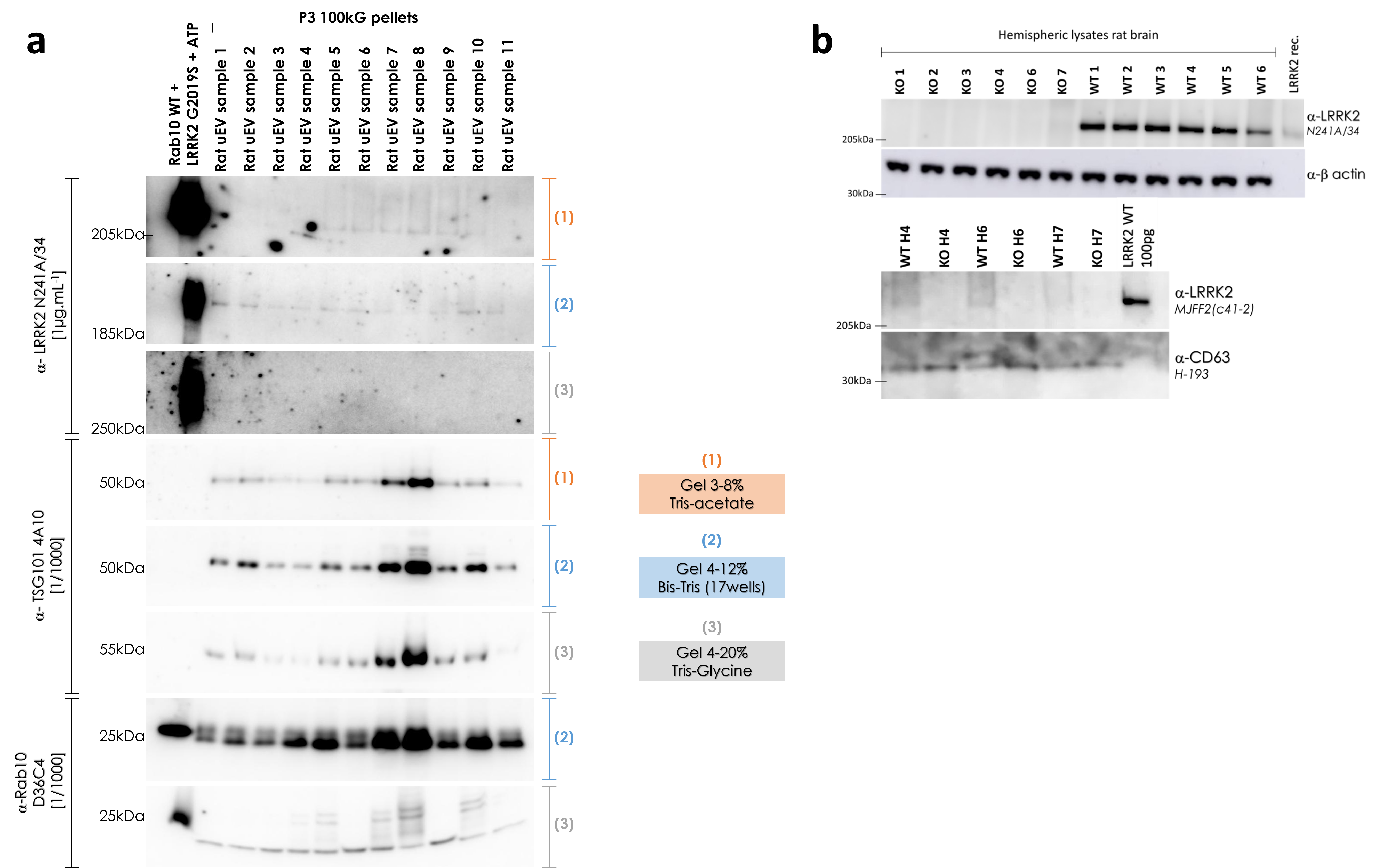


**Supplementary Figure 13**. Optimization of detection of LRRK2 and Rab10 in rat urinary EVs. A. 11 different samples of rat uEVs were submitted to western blot detection for total LRRK2, TSG101 and Rab10 using different gel types (3-8% Tris acetate, 4-12% Bis-Tris and 4-20% Tris-glycine. Included in the analysis in lane 1 are detection standards of LRRK2 G2019S and Rab10 recombinant proteins that had been incubated *in vitro* with ATP in order to phosphorylate the proteins. The blots illustrate that total LRRK2 detection is very low to absent and is insufficient for quantitation (note also that the pS935-LRRK2 epitope is also not detected, data not shown). Rab10 and TSG101 signal intensity was highest with the 4-12% Bis-Tris and these were used in the rat uEV analysis. B. Given the low signals of LRRK2 in rat uEVs, we tested for the presence of signal of LRRK2 in uEVs of normal rats compared to oLRRK2 KO rats. In the top panel, LRRK2 and beta-actin is detected in rat brains, confirming the loss of LRRK2 in LRRK2 KO tissues, and in the bottom panel, LRRK2 is detected in normal and LRRK2 KO uEVs. We observe a light signal for LRRK2 in the normal rat uEVs and this signal is not present in the LRRK2 KO uEVs, confirming that low levels of LRRK2 are present in rat uEVs. This level of detection is insufficient for quantification.


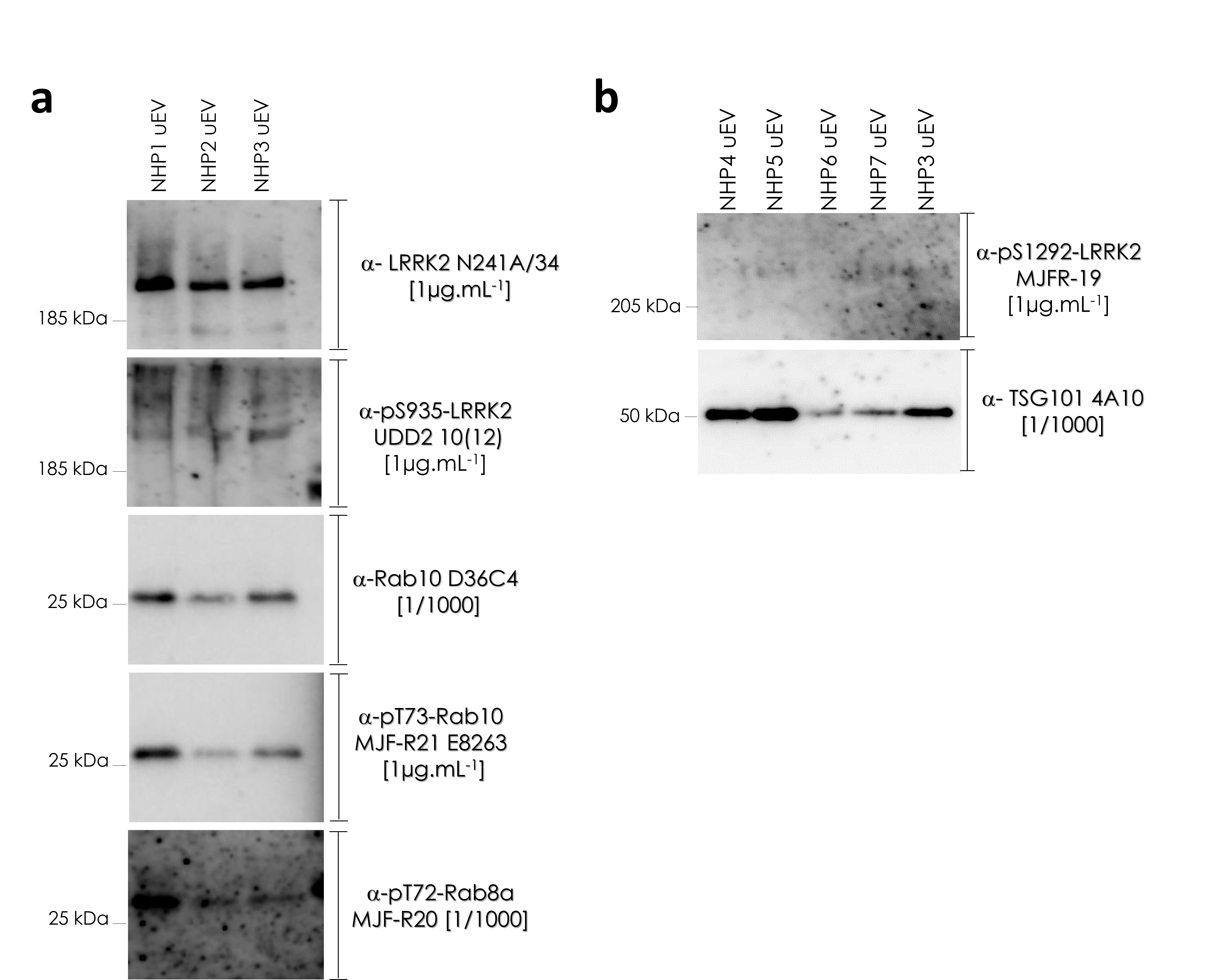


**Supplementary Figure 14**. Confirmation of LRRK2 and Rab10 detection in rhesus macaque urinary EVs. Different samples of NHP EVs were submitted to western blot detection for LRRK2 total and phospho-epitopes, Rab total and phospho-epitopes as well as TSG101. Detection was confirmed for all epitopes (A), with the exception of pS1292-LRRK2 (B) that was at or below the detection limit in these detection conditions.

Supplementary tables:

Supplementary Table 1:

|  |  | *Age @ diagnosis* | *Age @ onset* | *Age* | *Total Levodopa Equivalent dose* | *MoCA Total Score* | *Hoehn & Yahr Stage (modified)* | *Modified Schwab and England Consensus Rating - OVERALL* |
| --- | --- | --- | --- | --- | --- | --- | --- | --- |
| LRRK2 | *Pearson's r* | 0.143 | 0.1539 | 0.1266 | 0.08275 | **-0.2546** | 0.000668 | -0.1119 |
|  | *P (one-tailed)* | 0.2215 | 0.2043 | 0.1519 | 0.2799 | **0.0195** | 0.4979 | 0.31 |
|  | *P value summary* | ns | ns | ns | ns | ***** | ns | ns |
| TSG10 | *Pearson's r* | 0.07283 | 0.08082 | 0.108 | **0.2919** | -0.09289 | 0.1389 | **-0.6038** |
|  | *P (one-tailed)* | 0.3485 | 0.3328 | 0.1904 | **0.0179** | 0.2291 | 0.1348 | **0.0015** |
|  | *P value summary* | ns | ns | ns | ***** | ns | ns | ****** |
| LRRK2/TSG101 | *Pearson's r* | 0.185 | 0.2401 | -0.02276 | -0.1603 | -0.08775 | -0.1547 | 0.1418 |
|  | *P (one-tailed)* | 0.1514 | 0.0892 | 0.4258 | 0.1234 | 0.2384 | 0.1057 | 0.2645 |
|  | *P value summary* | ns | ns | ns | ns | ns | ns | ns |
| Ser(P)-1292-LRRK2 | *Pearson's r* | 0.2631 | **0.3483** | **0.21** | -0.02507 | -0.1561 | -0.007204 | -0.3018 |
|  | *P (one-tailed)* | 0.076382 | **0.027414** | **0.042844** | 0.429971 | 0.105313 | 0.477289 | 0.086119 |
|  | *P value summary* | ns | ***** | ***** | ns | ns | ns | ns |
| Ser(P)-1292-LRRK2/LRRK2 | *Pearson's r* | 0.1358 | 0.2043 | 0.1167 | 0.05283 | -0.1319 | **0.2425** | **-0.5172** |
|  | *P (one-tailed)* | 0.2372 | 0.1394 | 0.1734 | 0.3564 | 0.1474 | **0.0268** | **0.0069** |
|  | *P value summary* | ns | ns | ns | ns | ns | ***** | ****** |
| Ser(P)-1292-LRRK2/TSG101 | *Pearson's r* | 0.07582 | 0.1144 | -0.06359 | -0.139 | -0.02035 | -0.157 | 0.0937 |
|  | *P (one-tailed)* | 0.3426 | 0.27 | 0.3032 | 0.1629 | 0.4356 | 0.1058 | 0.3392 |
|  | *P value summary* | ns | ns | ns | ns | ns | ns | ns |

**Supplementary Table 1**: Correlation analysis performed across all groups for selected clinical parameters and selected LRRK2 measures. MoCA, Montreal Cognitive Assessment.

Supplementary Table 2:

|  |  |  | Statistical summary^§^ |  |  | Statistical summary^§^ |
| --- | --- | --- | --- | --- | --- | --- |
| Descriptor | HC-F | PD-F | Control vs iPD (F) | HC-M | PD-M | Control vs iPD (M) |
| Gender, M/F n | 31 | 24 |  | 22 | 36 |  |
| Weight (kg), mean ± SD (median) | 68 ± 13.3 (66) | 66 ± 12.9 (67) | n.s. | 83 ± 10.6 (80) | 87 ± 15.4 (82) | n.s. |
| Height (cm), mean ± SD (median) | 164 ± 7.1 (164) | 163 ± 5.9 (163) | n.s. | 177 ± 8 (178) | 176 ± 7.4 (175) | n.s. |
| Body Mass Index, mean ± SD (median) | 25.1 ± 4.2 (24.3) | 25.1 ± 4.8 (24.6) | n.s. | 26.5 ± 3.7 (25.6) | 28 ± 4.2 (26.8) | 0.2542 |
| Age at sampling (years), mean ± SD (median) | 64.1 ± 9.3 (63) | 63.1 ± 11.4 (65.5) | n.s. | 64.6 ± 10.1 (63) | 60.7 ± 11.1 (60.5) | 0.2869 |
| Age at onset (years), mean ± SD (median) | NA | 55.9 ± 14.1 (56) | NA | NA | 54.9 ± 11.1 (57) | NA |
| Age at diagnosis (years), mean ± SD (median) | NA | 57.3 ± 13.8 (57) | NA | NA | 55.9 ± 11 (57) | NA |
| Disease duration (years), mean ± SD (median) | NA | 5.9 ± 7 (3.5) | NA | NA | 4.9 ± 5.1 (4.5) | NA |
| *De novo*, total (%) | NA | 7 (29%) | NA | NA | 6 (17%) | NA |
| Familial History of PD, n= total no/total yes | NA | 11/13 | NA | NA | 29/7 | NA |
| LEDD mg, mean ± SD (median) | NA | 539.9 ± 542.4 (400) | NA | NA | 670.1 ± 589.6 (487) | NA |
| UPDRS III, mean ± SD (median) | NA | 18.4 ± 19.1 (12) | NA | NA | 22.8 ± 11.7 (20) | NA |
| Modified Hoehn and Yahr score, mean ± SD (median) | NA | 2.25 ± 0.83 (2) | NA | NA | 2.42 ± 0.7 (2.5) | NA |
| Schwab and England, mean ± SD (median) | NA | 80 ± 16.4 (90) | NA | NA | 80.6 ± 14.3 (90) | NA |
| MoCA, mean ± SD (median), out of 30 | NA | 25.3 ± 3.9 (26) | NA | NA | 25.1 ± 2.7 (25) | NA |
| MMSE, mean ± SD (median) | 28.3 ± 1.4 (29) | 27.6 ± 2.1 (28) | n.s. | 28.5 ± 1.4 (28.5) | 27.3 ± 1.9 (28) | ***0.0363*** |
| Cognitive Impairment, n | NA | MCI n=9, dementia n=2, | NA | NA | MCI n=12, dementia n=3, | NA |
|  |  | no cognitive impairment n=10, |  |  | no cognitive impairment n=14, |  |
|  |  | unknown n=3 |  |  | unknown n=7 |  |

**Supplementary Table 2**: Overview of clinical parameters from clinical cohort 2 (cohort from Lille University Hospital), broken down for men and women.

LEDD, L-dopa equivalent daily dosage; MMSE, Mini-Mental State Examination; MoCA, The Montreal Cognitive Assessment; PD, Parkinson disease; UPDRS, Unified Parkinson’s Disease Rating Scale; NA, not applicable. HC-F, HC-M, Health control female or male, PD-F, PD-M, PD patient female or male.

§P values > 0.3 are indicated as n.s. (not significant). P values < 0.05 are bold italics. Statistical comparisons are carried out for controls compared to idiopathic PD and for idiopathic PD compared to LRRK2 PD by use of the 2-tailed Mann–Whitney test. Statistical comparisons indicated with £ are determined via a Chi² test.
