# Supplementary material for: Alterations in the LRRK2-Rab pathway in urinary extracellular vesicles as Parkinson’s disease and pharmacodynamic biomarkers
Source: NPJ Parkinsons Dis. 2023 Feb 7;9:21. doi: 10.1038/s41531-023-00445-9 (PMC9905493; doi:10.1038/s41531-023-00445-9)

Supplementary Information file with full, uncropped western blots (Figure 1d, 5a, 5b, 5c, 6a, and supplementary figures: 1b, 3a, 5a, 5b, 6, 7, 10, 11a, 13a, 13b, 14a, 14b)

Title: Alterations in the LRRK2-Rab pathway in urinary extracellular vesicles as Parkinson's disease and pharmacodynamic biomarkers

Authors: Jean-Marc Taymans, Eugenie Mutez, William Sibran, Laurine Vandewynckel, Claire Deldycke, Séverine Bleuse, Antoine Marchand, Alessia Sarchione, Alexandre Kreisler, Clémence Simonin, James Koprich, Guillaume Baille, Luc Defebvre, Kathy Dujardin, Alain Destée, and Marie-Christine Chartier-Harlin

Figure 1d

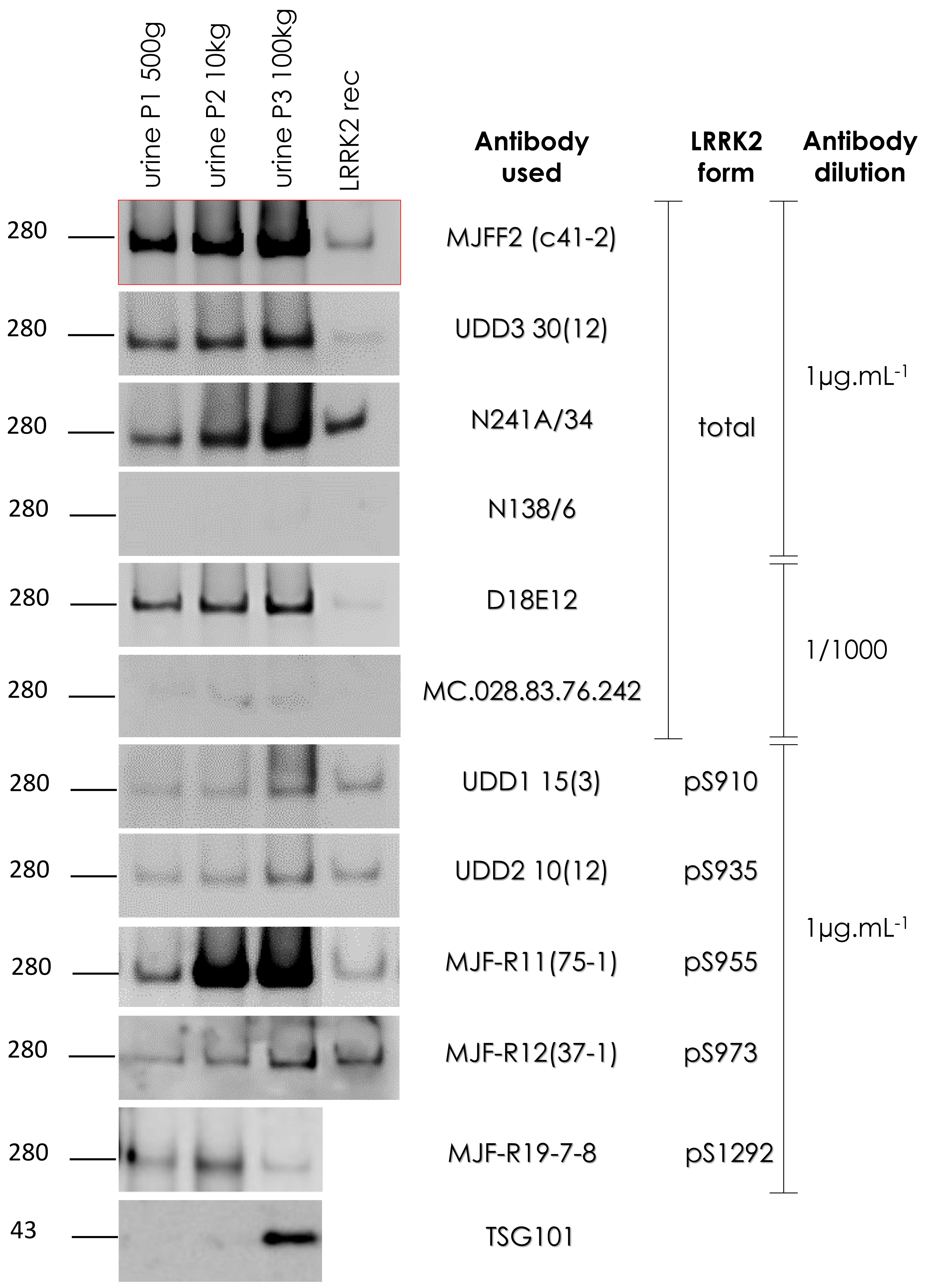

urine P1 500g
urine P2 10kg
urine P3 100kg
LRRK2 rec
Antibody used
LRRK2 form
Antibody dilution
280
MJFF2 (c41-2)
280
UDD3 30(12)
280
N241A/34
280
N138/6
280
D18E12
280
MC.028.83.76.242
total
1µg.mL-1
1/1000
280
UDD1 15(3)
pS910
280
UDD2 10(12)
pS935
280
MJF-R11(75-1)
pS955
280
MJF-R12(37-1)
pS973
280
MJF-R19-7-8
pS1292
1µg.mL-1
43
TSG101

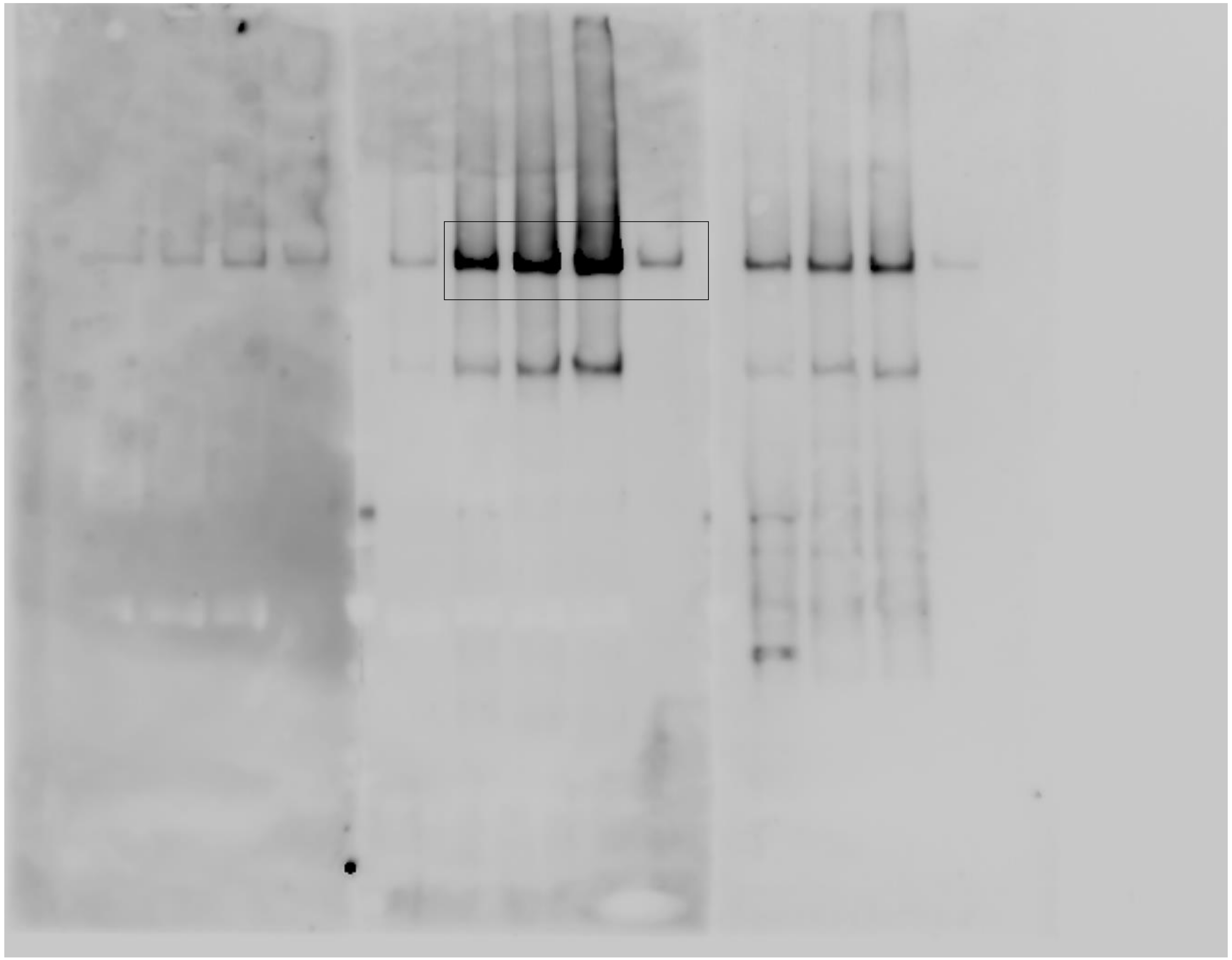

Figure 1d

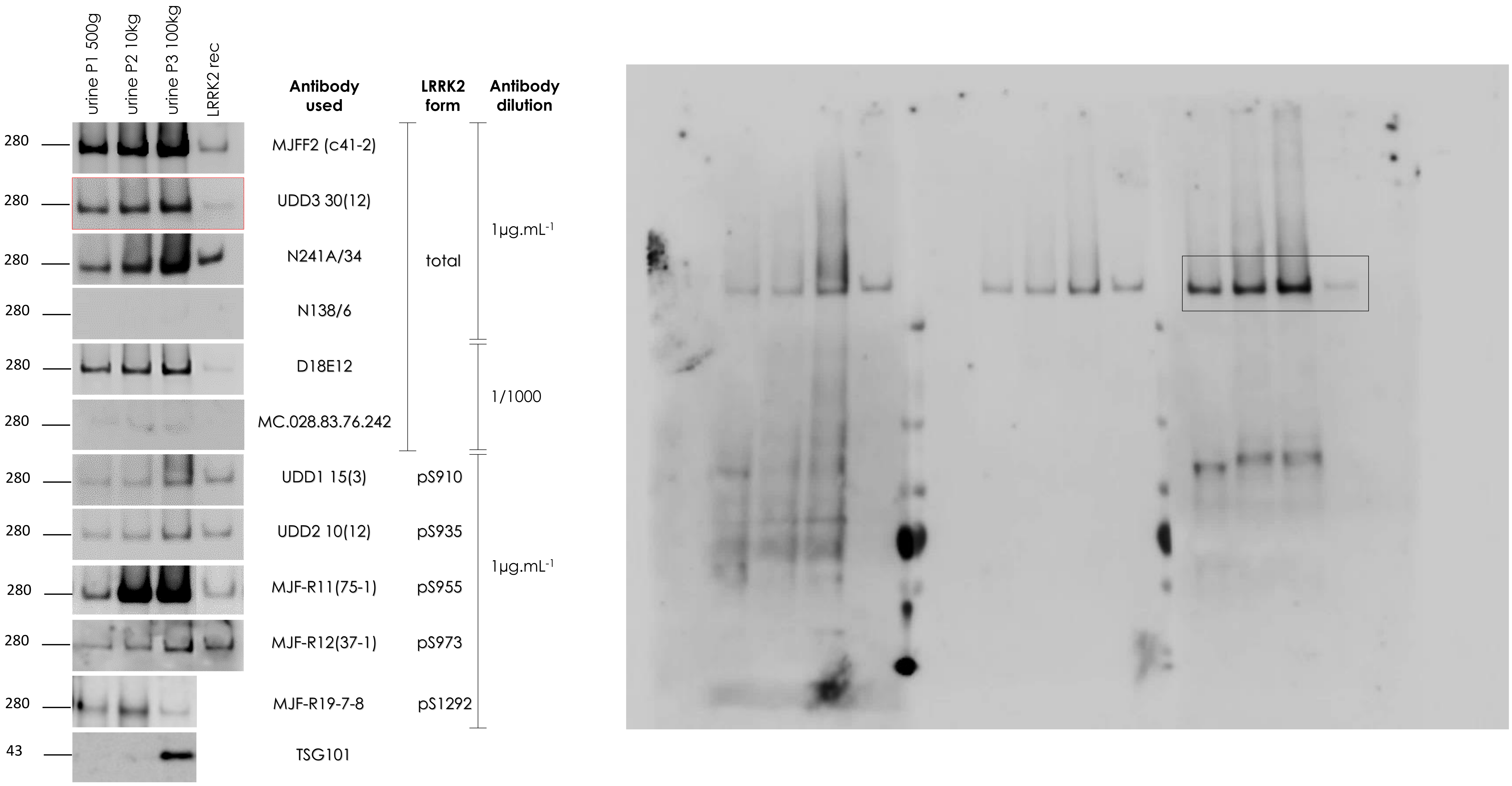
urine P1 500g
urine P2 10kg
urine P3 100kg
LRRK2 rec
Antibody used
LRRK2 form
Antibody dilution
280
MJFF2 (c41-2)
280
UDD3 30(12)
280
N241A/34
280
N138/6
280
D18E12
280
MC.028.83.76.242
280
UDD1 15(3)
pS910
280
UDD2 10(12)
pS935
280
MJF-R11(75-1)
pS955
280
MJF-R12(37-1)
pS973
280
MJF-R19-7-8
pS1292
43
TSG101
total
1µg.mL-1
1/1000
1µg.mL-1

Figure 1d

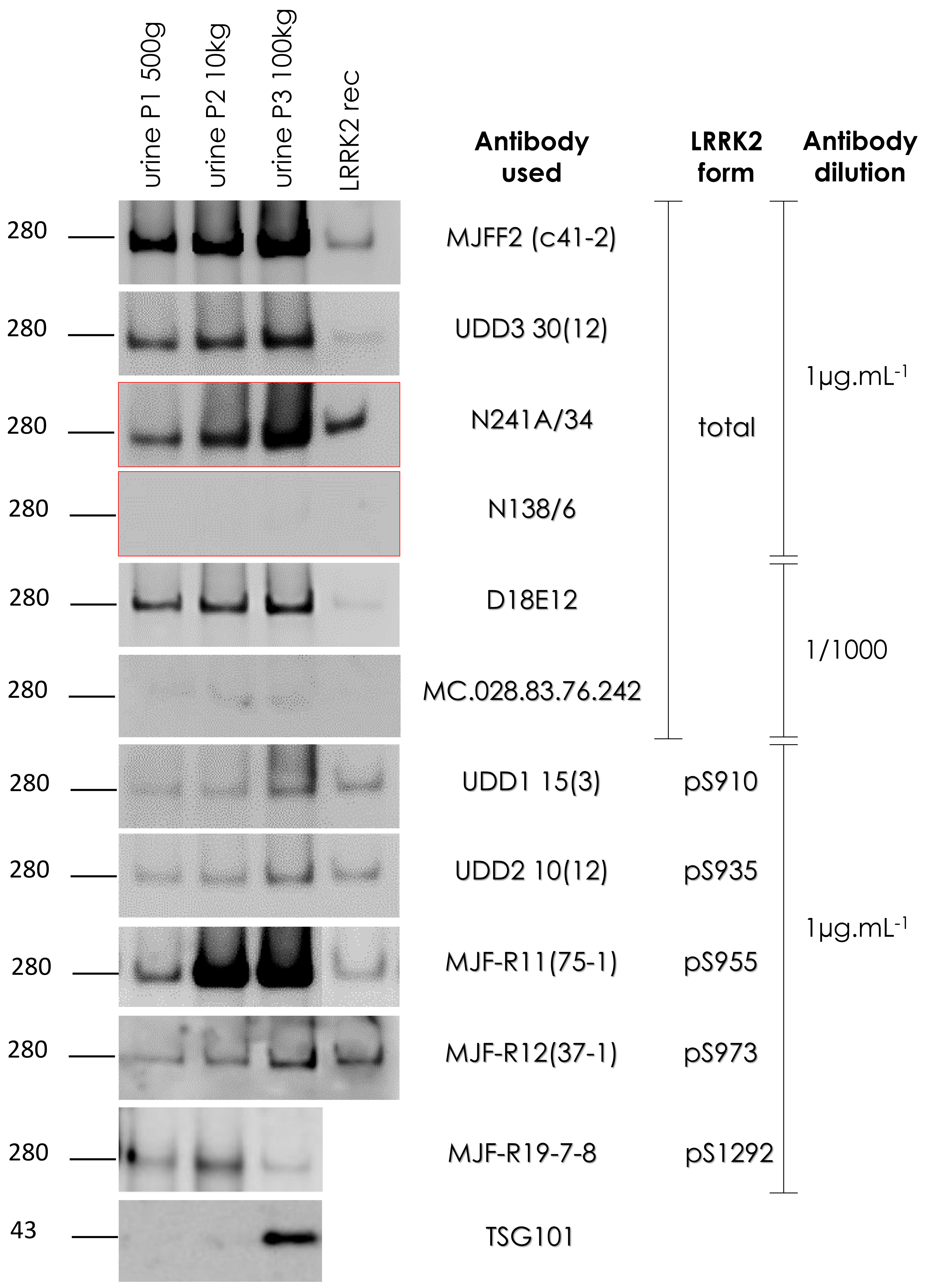
urine P1 500g
urine P2 10kg
urine P3 100kg
LRRK2 rec
Antibody used
LRRK2 form
Antibody dilution
280
MJFF2 (c41-2)
UDD3 30(12)
N241A/34
N138/6
D18E12
MC.028.83.76.242
UDD1 15(3)
UDD2 10(12)
MJF-R11(75-1)
MJF-R12(37-1)
MJF-R19-7-8
43
TSG101
total
pS910
pS935
pS955
pS973
pS1292
1µg.mL-1
1/1000
1µg.mL-1

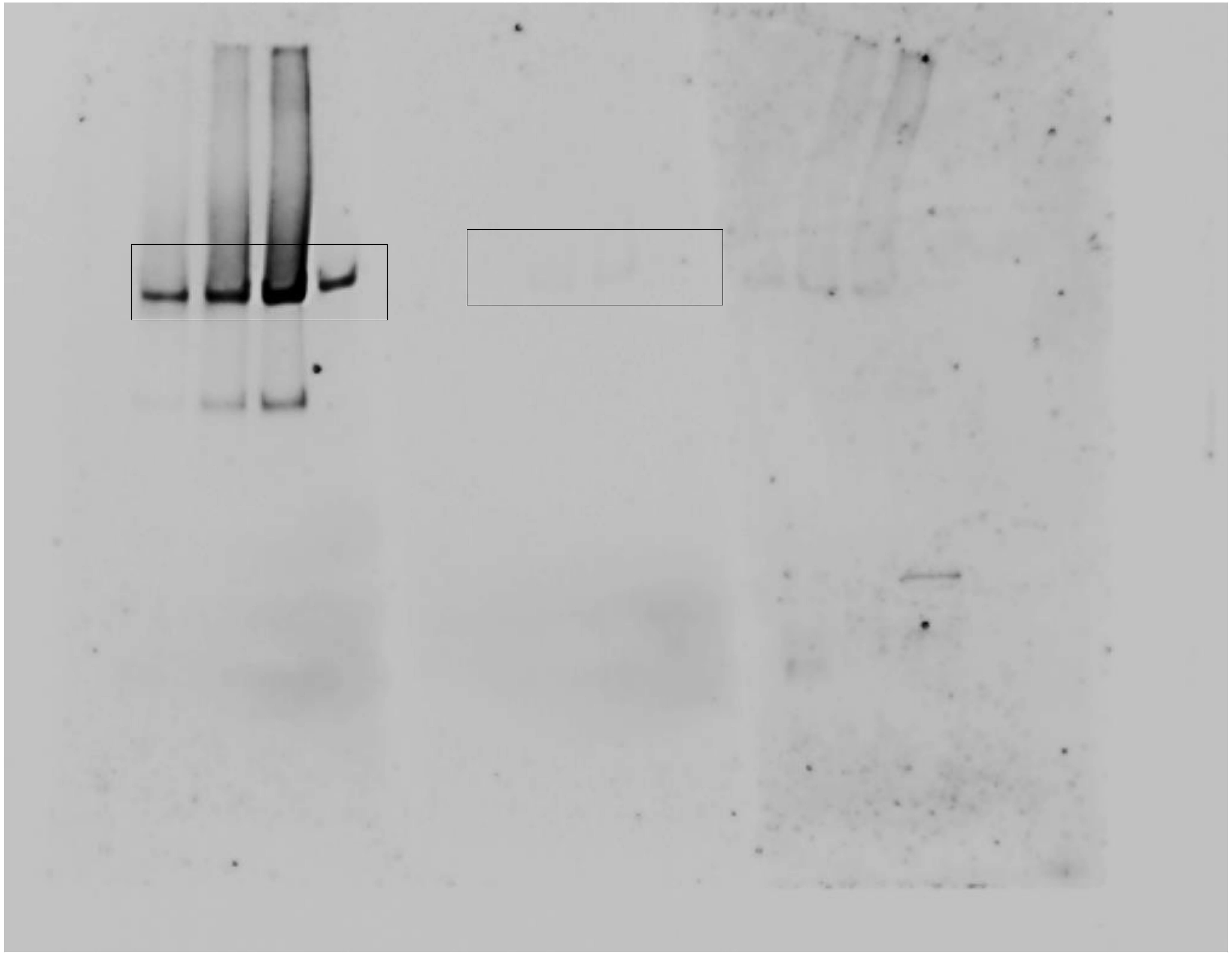

Figure 1d

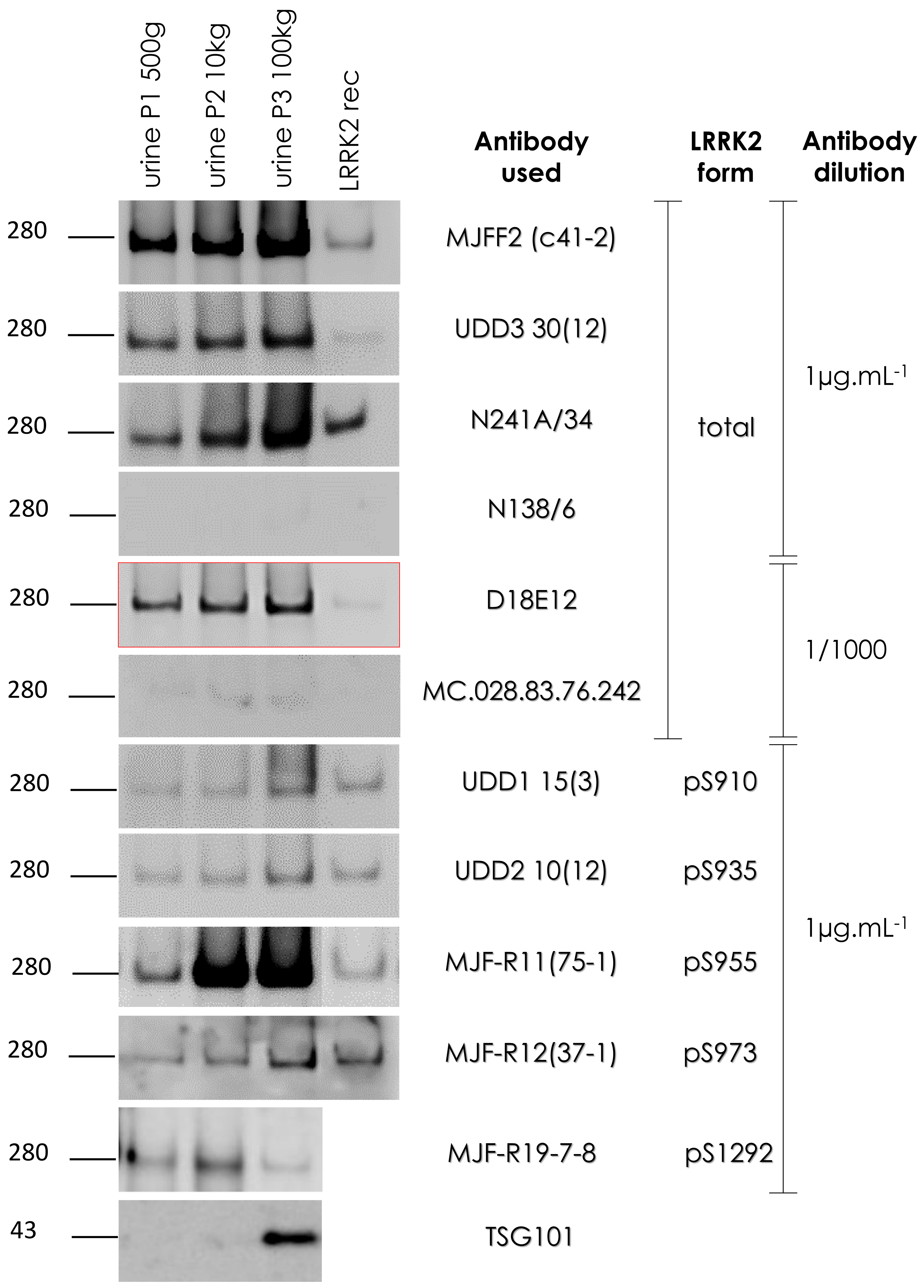

urine P1 500g
urine P2 10kg
urine P3 100kg
LRRK2 rec
Antibody used
LRRK2 form
Antibody dilution
280
MJFF2 (c41-2)
280
UDD3 30(12)
280
N241A/34
280
N138/6
280
D18E12
280
MC.028.83.76.242
total
1μg.mL-1
1/1000
280
UDD1 15(3)
pS910
280
UDD2 10(12)
pS935
280
MJF-R11(75-1)
pS955
280
MJF-R12(37-1)
pS973
280
MJF-R19-7-8
pS1292
1μg.mL-1
43
TSG101

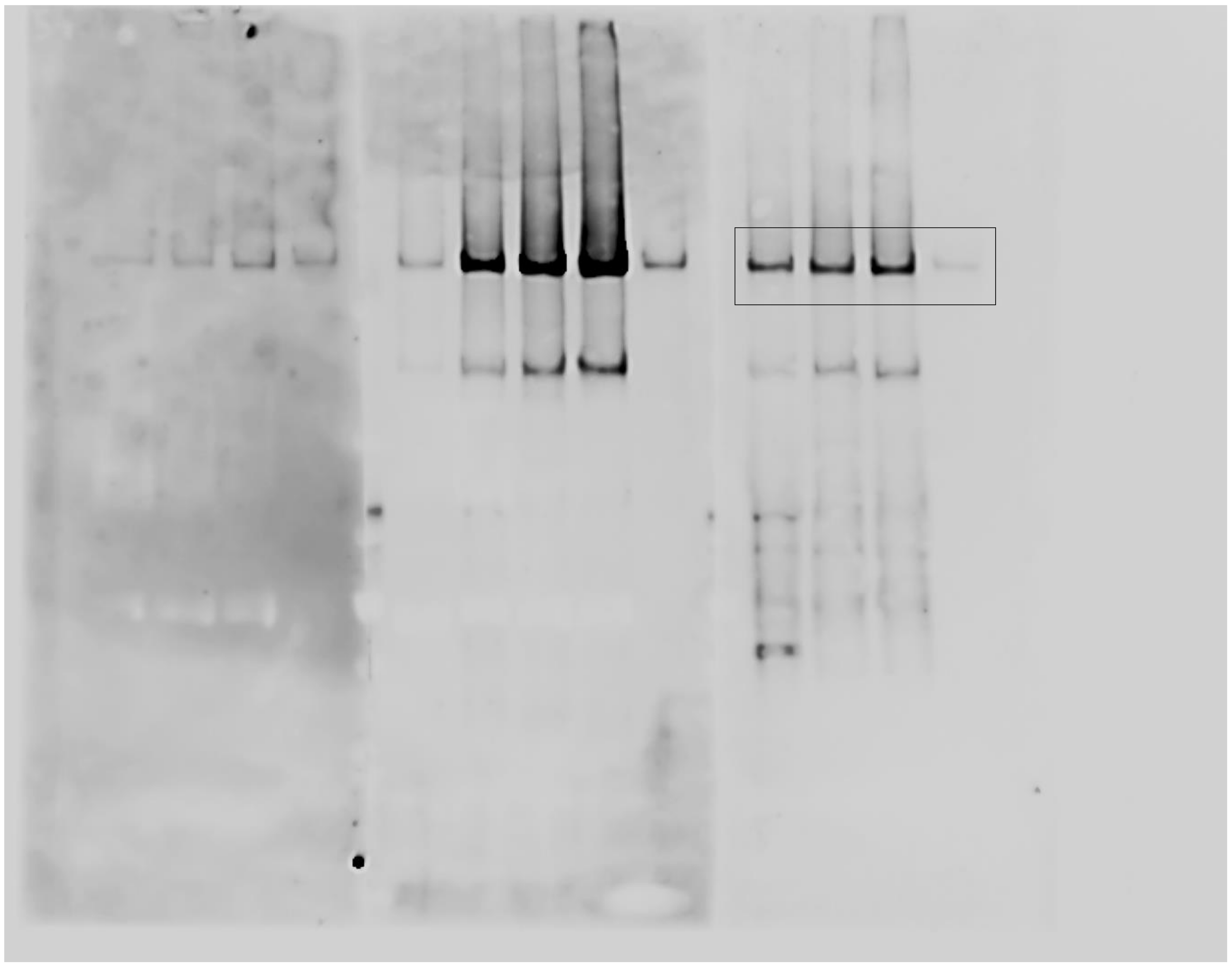

Figure 1d

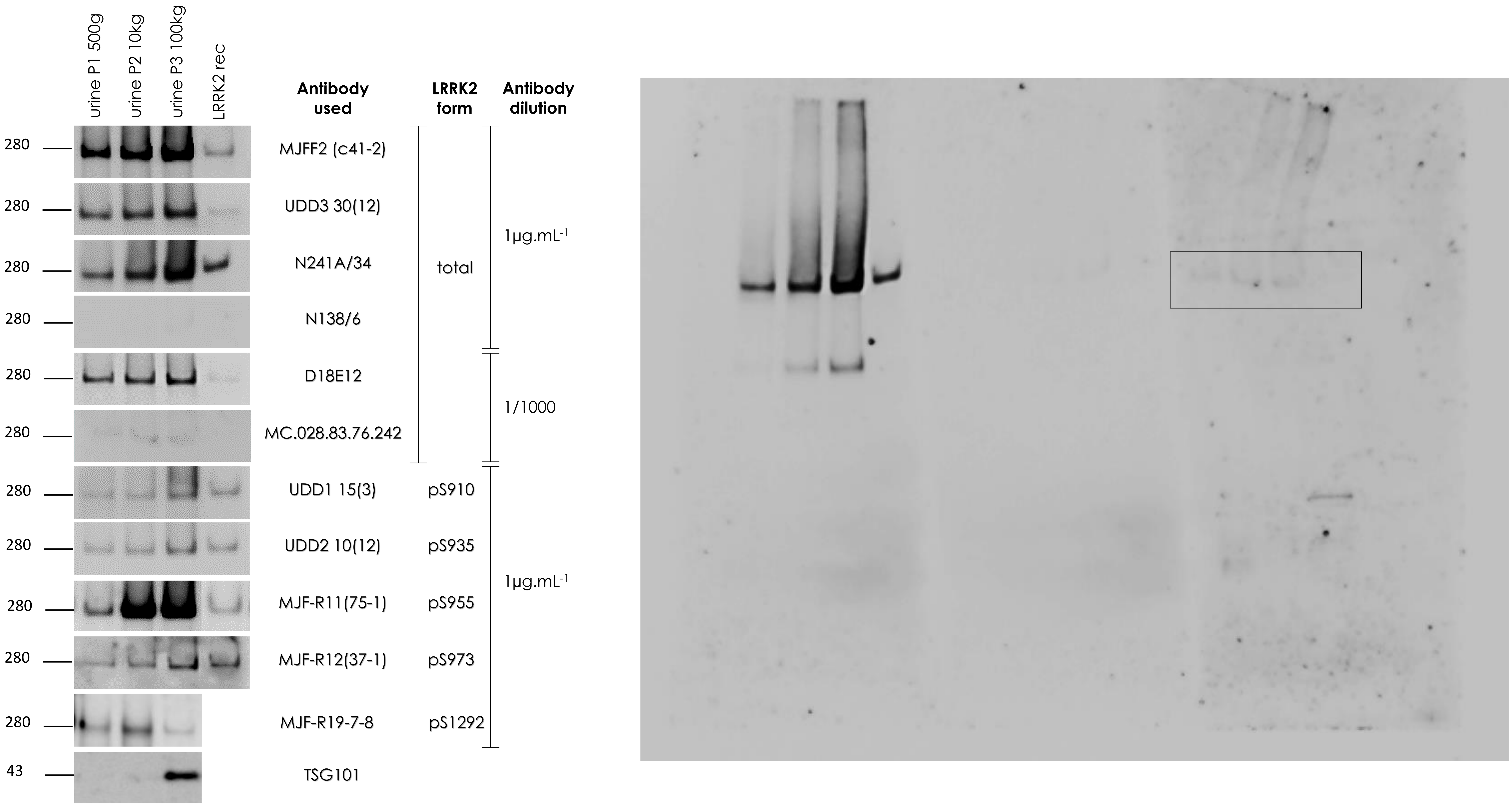

urine P1 500g
urine P2 10kg
urine P3 100kg
LRRK2 rec
Antibody used
LRRK2 form
Antibody dilution
280
MJFF2 (c41-2)
UDD3 30(12)
N241A/34
N138/6
D18E12
MC.028.83.76.242
UDD1 15(3)
UDD2 10(12)
MJF-R11(75-1)
MJF-R12(37-1)
MJF-R19-7-8
43
TSG101
total
pS910
pS935
pS955
pS973
pS1292
1µg.mL-1
1/1000
1µg.mL-1

Figure 1d

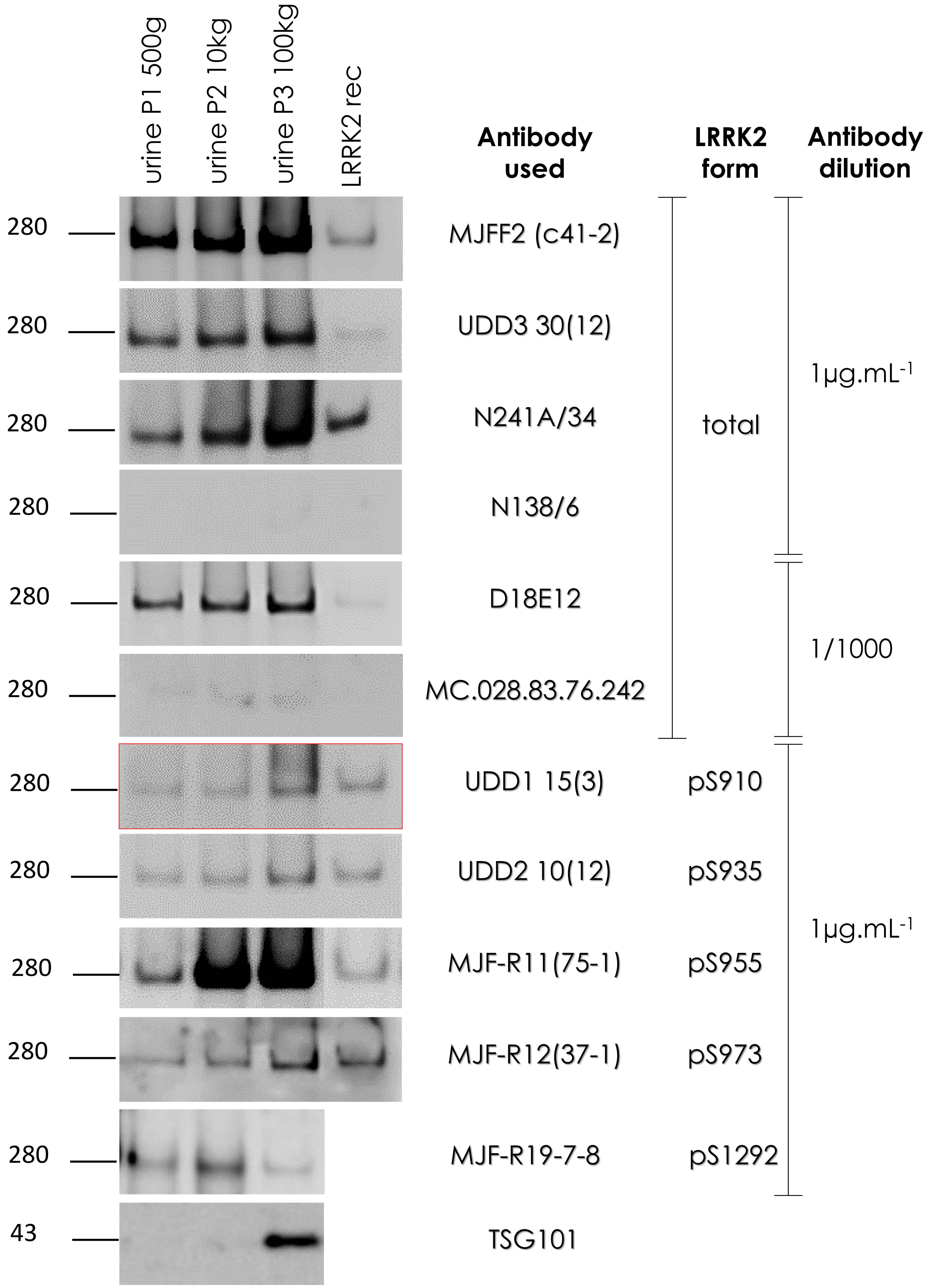

urine P1 500g
urine P2 10kg
urine P3 100kg
LRRK2 rec
Antibody used
LRRK2 form
Antibody dilution
280
MJFF2 (c41-2)
280
UDD3 30(12)
280
N241A/34
280
N138/6
280
D18E12
280
MC.028.83.76.242
280
UDD1 15(3)
pS910
280
UDD2 10(12)
pS935
280
MJF-R11(75-1)
pS955
280
MJF-R12(37-1)
pS973
280
MJF-R19-7-8
pS1292
43
TSG101
total
1µg.mL-1
1/1000
1µg.mL-1

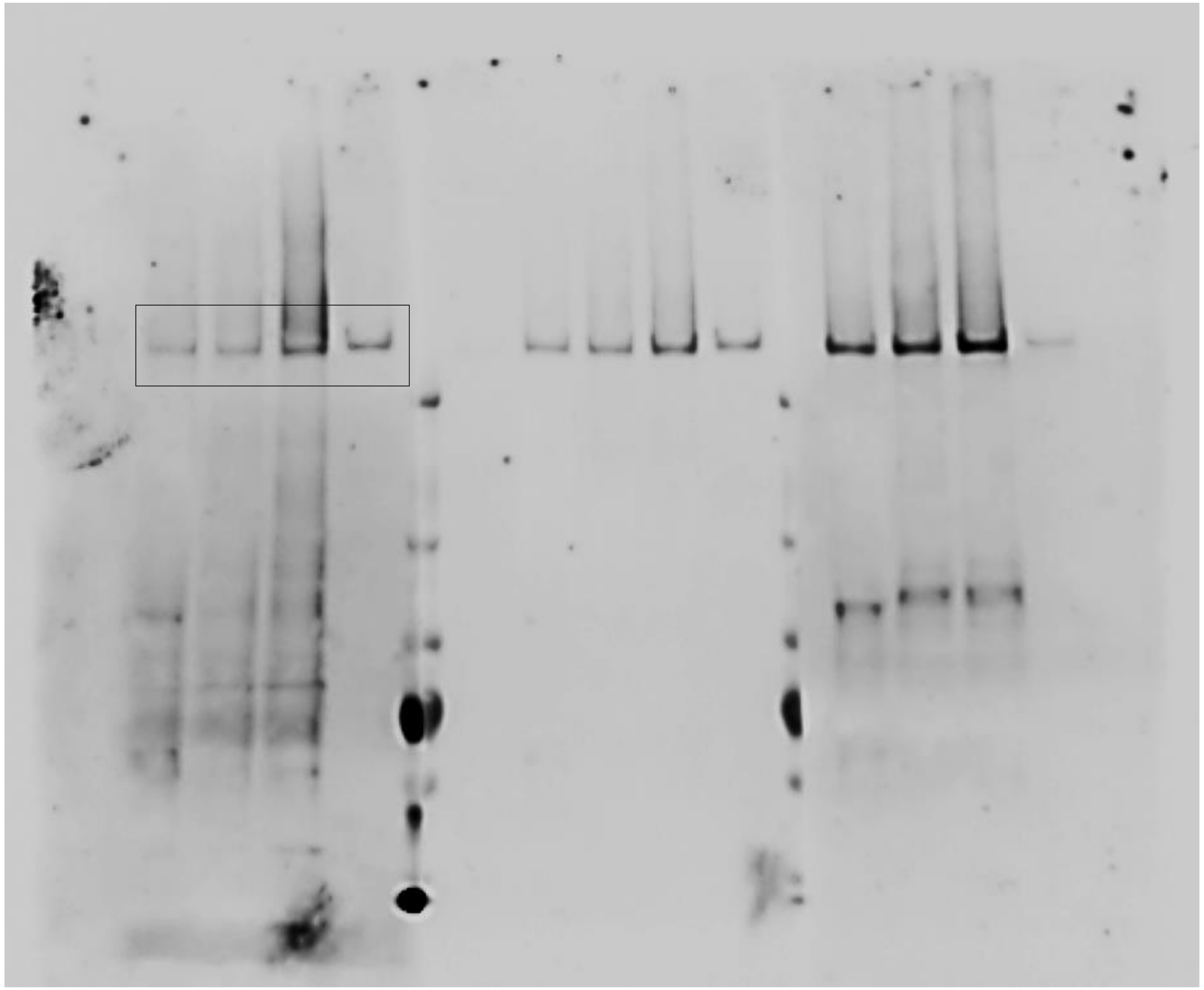

Figure 1d

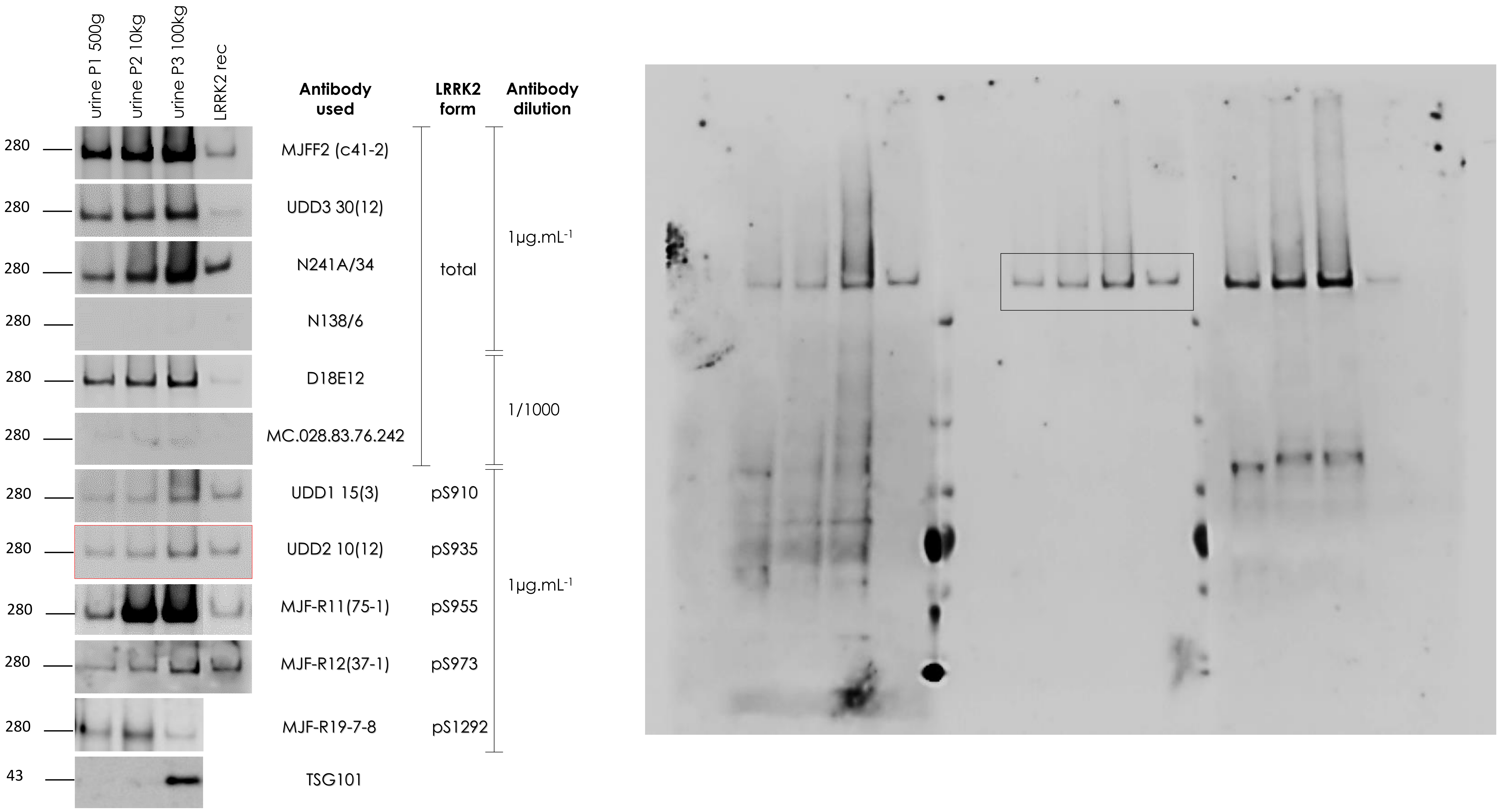

urine P1 500g
urine P2 10kg
urine P3 100kg
LRRK2 rec
Antibody used
LRRK2 form
Antibody dilution
280
MJFF2 (c41-2)
UDD3 30(12)
N241A/34
N138/6
D18E12
MC.028.83.76.242
total
1µg.mL-1
1/1000
UDD1 15(3)
pS910
UDD2 10(12)
pS935
MJF-R11(75-1)
pS955
MJF-R12(37-1)
pS973
MJF-R19-7-8
pS1292
1µg.mL-1
43
TSG101

Figure 1d

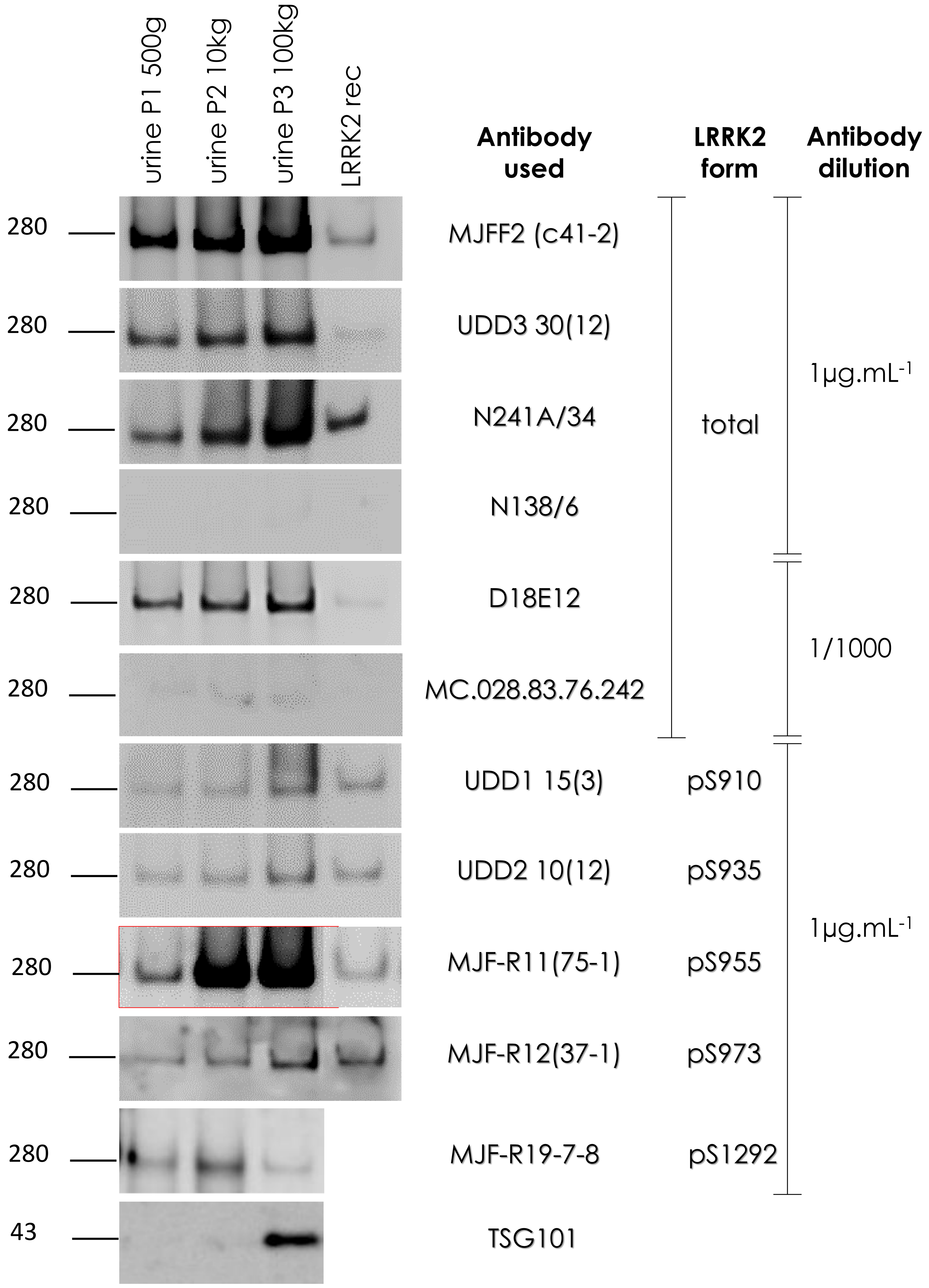

urine P1 500g
urine P2 10kg
urine P3 100kg
LRRK2 rec
Antibody used
LRRK2 form
Antibody dilution
280
MJFF2 (c41-2)
UDD3 30(12)
N241A/34
N138/6
D18E12
MC.028.83.76.242
UDD1 15(3)
UDD2 10(12)
MJF-R11(75-1)
MJF-R12(37-1)
MJF-R19-7-8
43
TSG101
total
pS910
pS935
pS955
pS973
pS1292
1µg.mL-1
1/1000

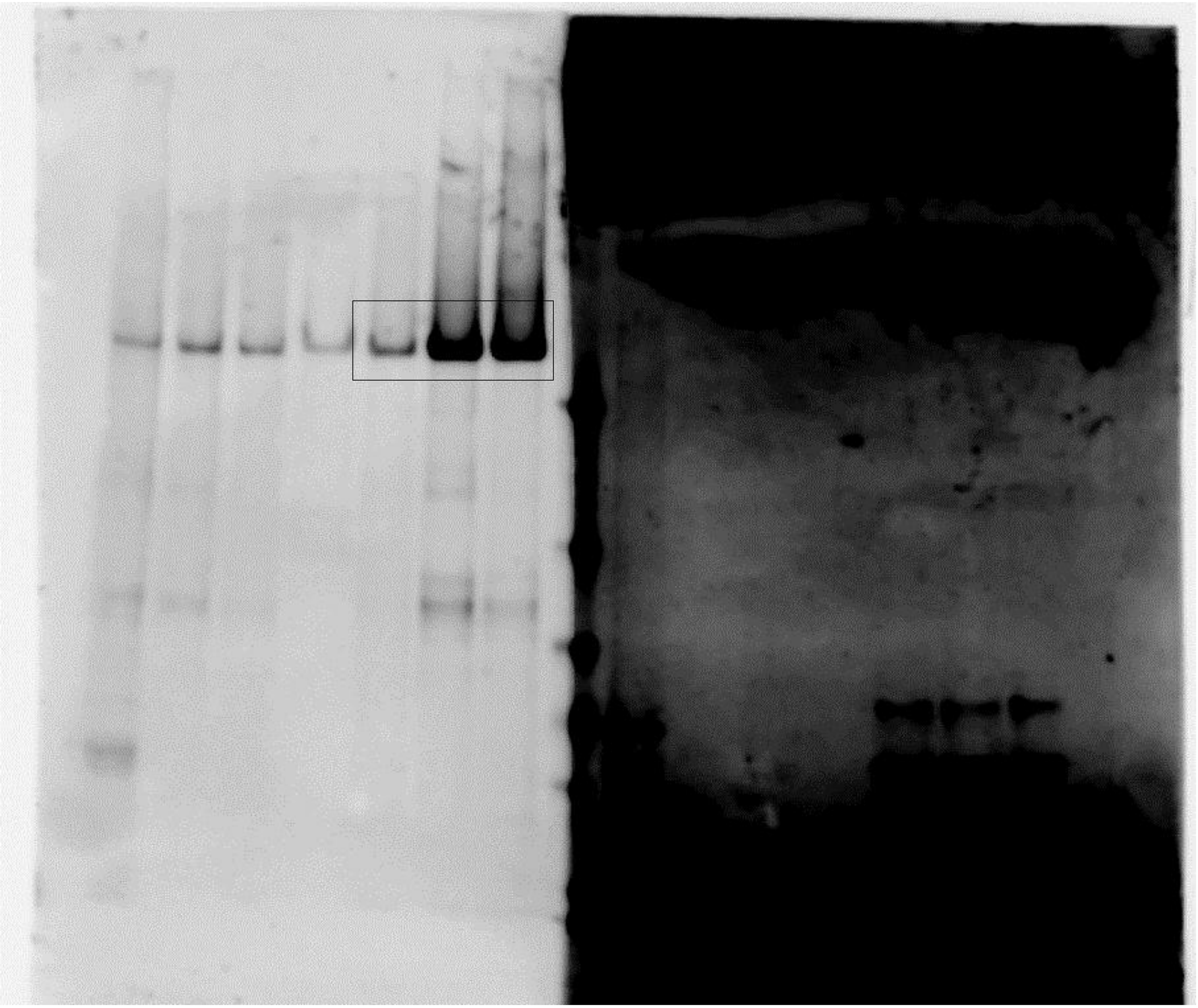

Figure 1d

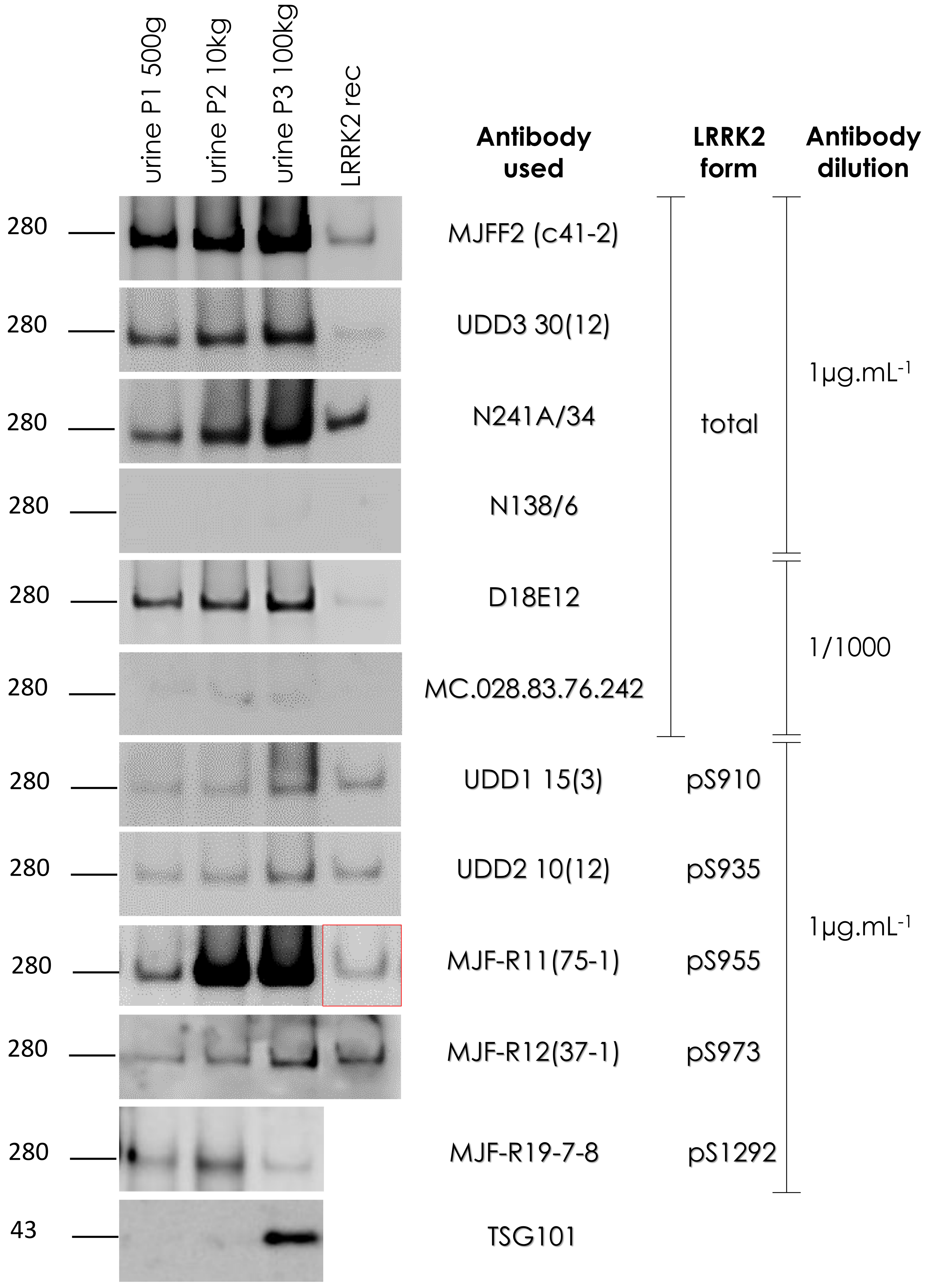

urine P1 500g
urine P2 10kg
urine P3 100kg
LRRK2 rec
Antibody used
LRRK2 form
Antibody dilution
280
MJFF2 (c41-2)
UDD3 30(12)
N241A/34
N138/6
D18E12
MC.028.83.76.242
total
1µg.mL-1
1/1000
UDD1 15(3)
pS910
UDD2 10(12)
pS935
MJF-R11(75-1)
pS955
MJF-R12(37-1)
pS973
MJF-R19-7-8
pS1292
1µg.mL-1
43
TSG101

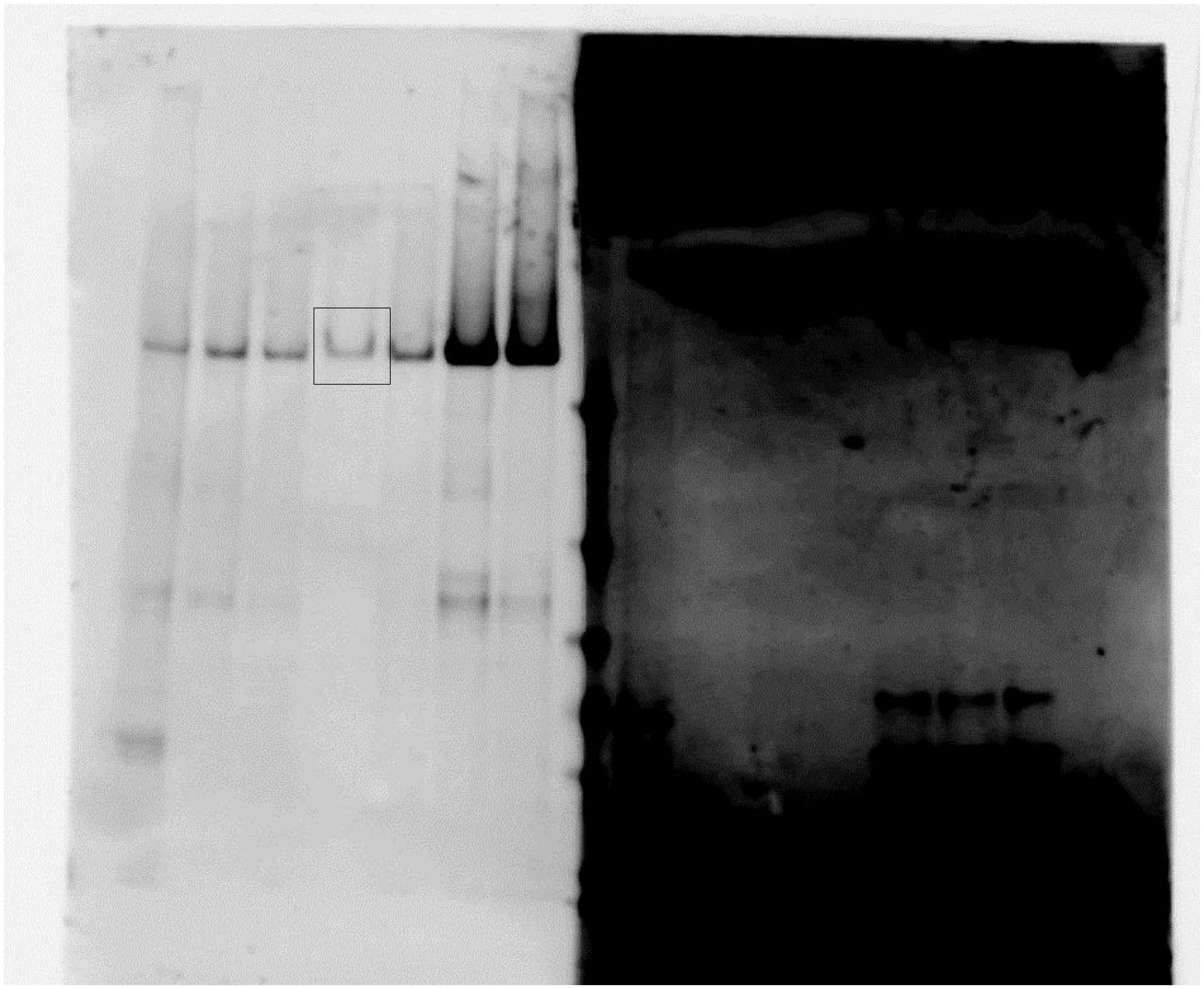

Figure 1d

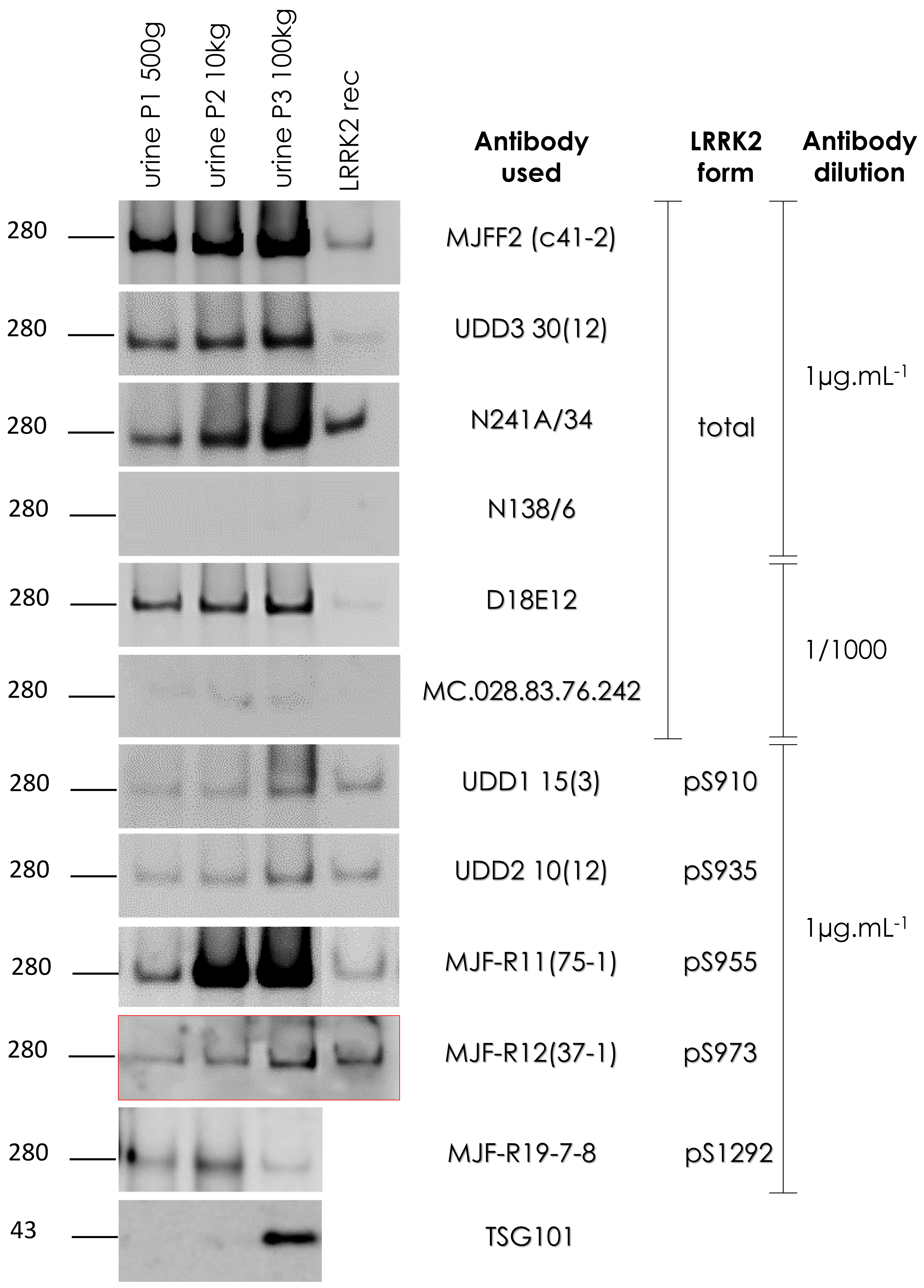

urine P1 500g
urine P2 10kg
urine P3 100kg
LRRK2 rec
Antibody used
LRRK2 form
Antibody dilution
280
MJFF2 (c41-2)
UDD3 30(12)
N241A/34
N138/6
D18E12
MC.028.83.76.242
UDD1 15(3)
UDD2 10(12)
MJF-R11(75-1)
MJF-R12(37-1)
MJF-R19-7-8
TSG101
43
total
pS910
pS935
pS955
pS973
pS1292
1µg.mL-1
1/1000
1µg.mL-1

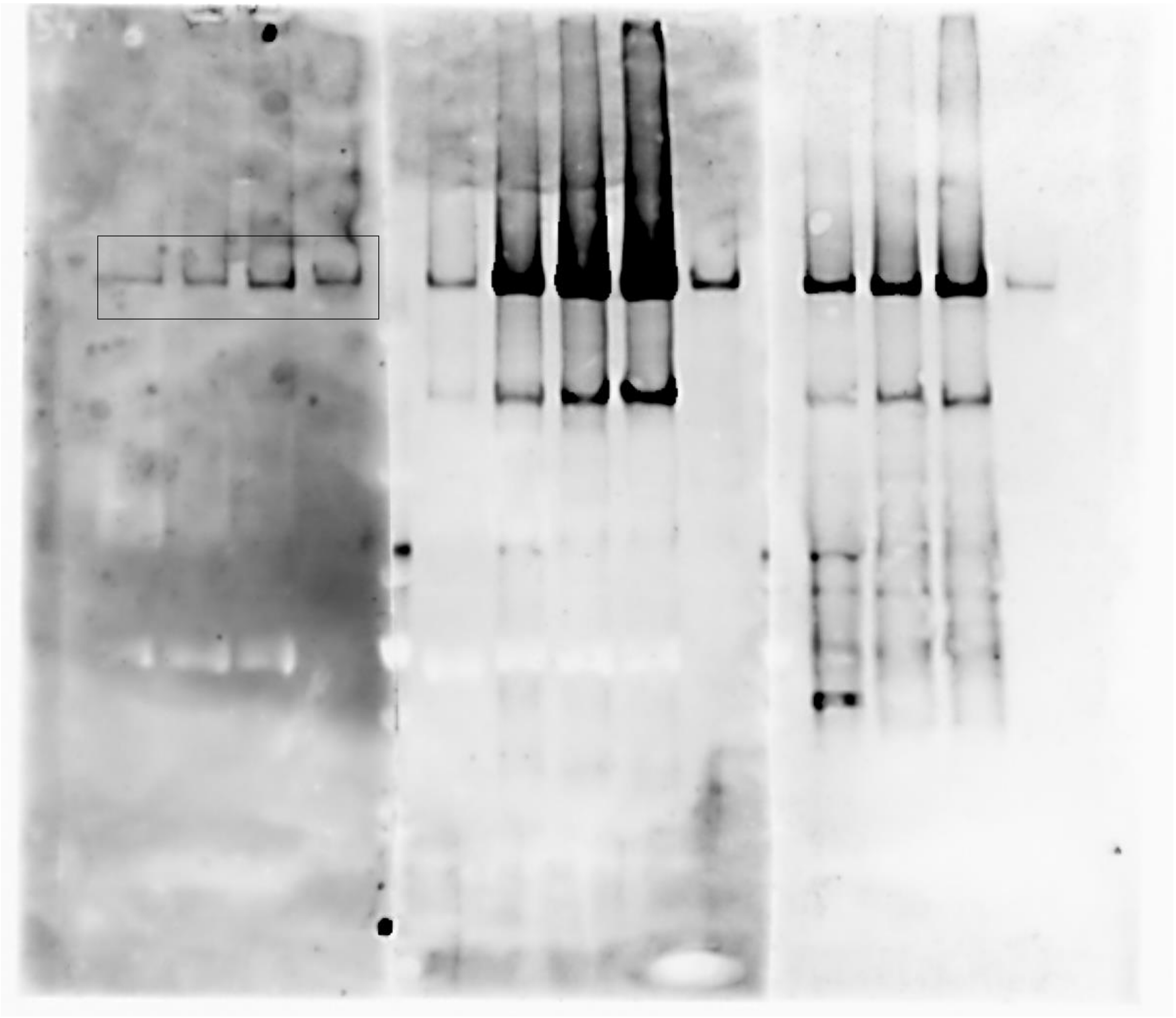

Figure 1d

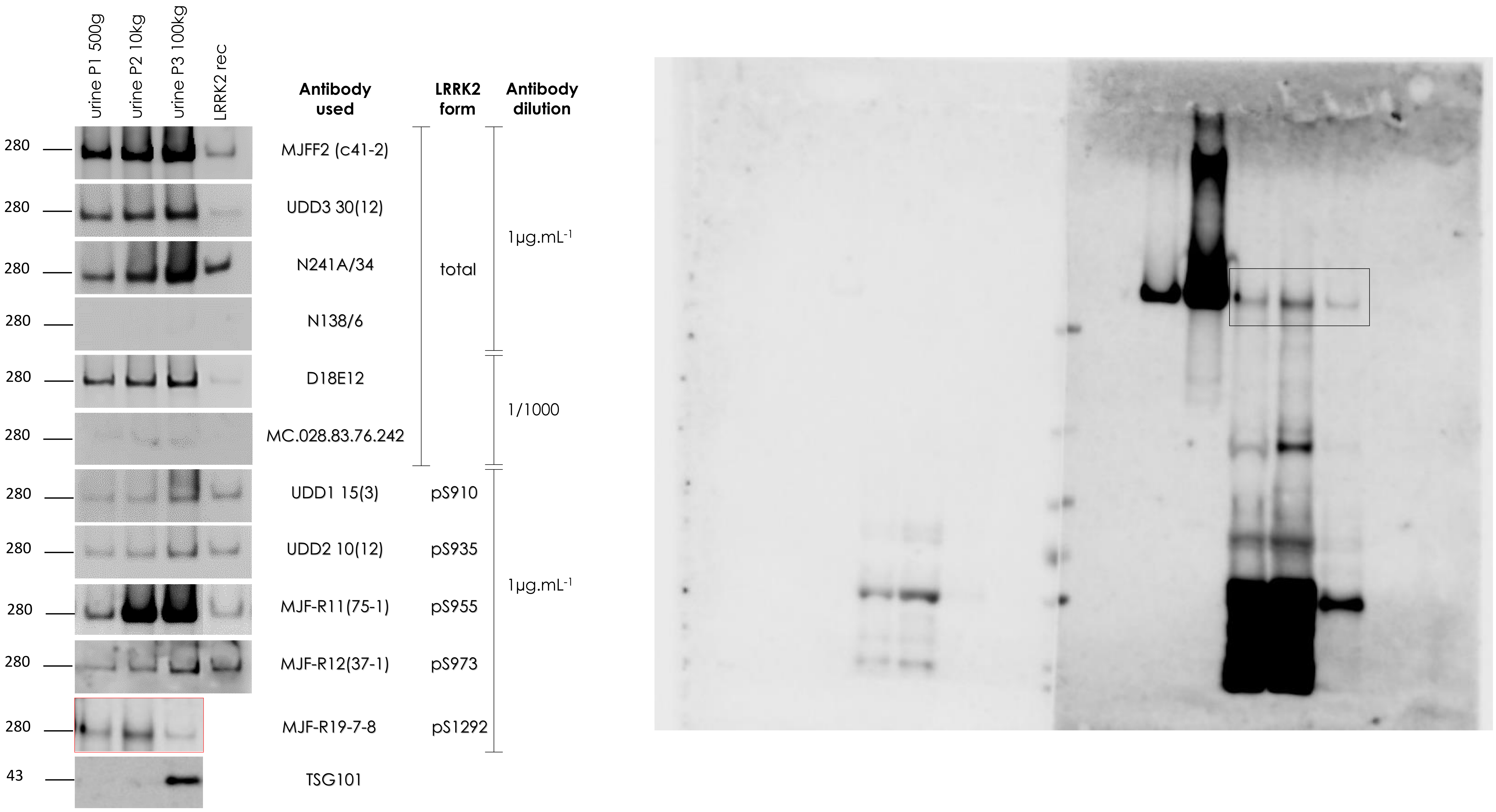
urine P1 500g
urine P2 10kg
urine P3 100kg
LRRK2 rec
Antibody used
LRRK2 form
Antibody dilution
280
MJFF2 (c41-2)
UDD3 30(12)
N241A/34
N138/6
D18E12
MC.028.83.76.242
total
1µg.mL-1
1/1000
UDD1 15(3)
pS910
UDD2 10(12)
pS935
MJF-R11(75-1)
pS955
MJF-R12(37-1)
pS973
MJF-R19-7-8
pS1292
43
TSG101

Figure 1d

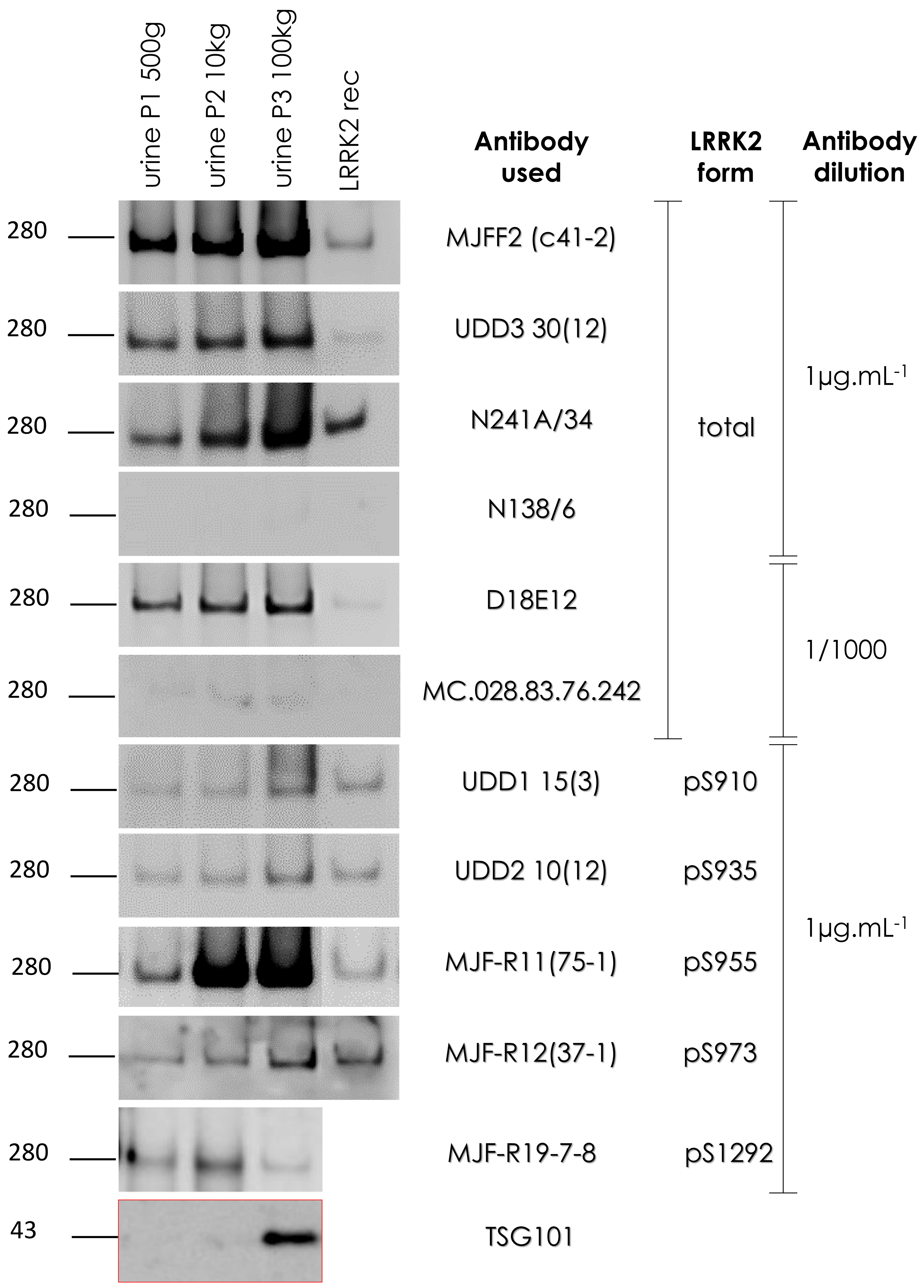

urine P1 500g
urine P2 10kg
urine P3 100kg
LRRK2 rec
Antibody used
LRRK2 form
Antibody dilution
280
MJFF2 (c41-2)
UDD3 30(12)
N241A/34
N138/6
D18E12
MC.028.83.76.242
UDD1 15(3)
UDD2 10(12)
MJF-R11(75-1)
MJF-R12(37-1)
MJF-R19-7-8
43
TSG101
total
pS910
pS935
pS955
pS973
pS1292
1μg.mL-1
1/1000
1μg.mL-1

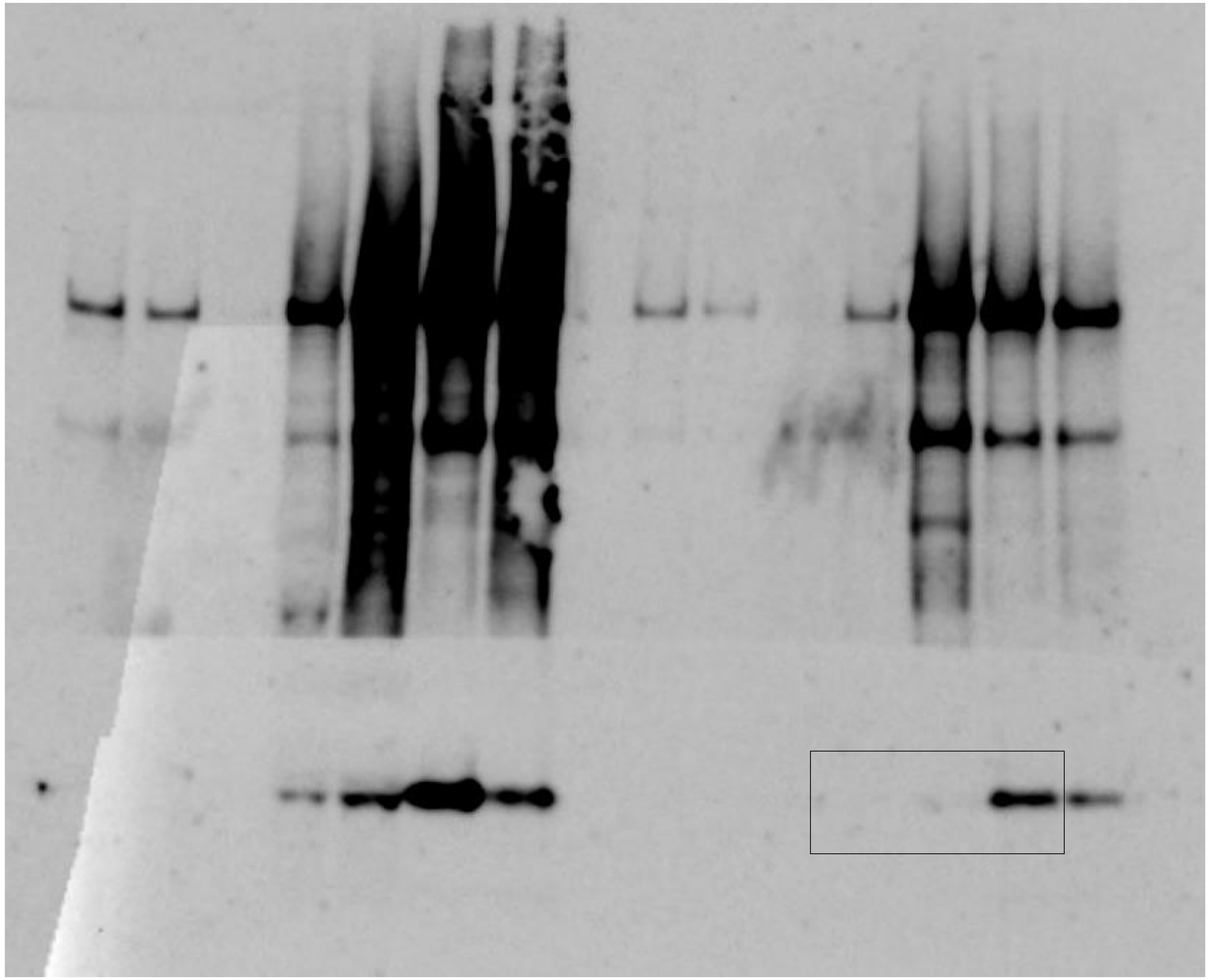

Figure 5a

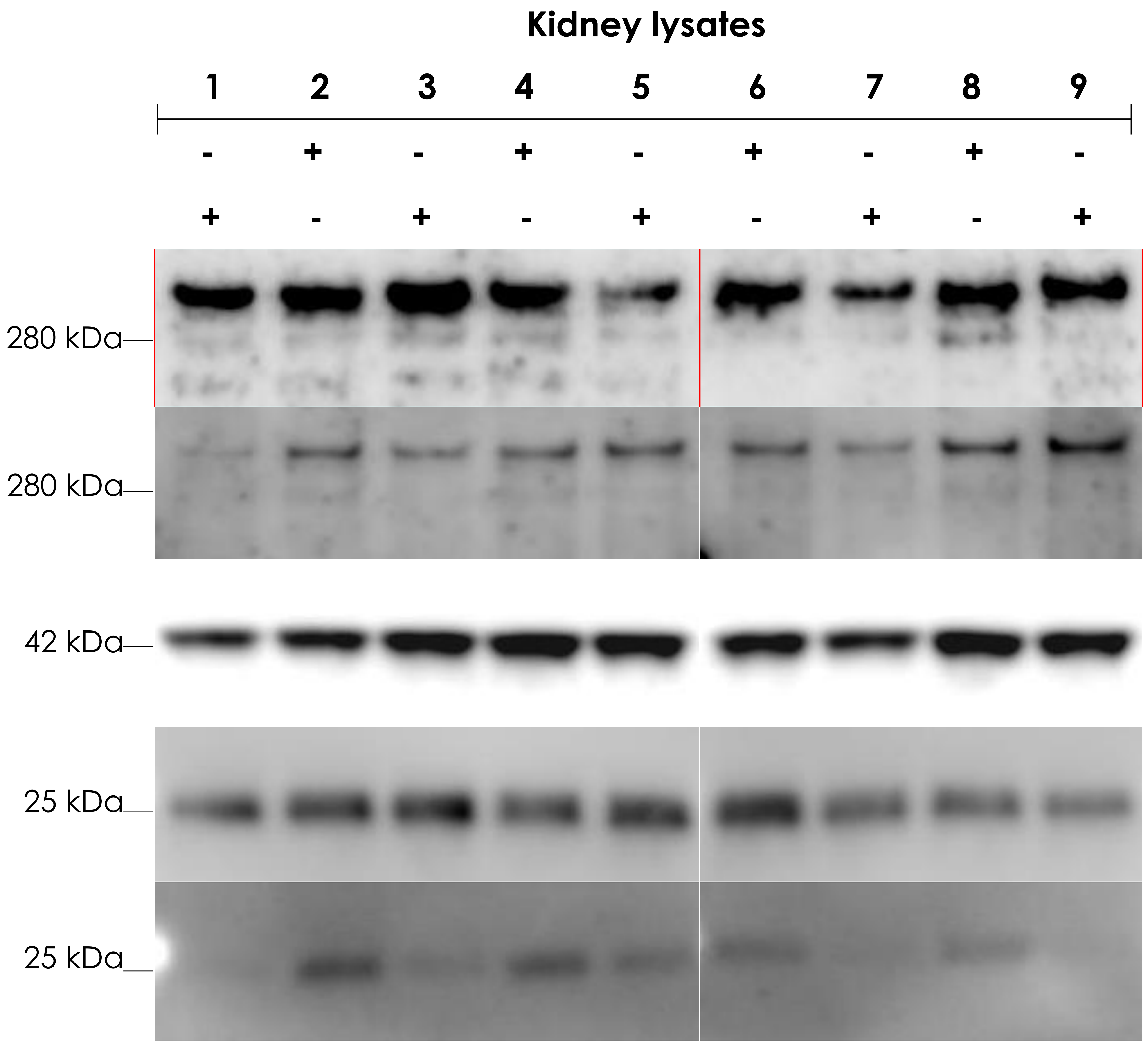

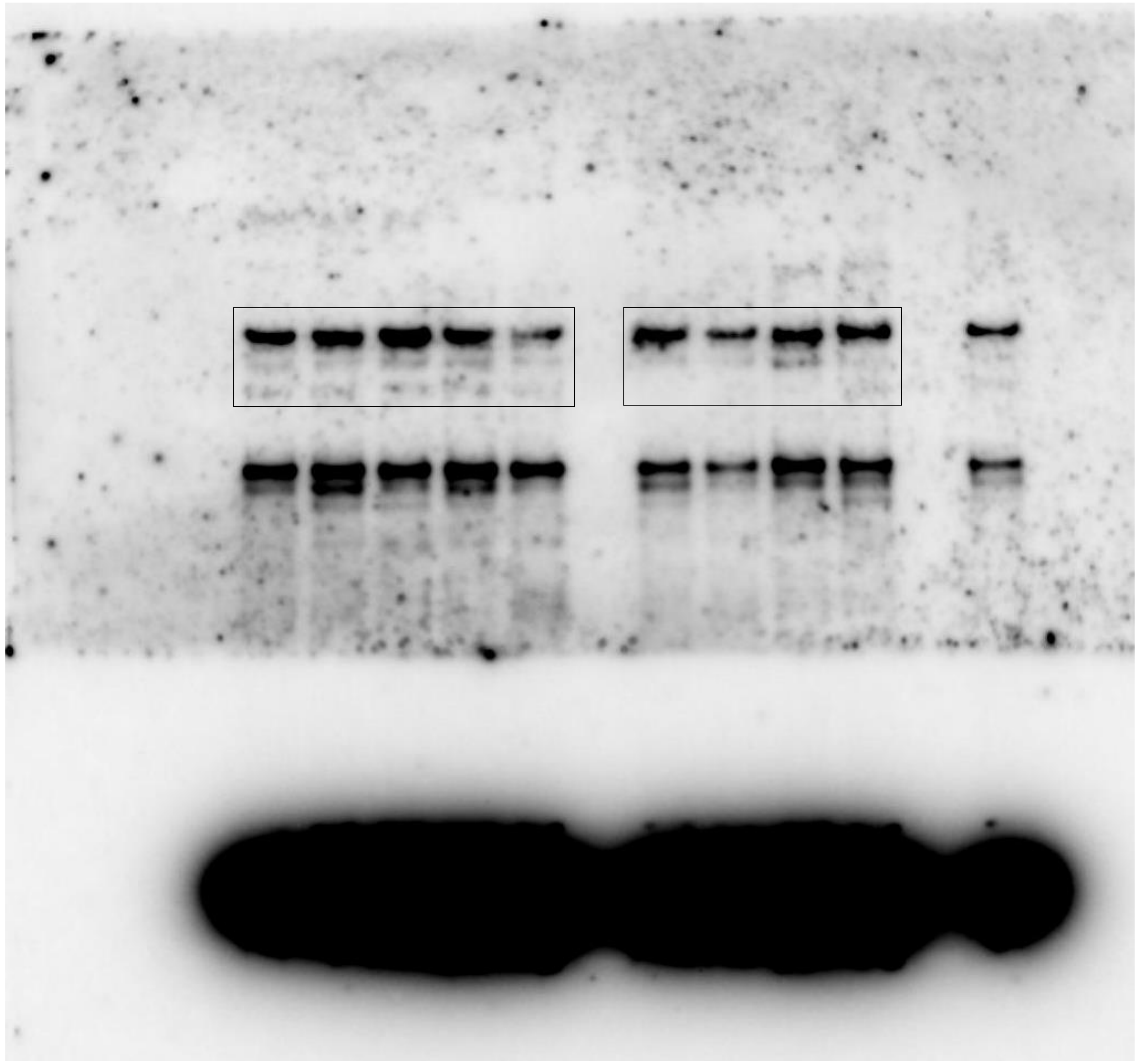

Figure 5a

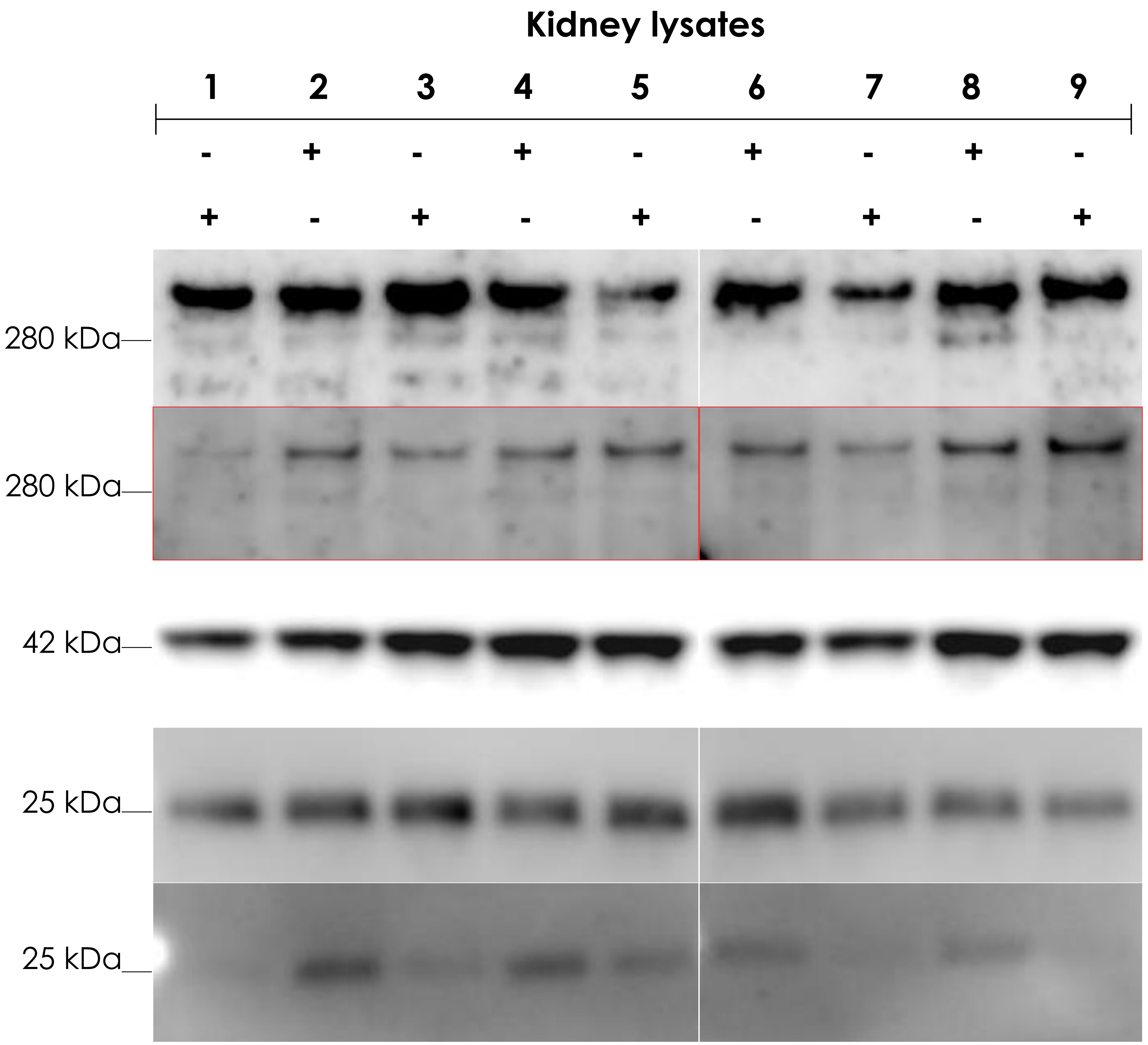

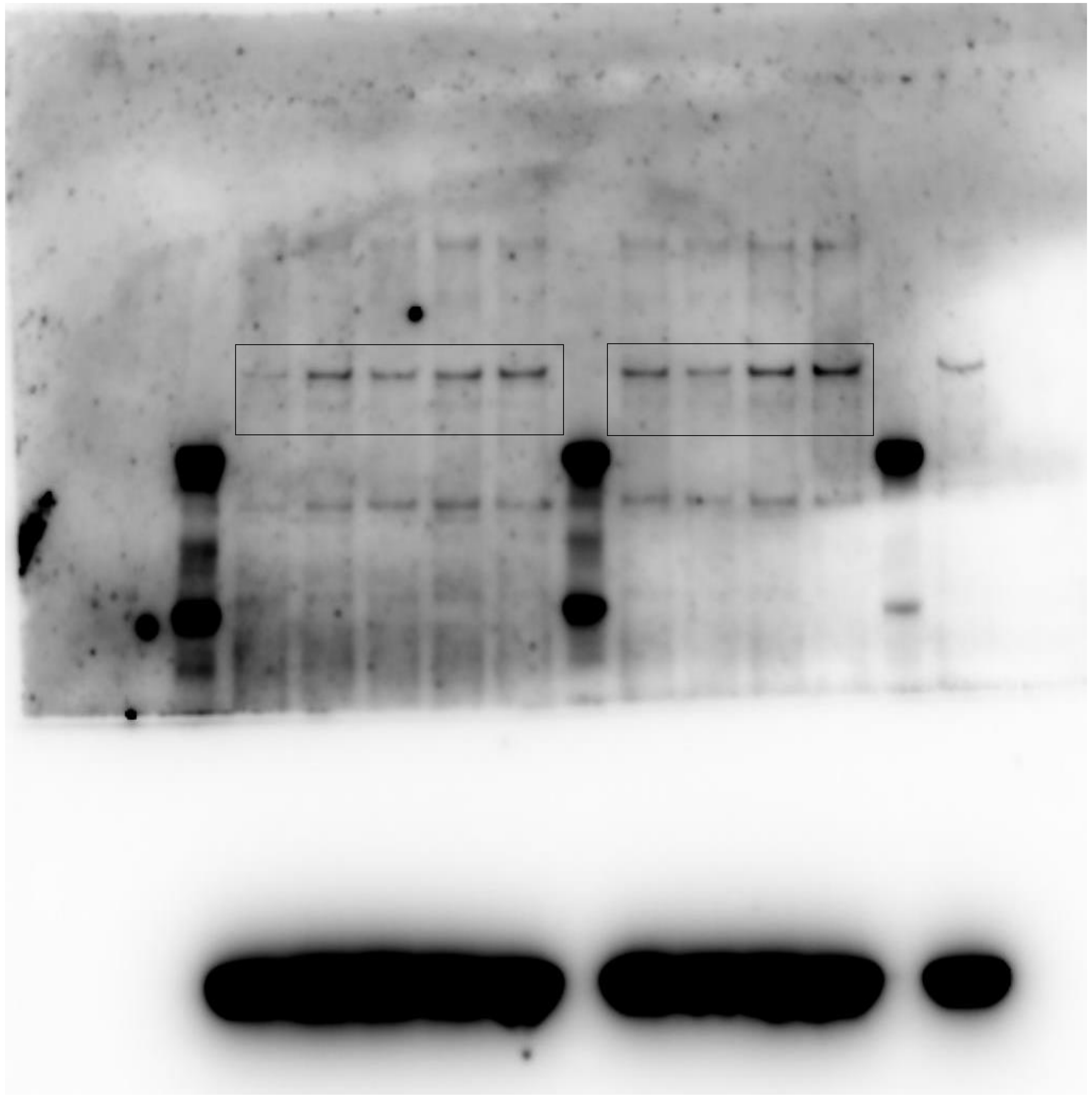

Figure 5a

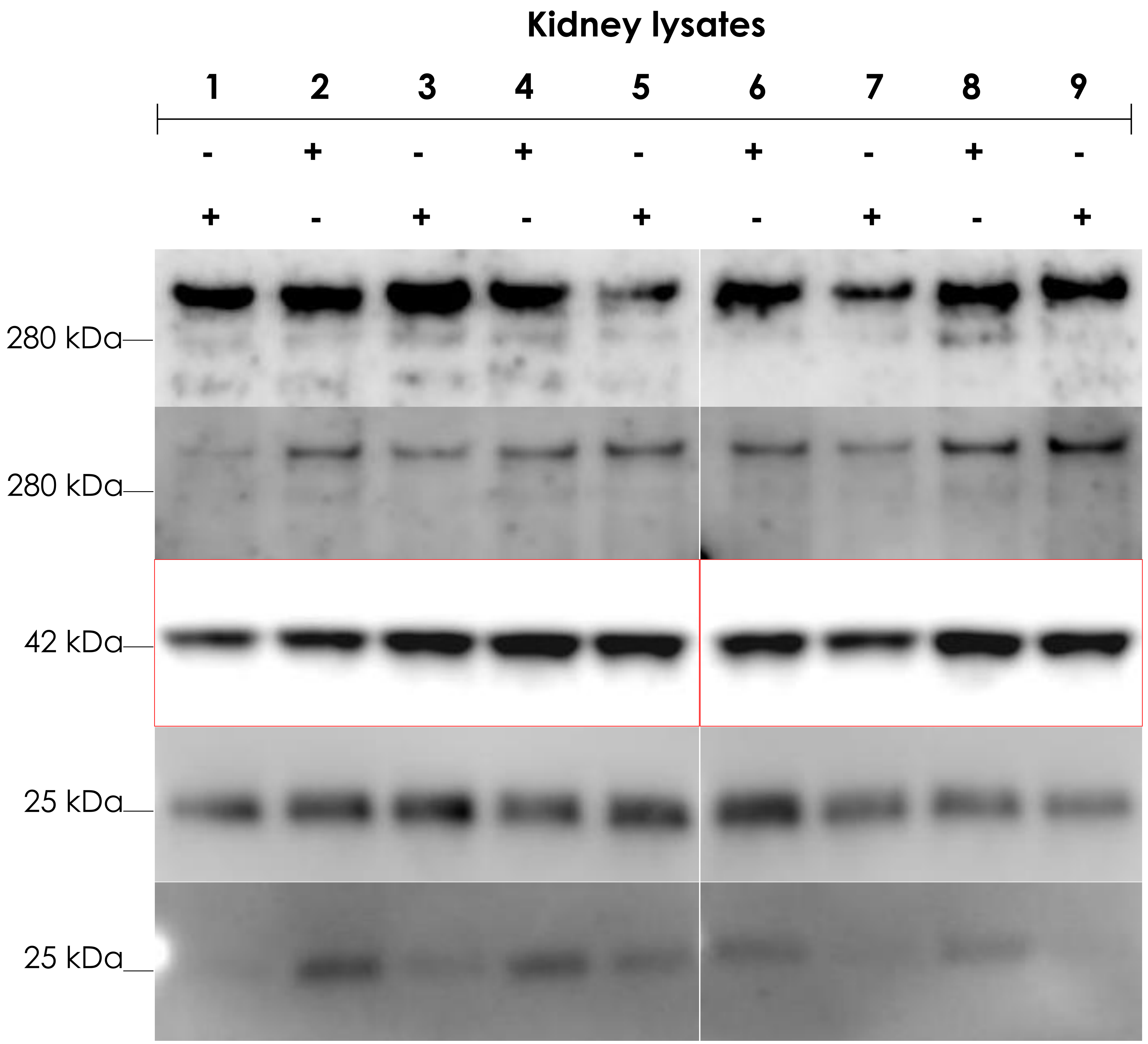

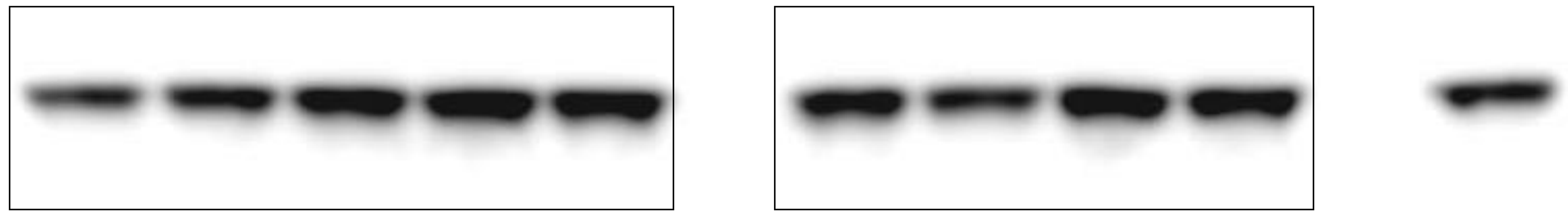

Figure 5a

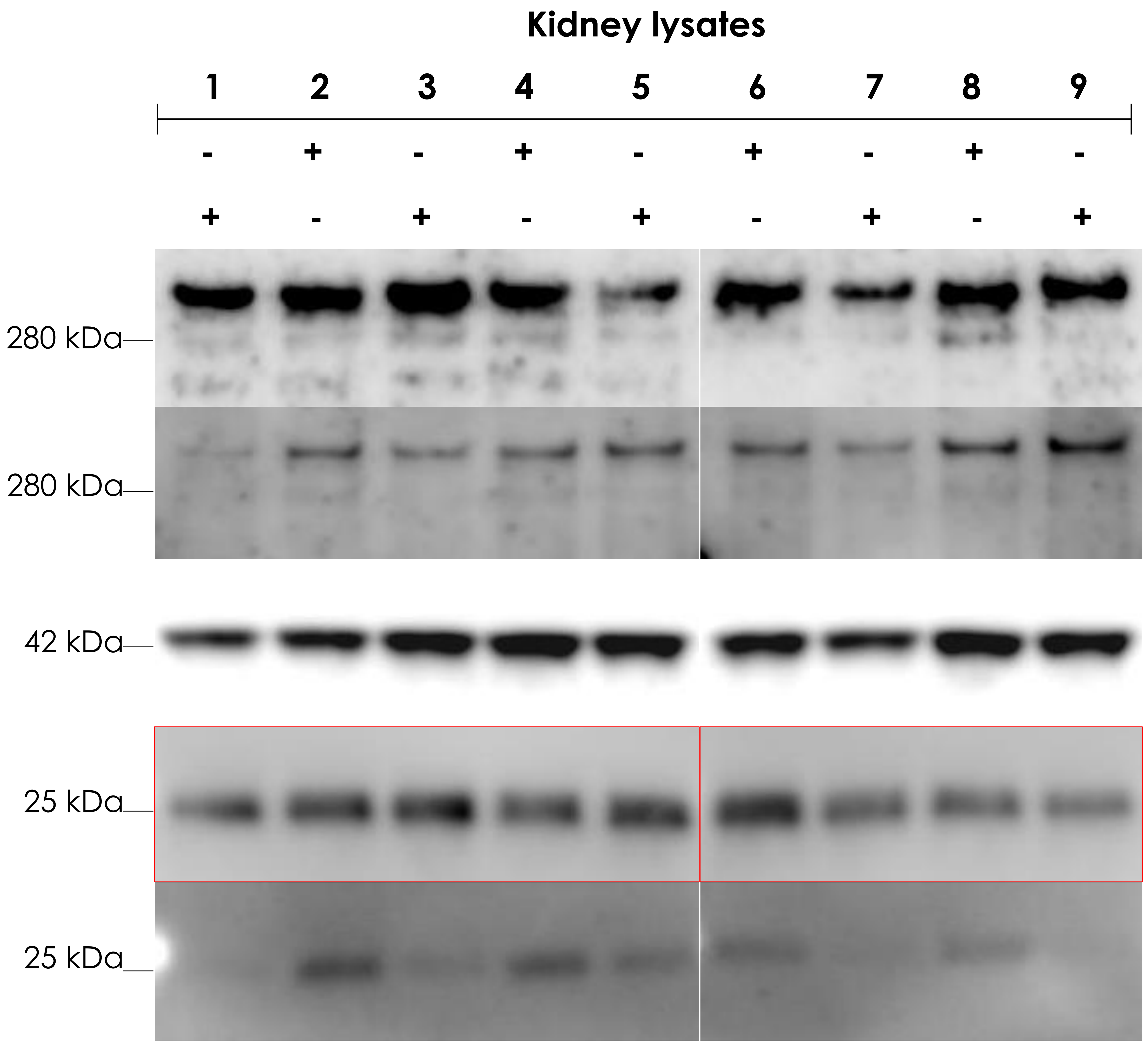

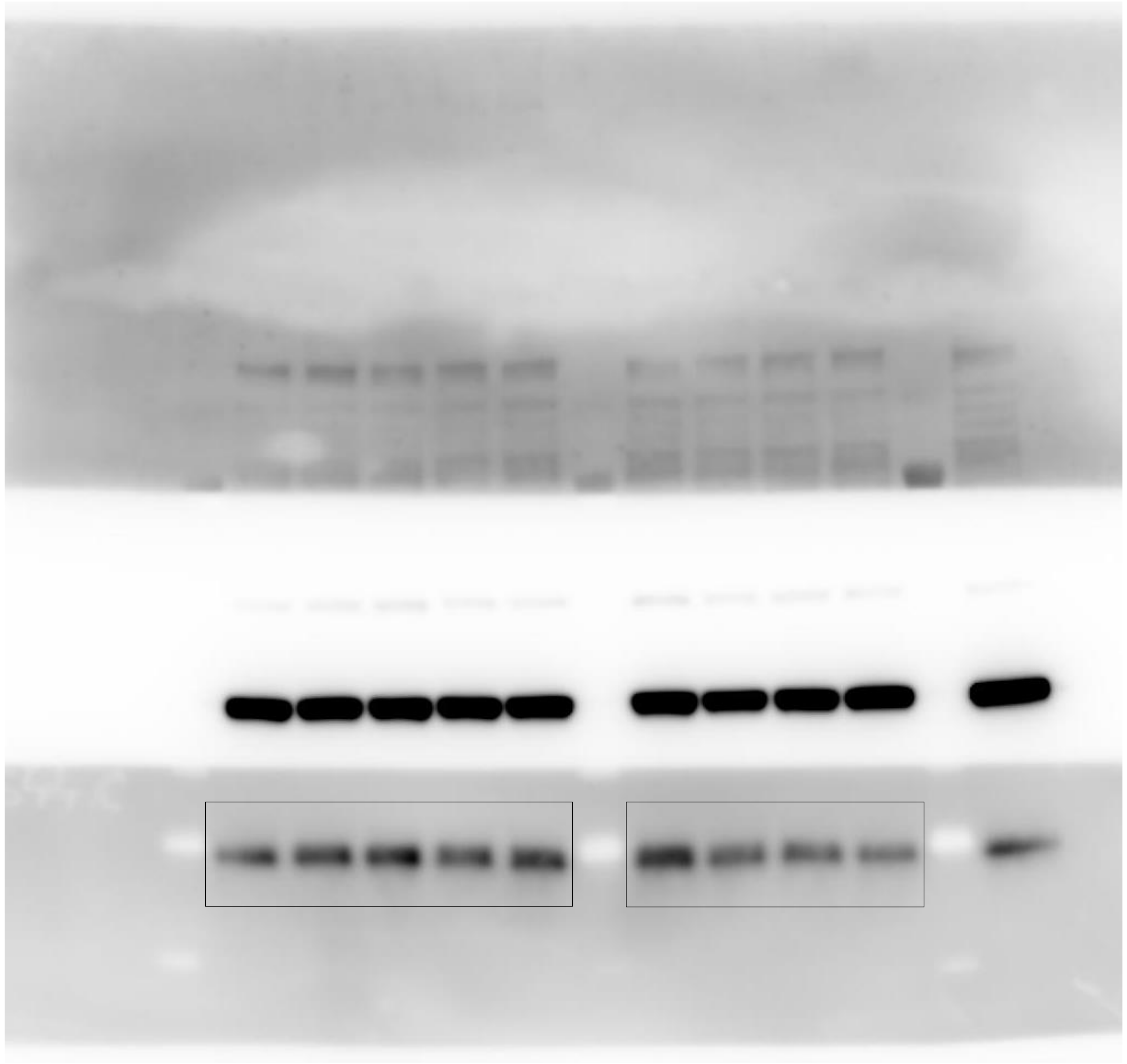

Figure 5a

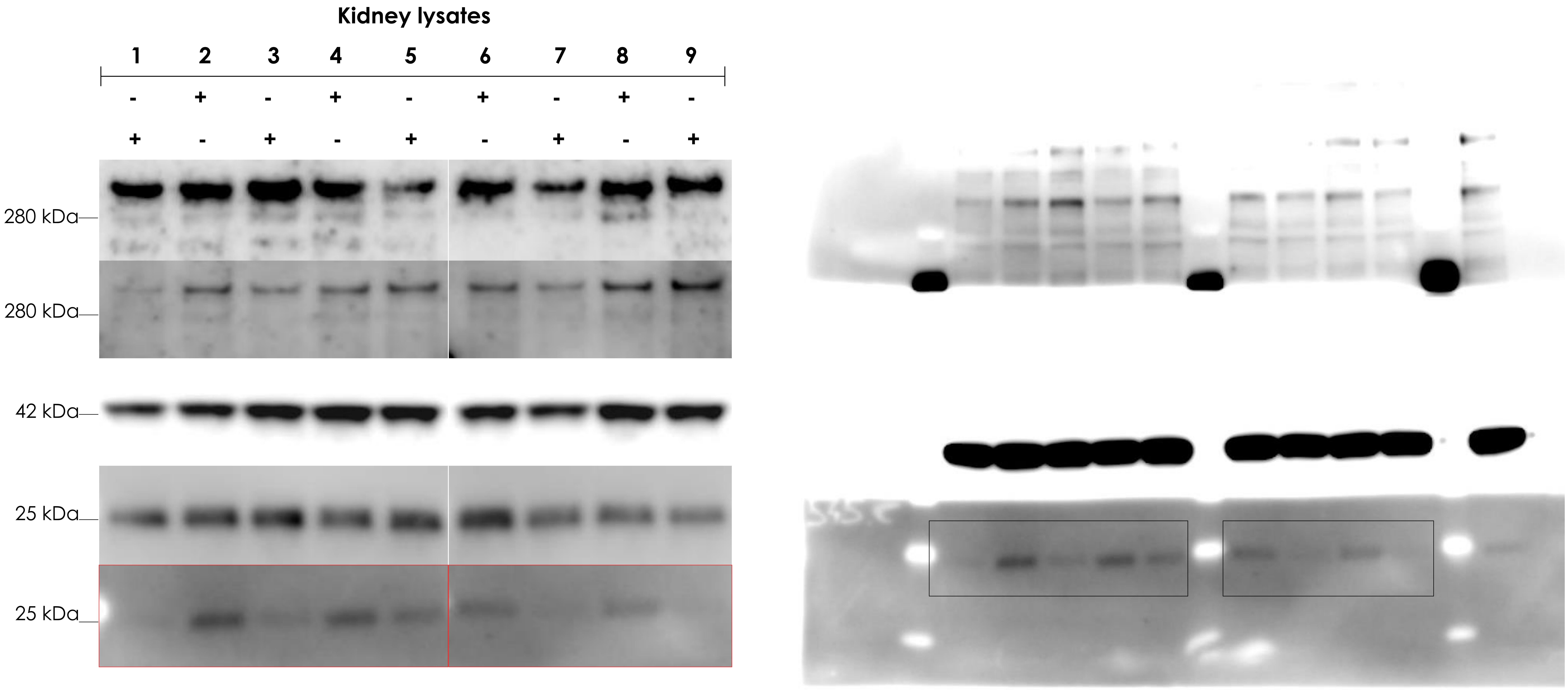

Figure 5a

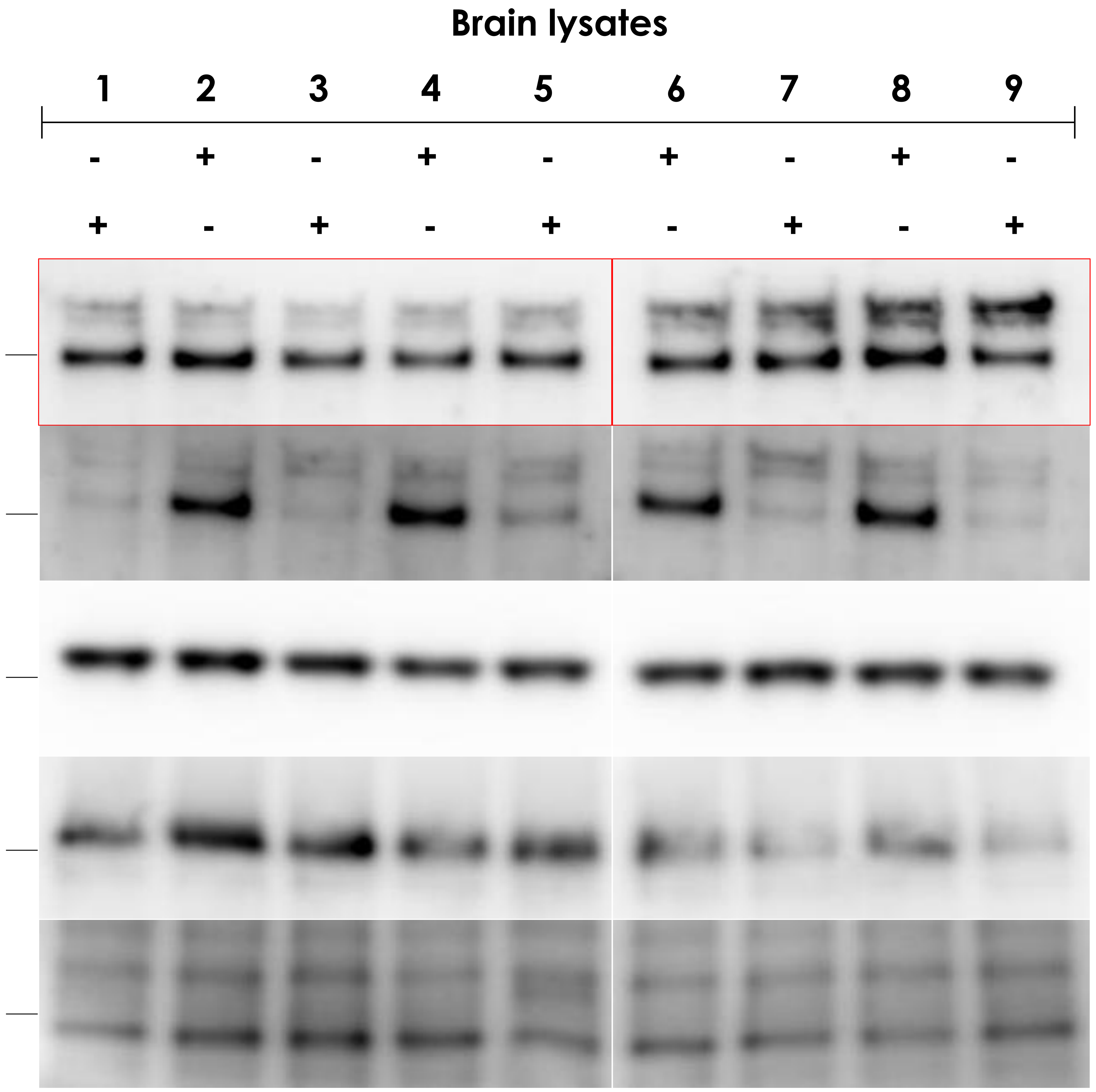

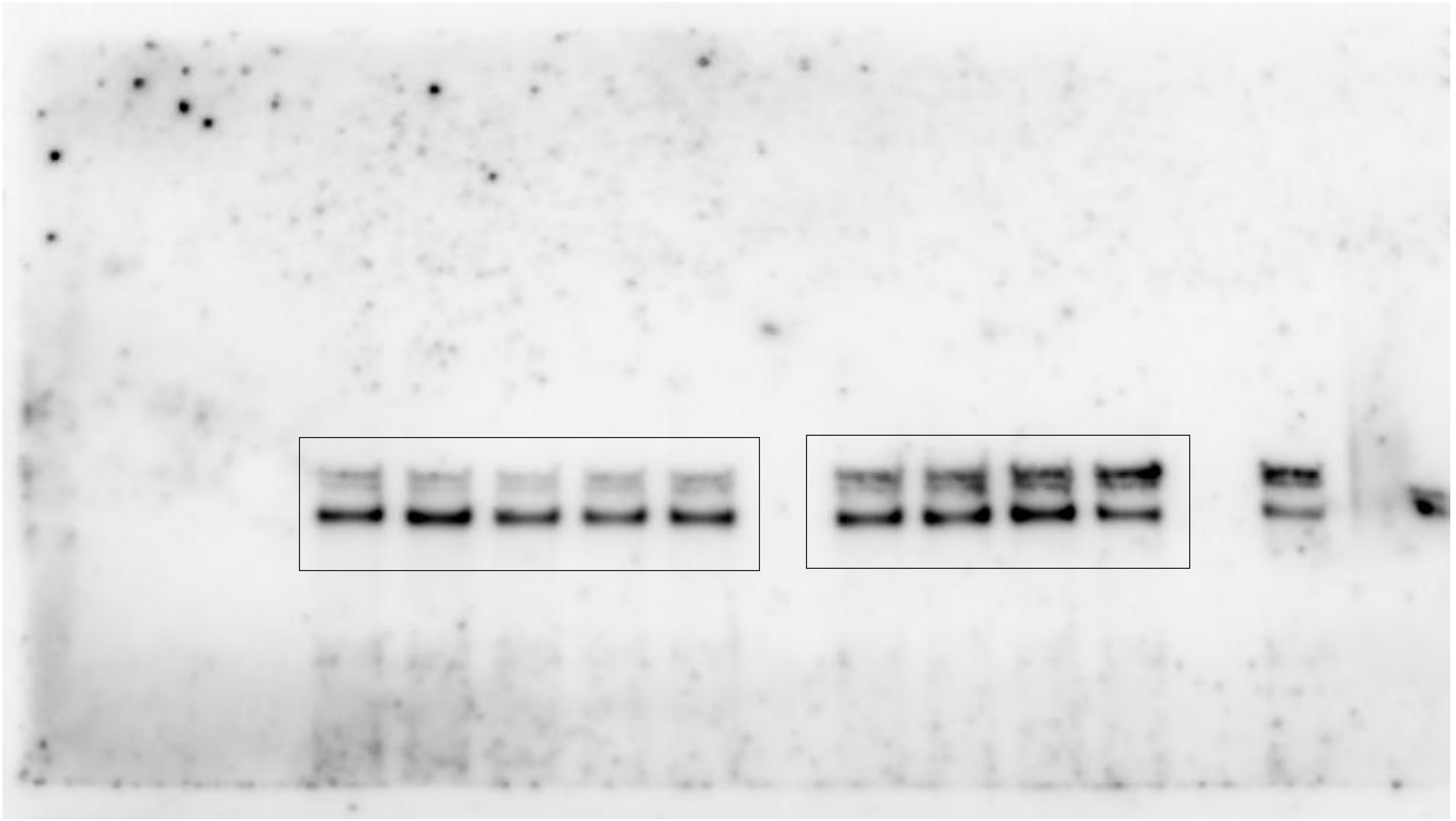

Figure 5a

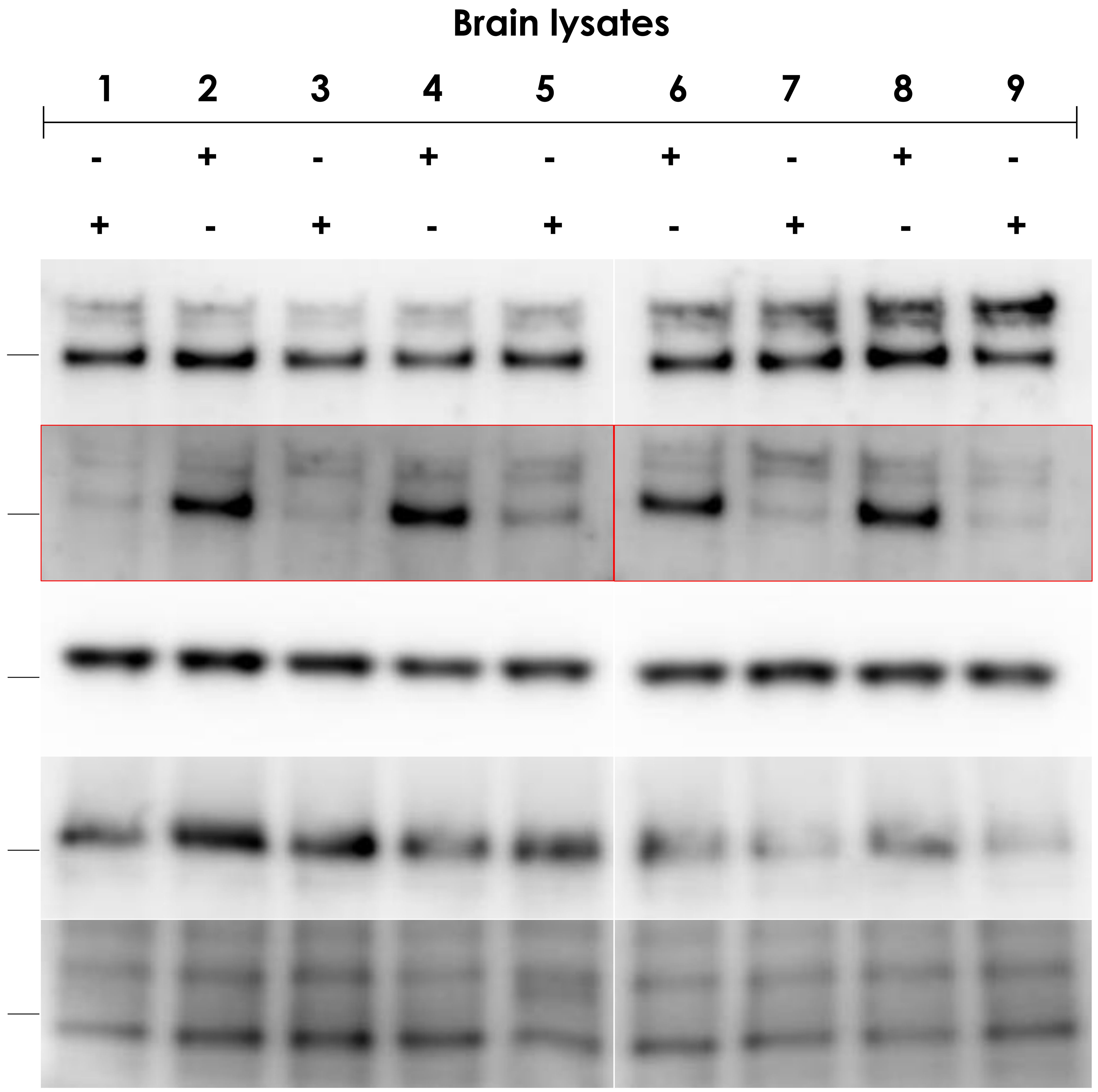

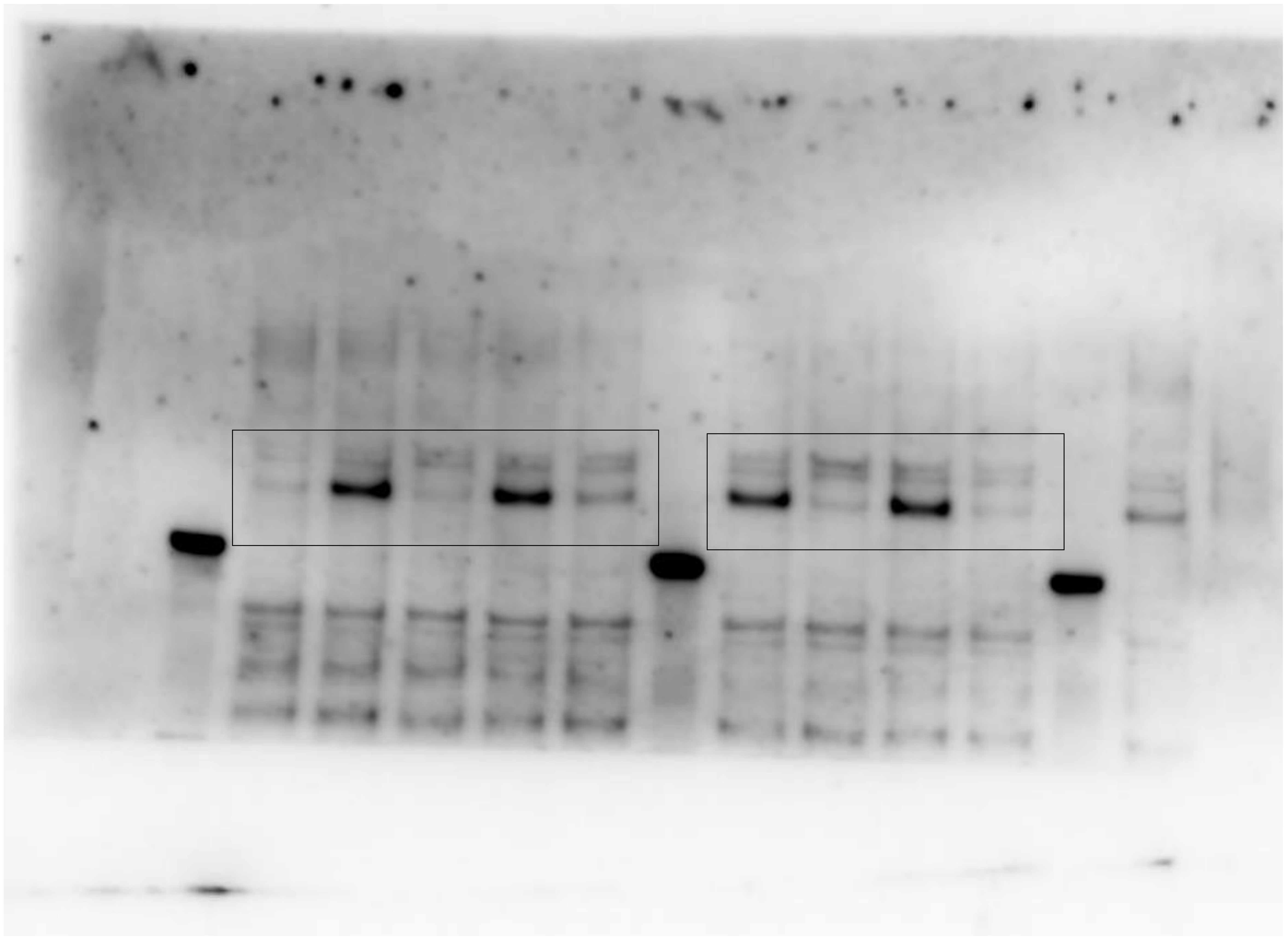

Figure 5a

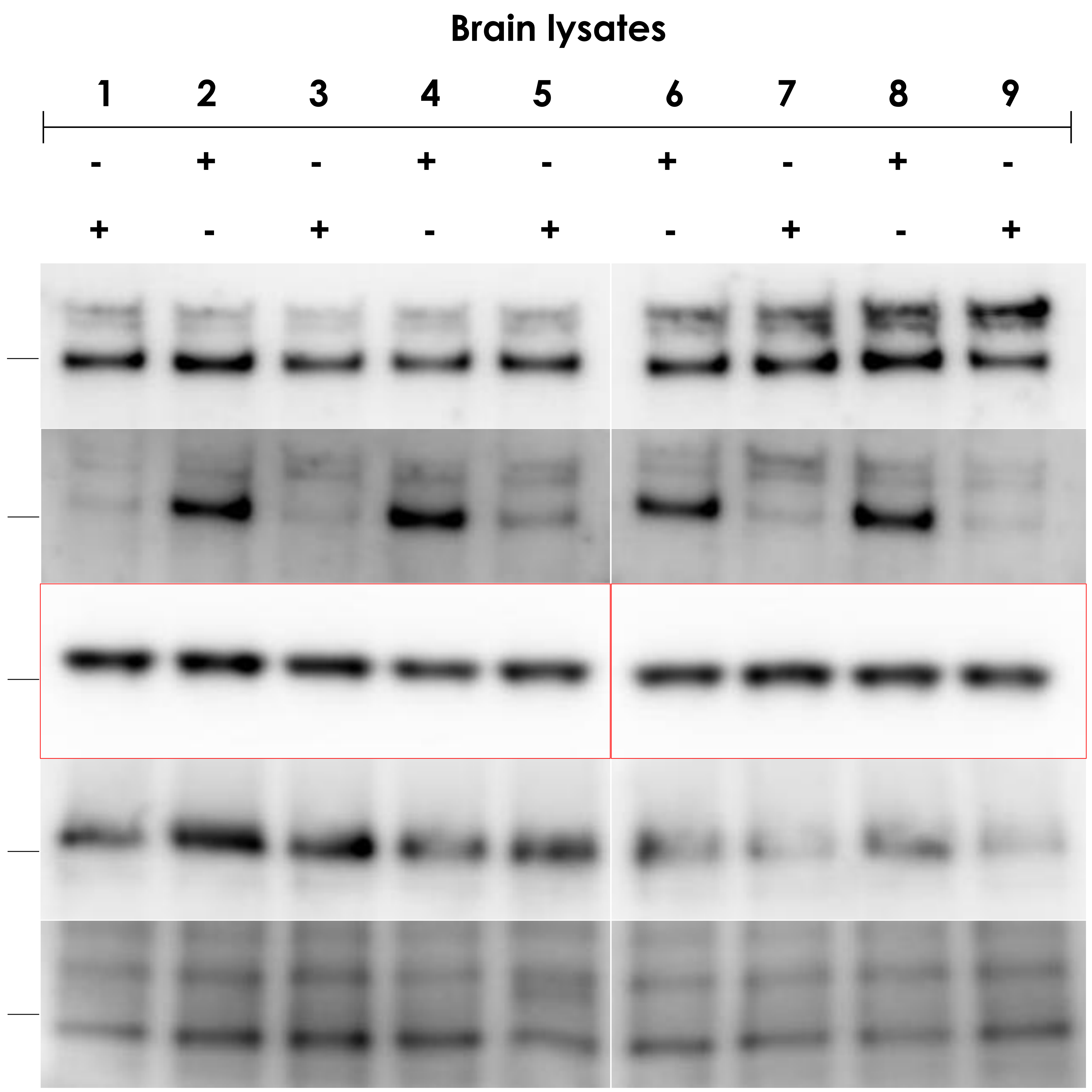

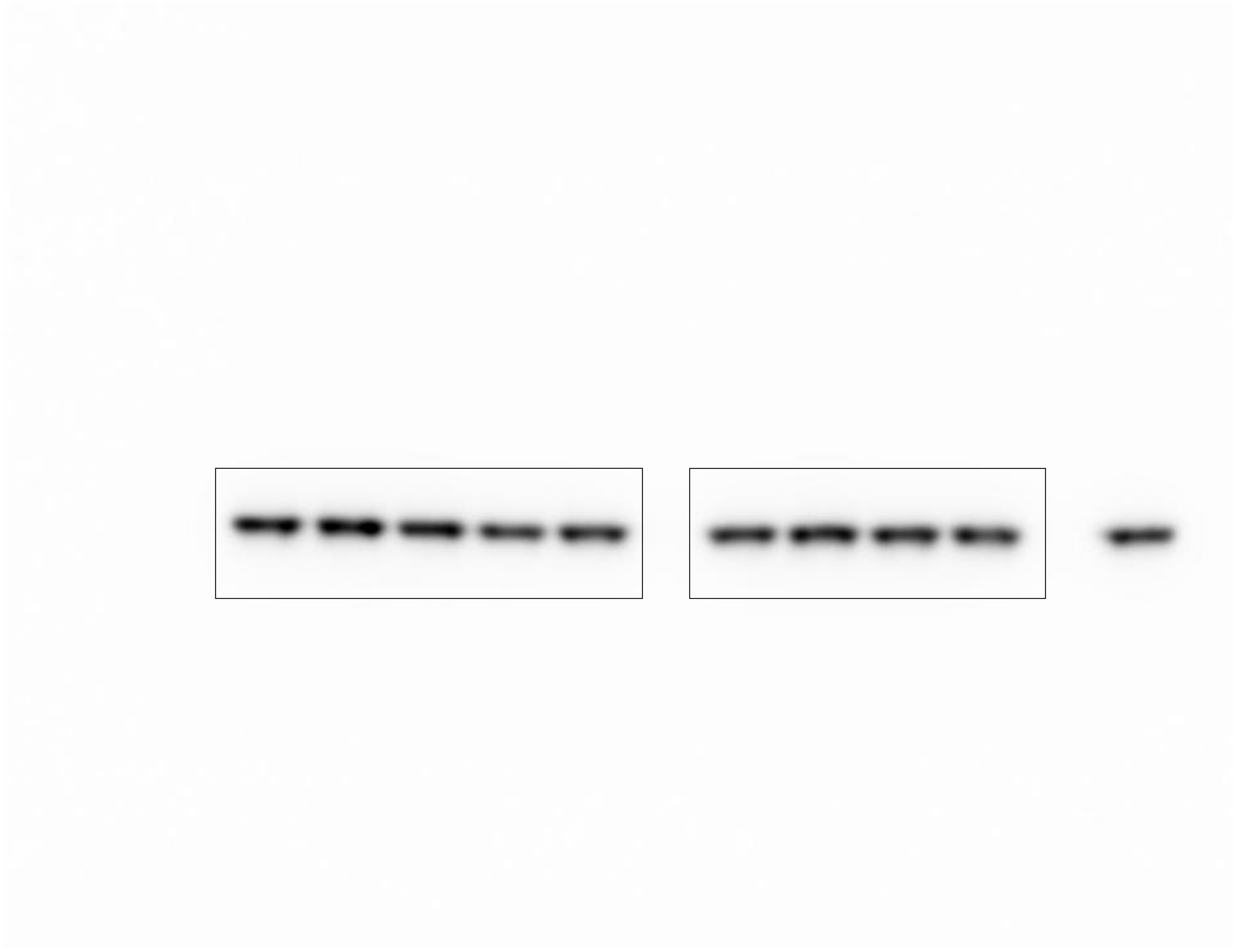

Figure 5a

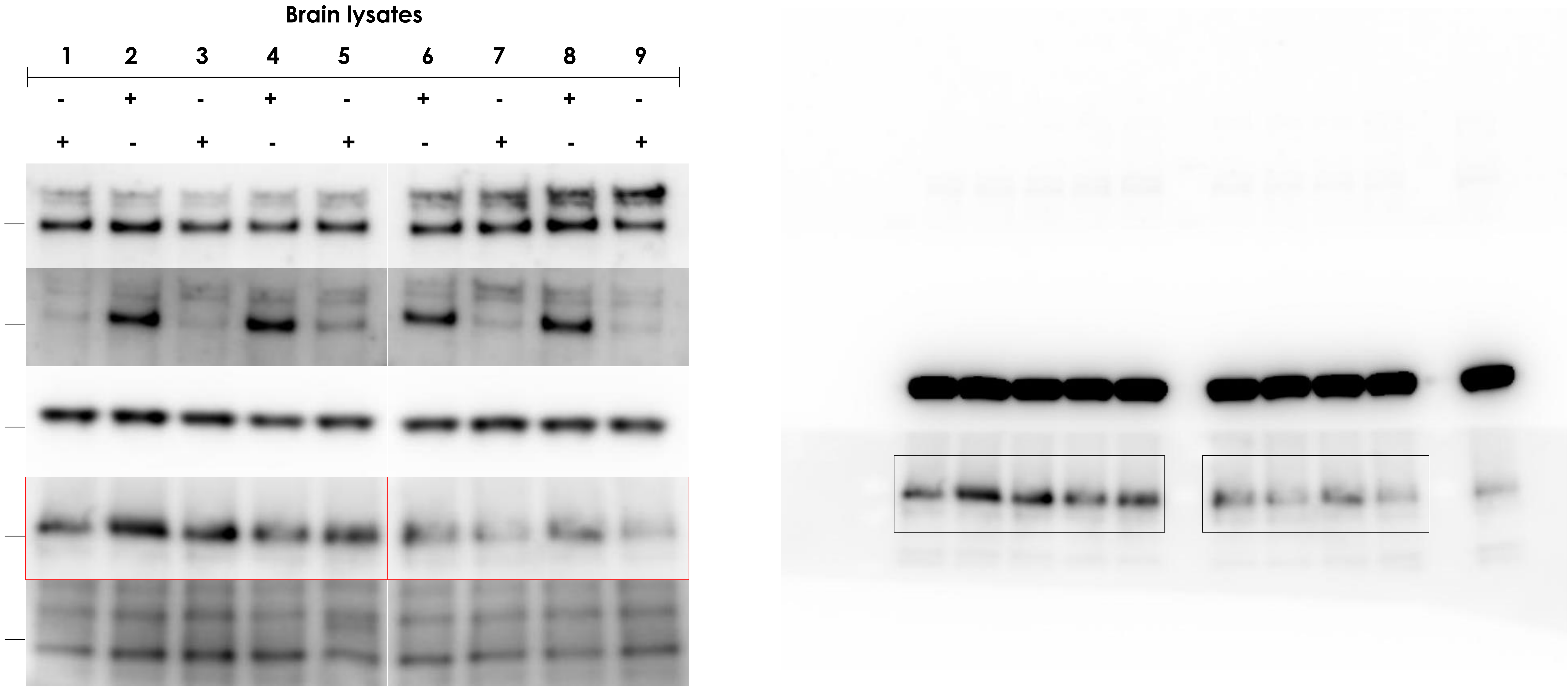

Figure 5a

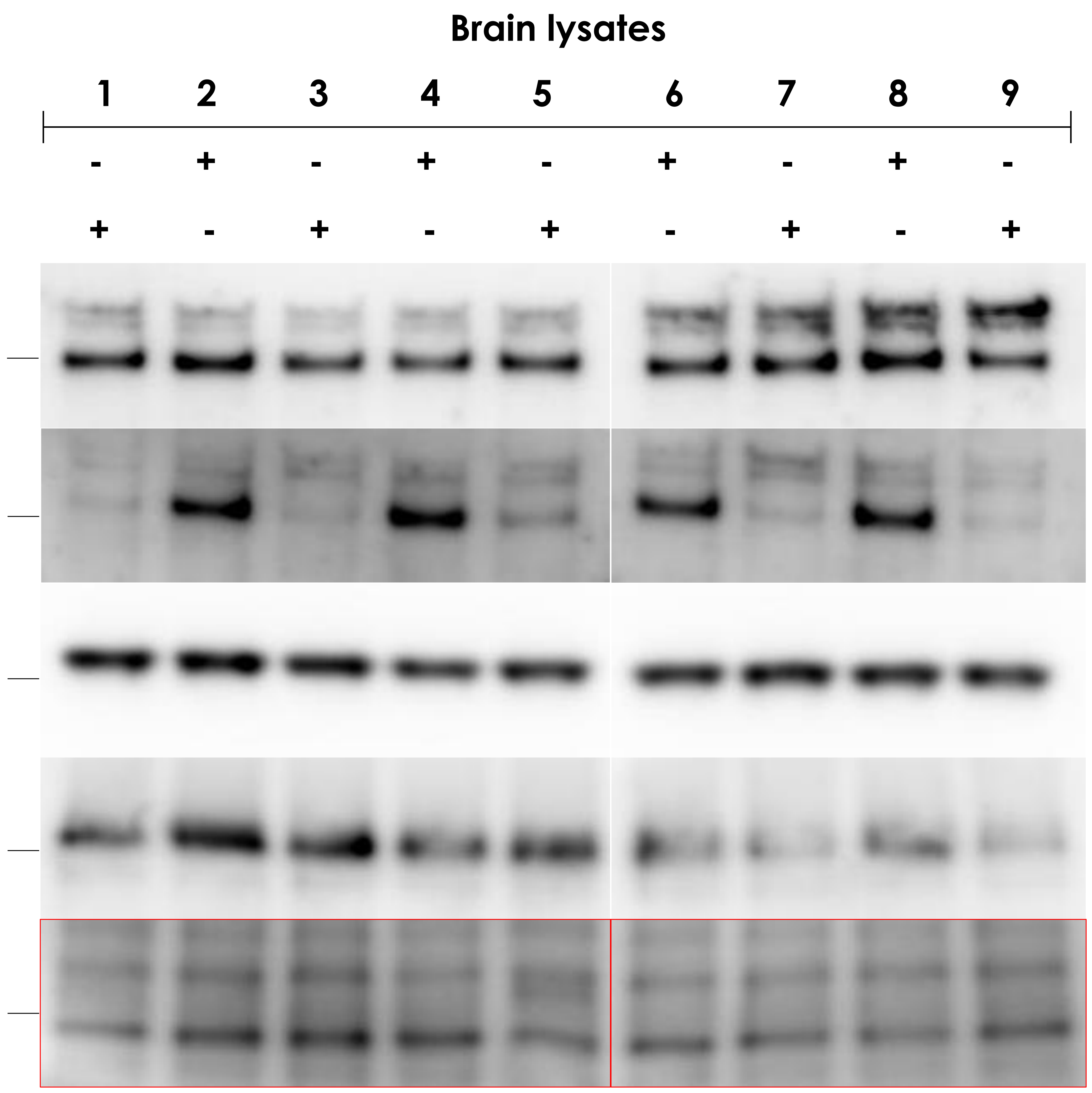

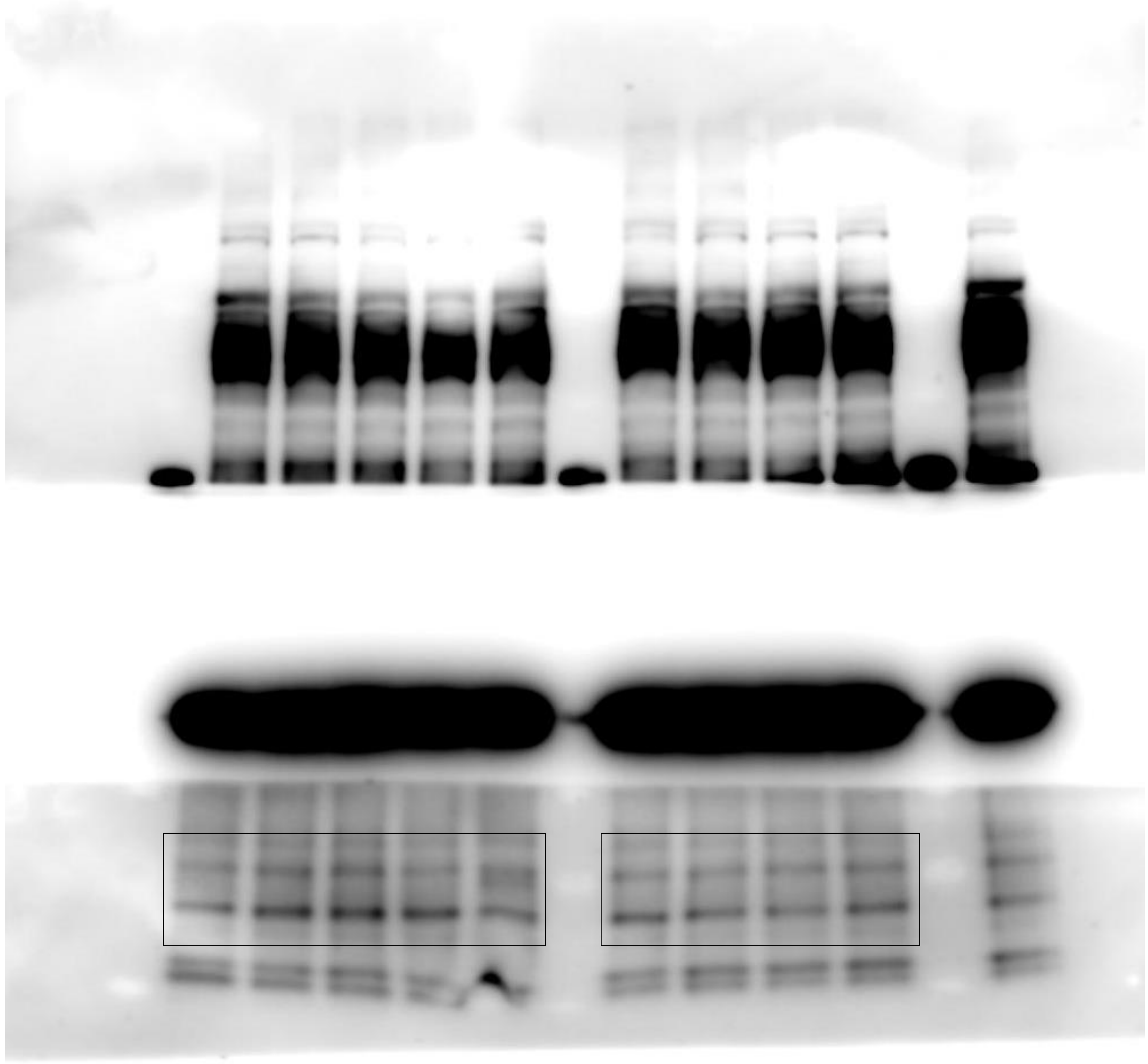

Figure 5a

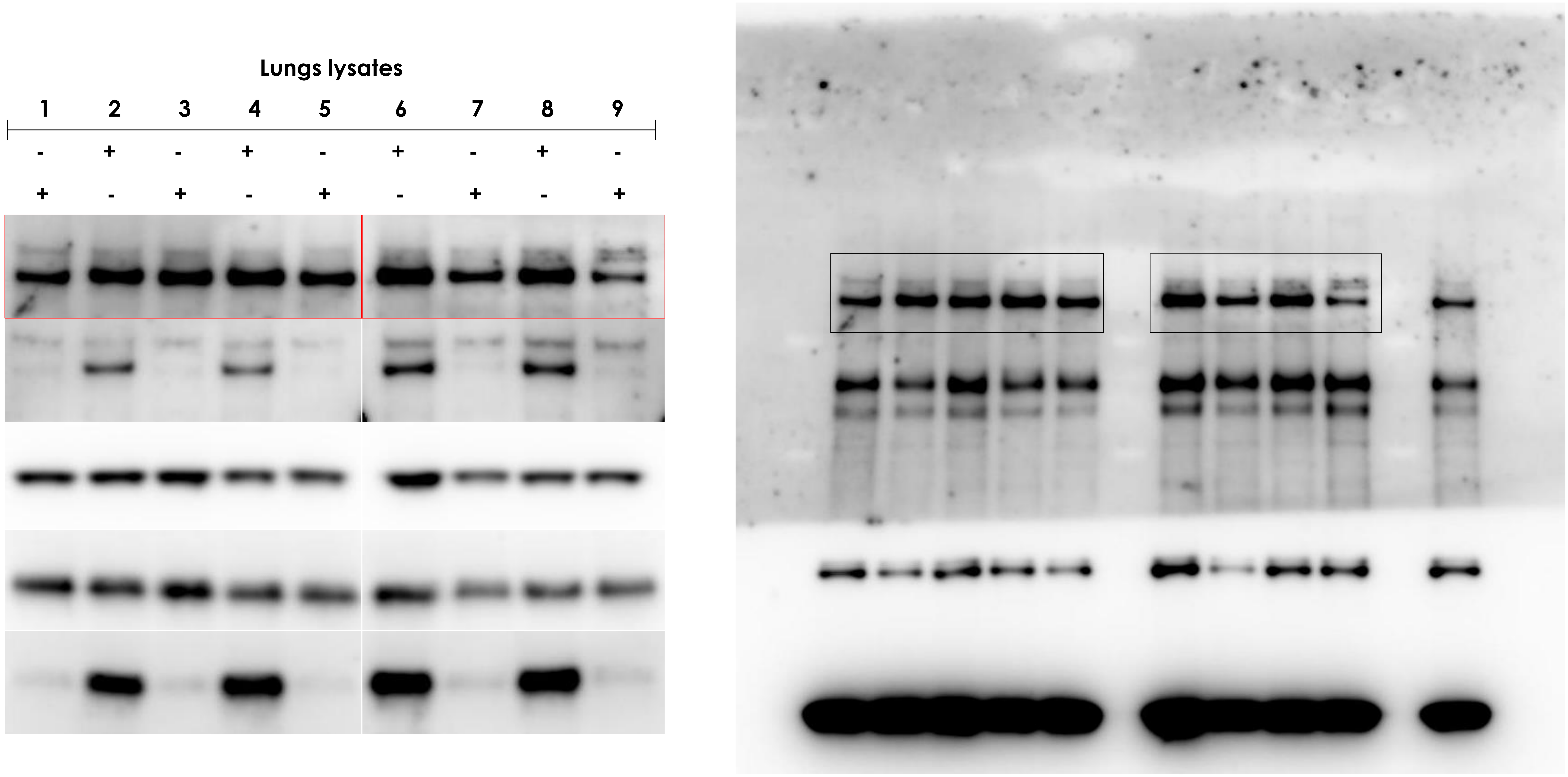

Figure 5a

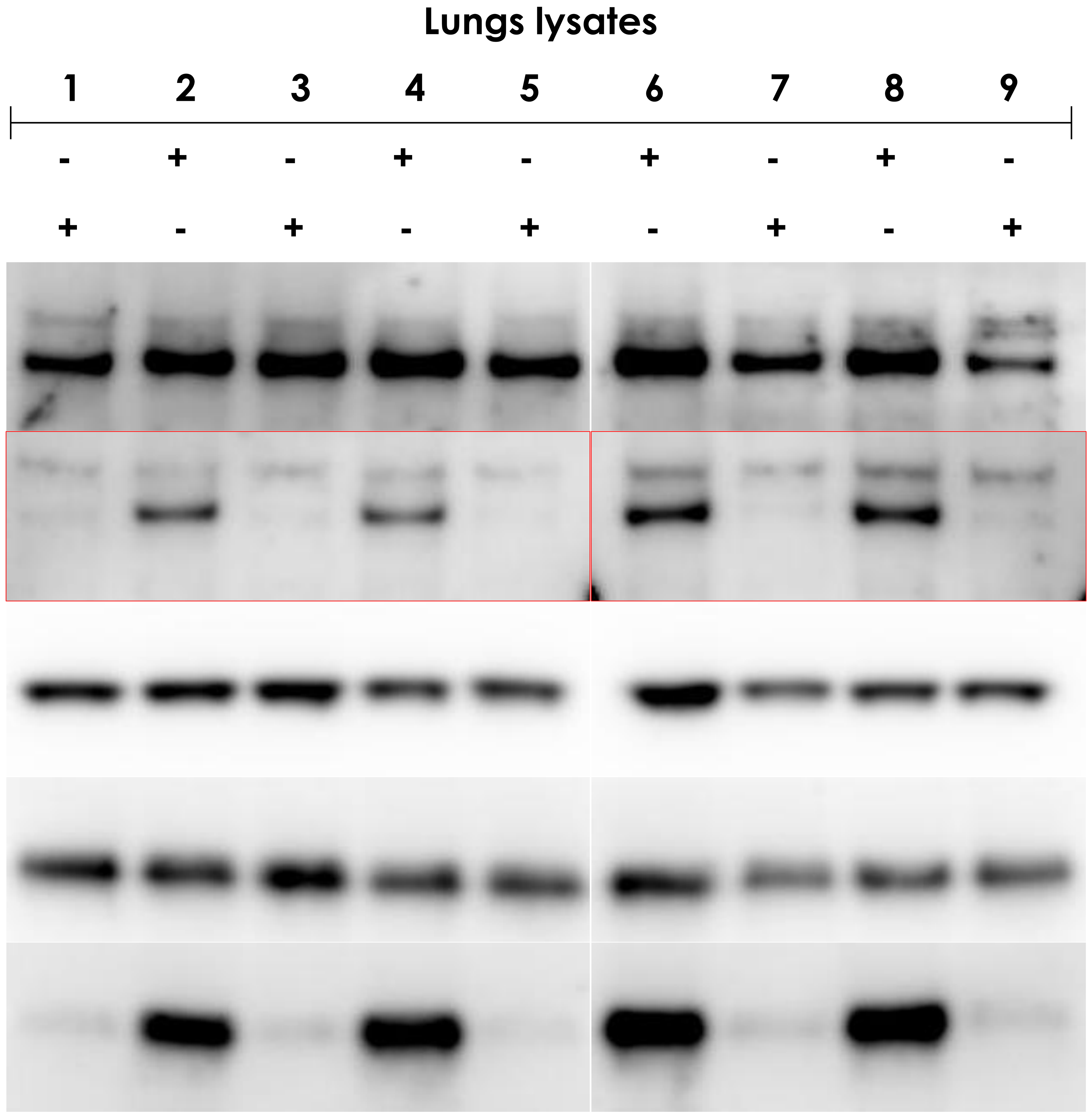

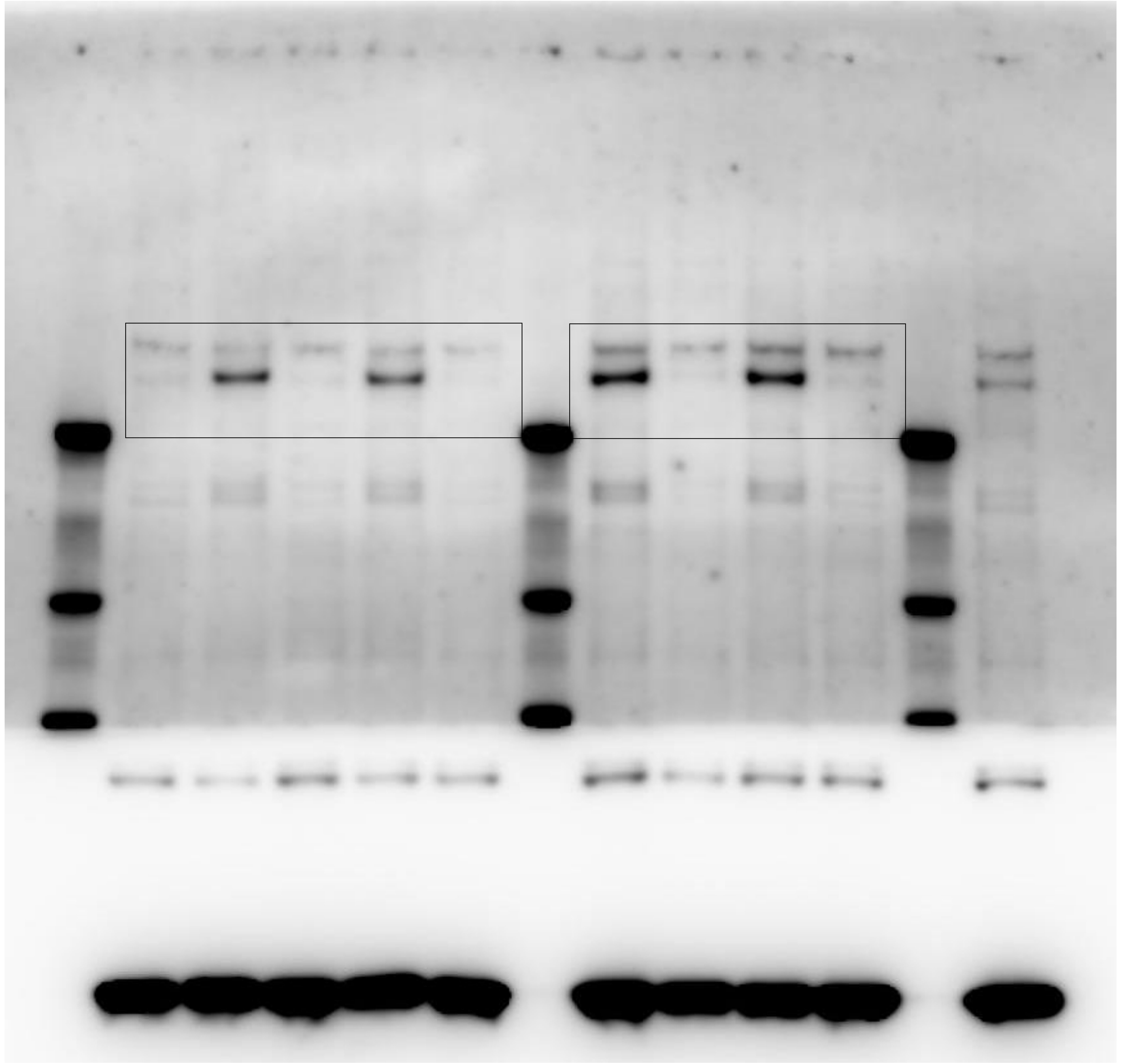

Figure 5a

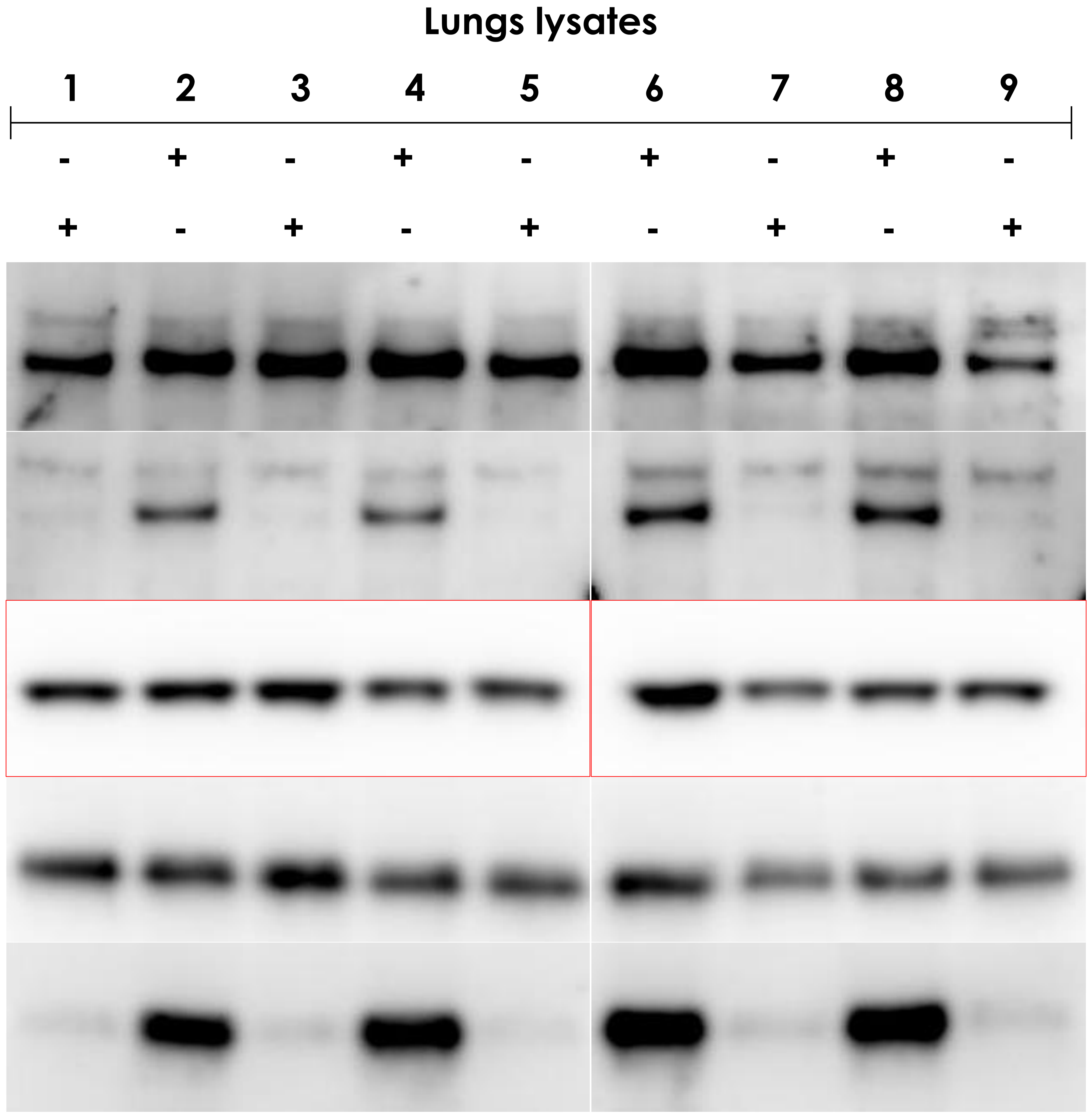

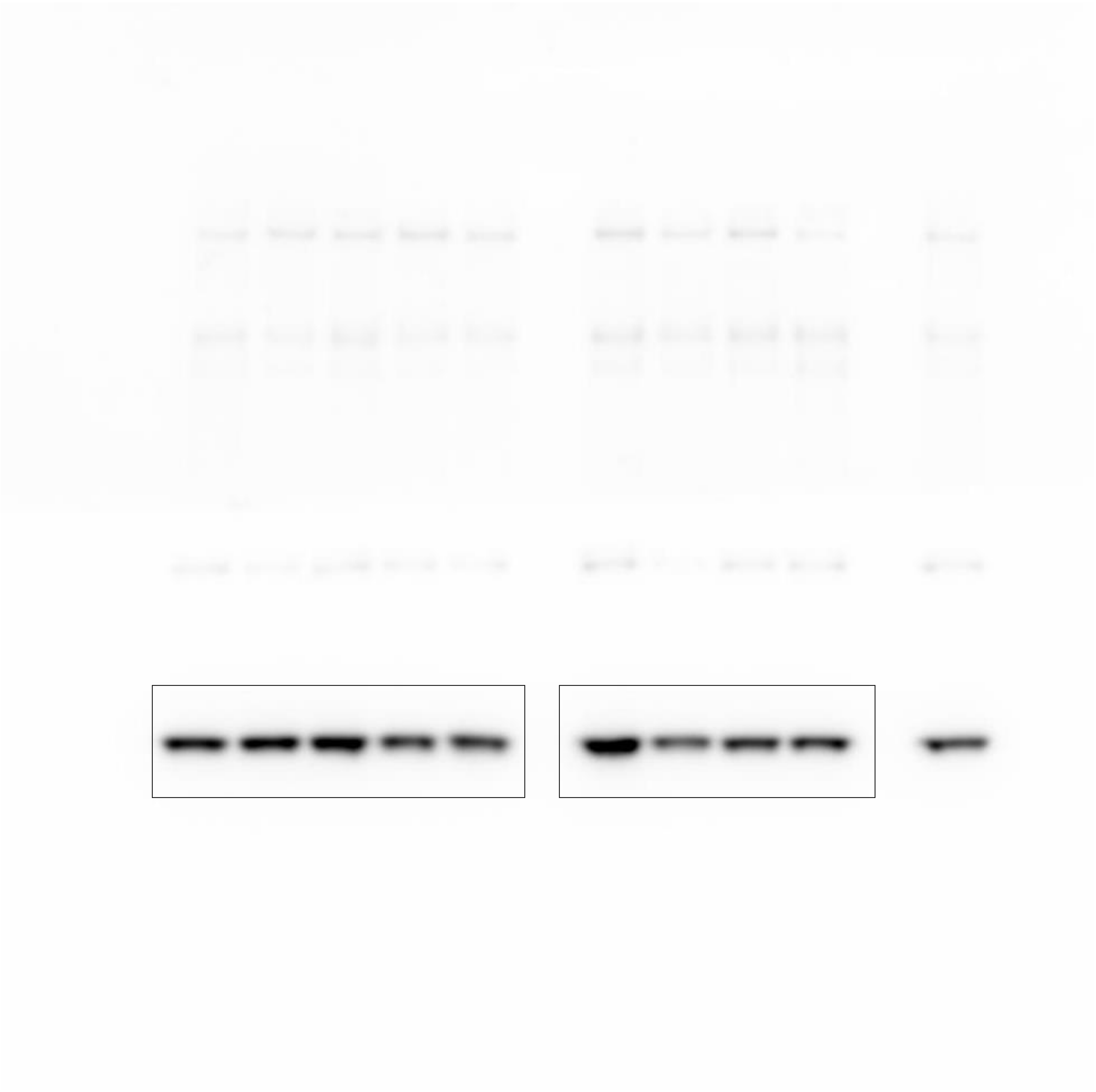

Figure 5a

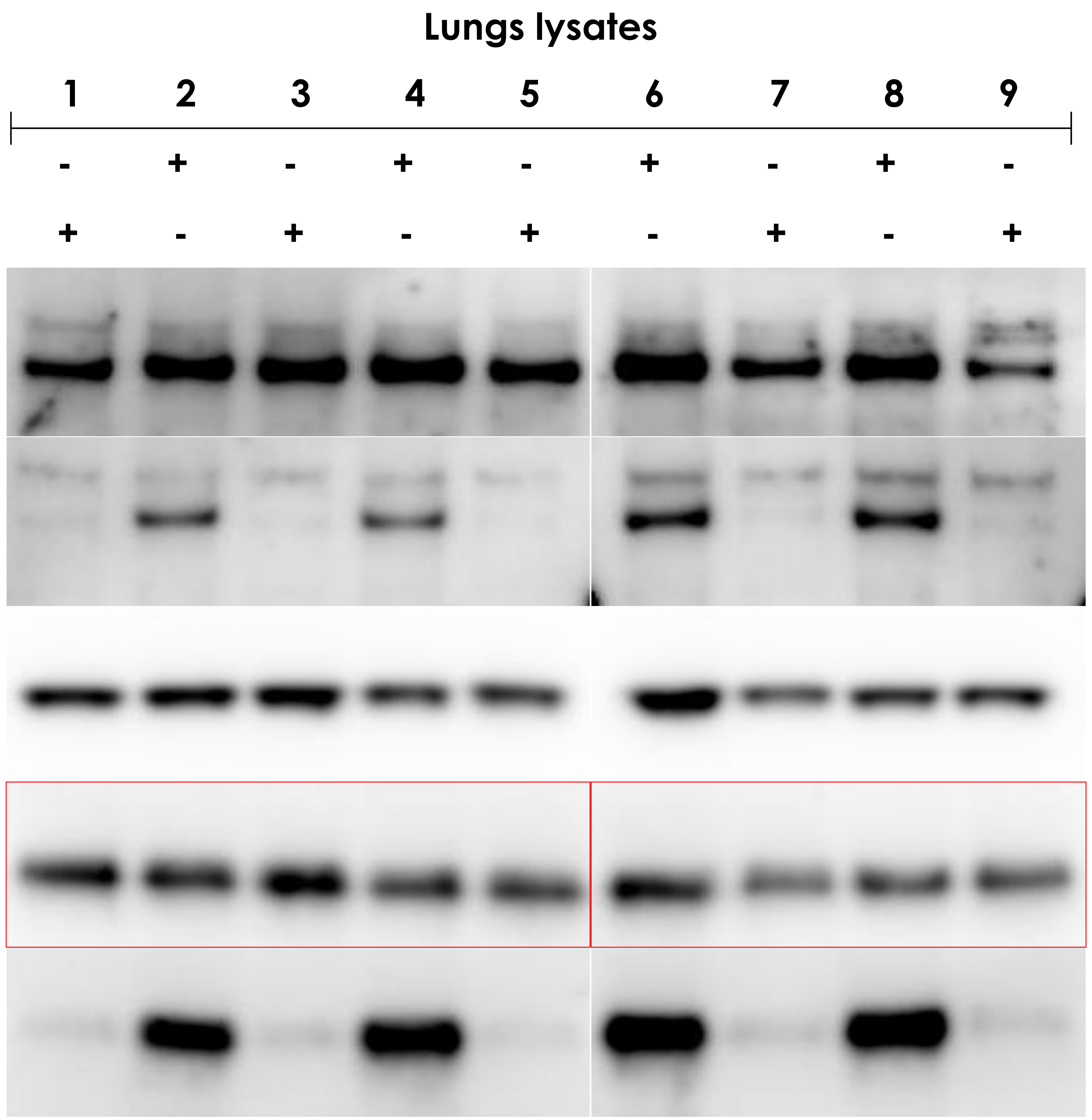

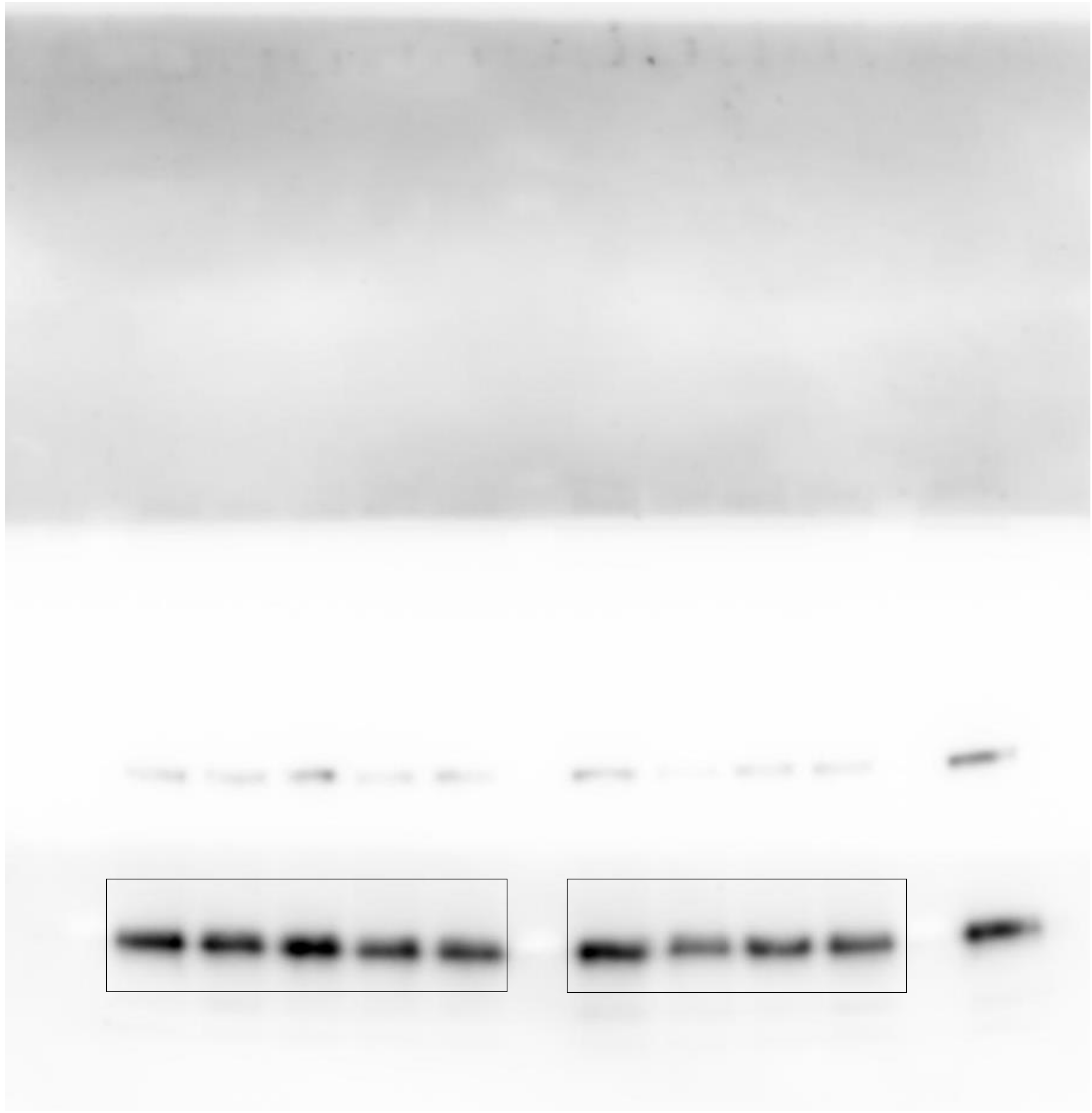

Figure 5a

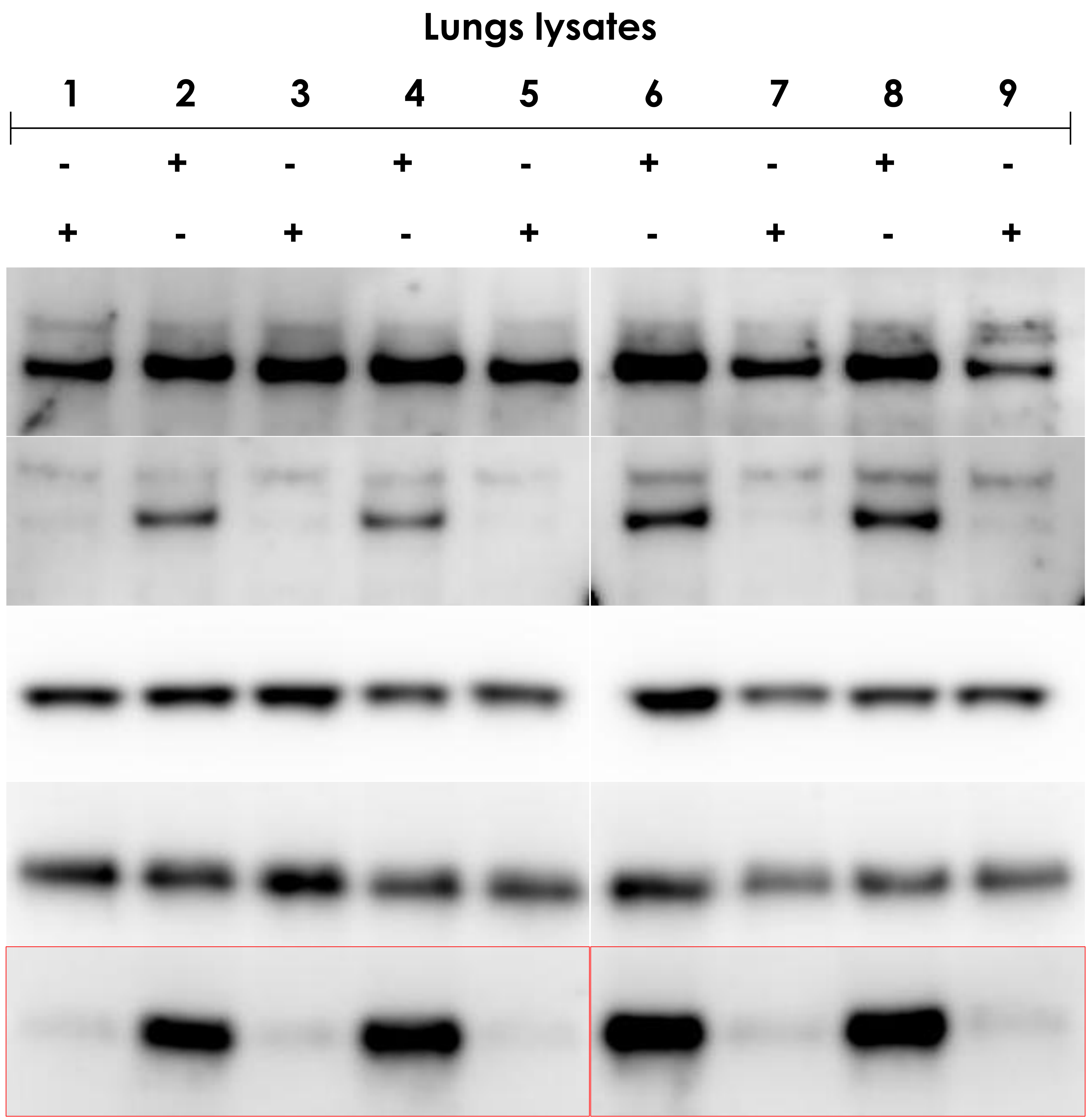

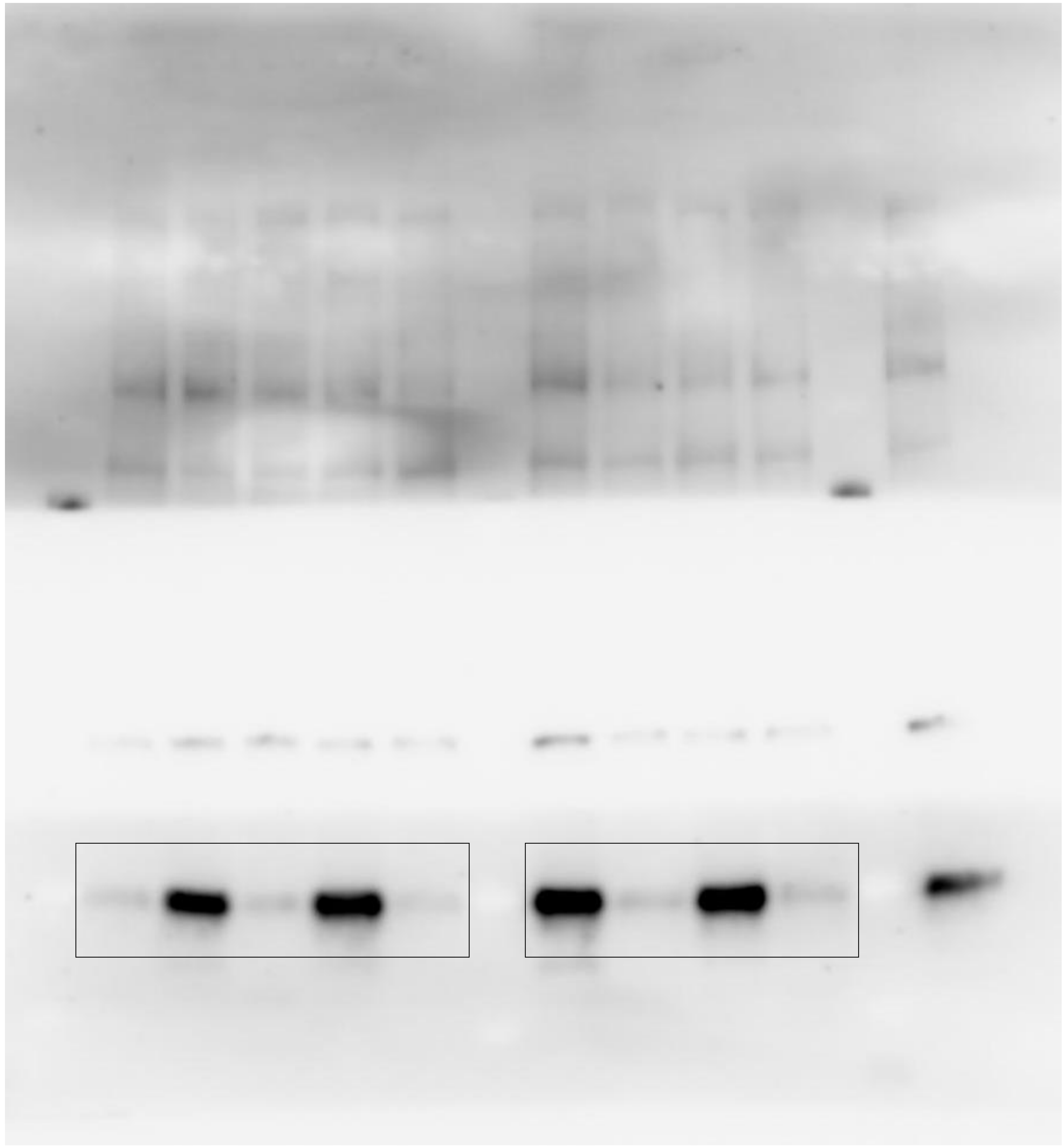

Figure 5b

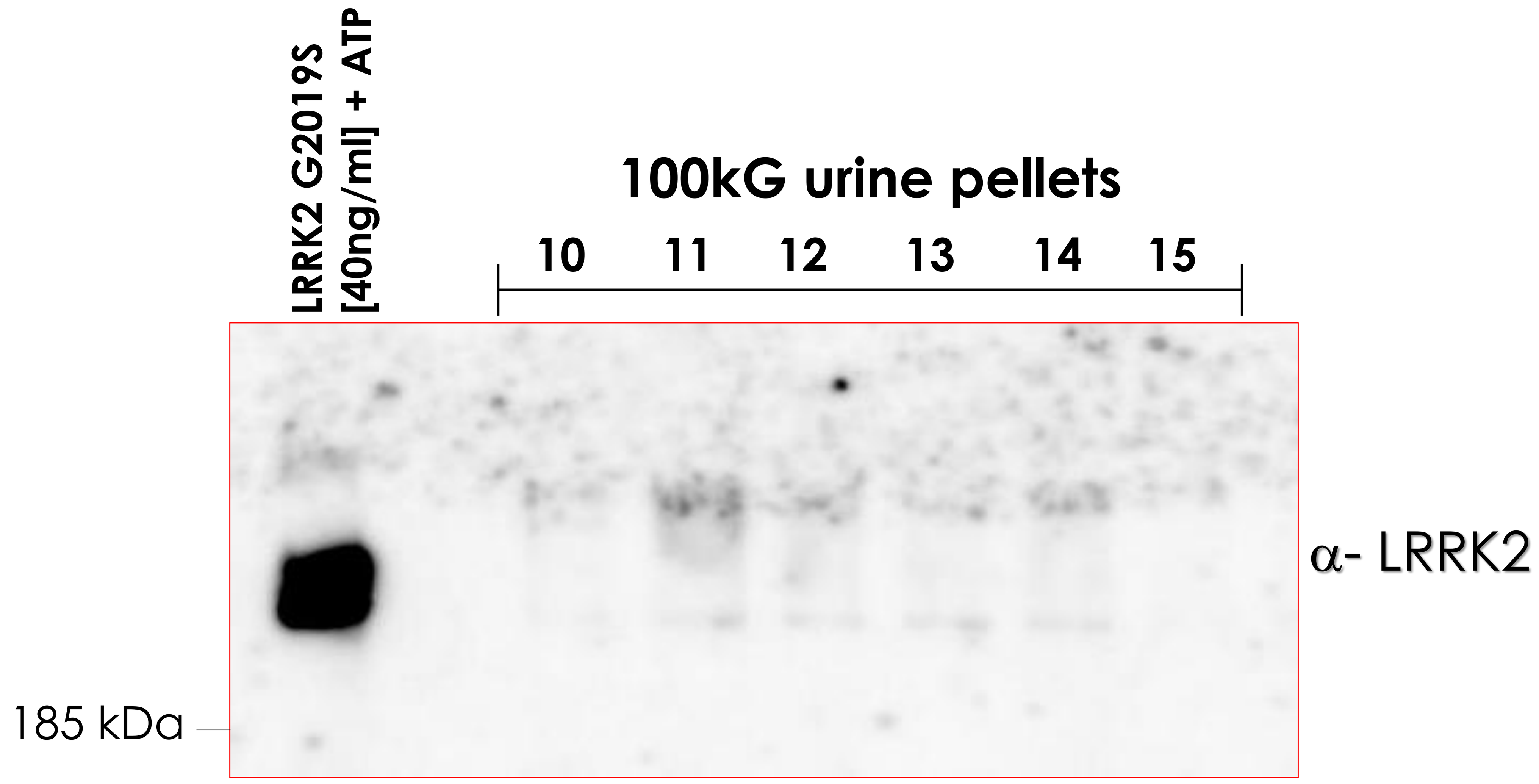
LRRK2 G2019S [40ng/ml] + ATP
100kG urine pellets
10
11
12
13
14
15
α- LRRK2
185 kDa

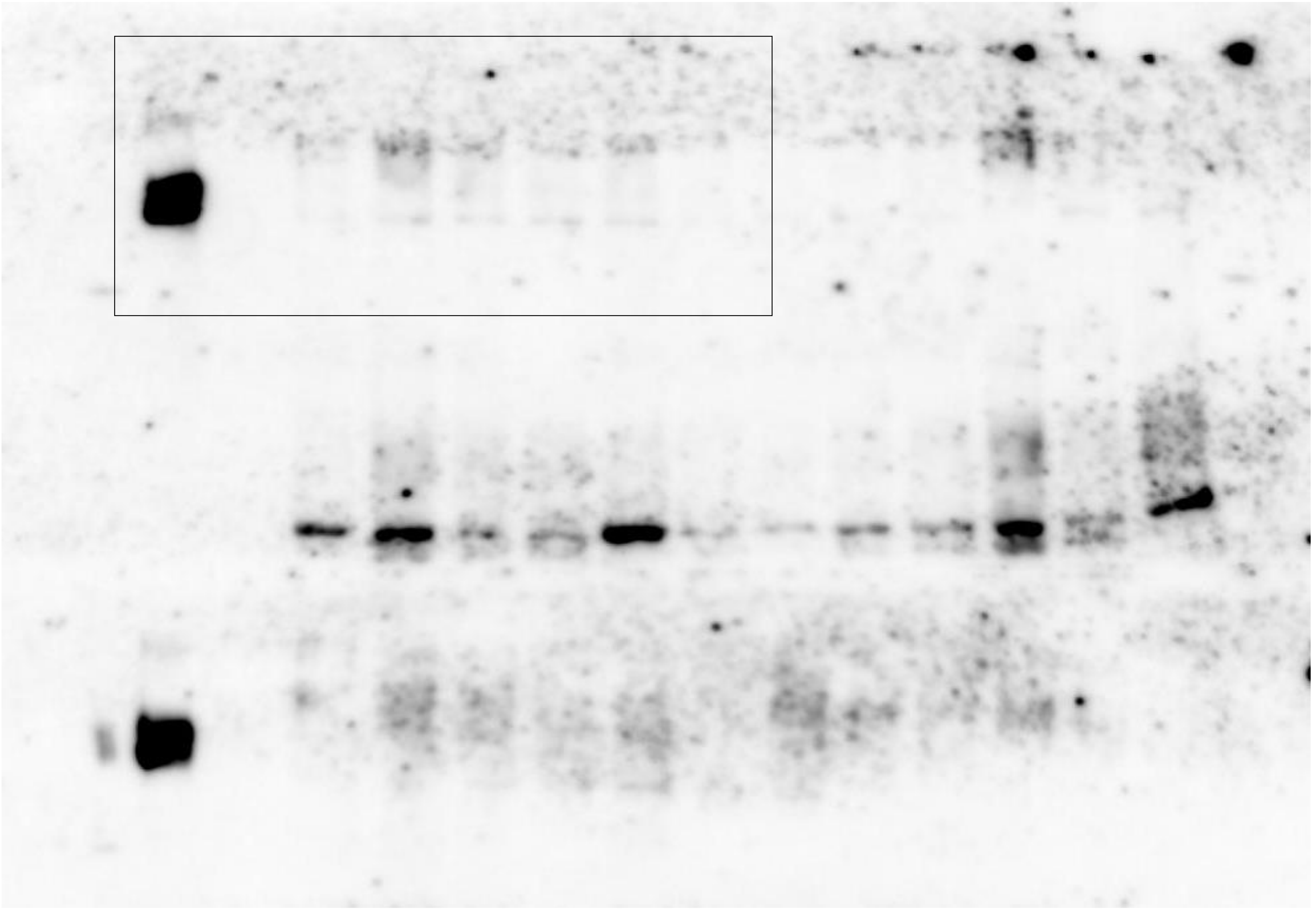

Figure 5c

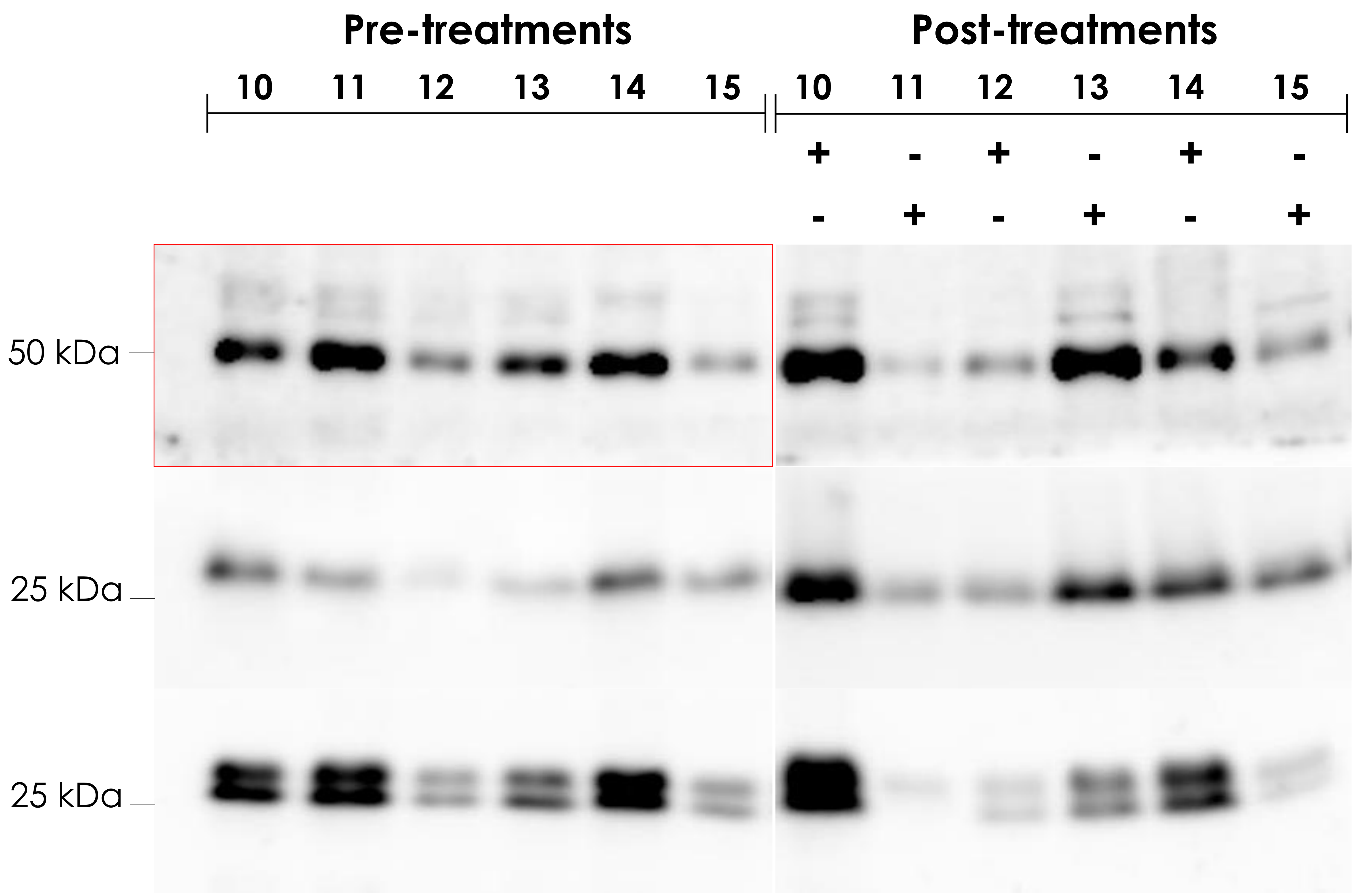

Pre-treatments
Post-treatments
10 11 12 13 14 15
10 11 12 13 14 15
+ - + - + -
- + - + - +
50 kDa
25 kDa
25 kDa

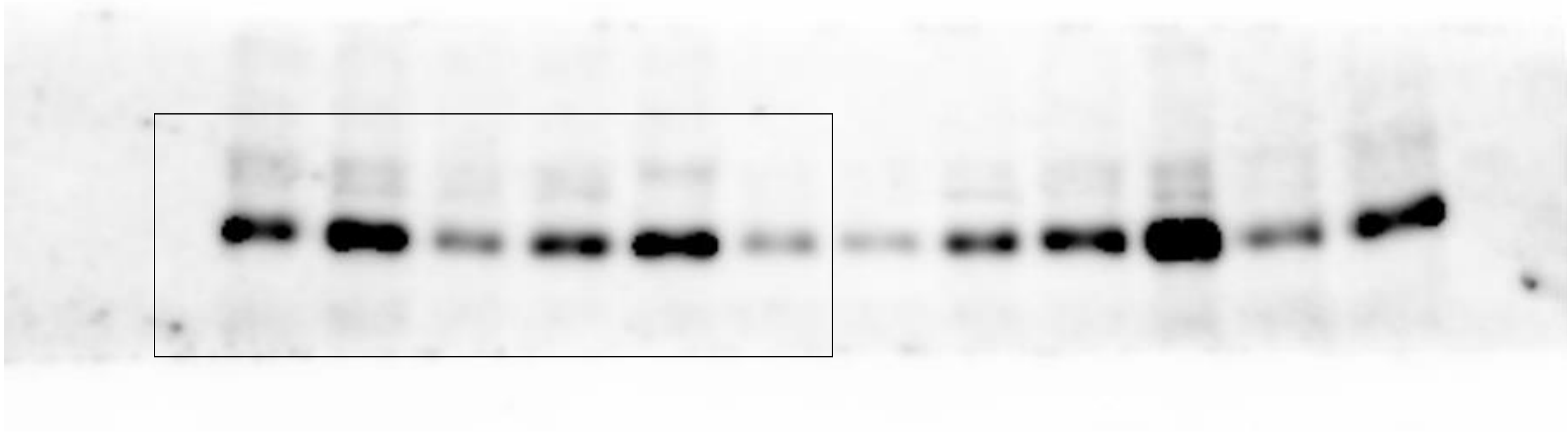

Figure 5c

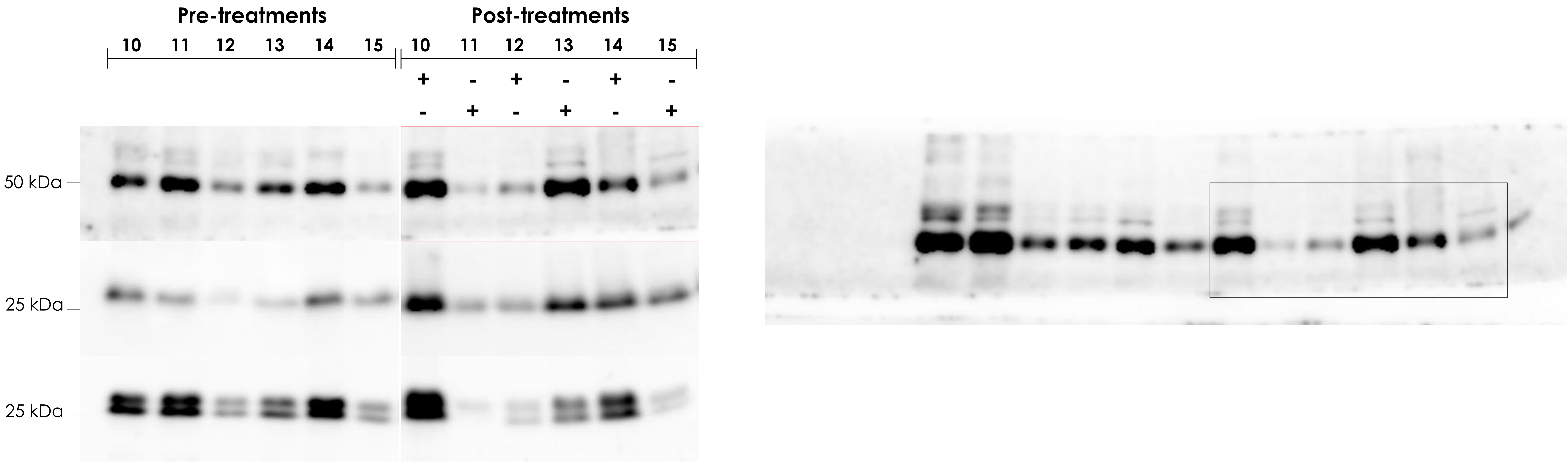
Pre-treatments
Post-treatments
10 11 12 13 14 15
10 11 12 13 14 15
+ - + - + -
- + - + - +
50 kDa
25 kDa
25 kDa

Figure 5c

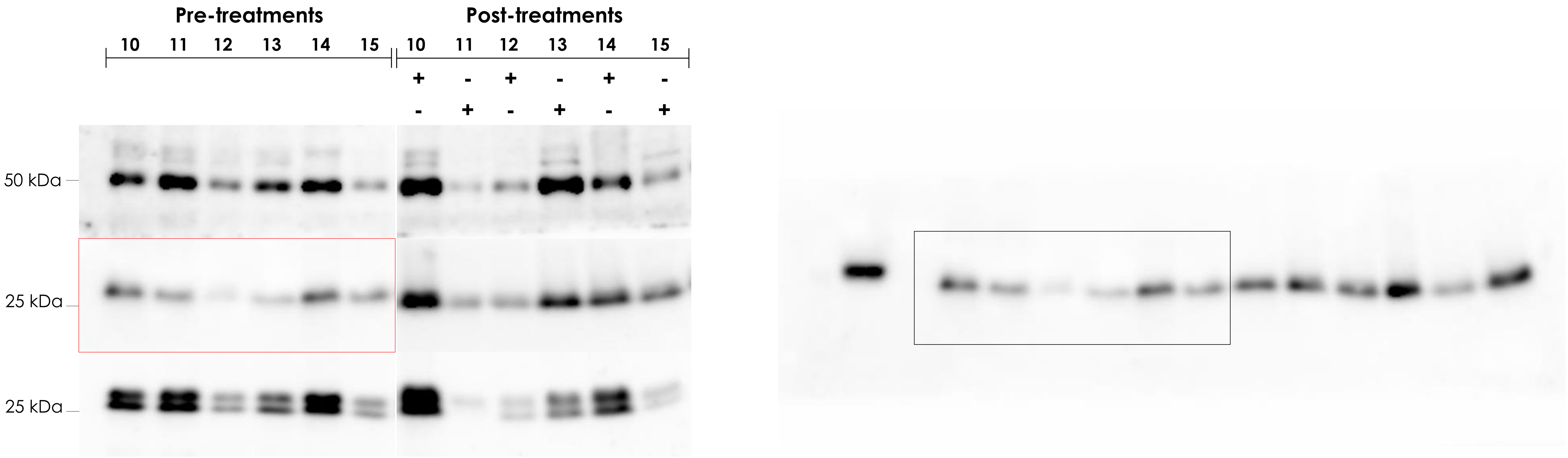

Pre-treatments
Post-treatments
10 11 12 13 14 15
10 11 12 13 14 15
+ - + - + -
- + - + - +
50 kDa
25 kDa
25 kDa

Figure 5c

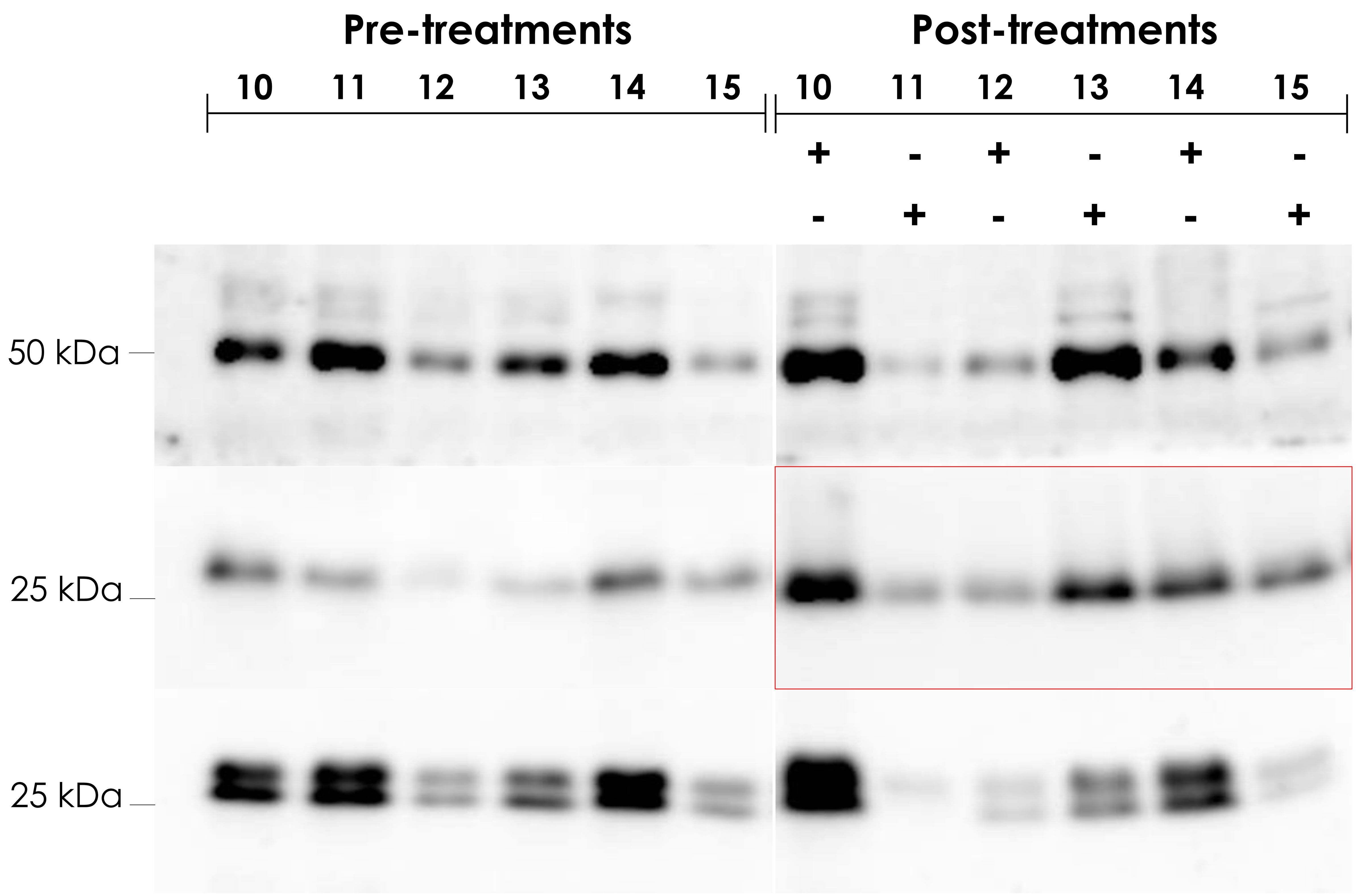
Pre-treatments
Post-treatments
10
11
12
13
14
15
10
11
12
13
14
15
+
-
+
-
+
-
-
+
-
+
-
+
50 kDa
25 kDa
25 kDa

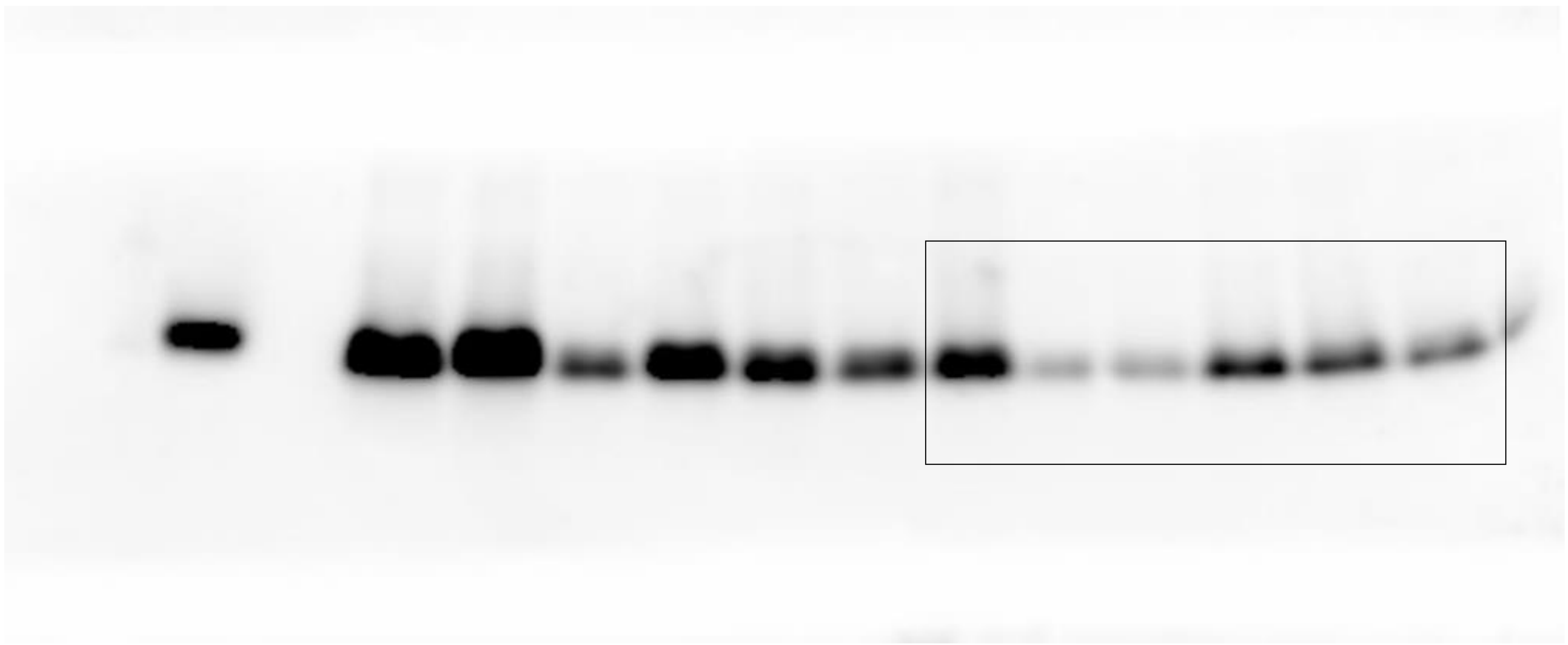

Figure 5c

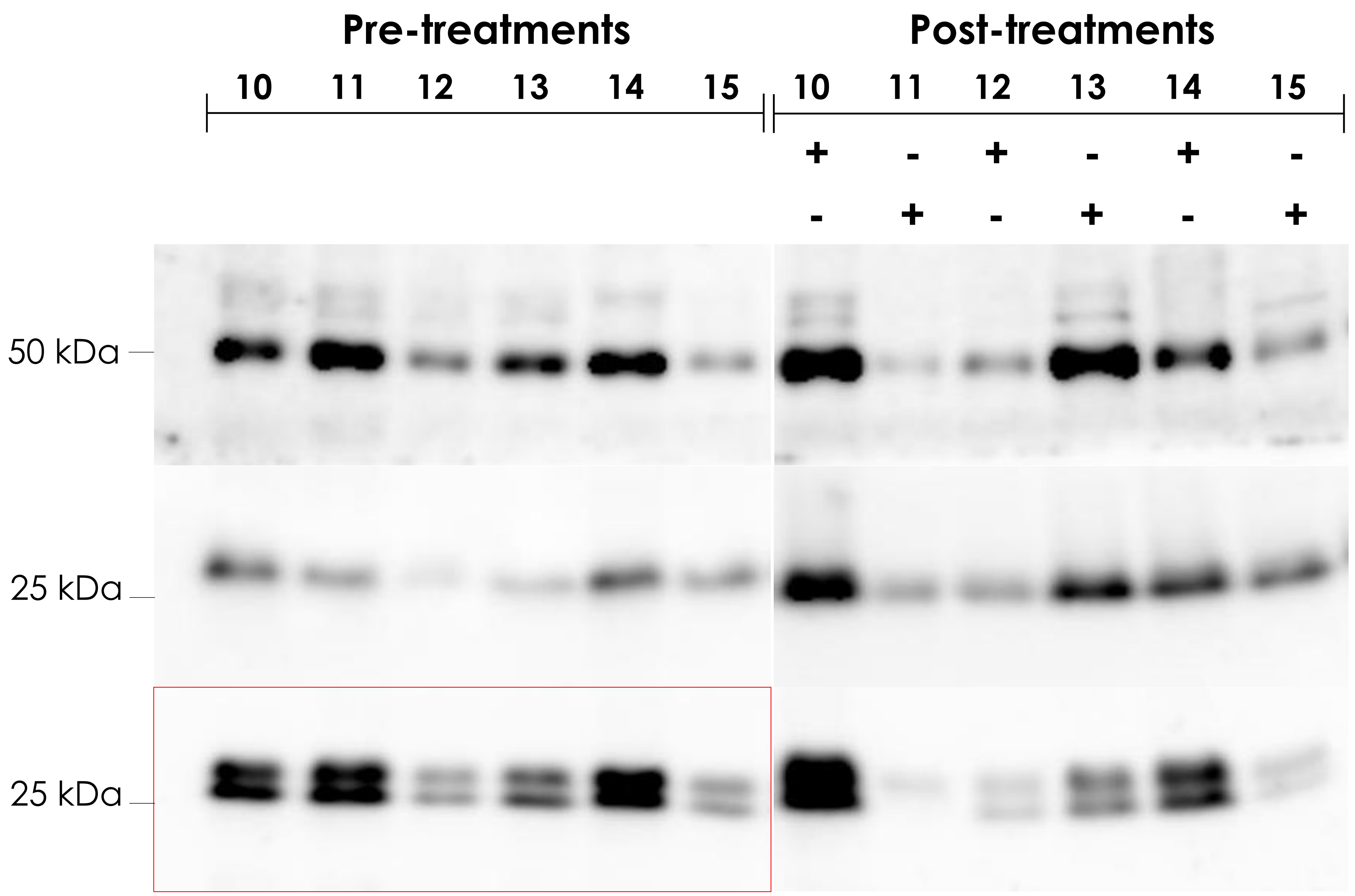

Pre-treatments
Post-treatments
10 11 12 13 14 15
10 11 12 13 14 15
+ - + - + -
- + - + - +
50 kDa
25 kDa
25 kDa

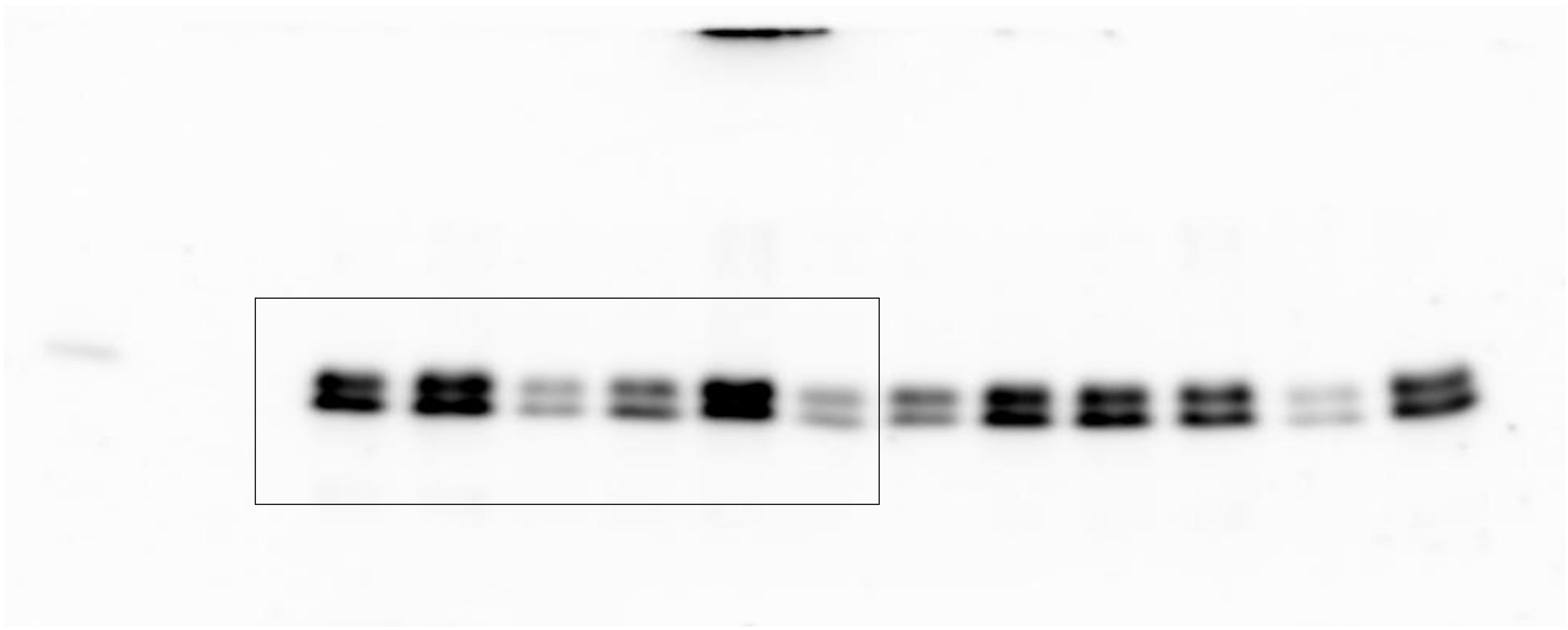

Figure 5c

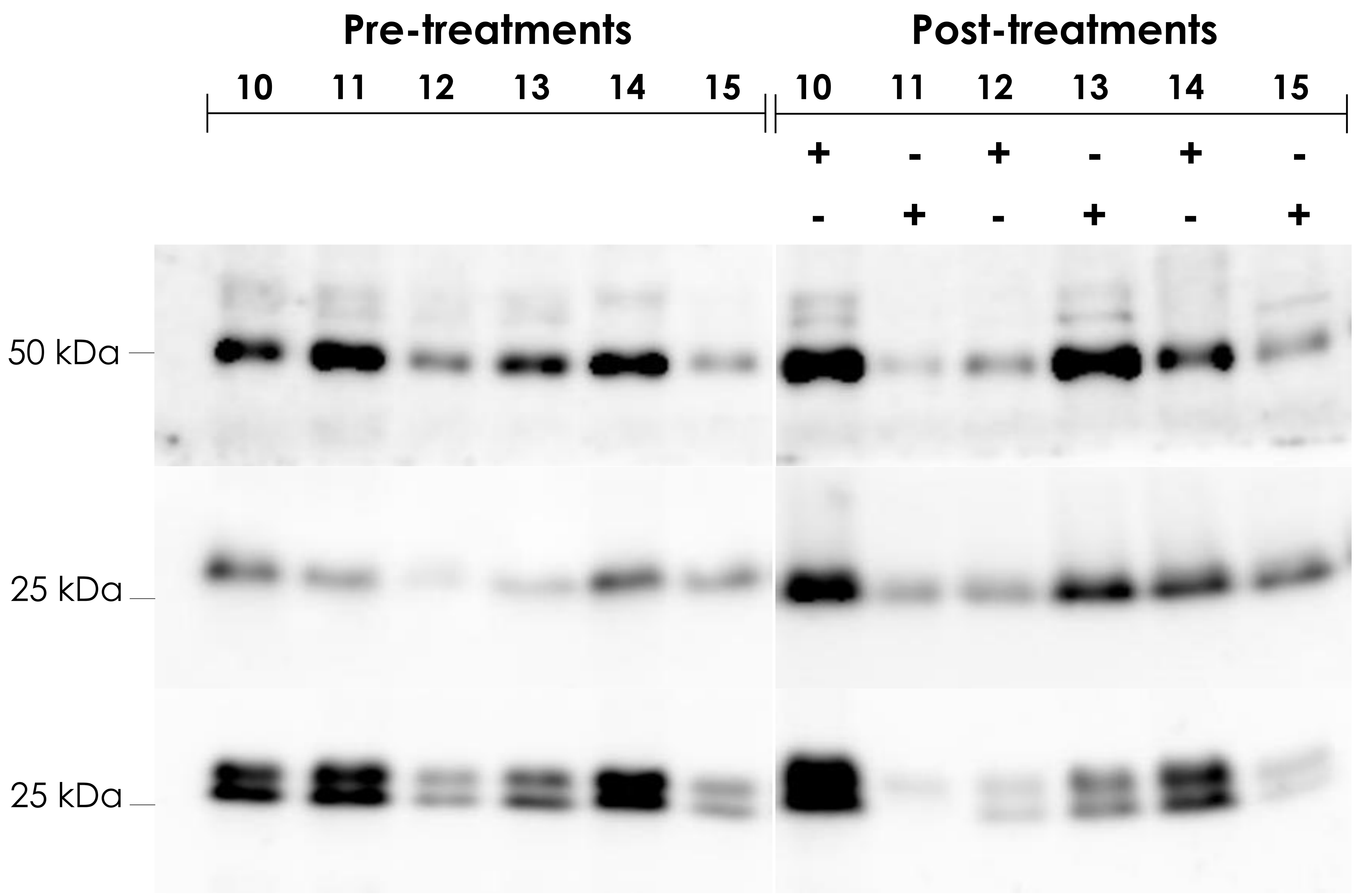

Pre-treatments
Post-treatments
10
11
12
13
14
15
10
11
12
13
14
15
+
-
+
-
+
-
-
+
-
+
-
+
50 kDa
25 kDa
25 kDa

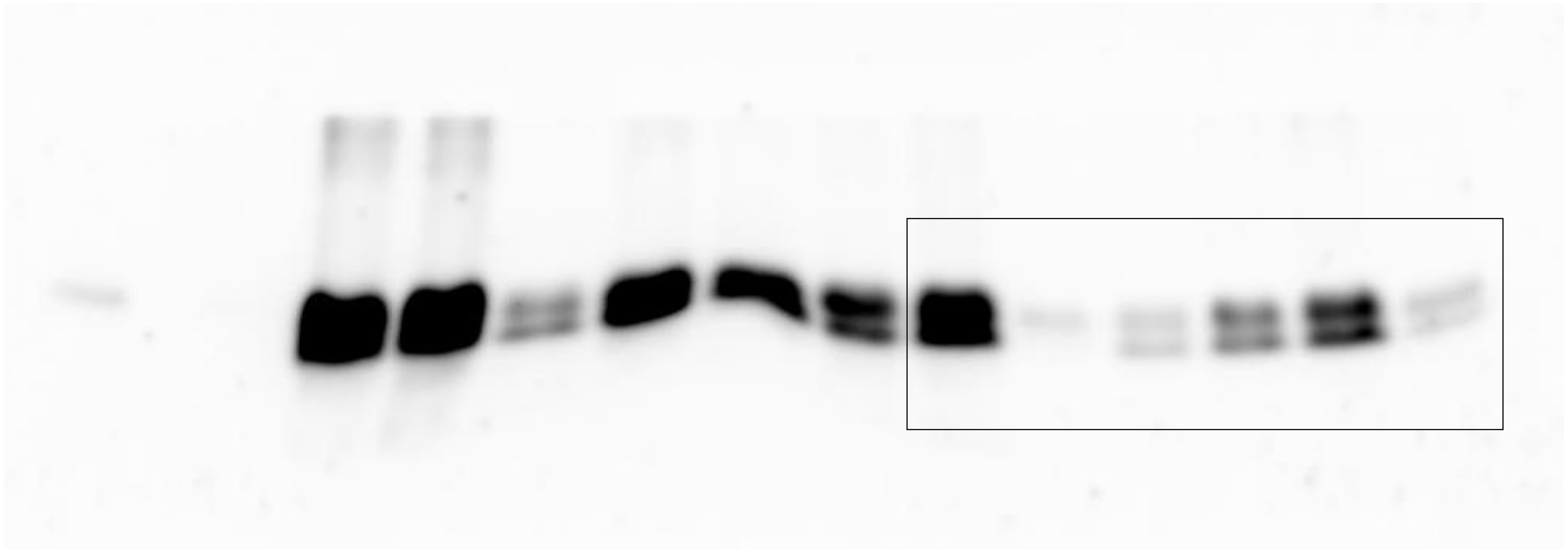

Figure 6a

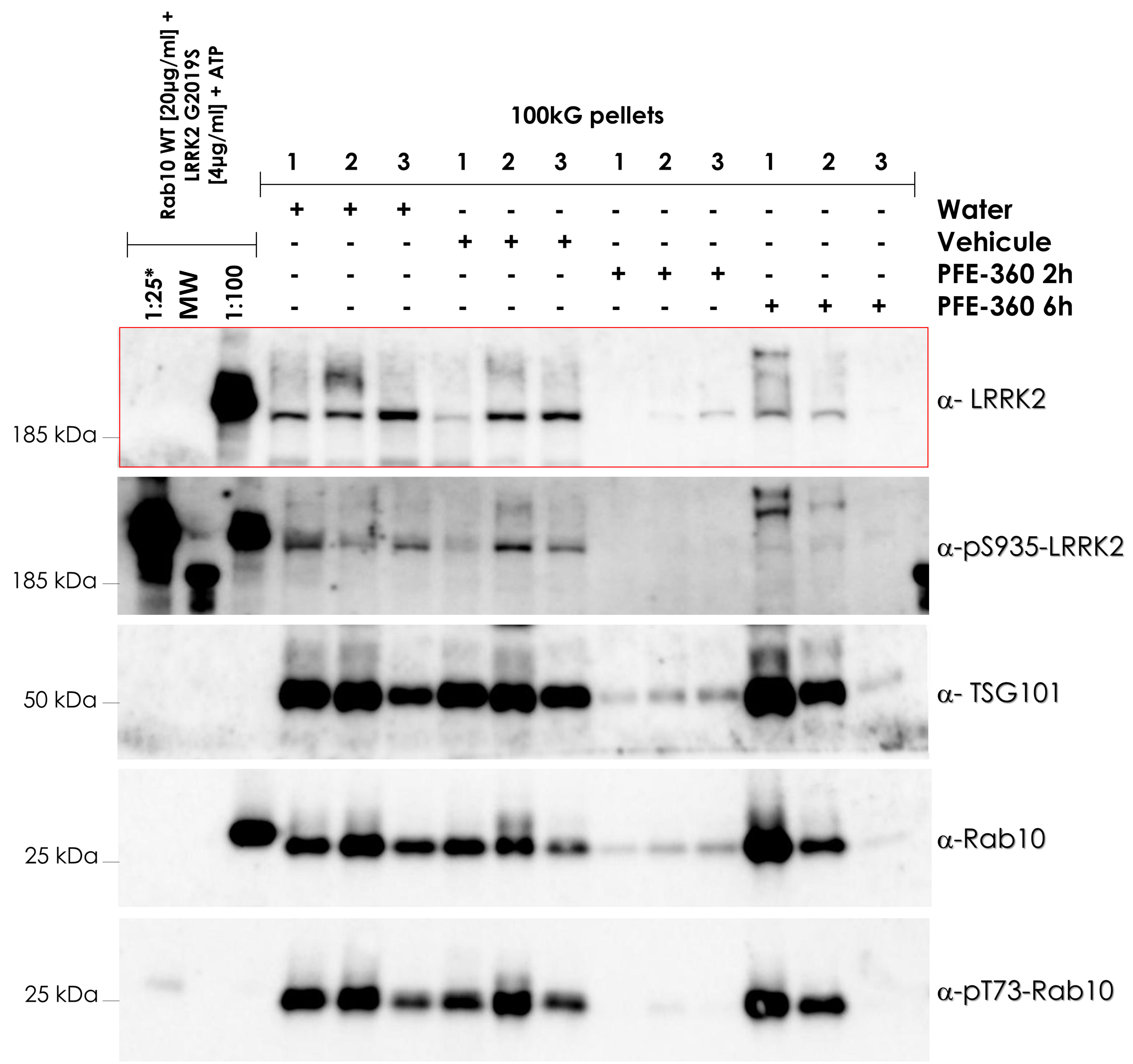

*Only loaded for α-pS935-LRRK2 and α-pT73-Rab10 antibodies

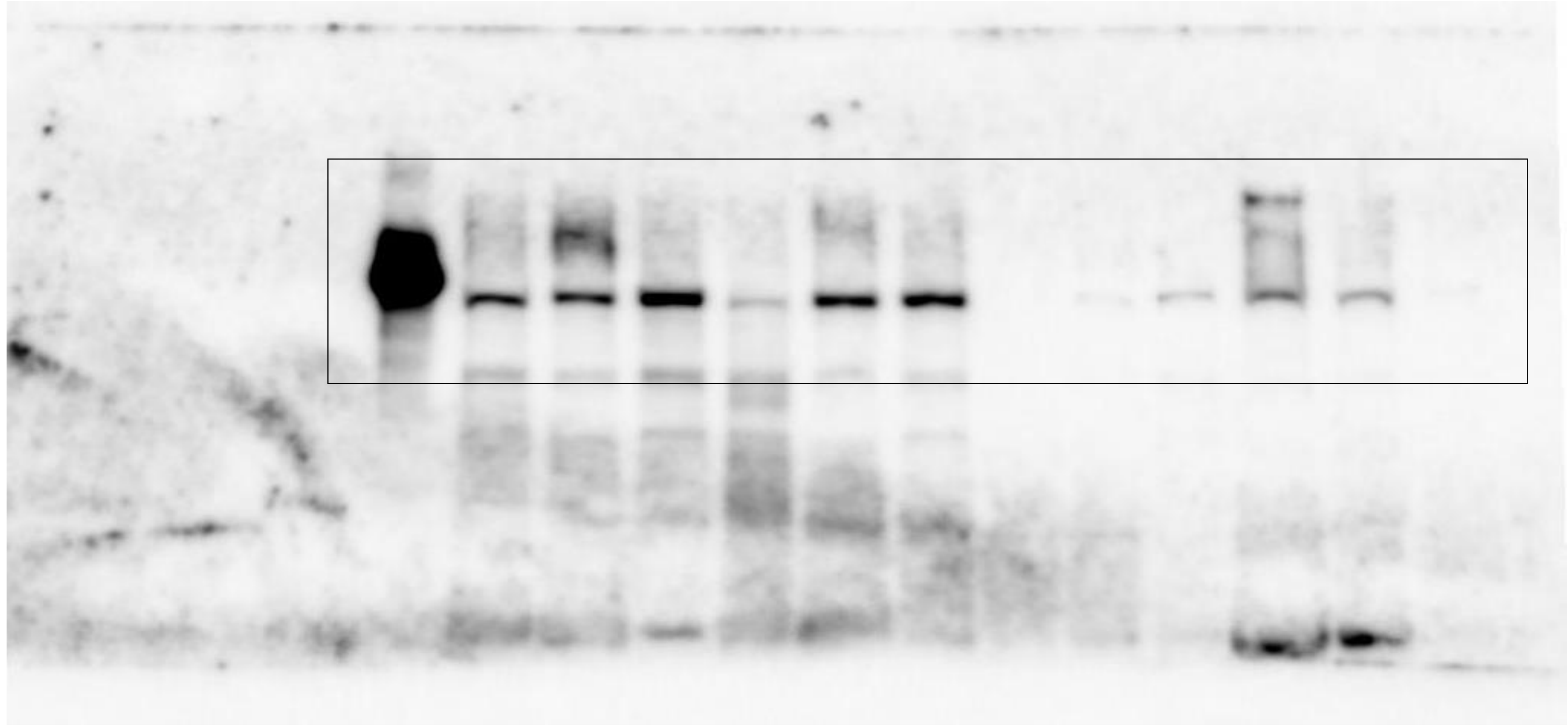

Figure 6a

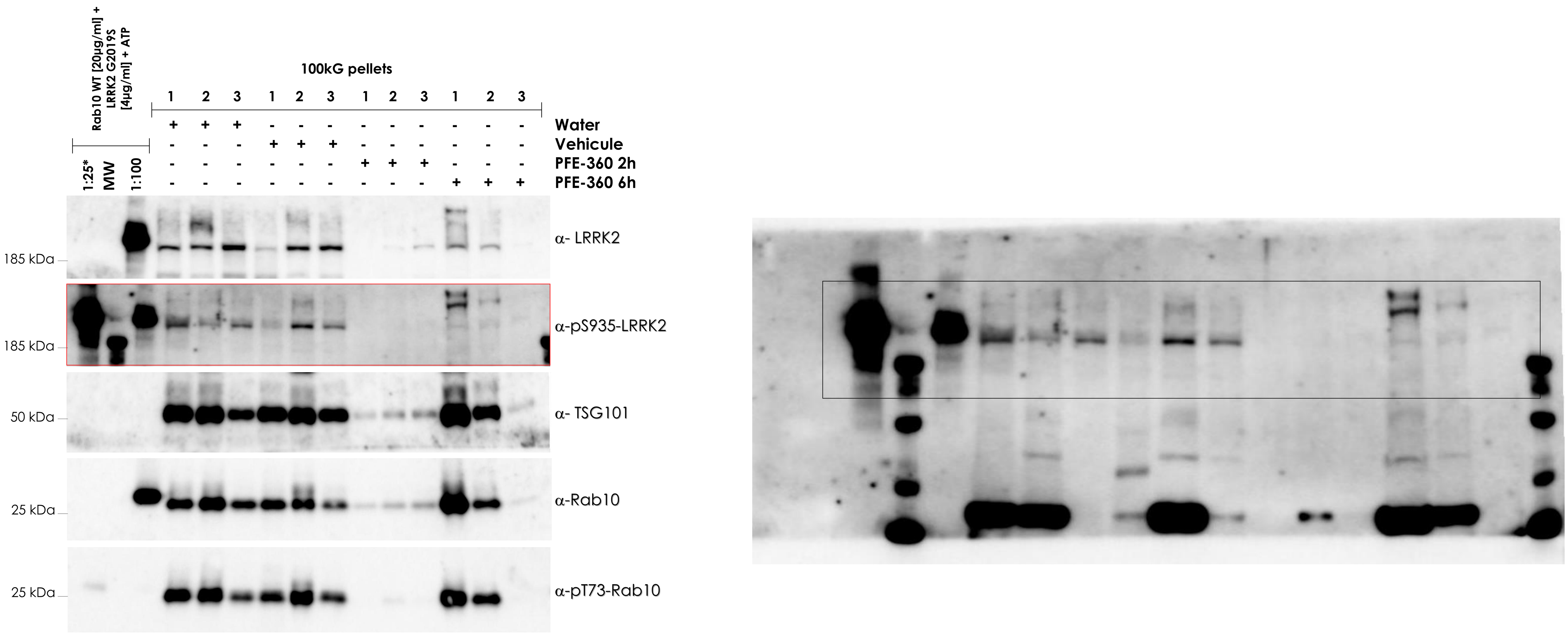

*Only loaded for α-pS935-LRRK2 and α-pT73-Rab10 antibodies

Figure 6a

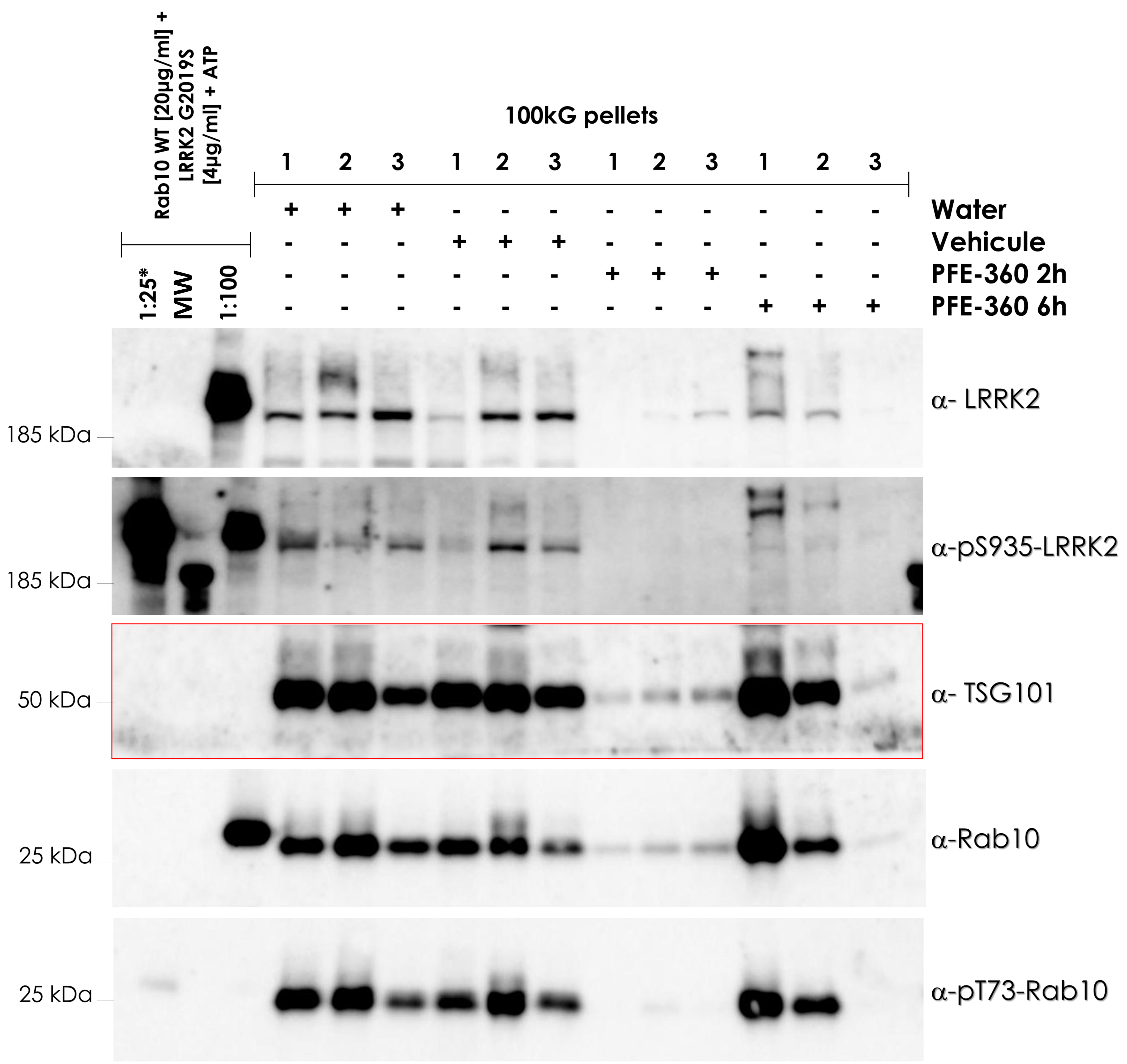

*Only loaded for α-pS935-LRRK2 and α-pT73-Rab10 antibodies

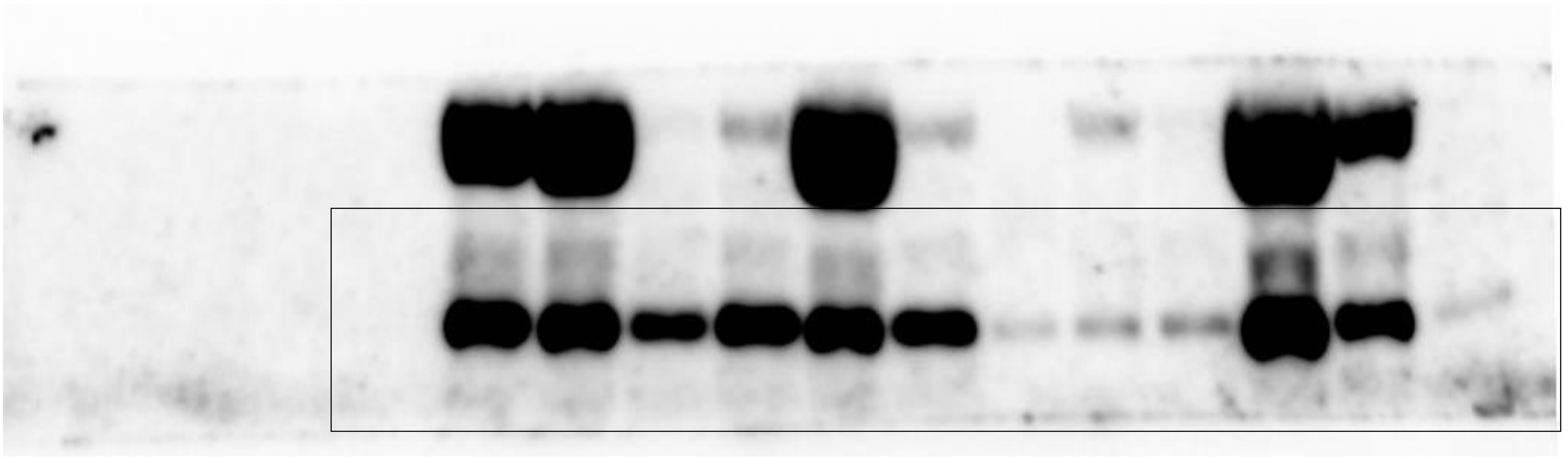

Figure 6a

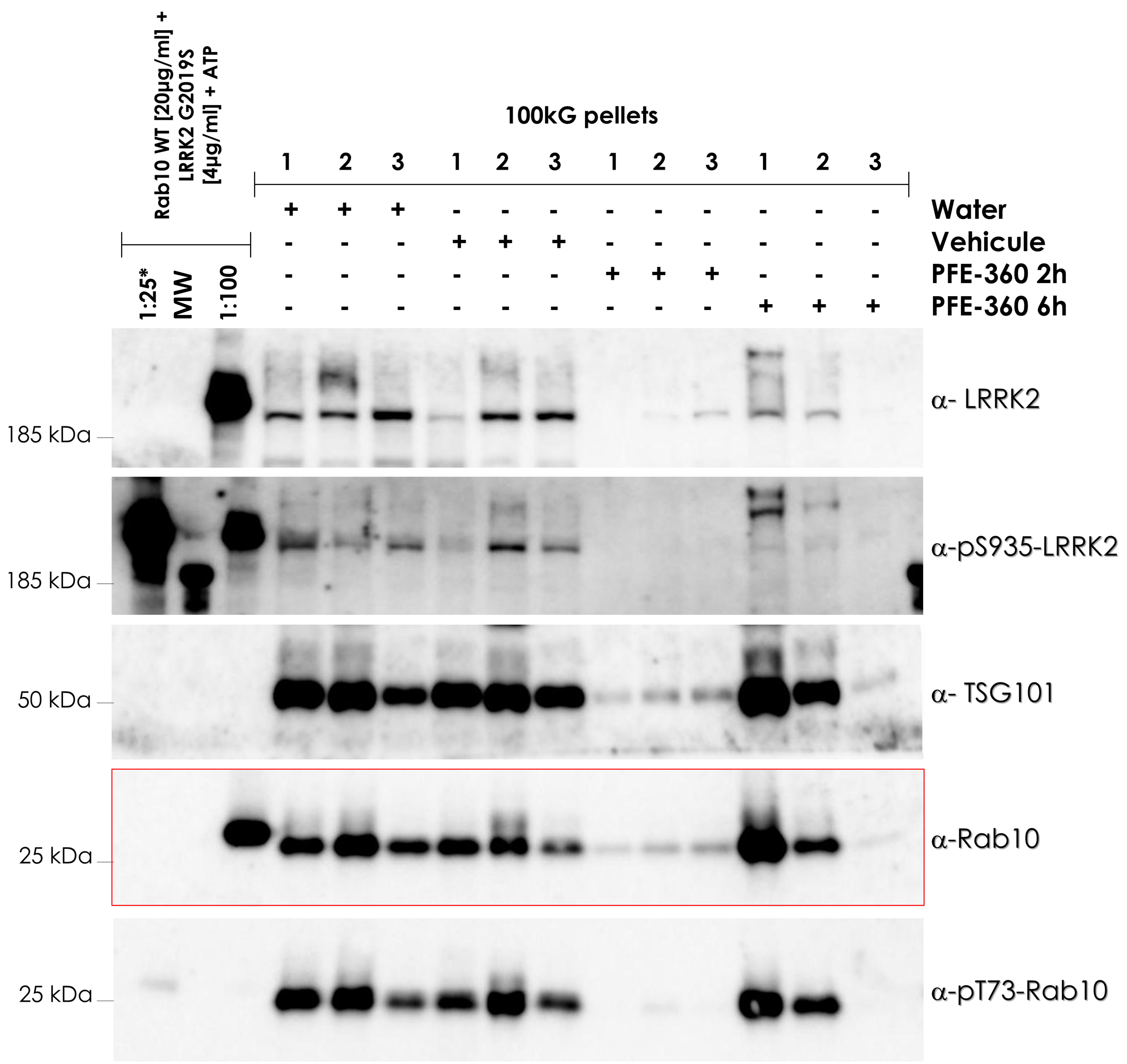

*Only loaded for α-pS935-LRRK2 and α-pT73-Rab10 antibodies

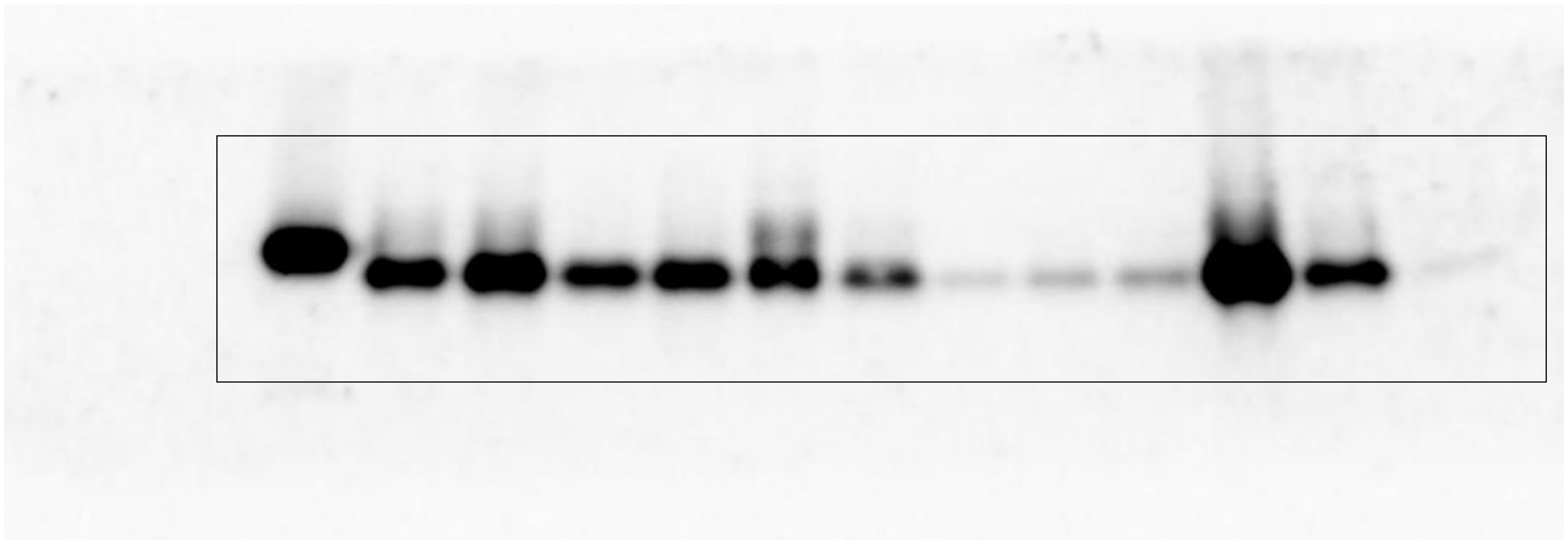

Figure 6a

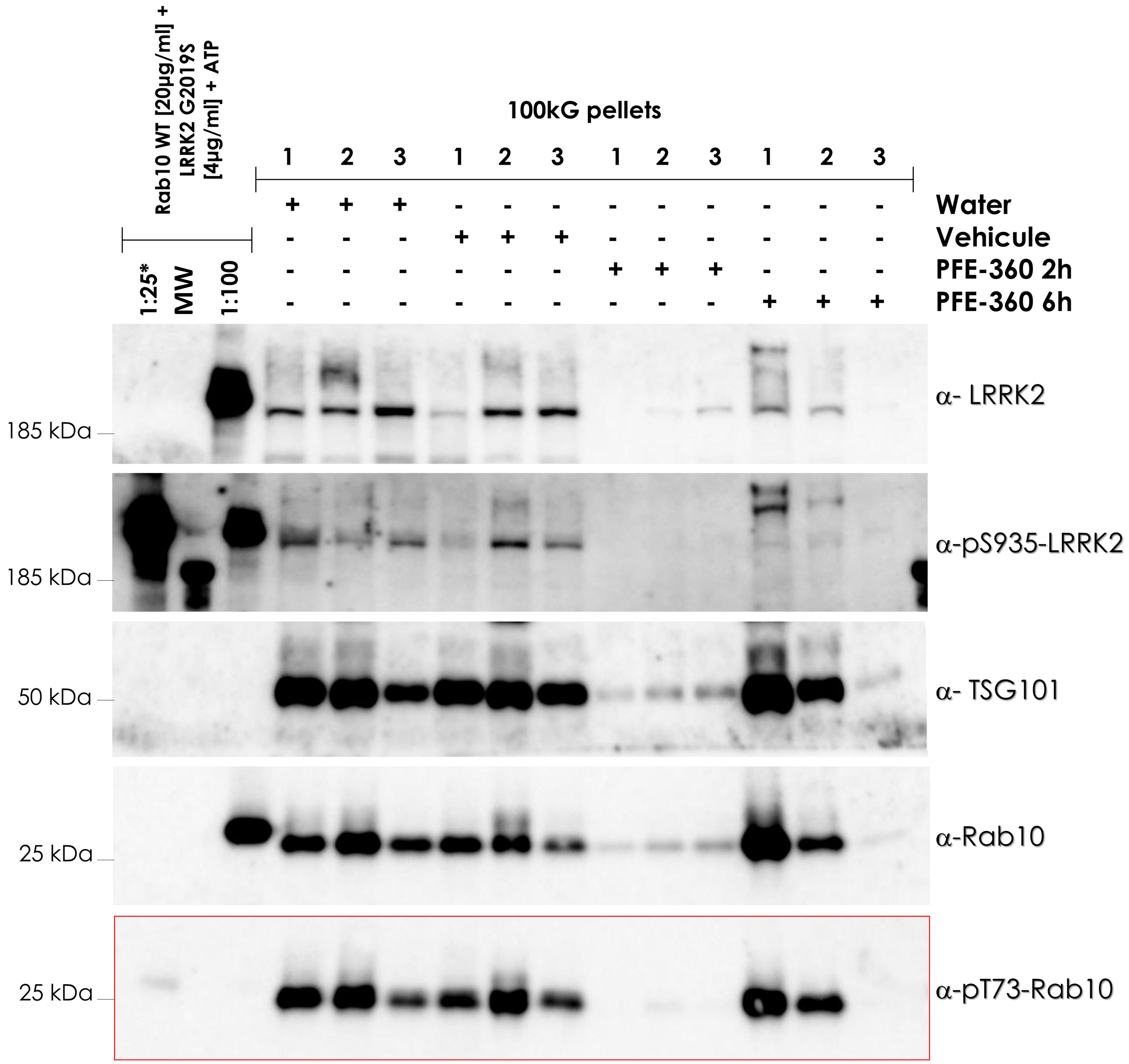

*Only loaded for α-pS935-LRRK2 and α-pT73-Rab10 antibodies

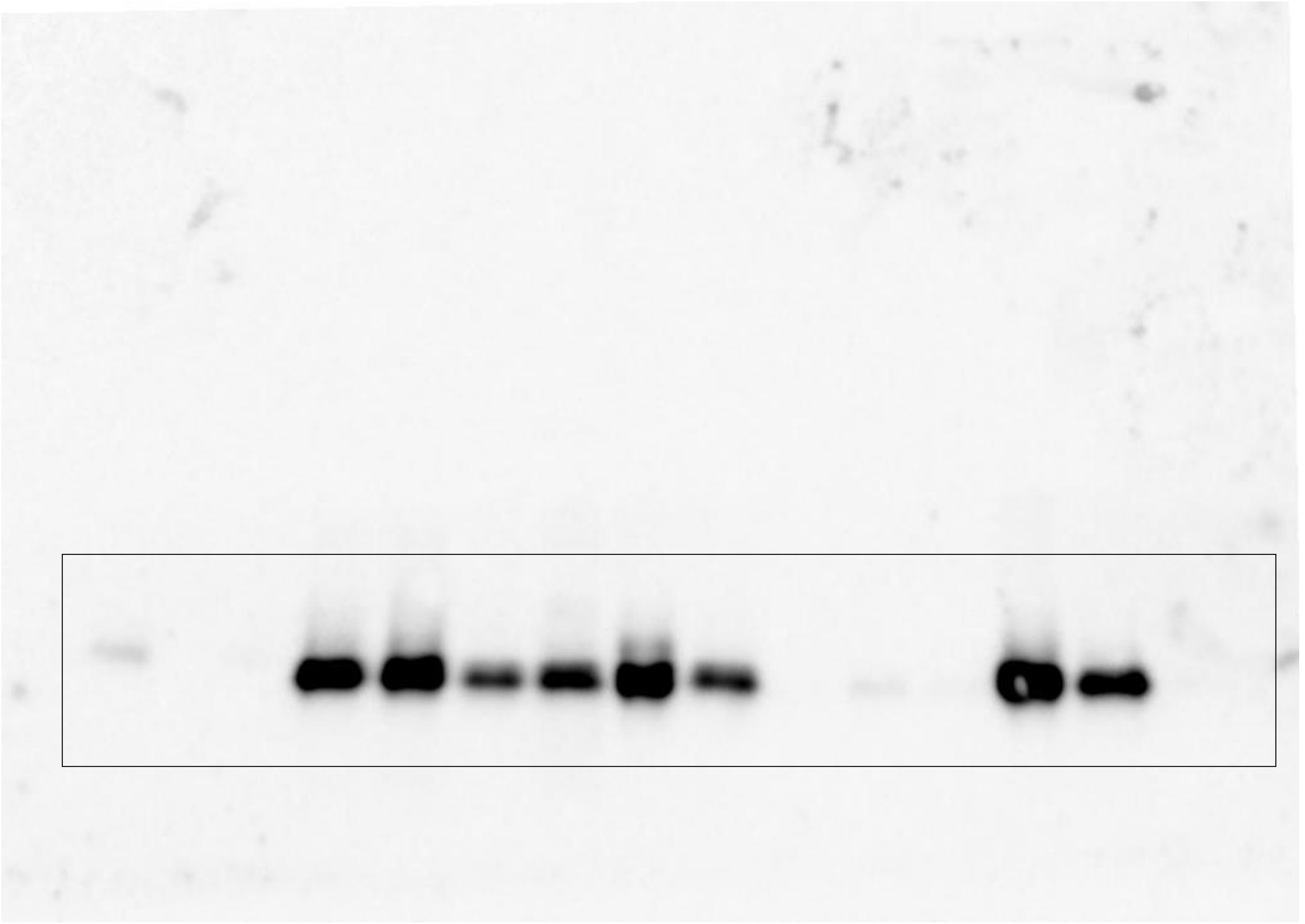

Suppl Figure 1b

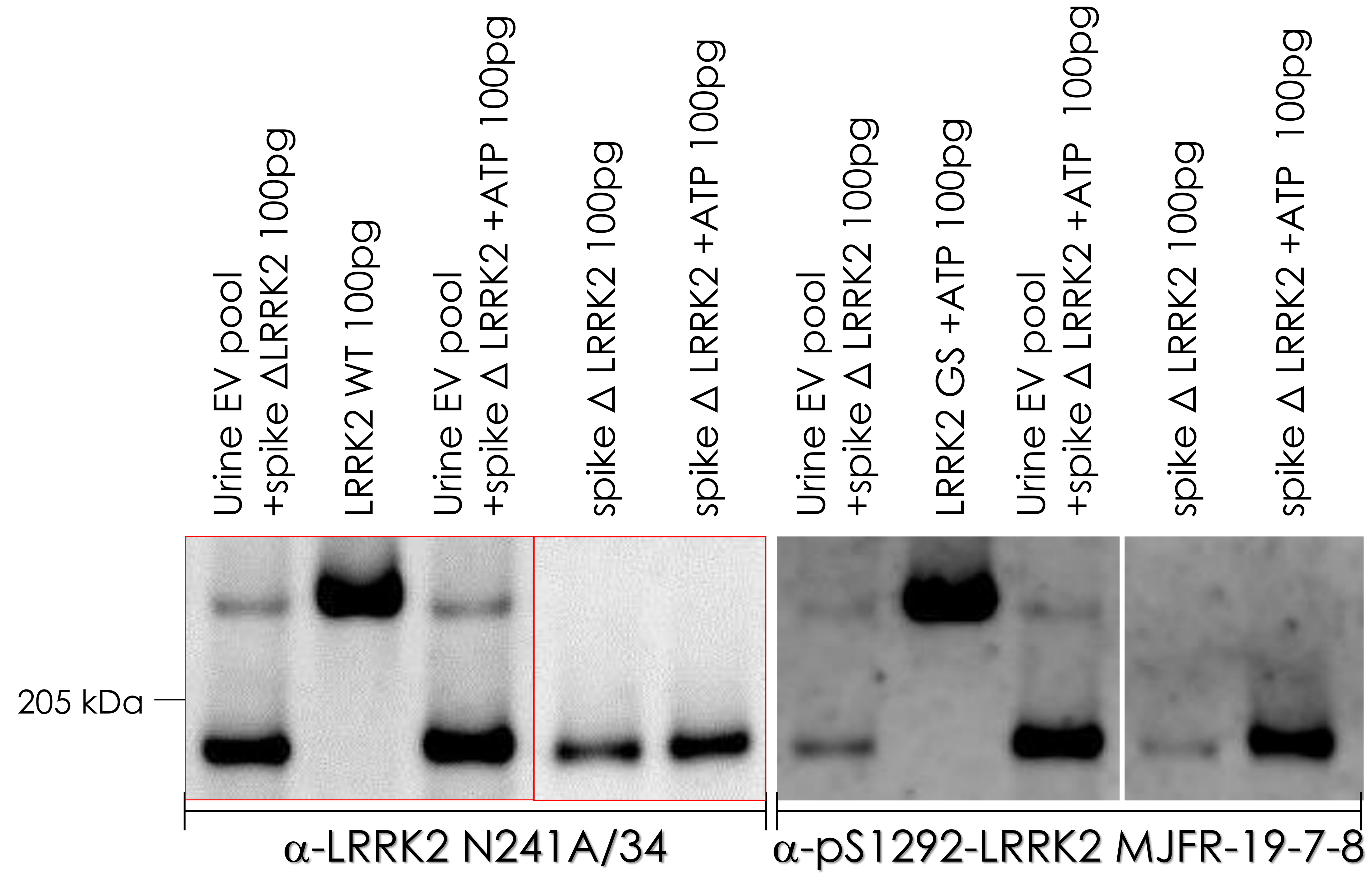

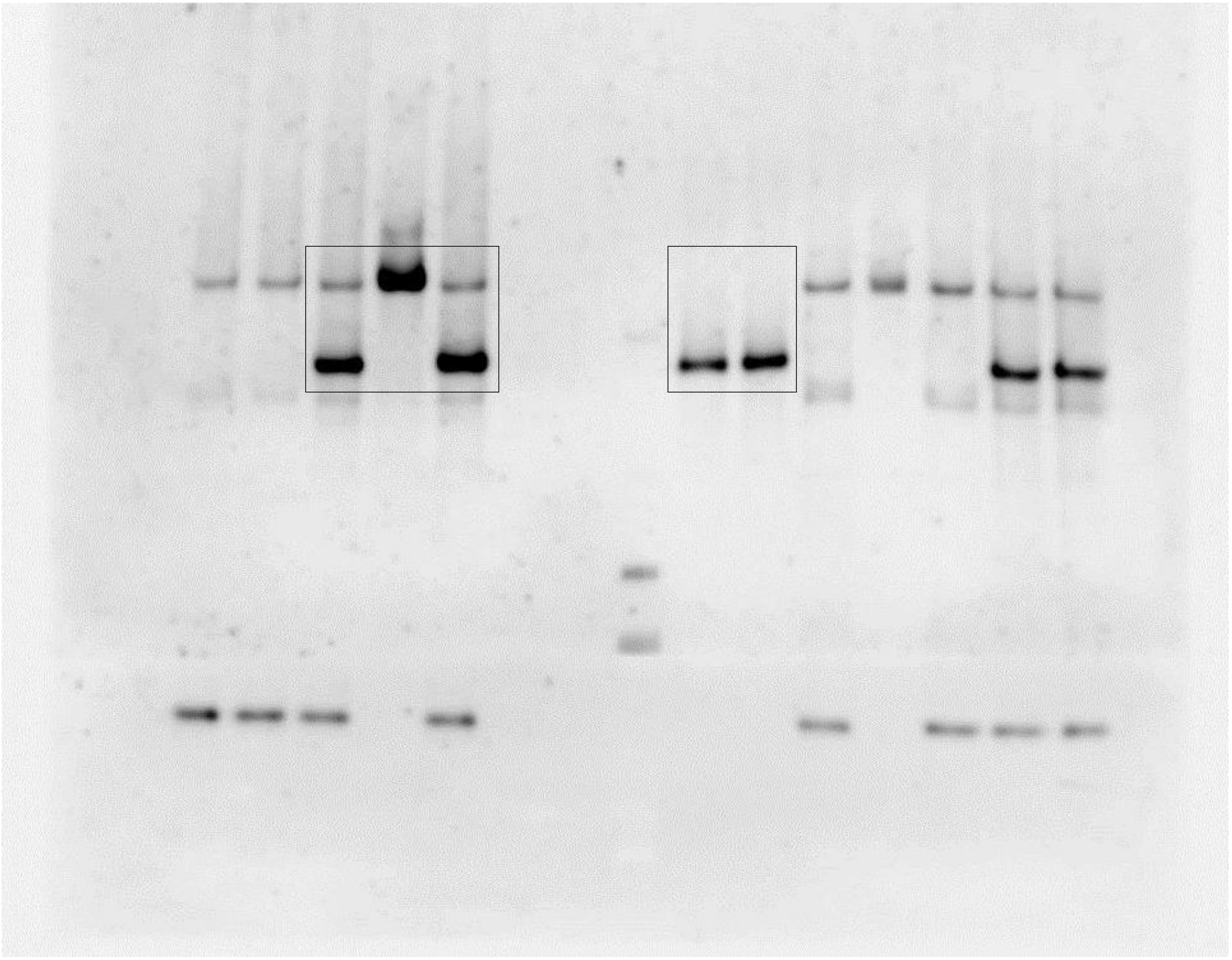

Suppl Figure 1b

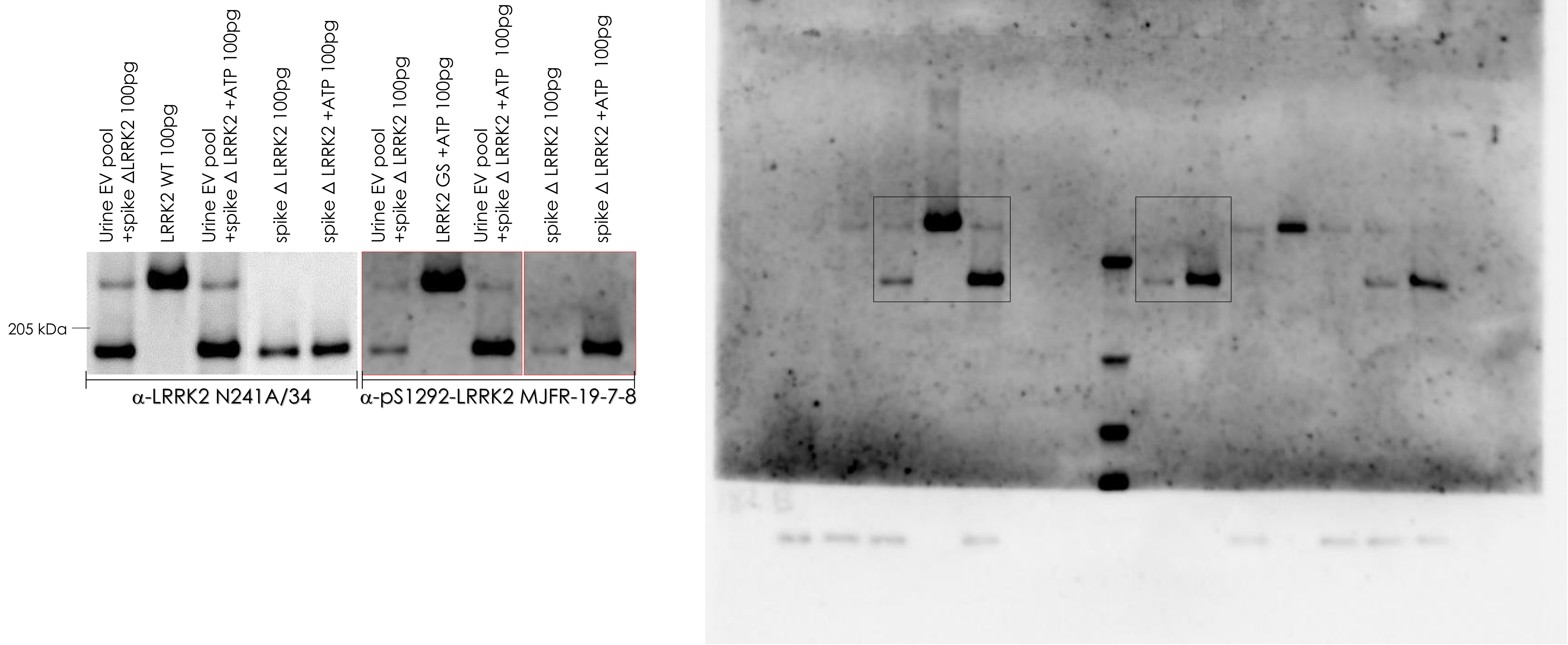

Suppl Figure 3a

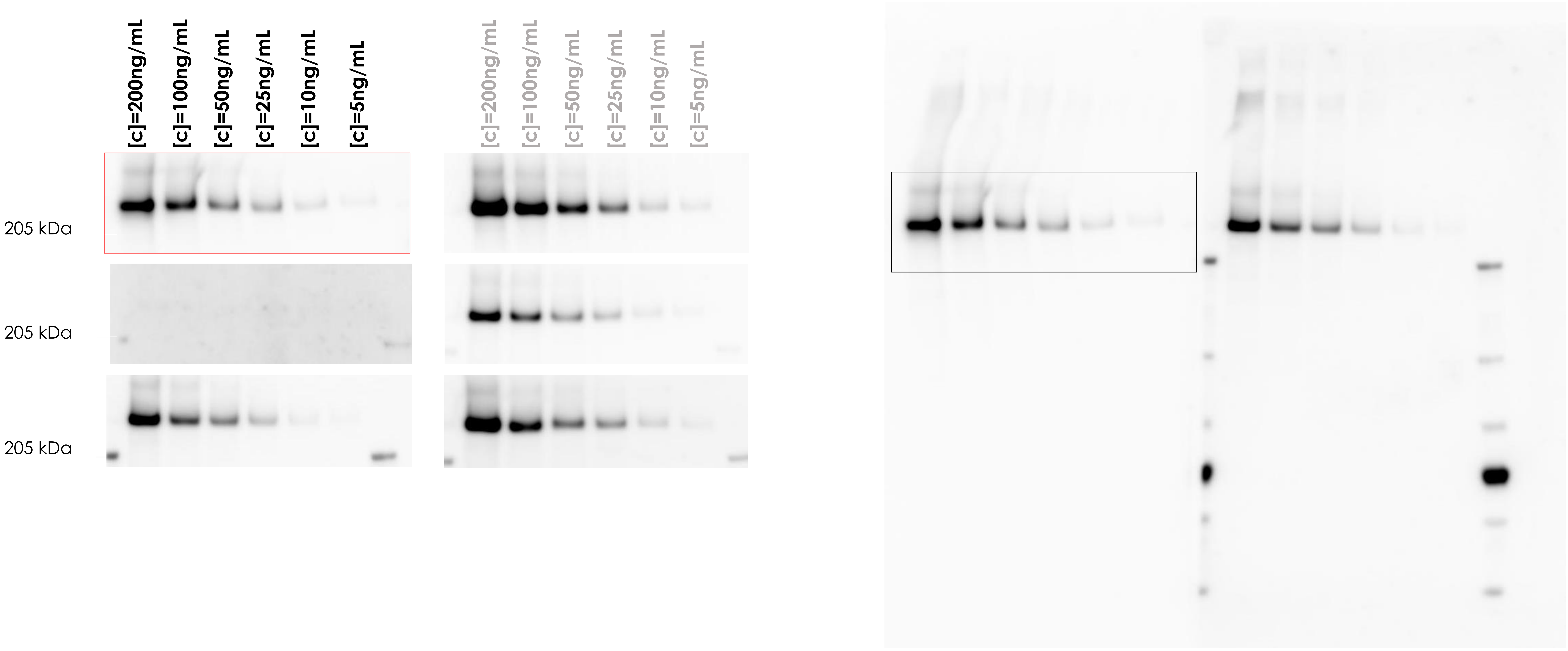

Suppl Figure 3a

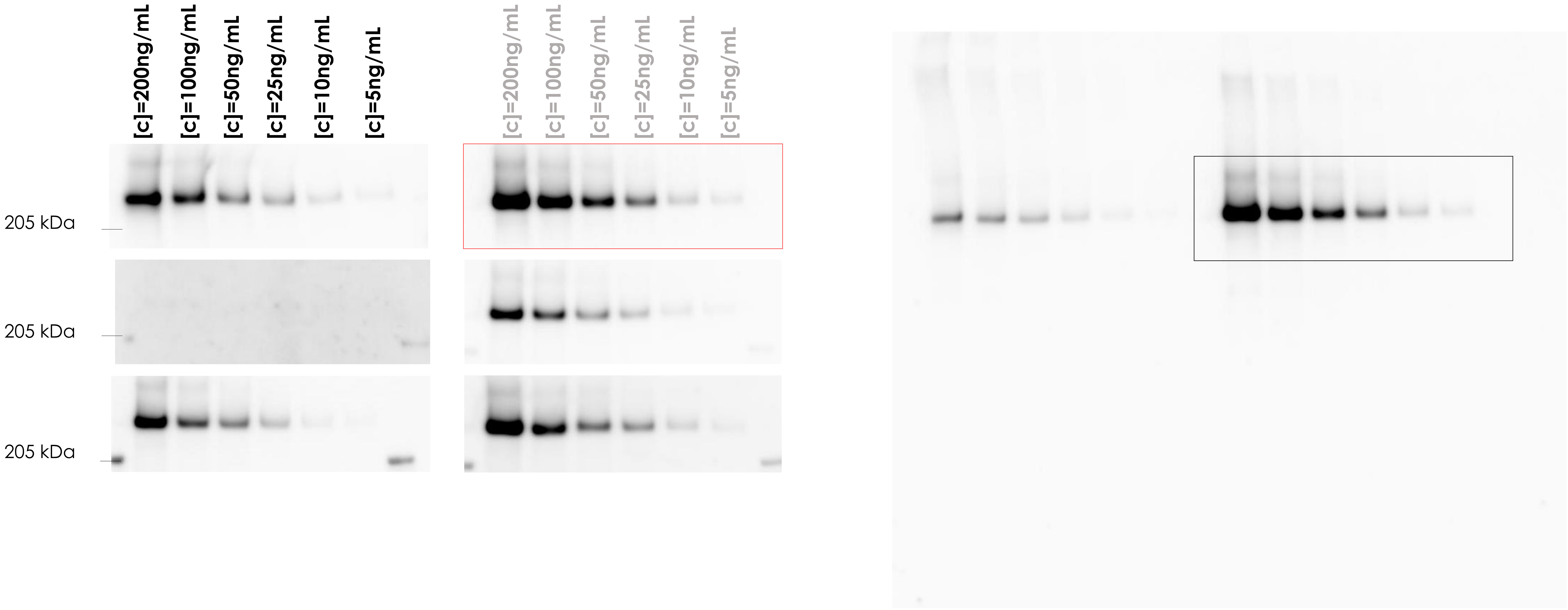

Suppl Figure 3a

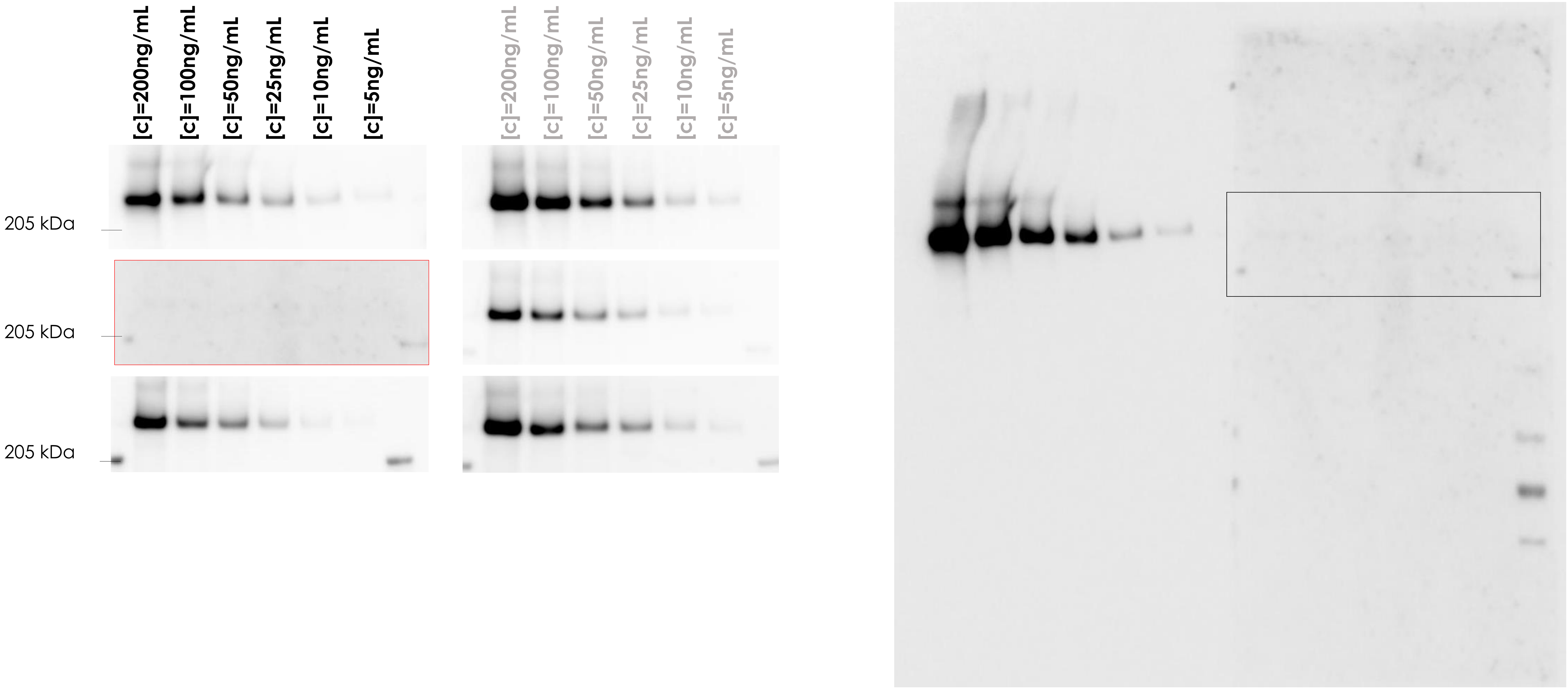

Suppl Figure 3a

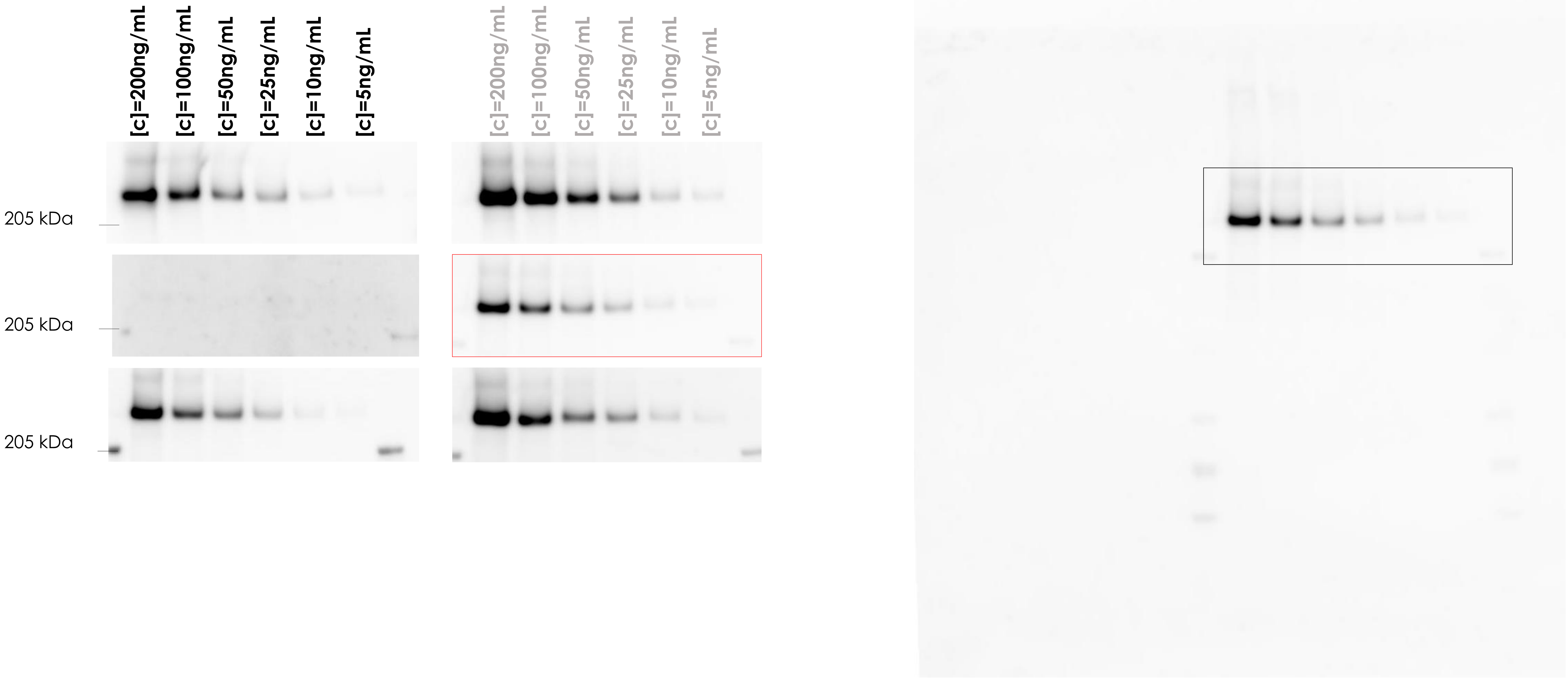

Suppl Figure 3a

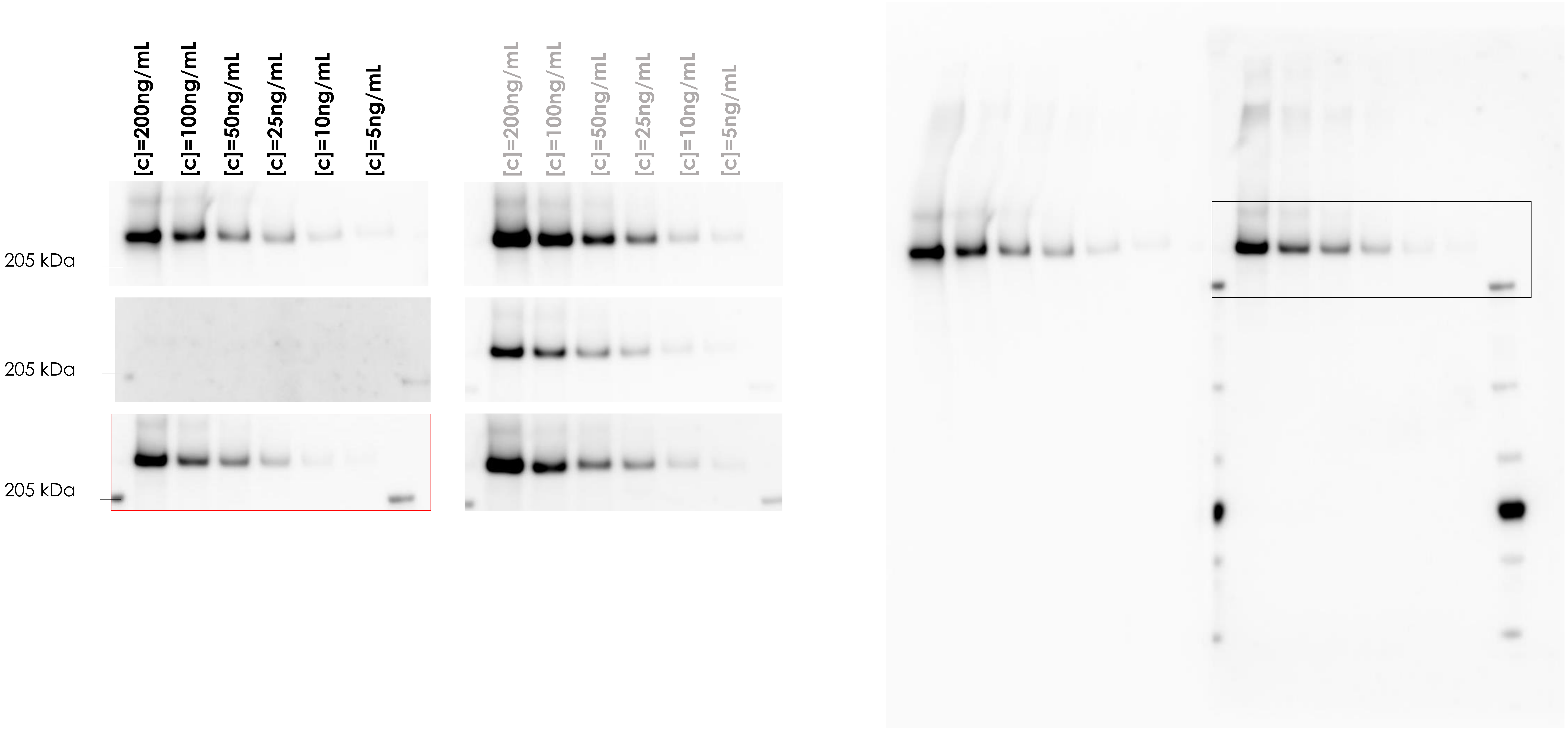

Suppl Figure 3a

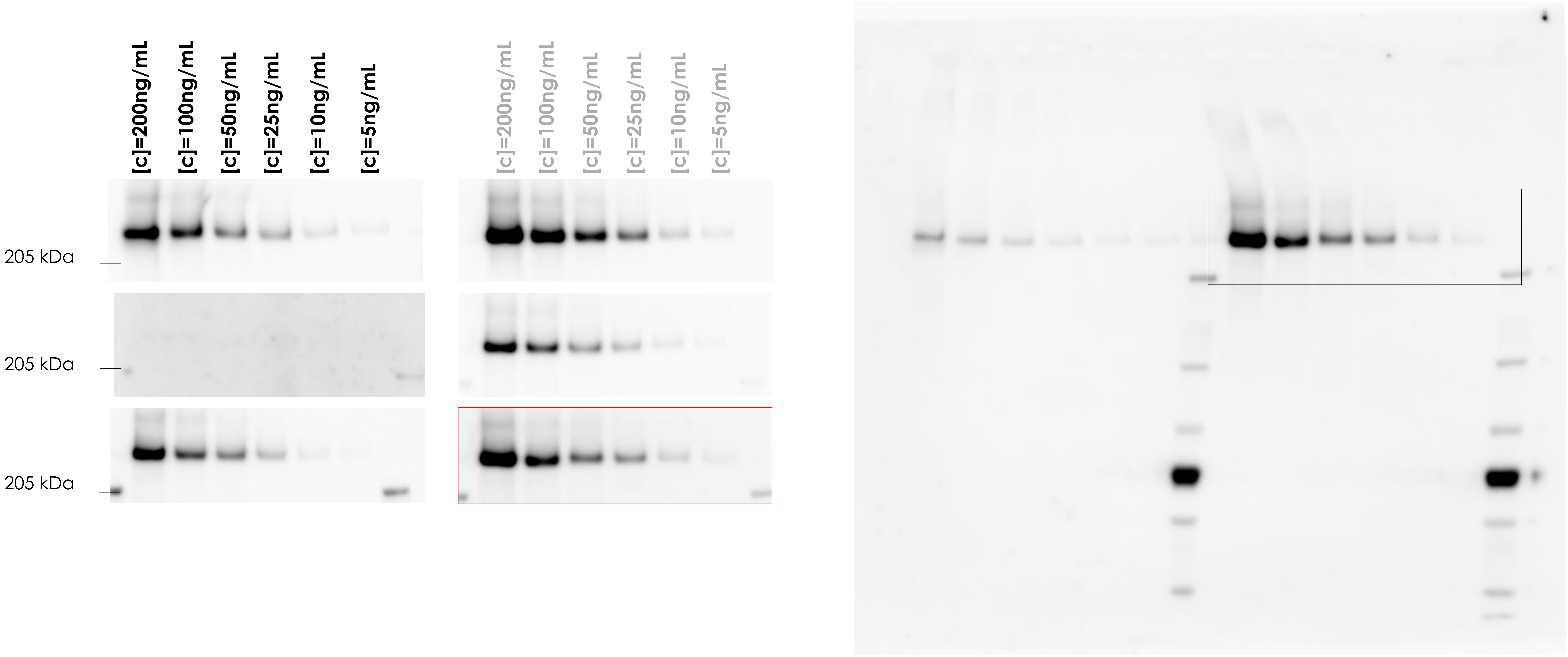

Suppl Figure 5a

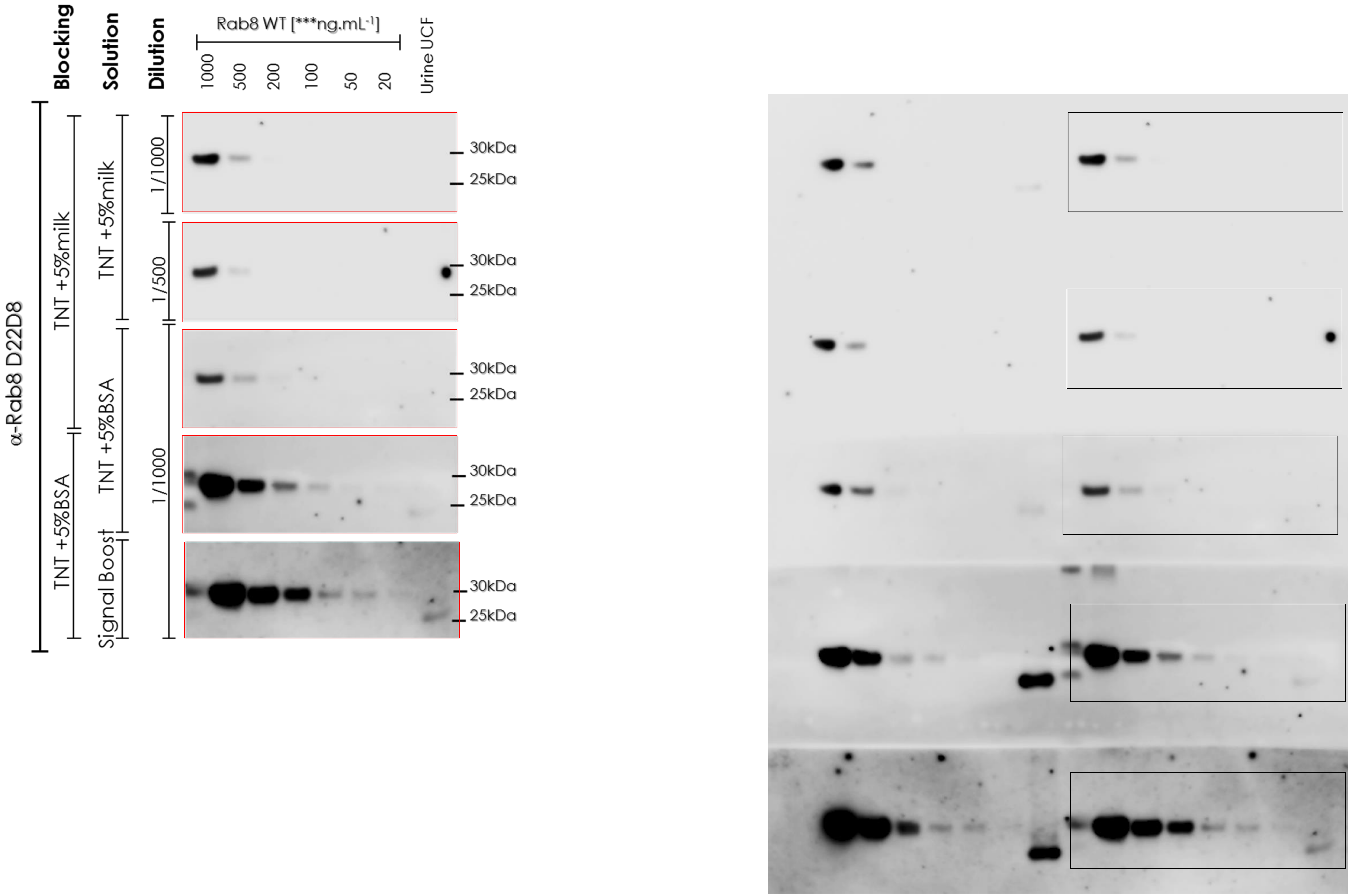

Suppl Figure 5b

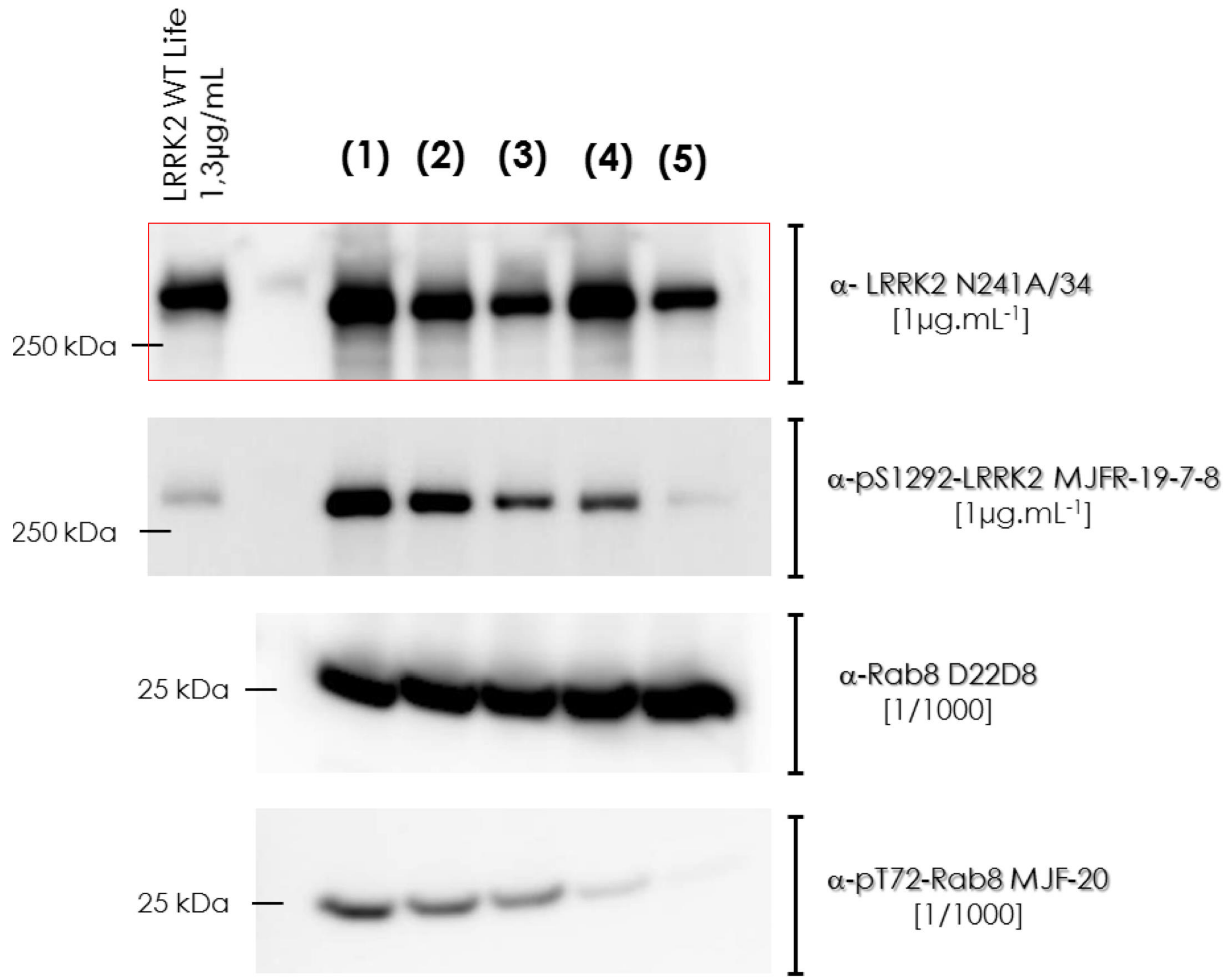

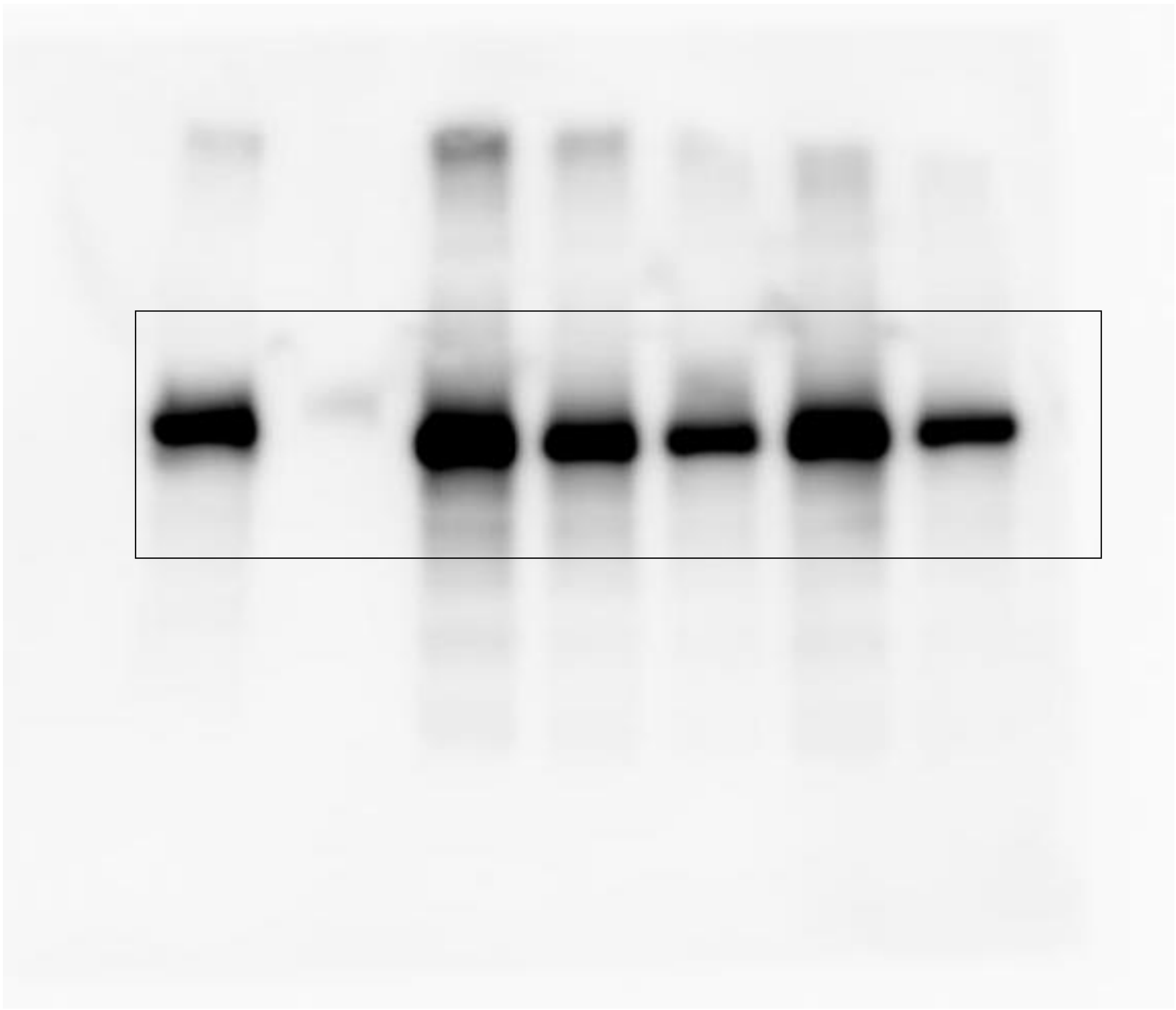

Suppl Figure 5b

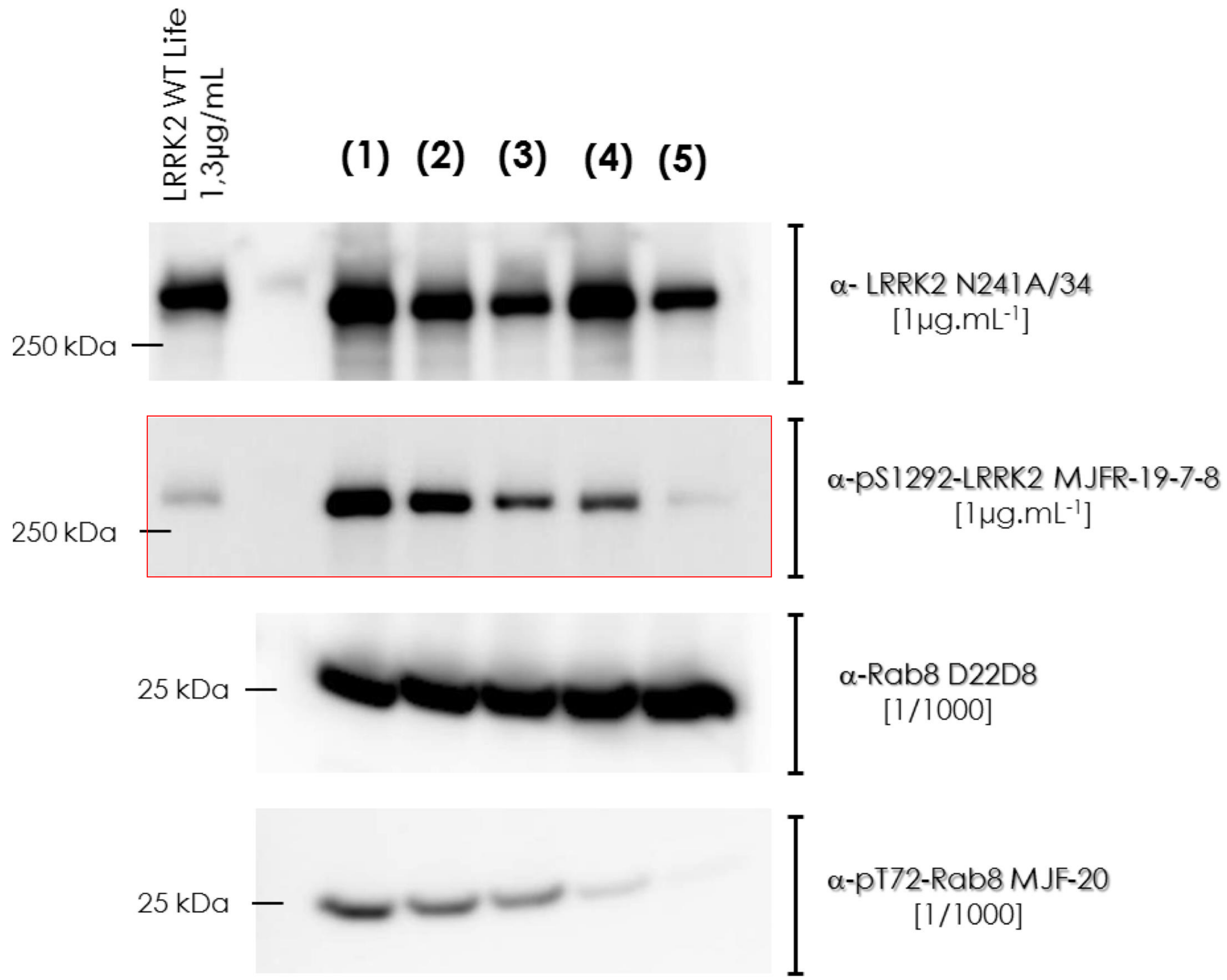

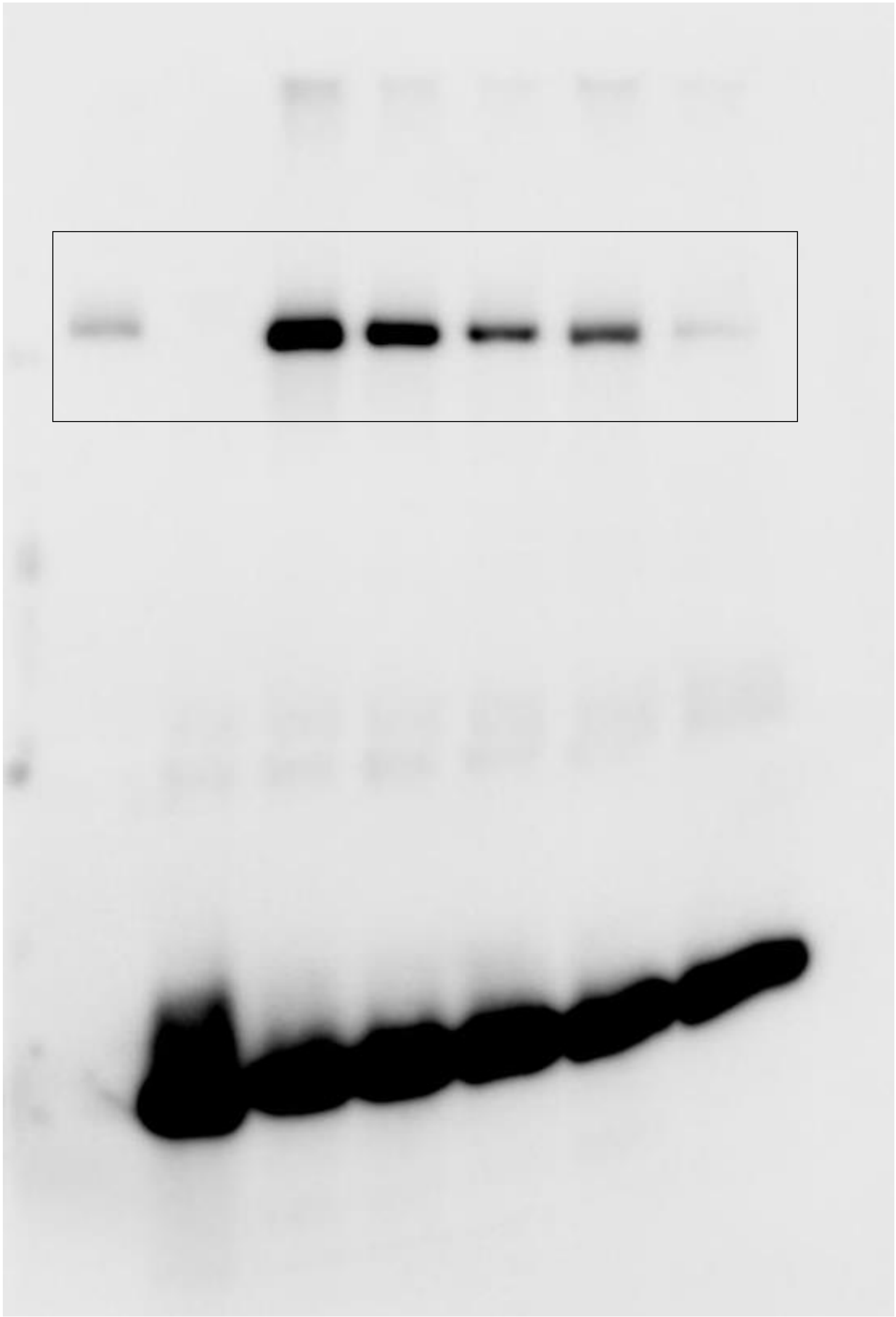

Suppl Figure 5b

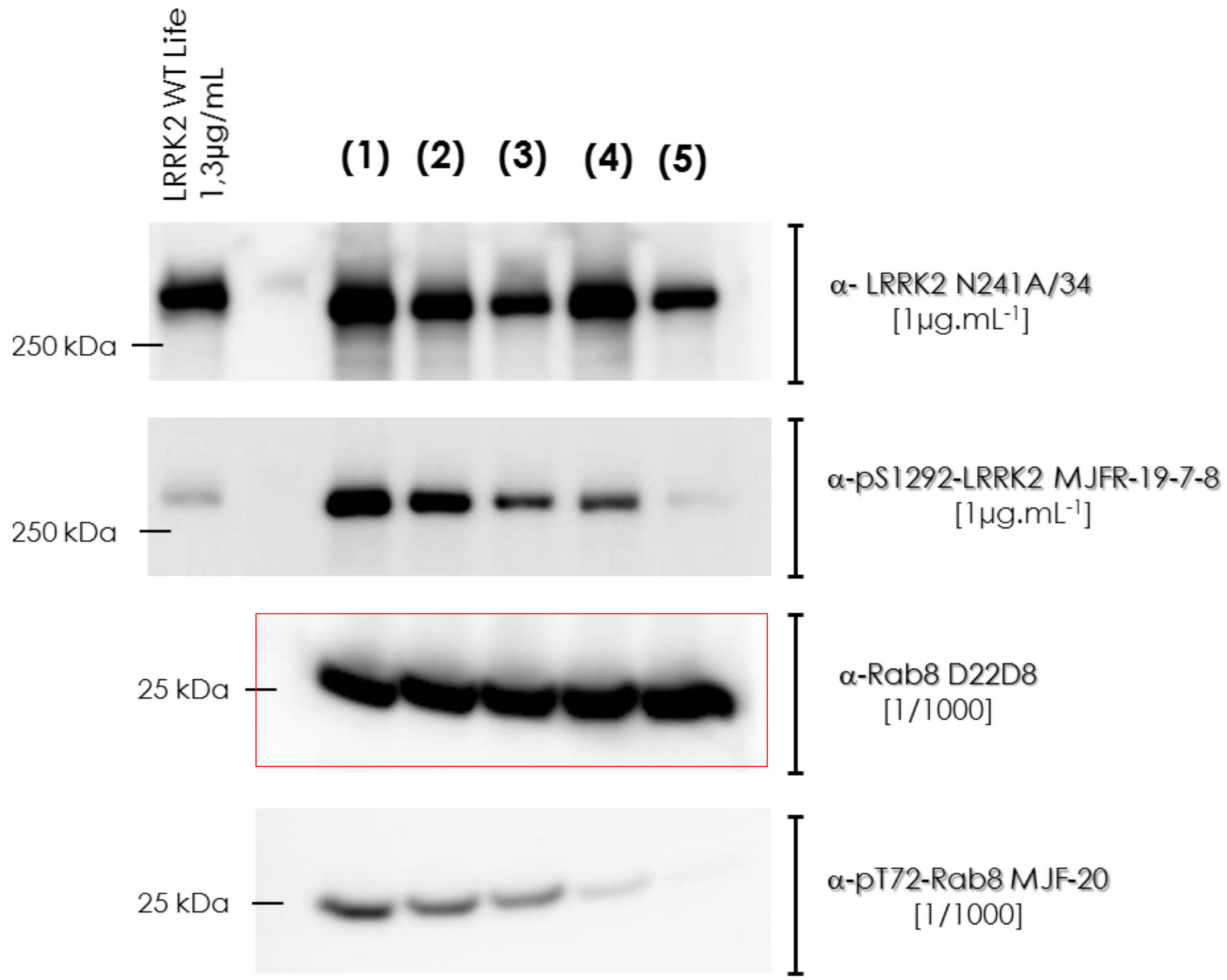

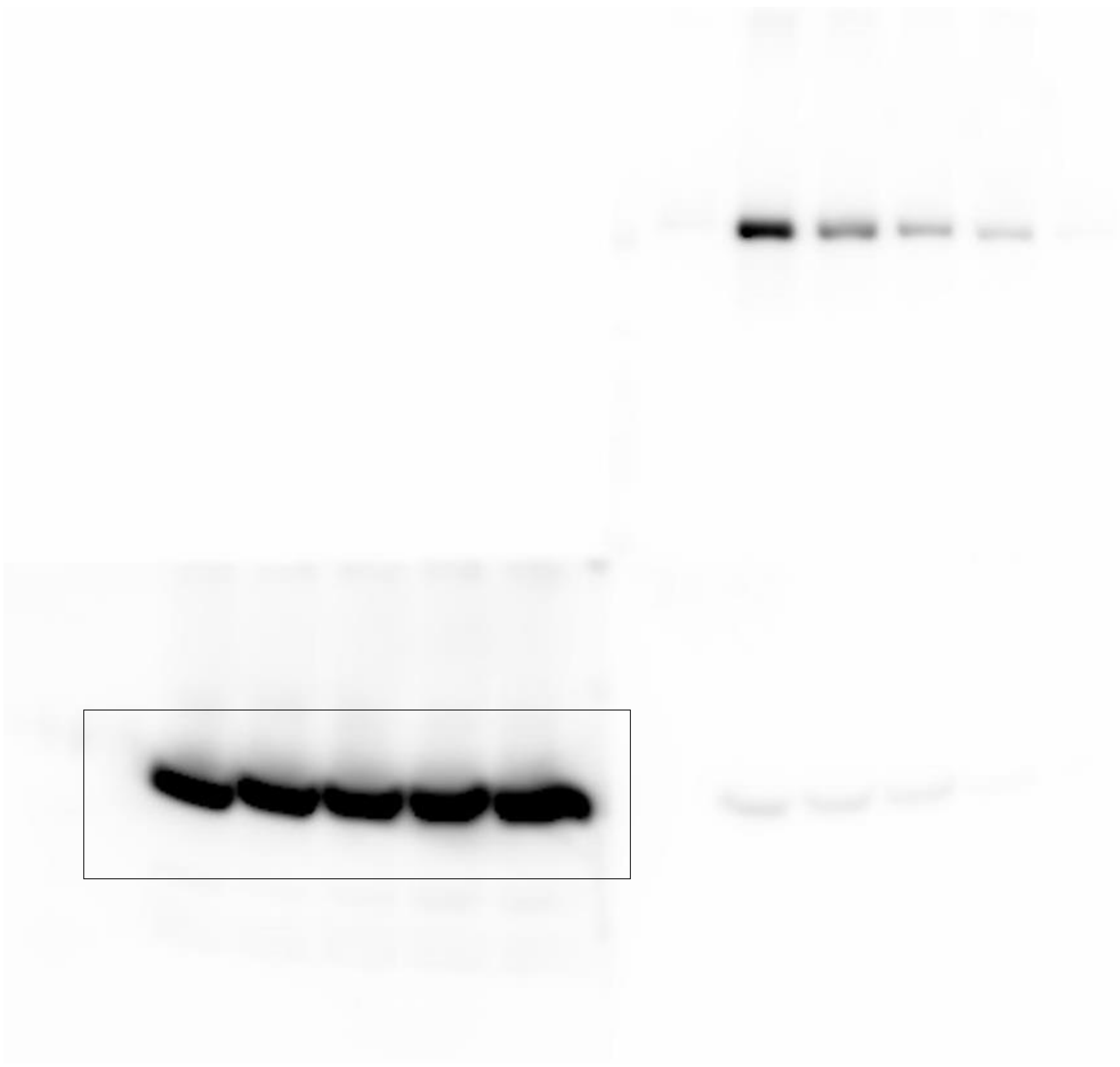

Suppl Figure 5b

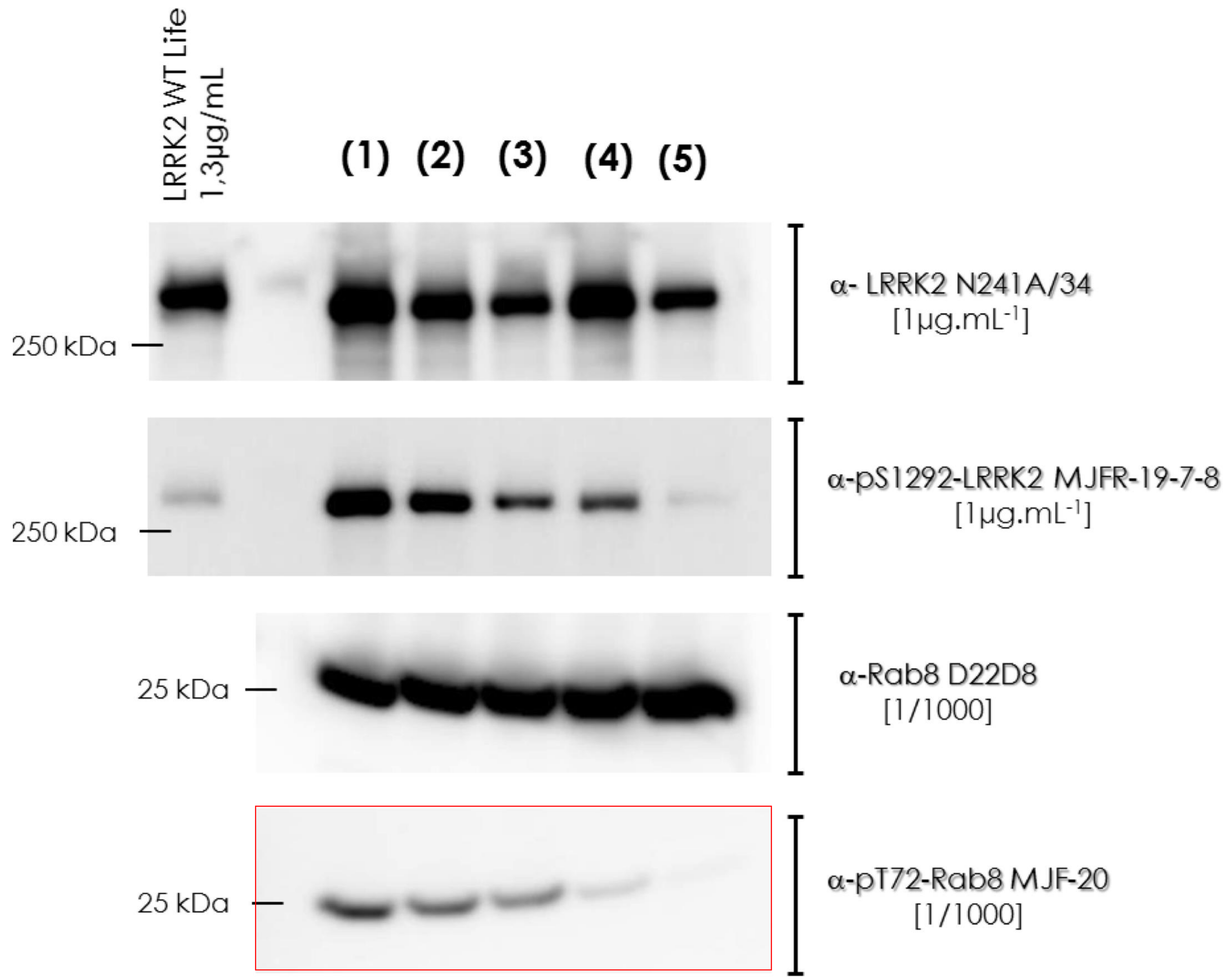

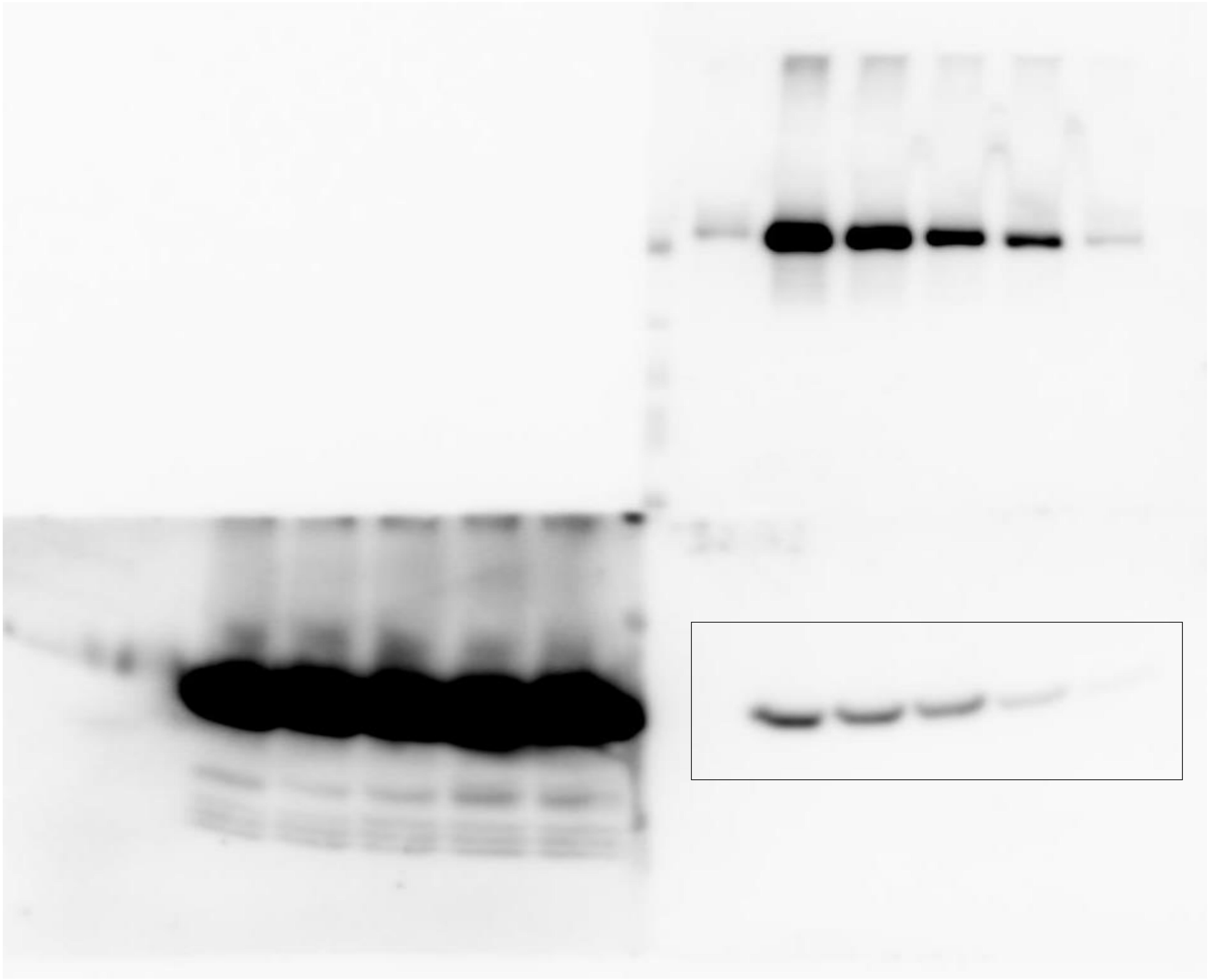

Suppl Figure S6

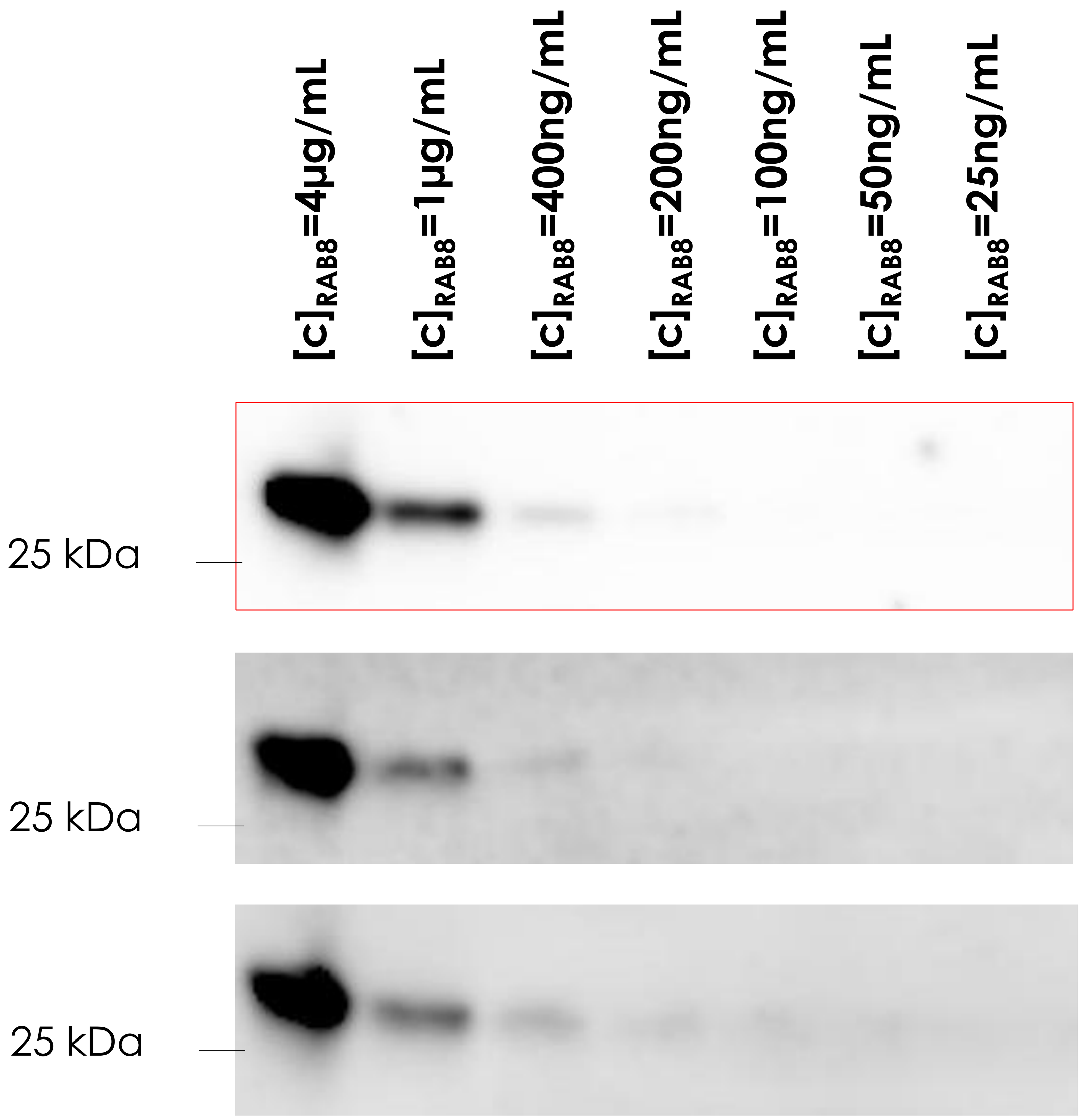

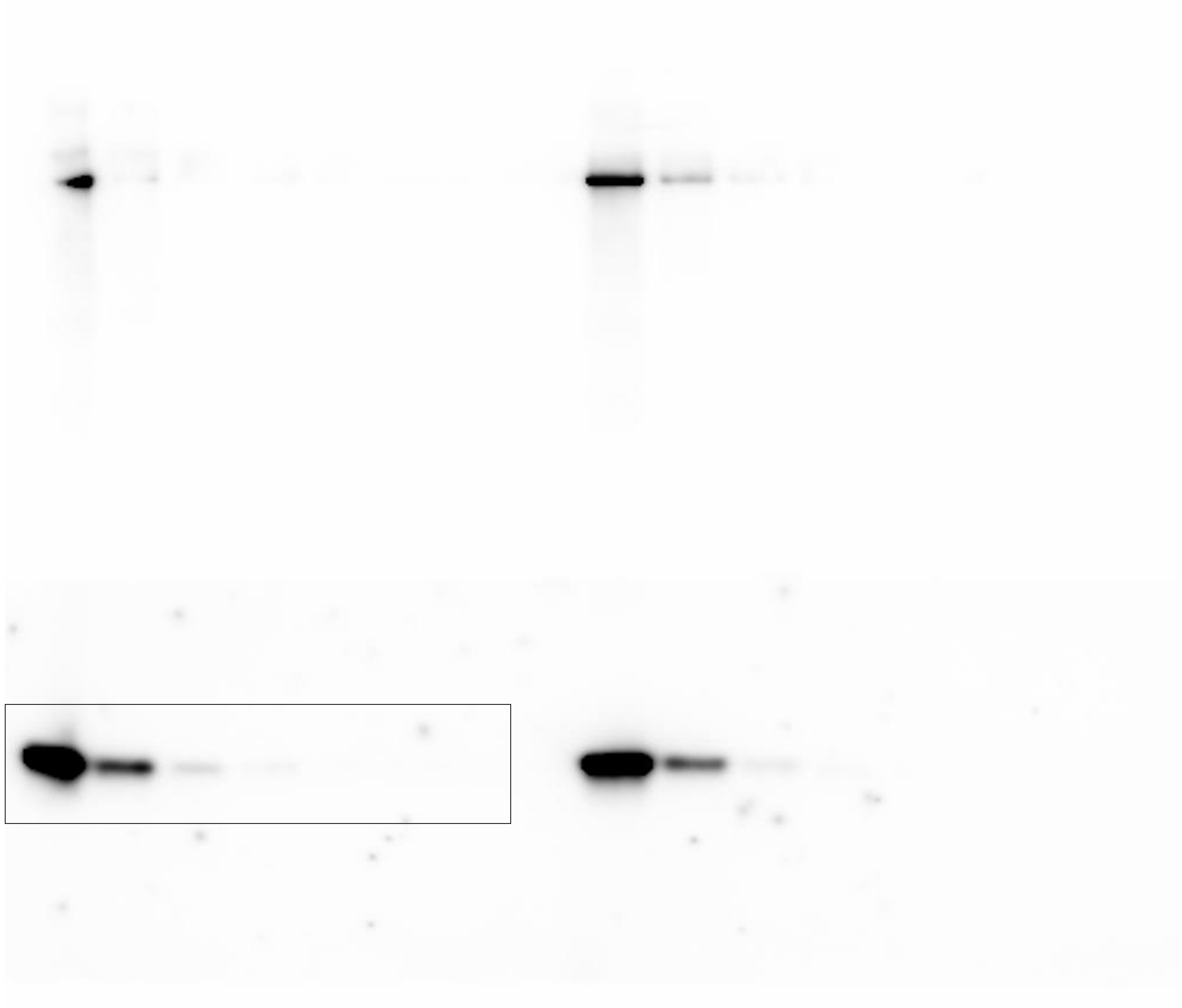

Suppl Figure S6

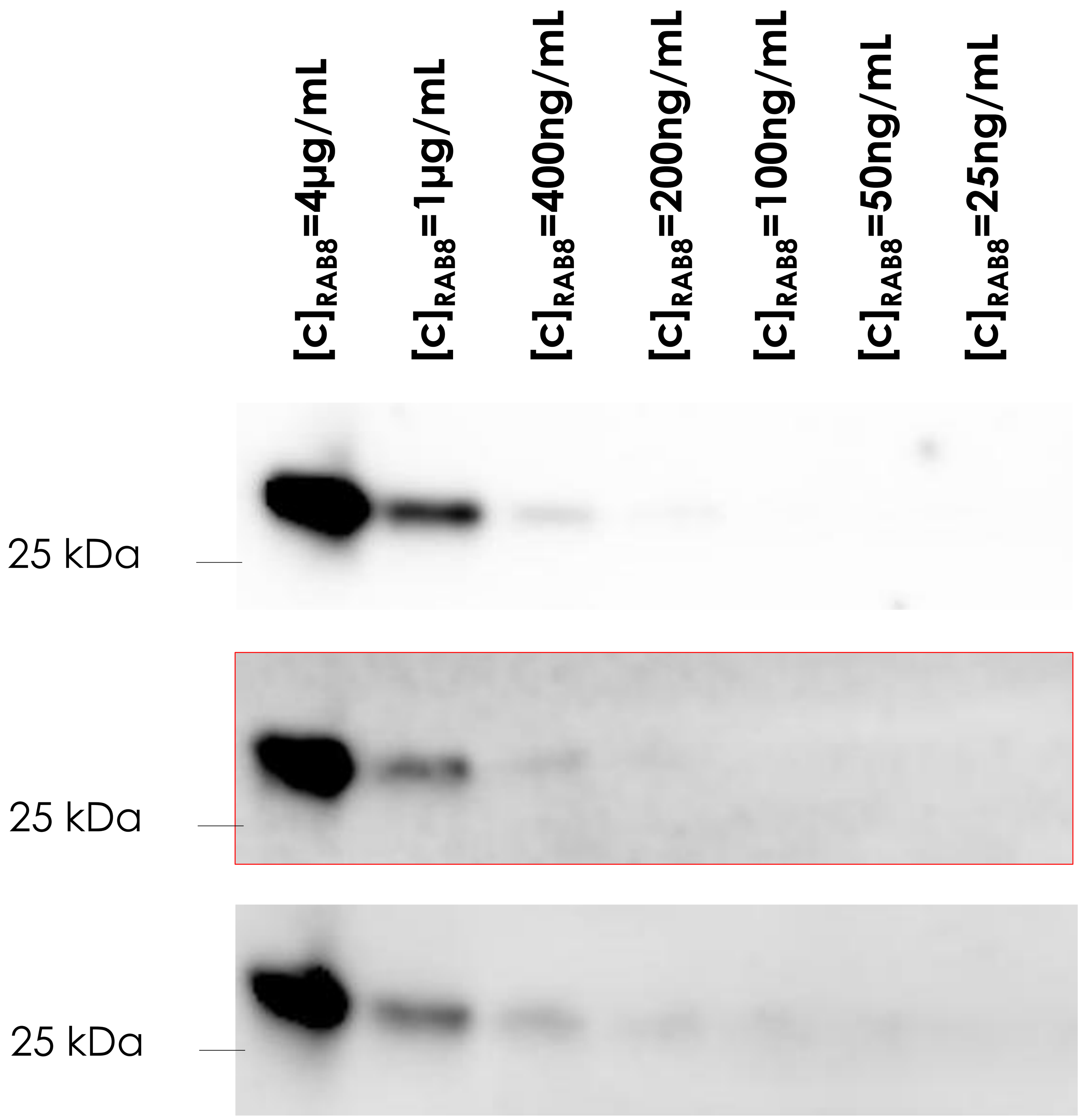

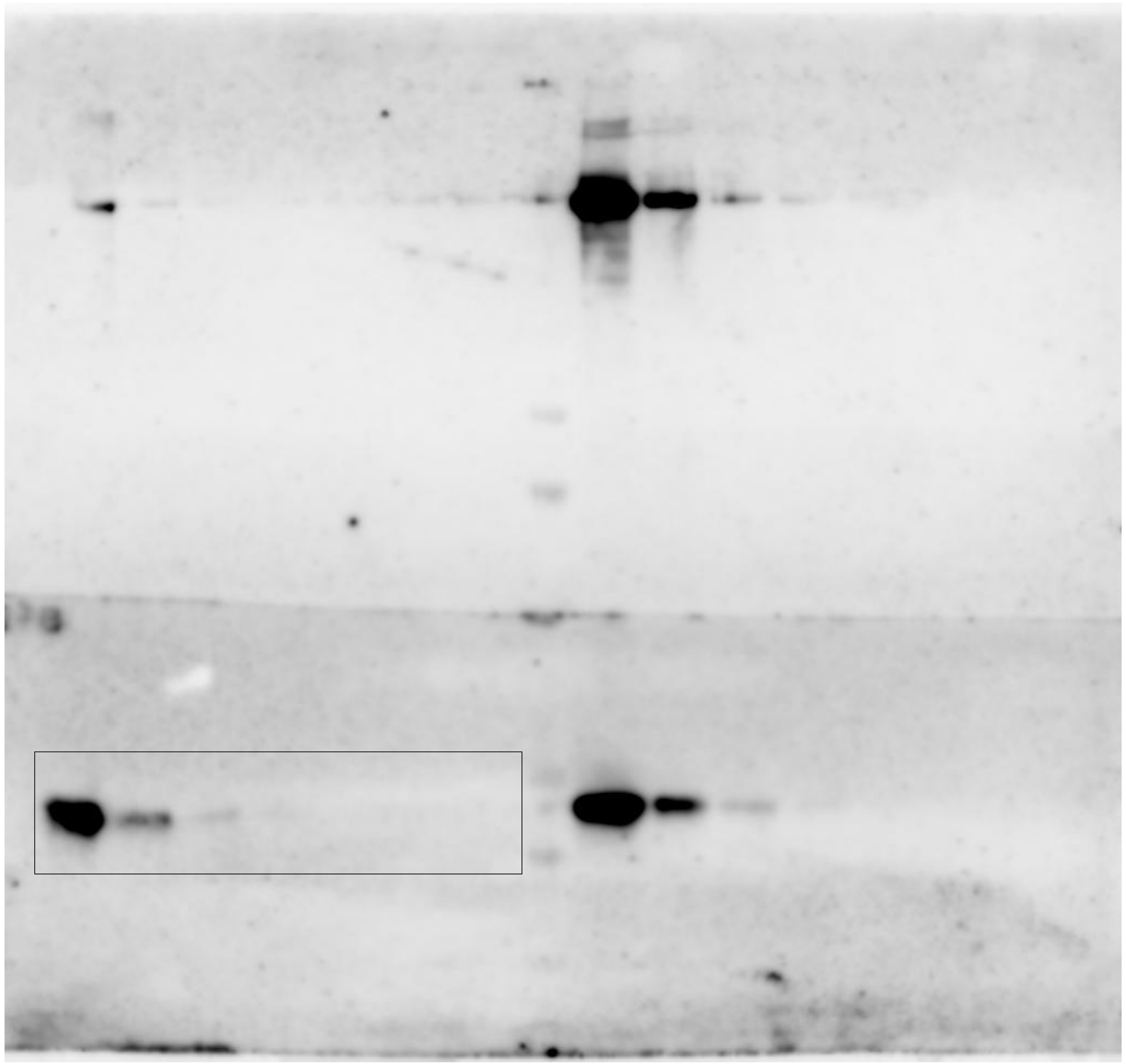

Suppl Figure S6

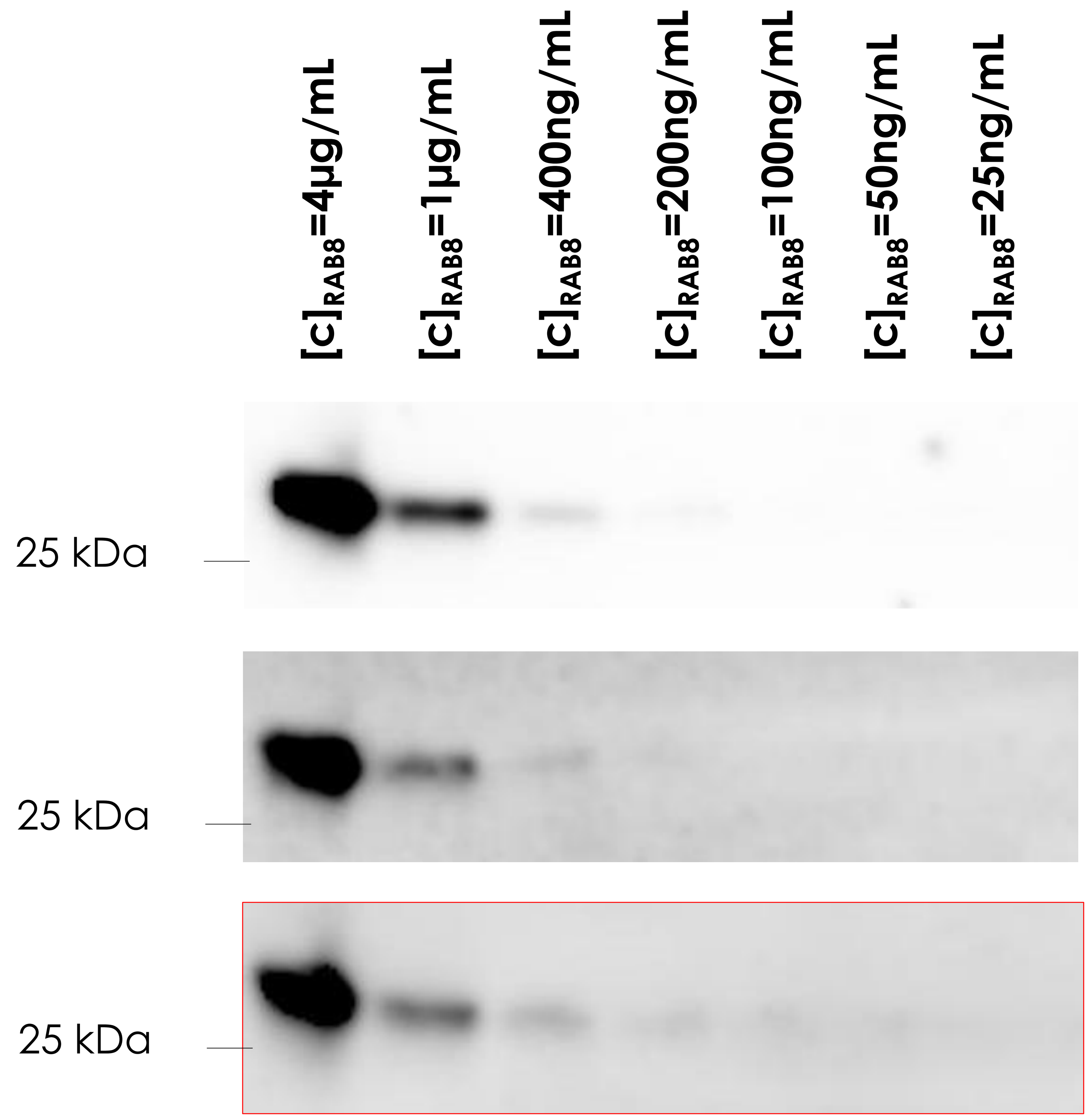

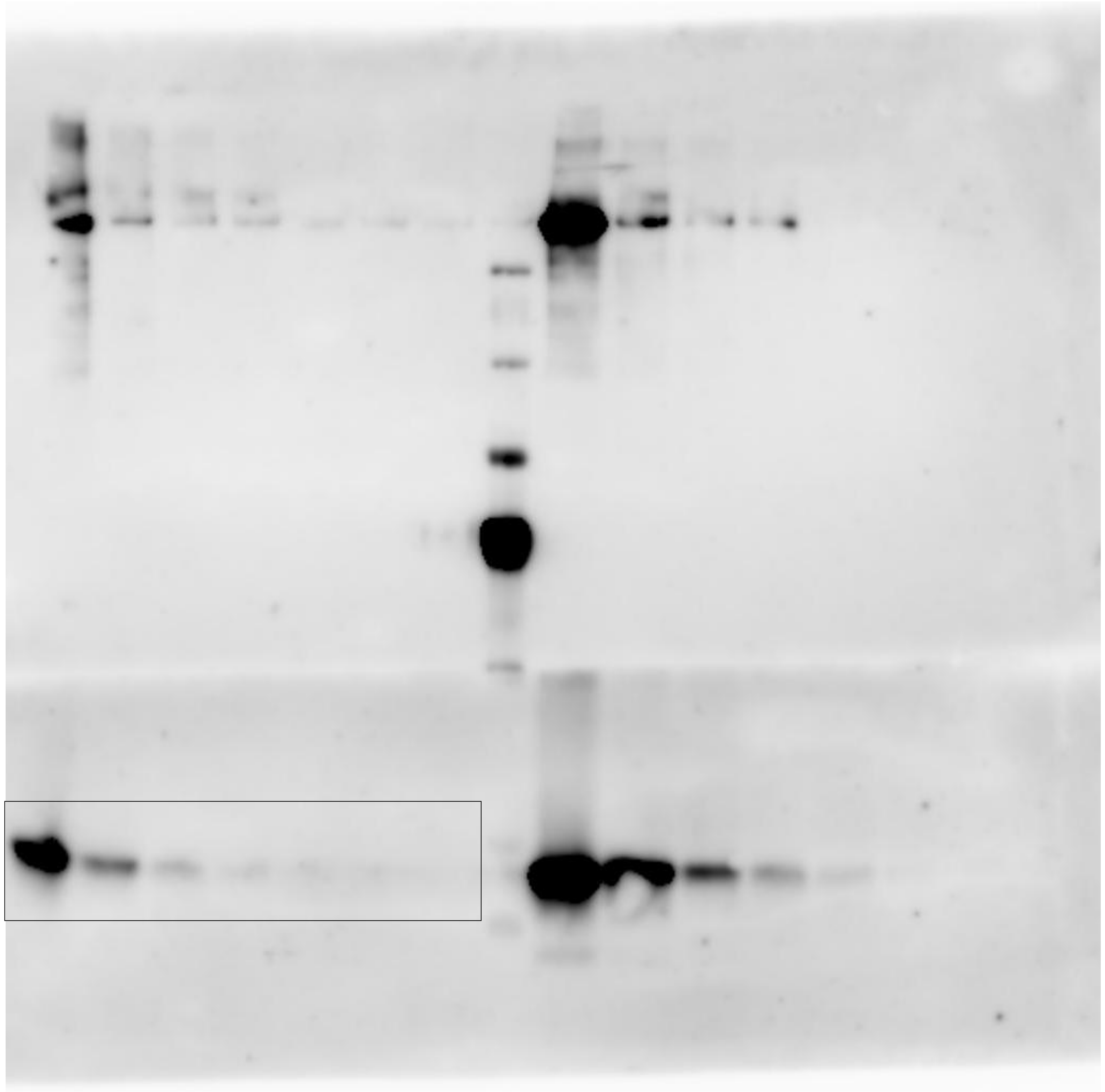

Suppl Figure S6

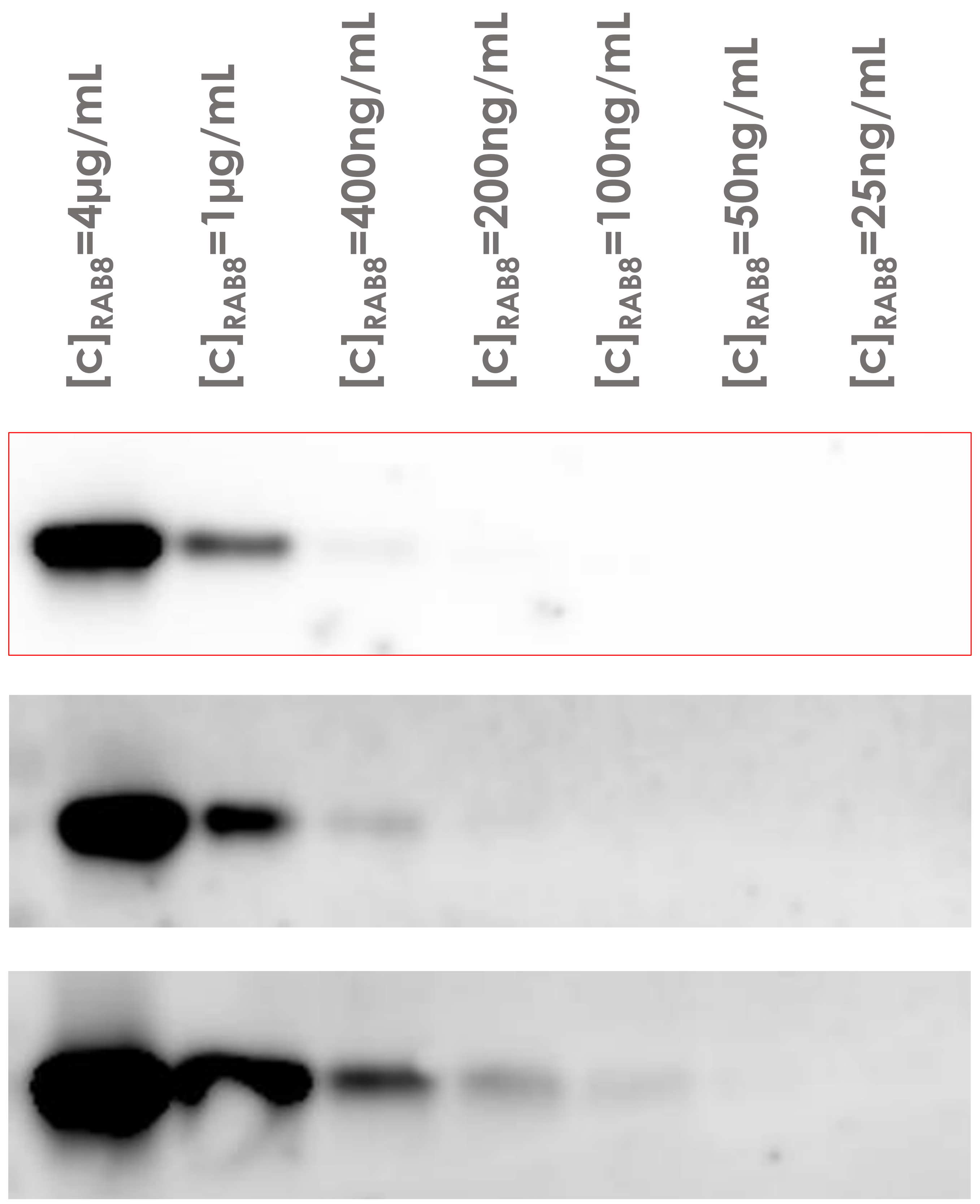

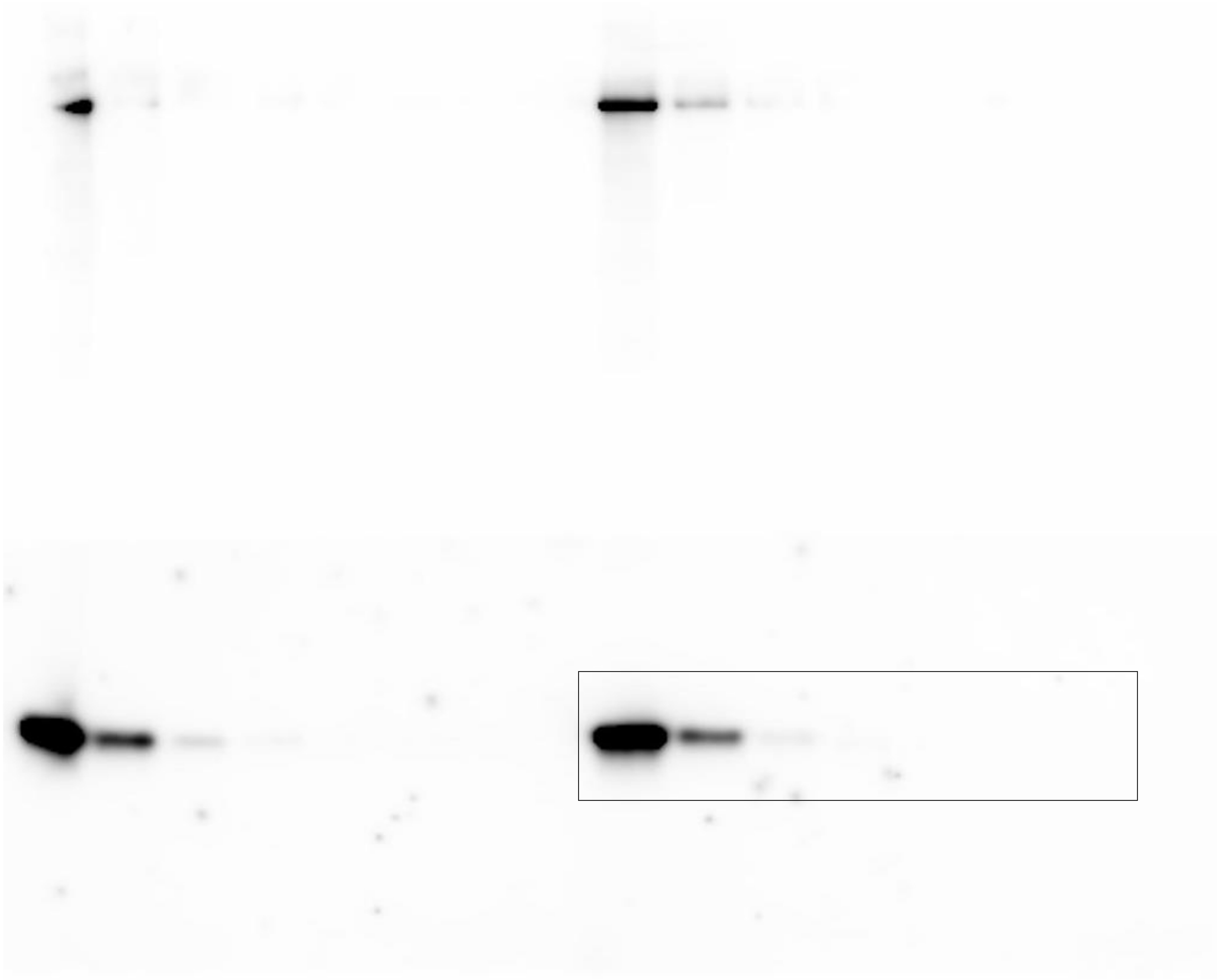

Suppl Figure S6

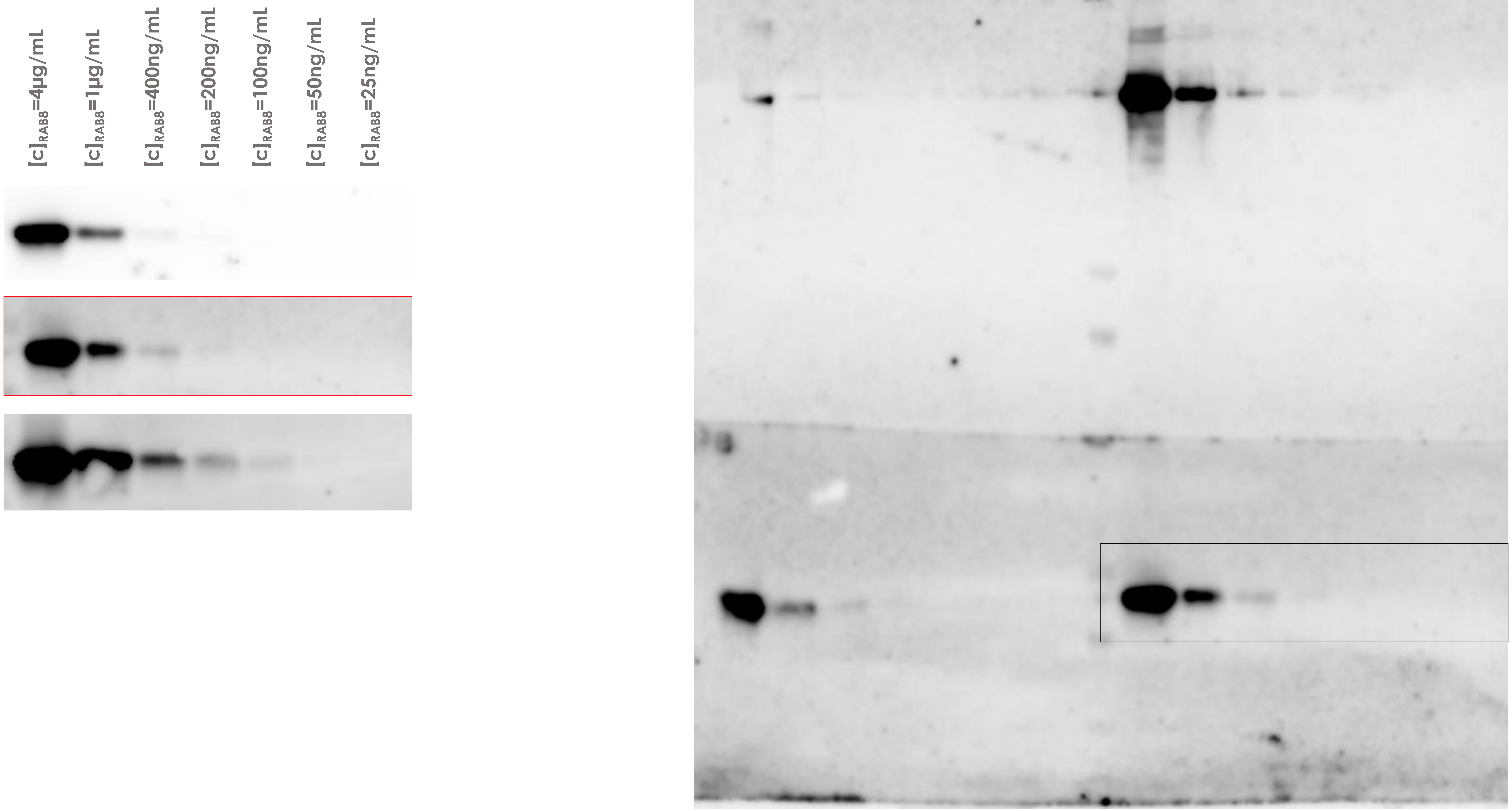

Suppl Figure S6

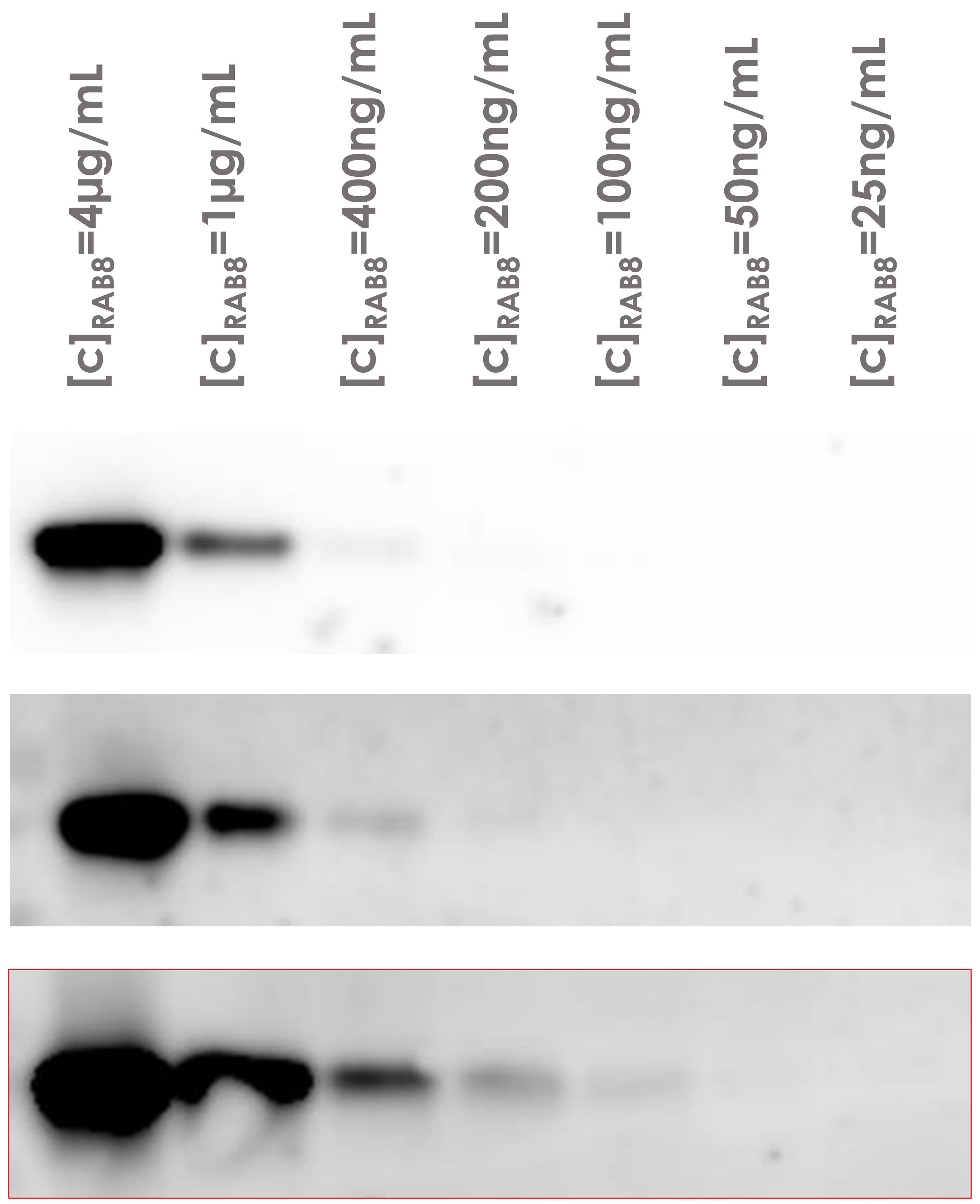

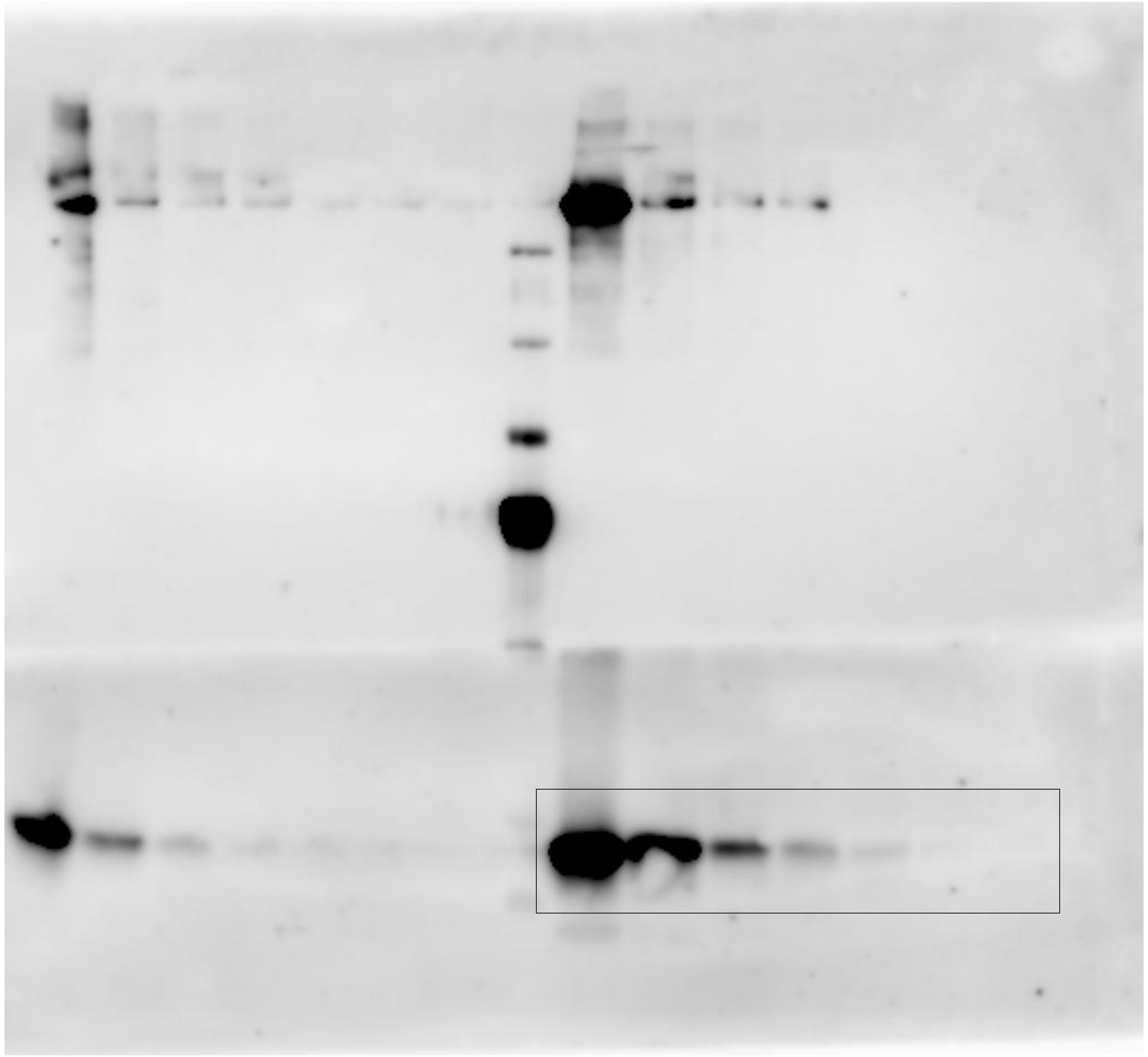

Suppl Figure 7

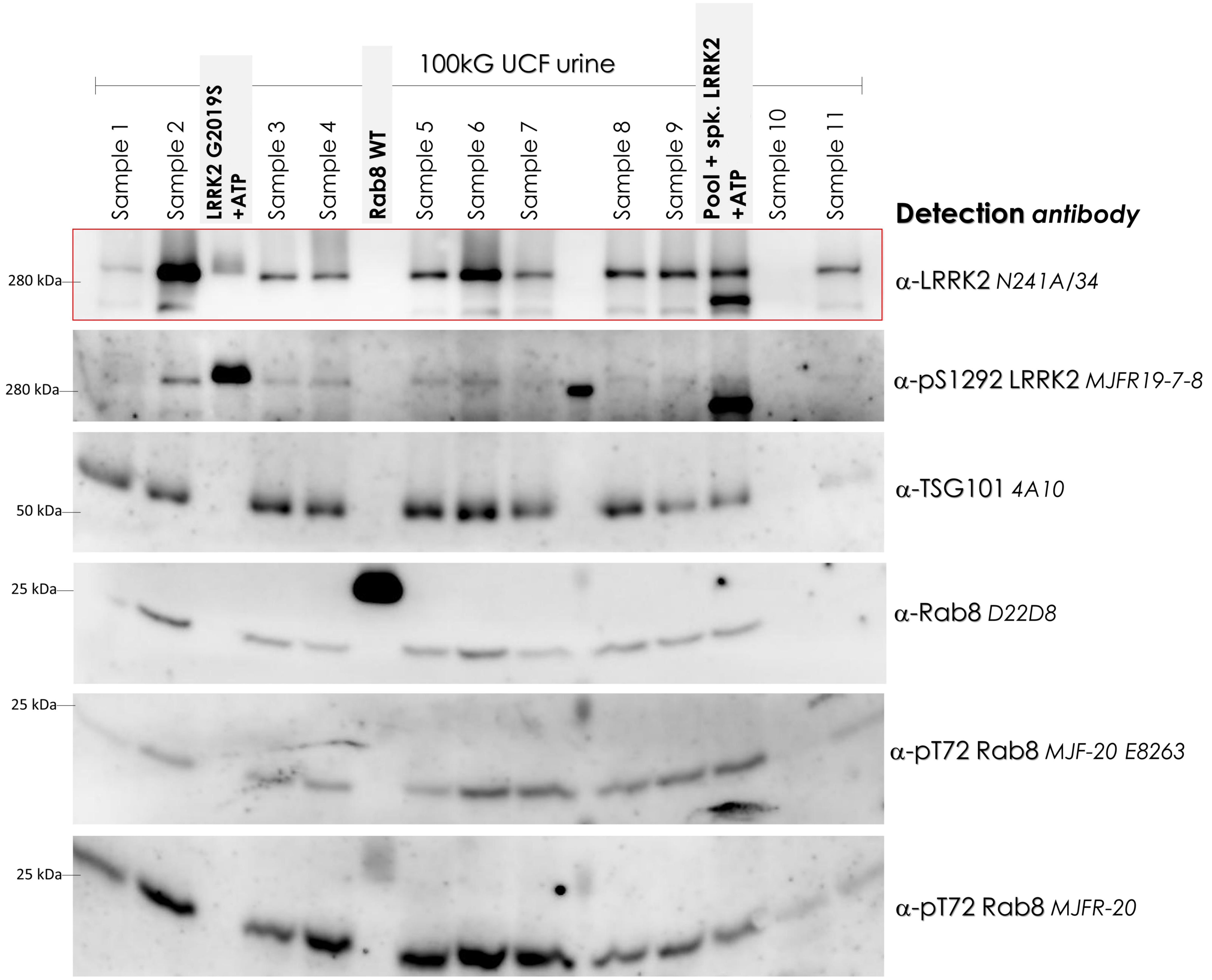

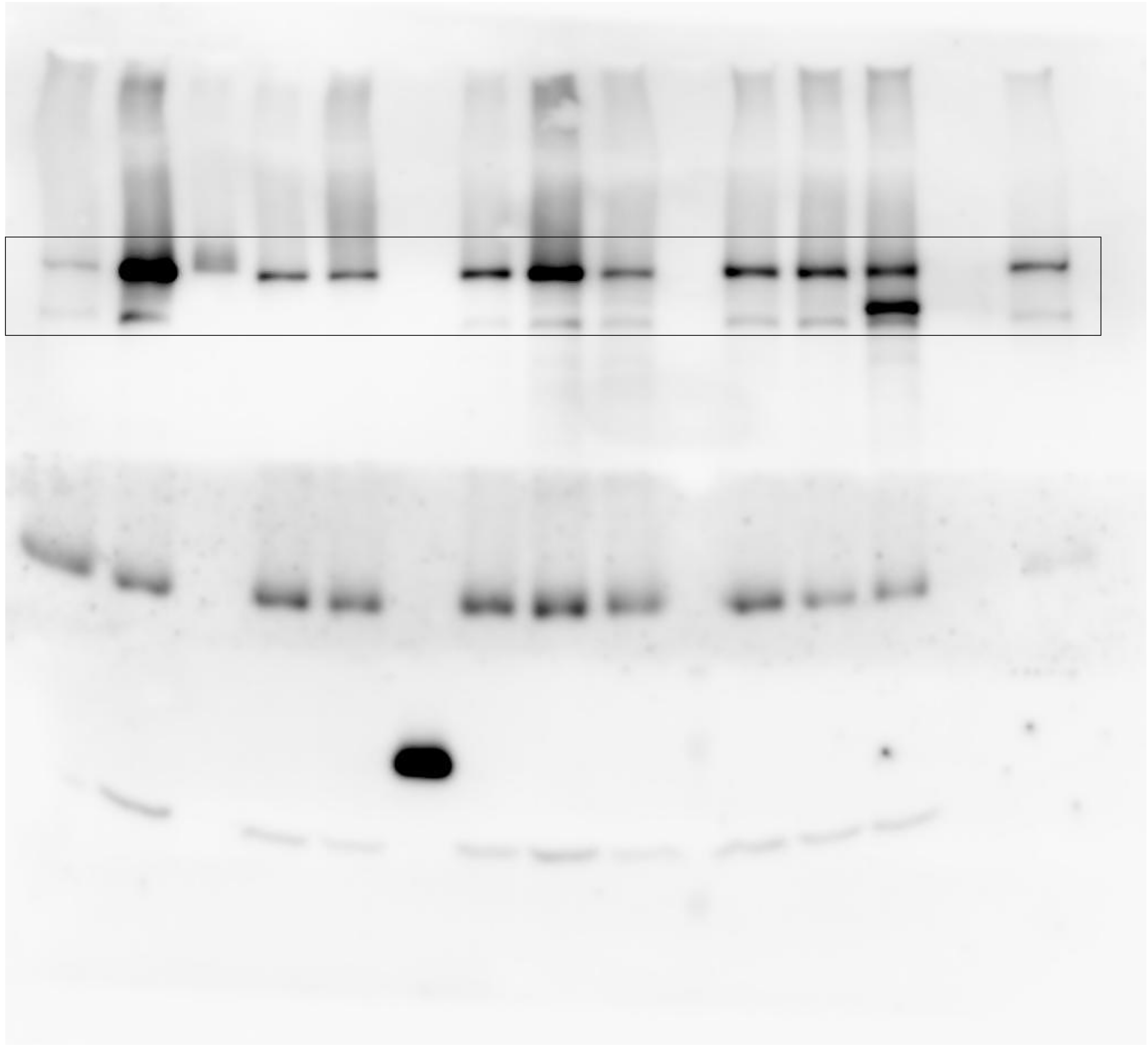

Suppl Figure 7

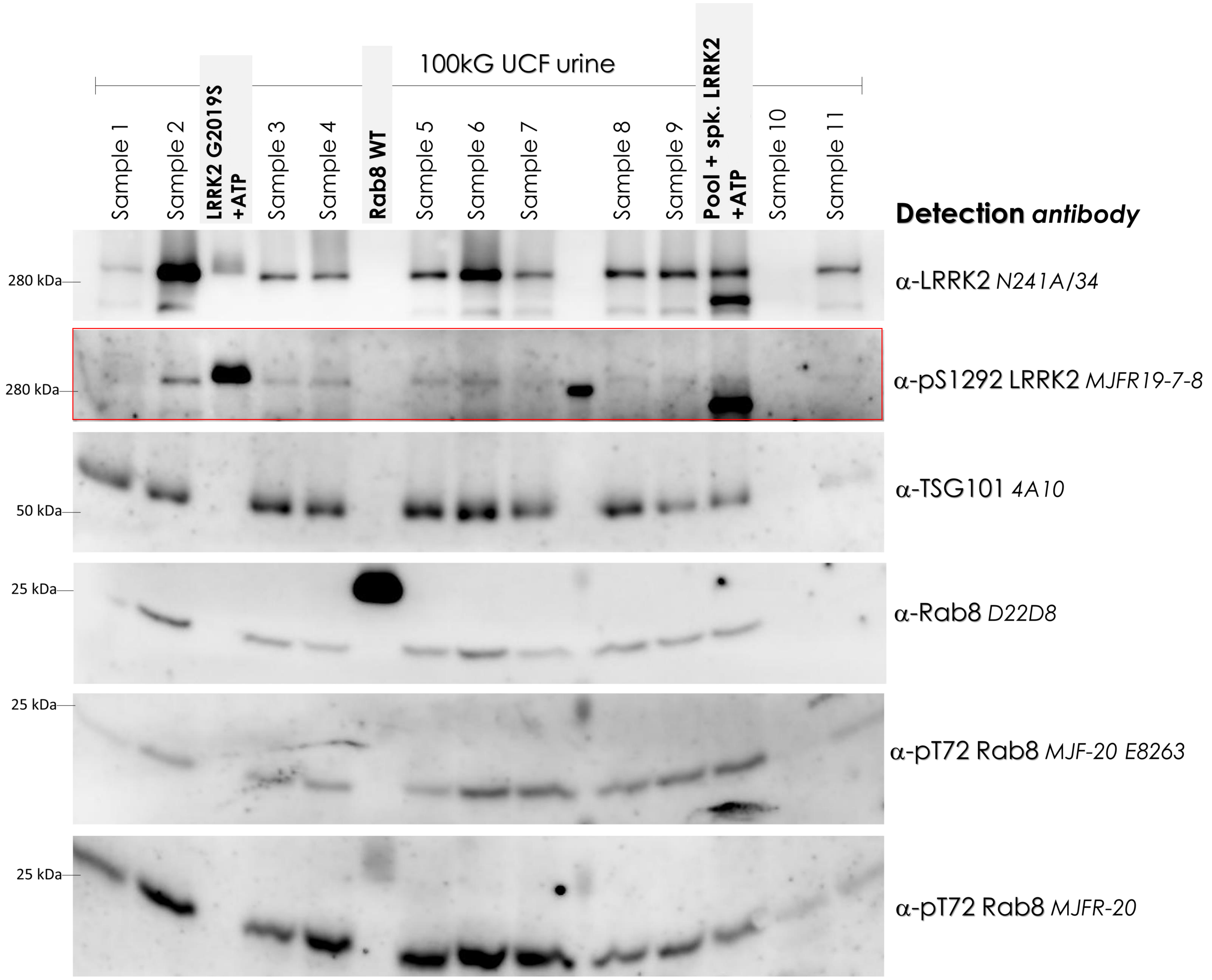

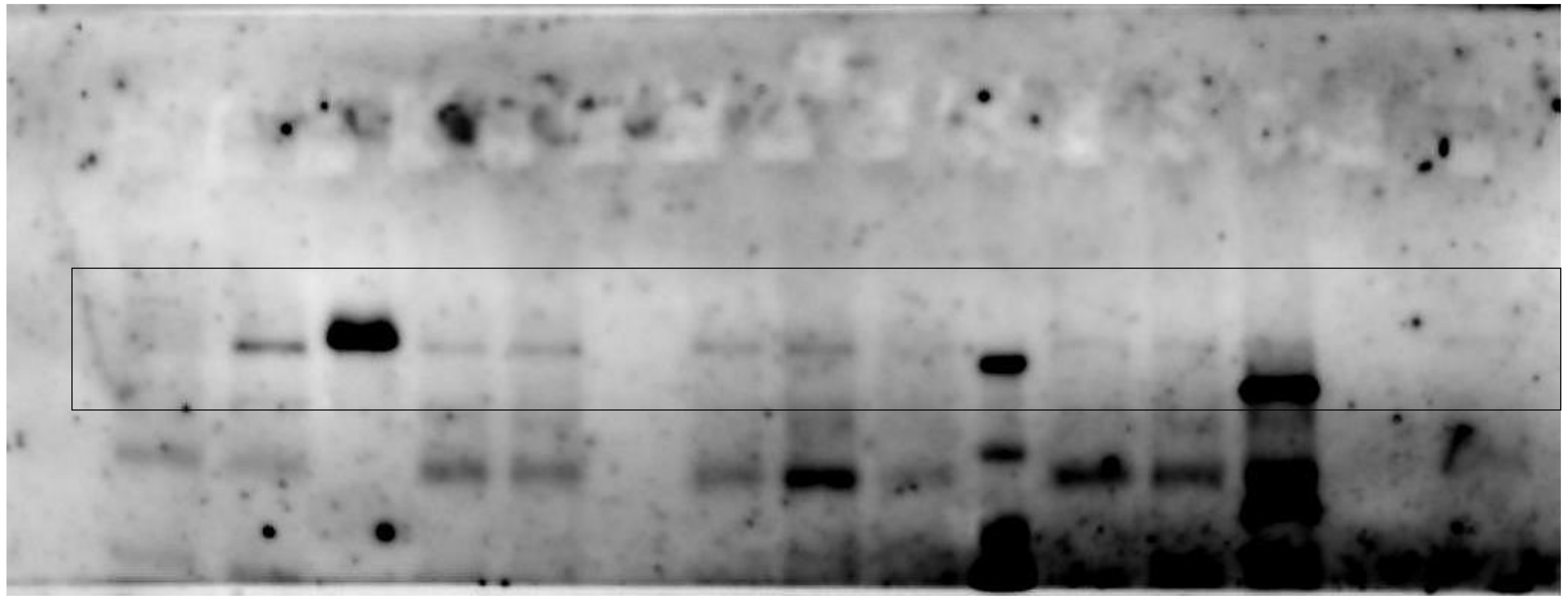

Suppl Figure 7

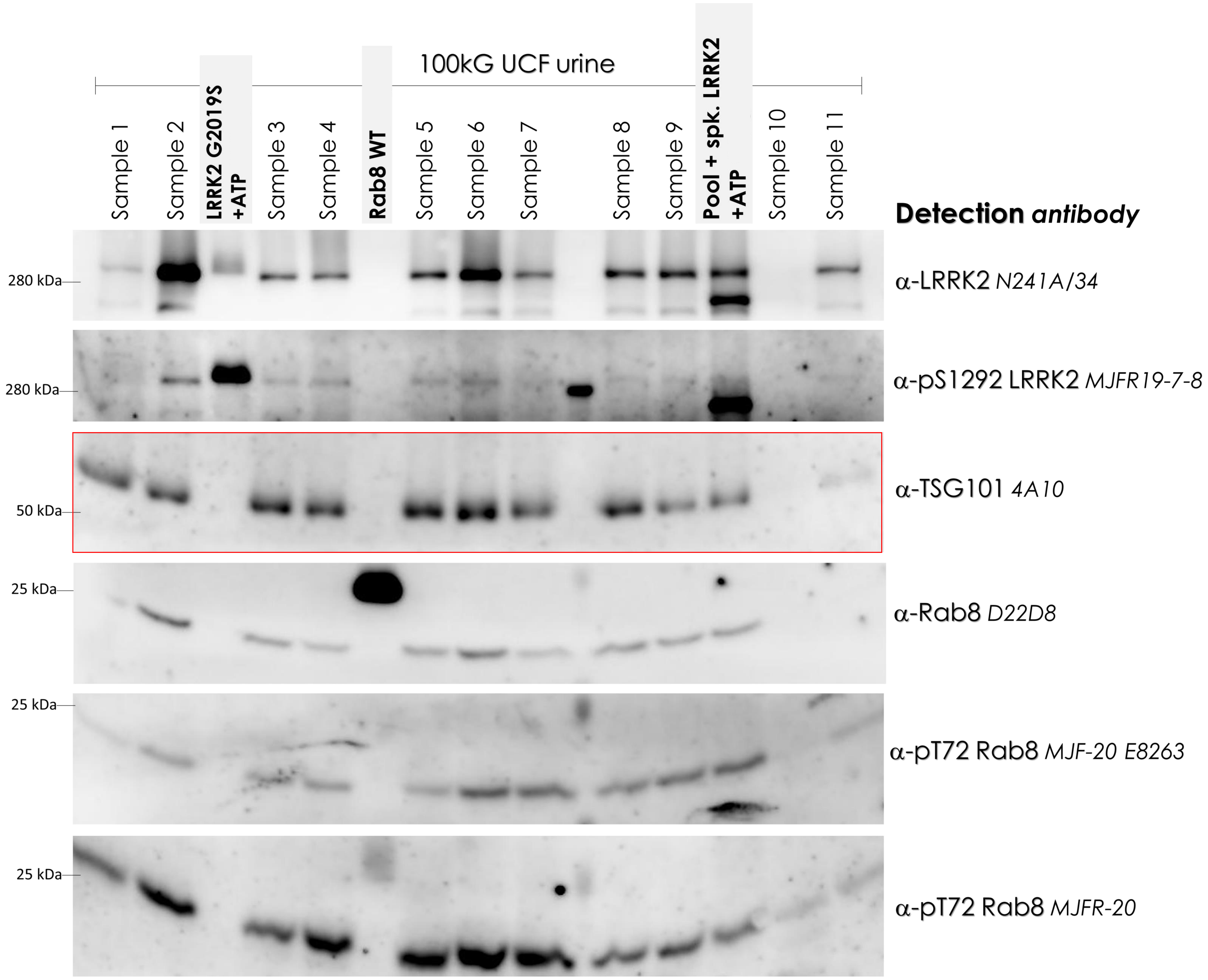

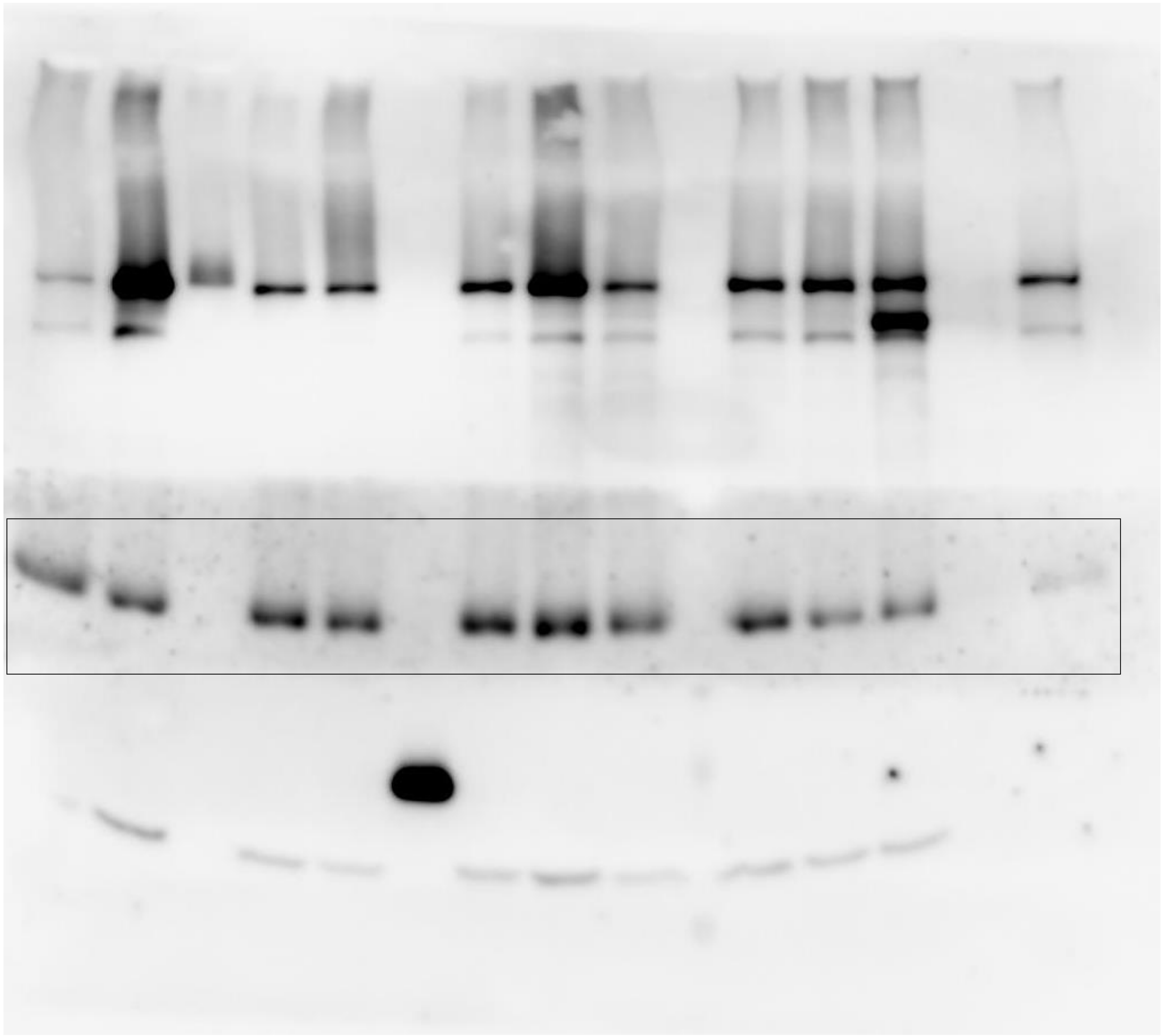

Suppl Figure 7

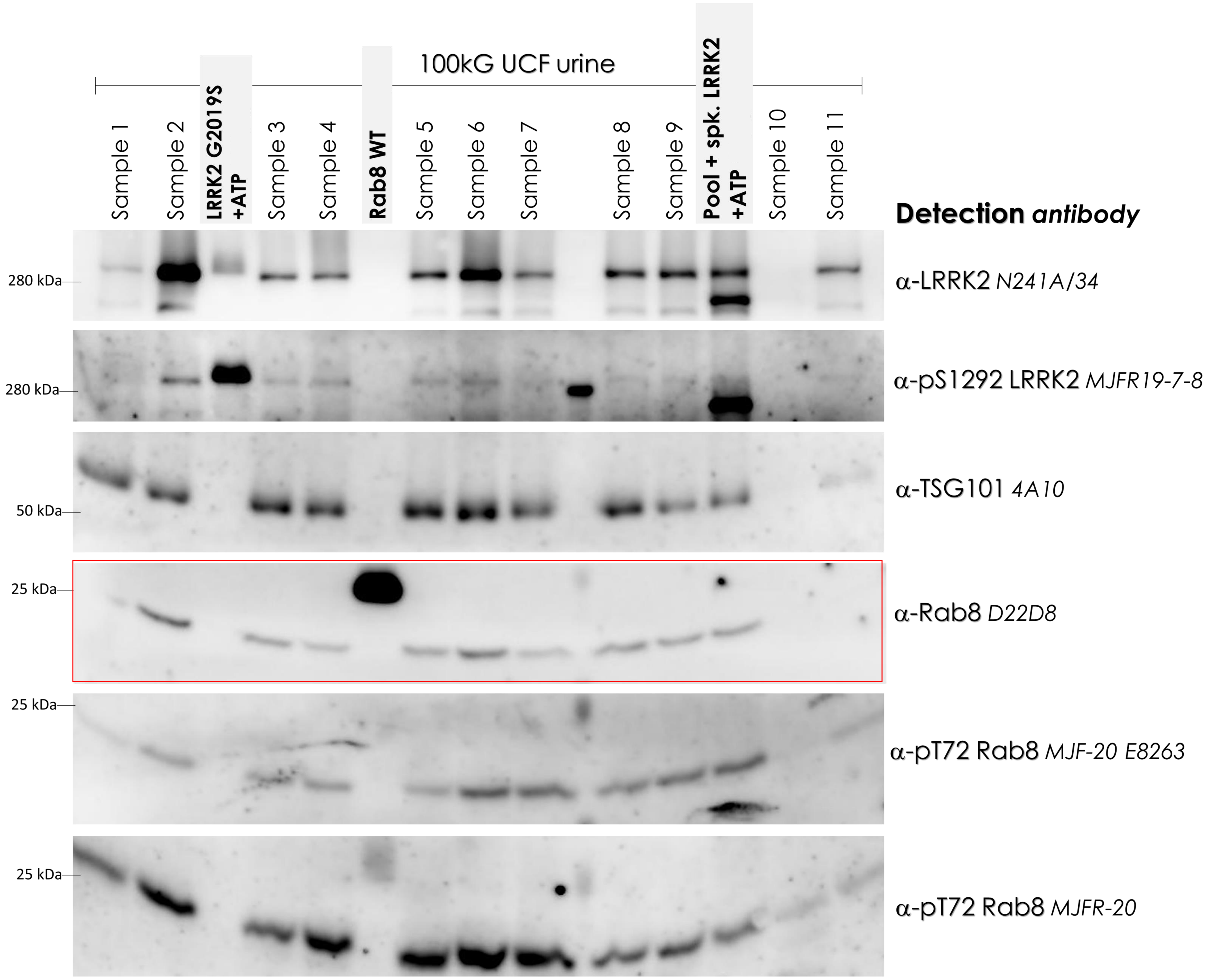

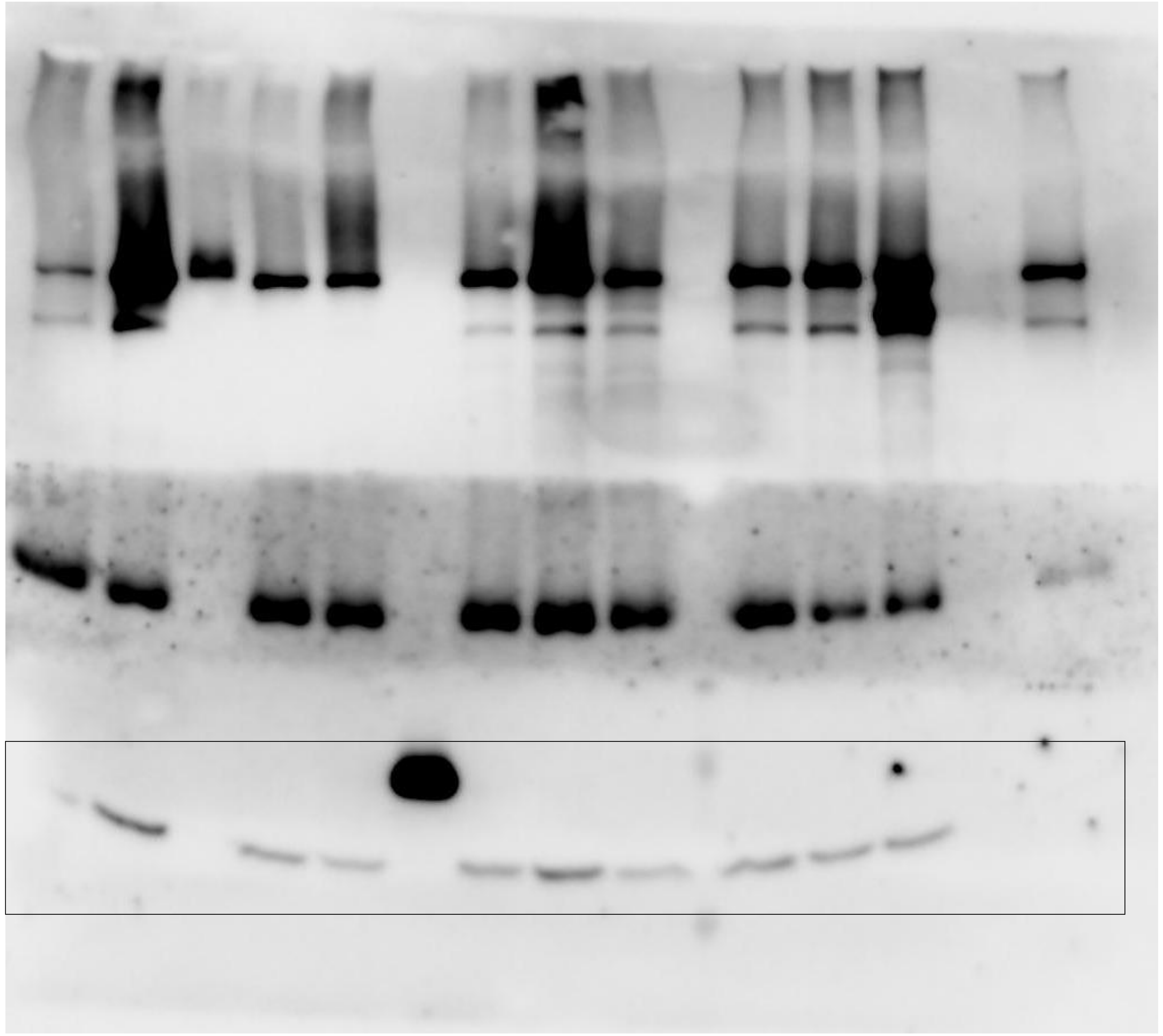

Suppl Figure 7

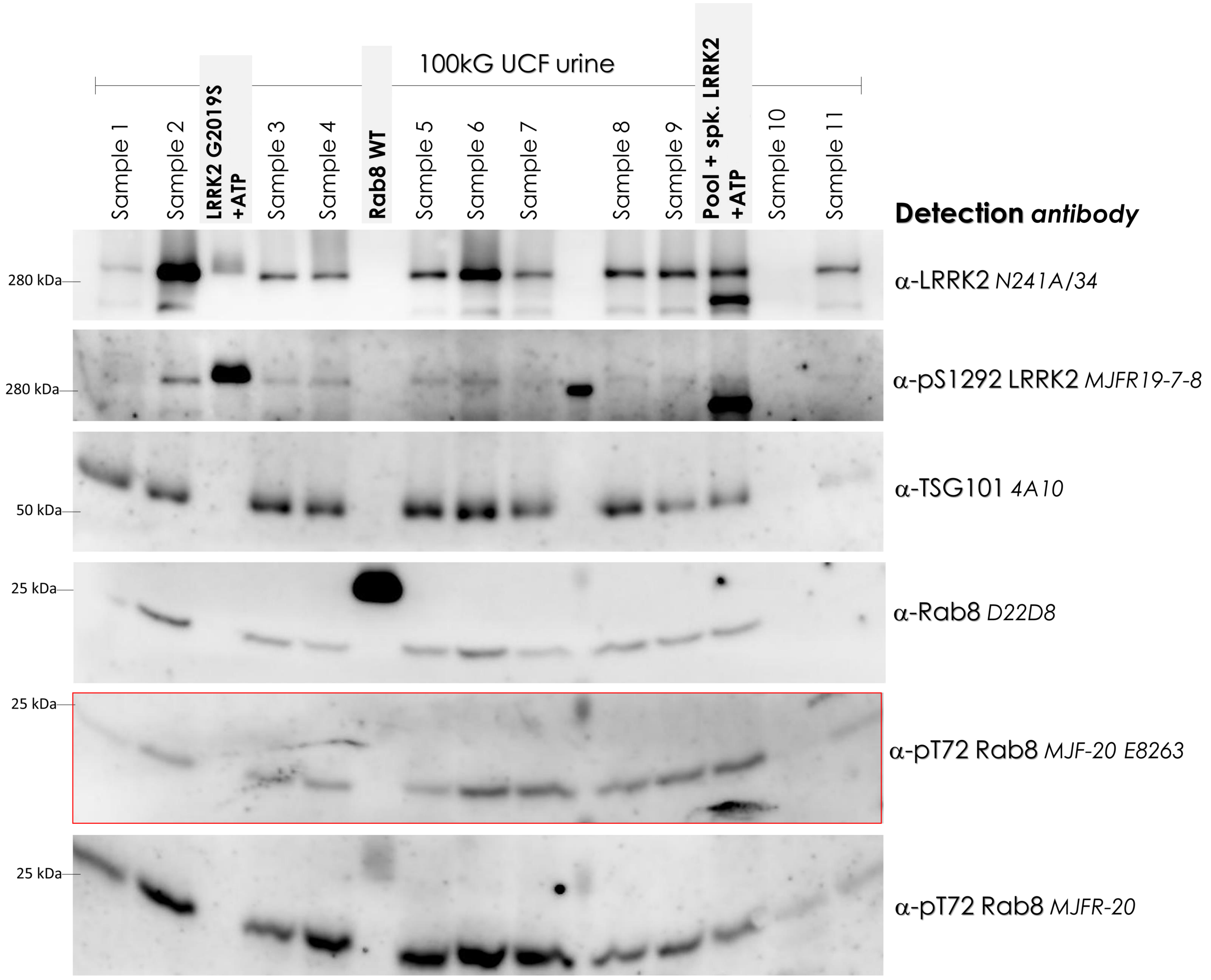

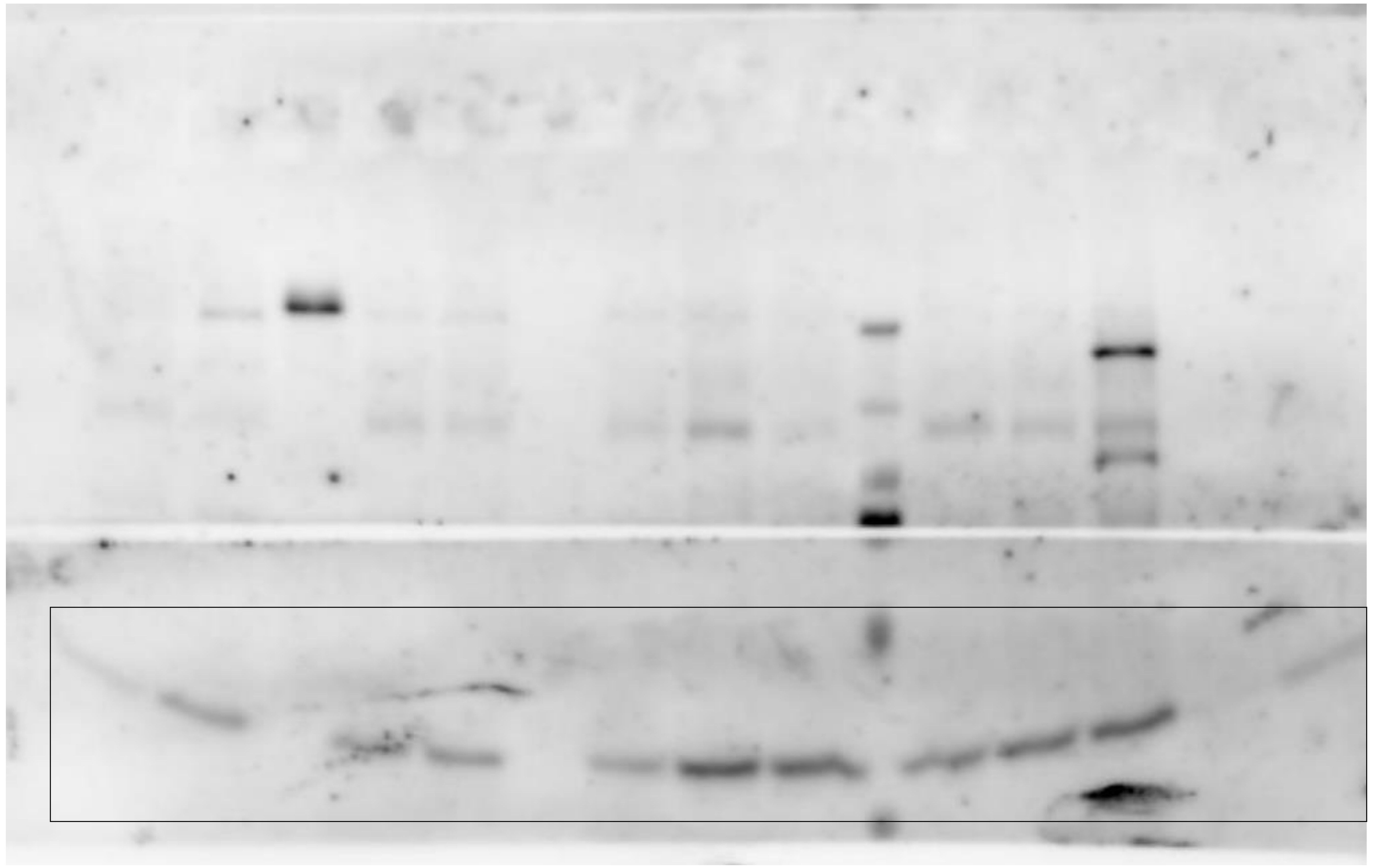

Suppl Figure 7

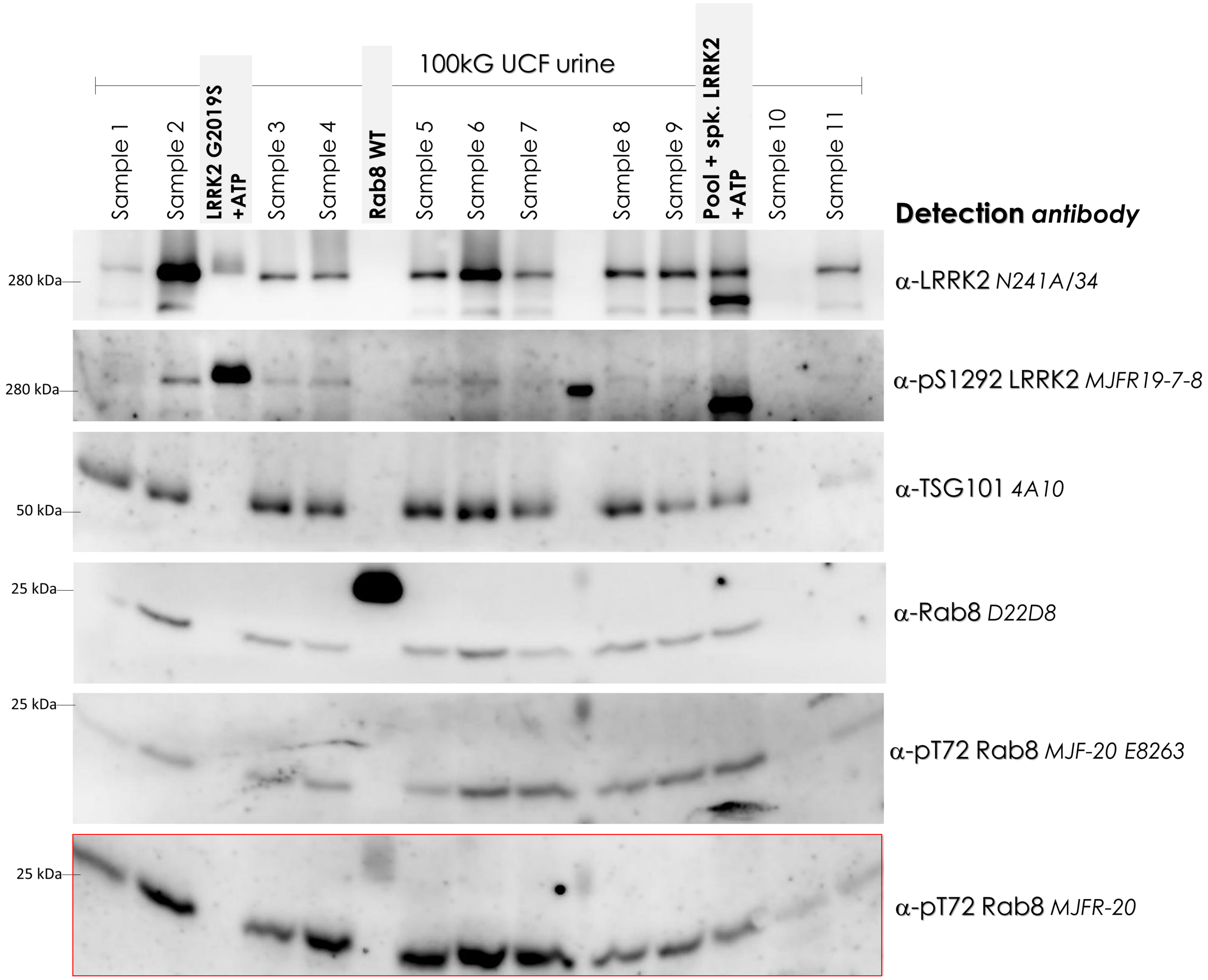

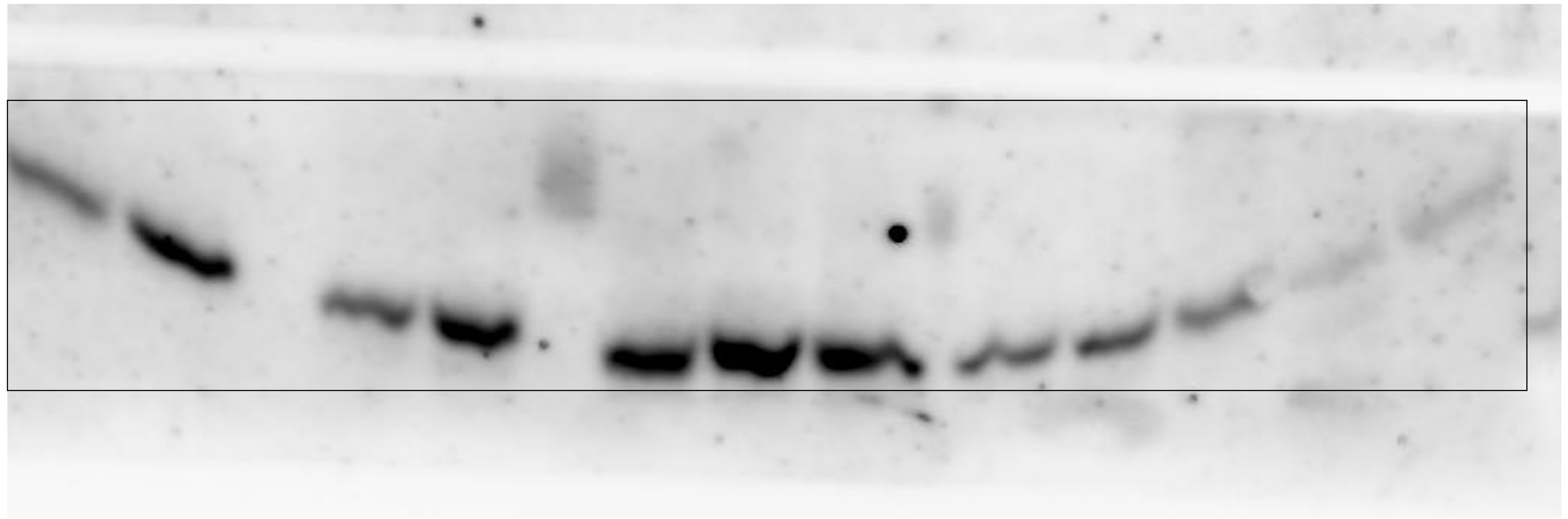

Suppl Figure 10

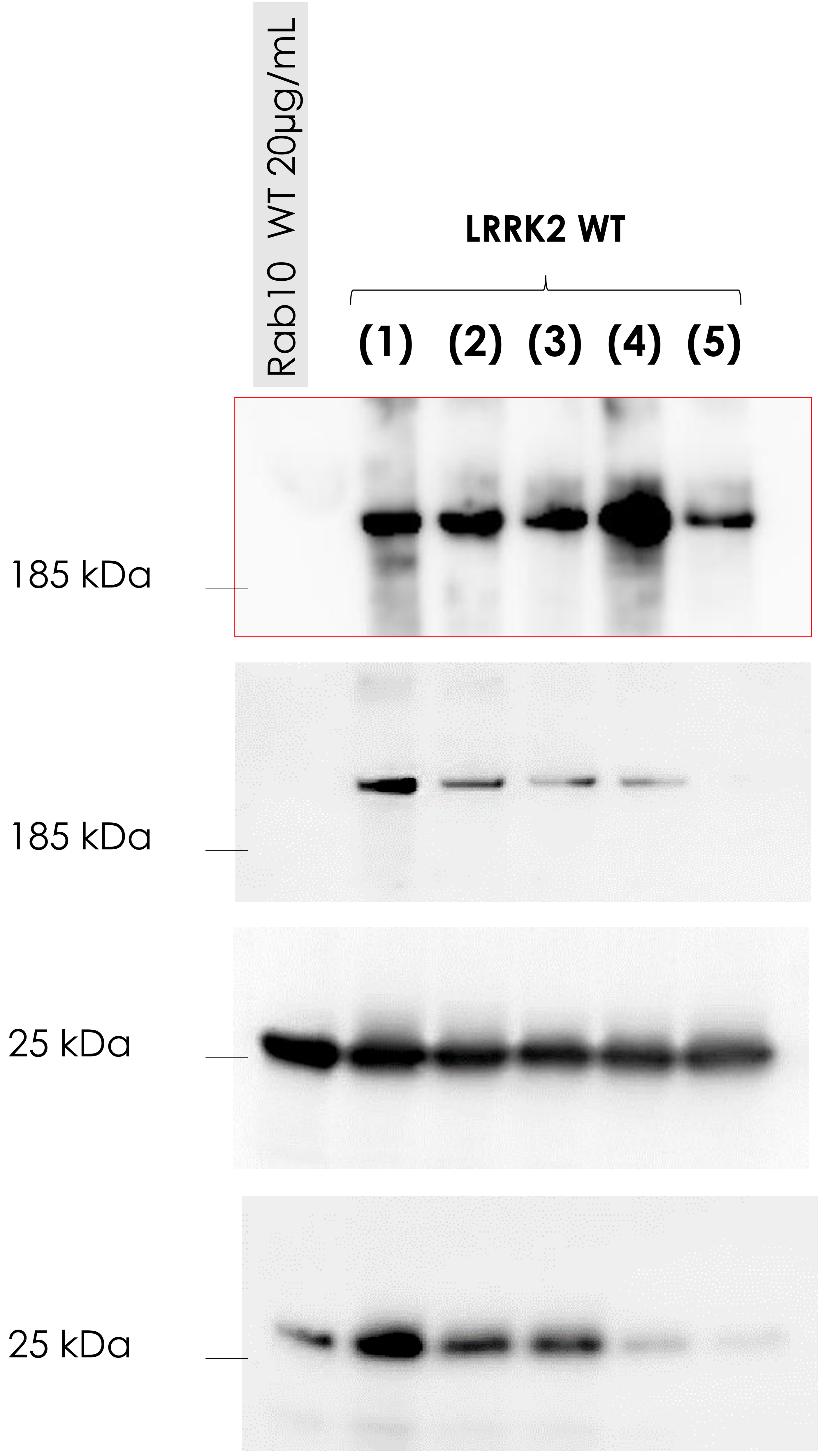

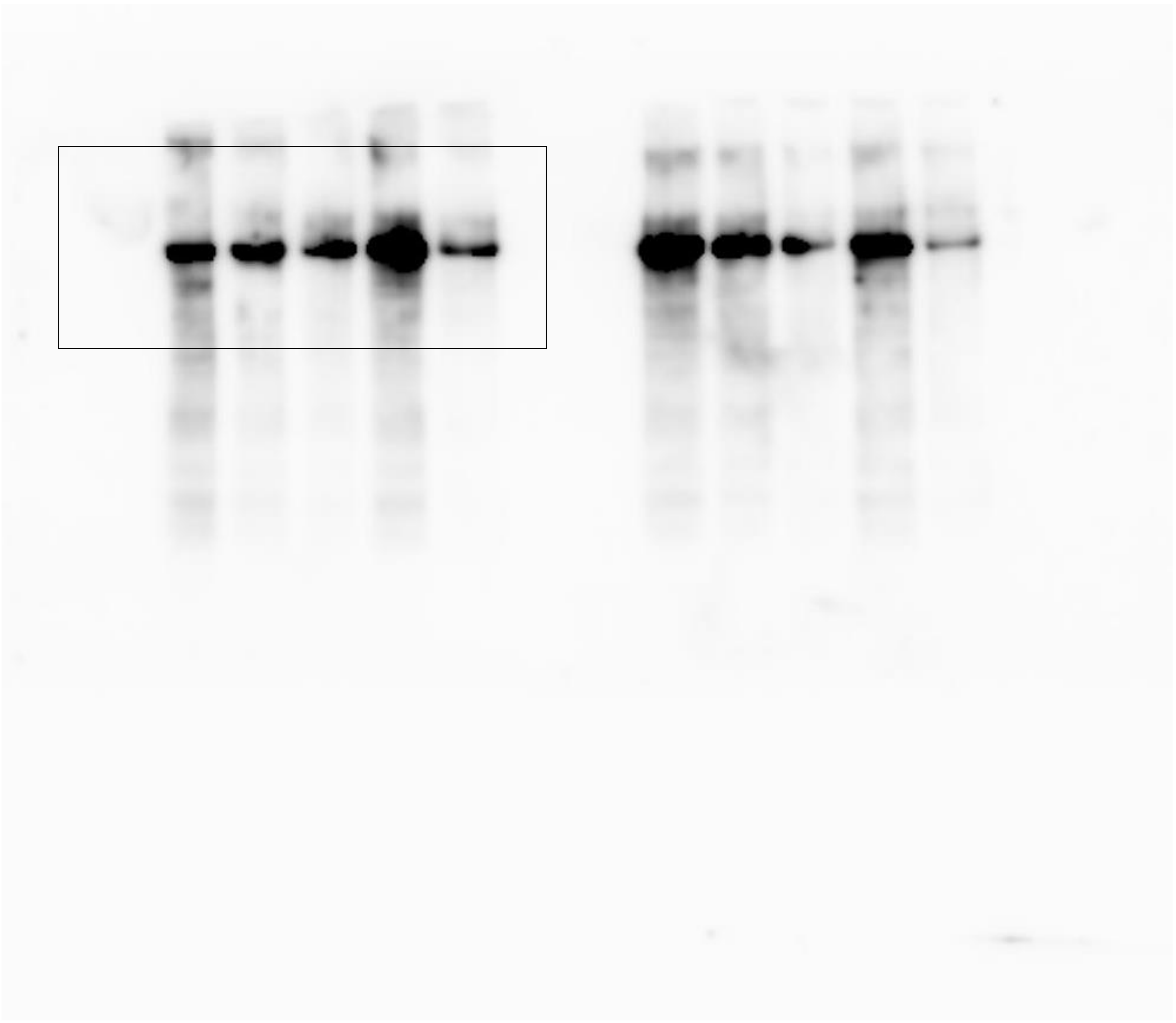

Suppl Figure 10

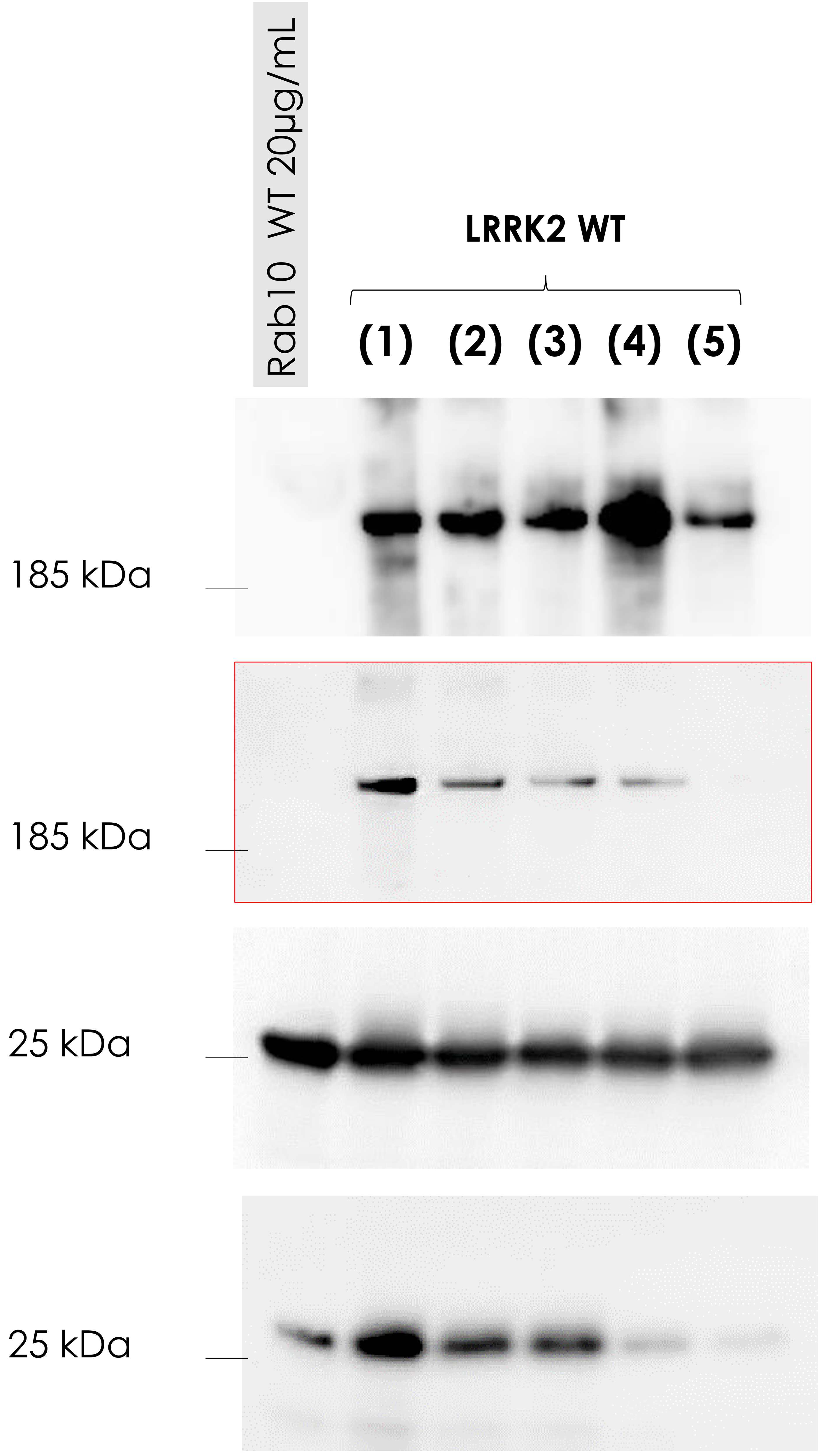

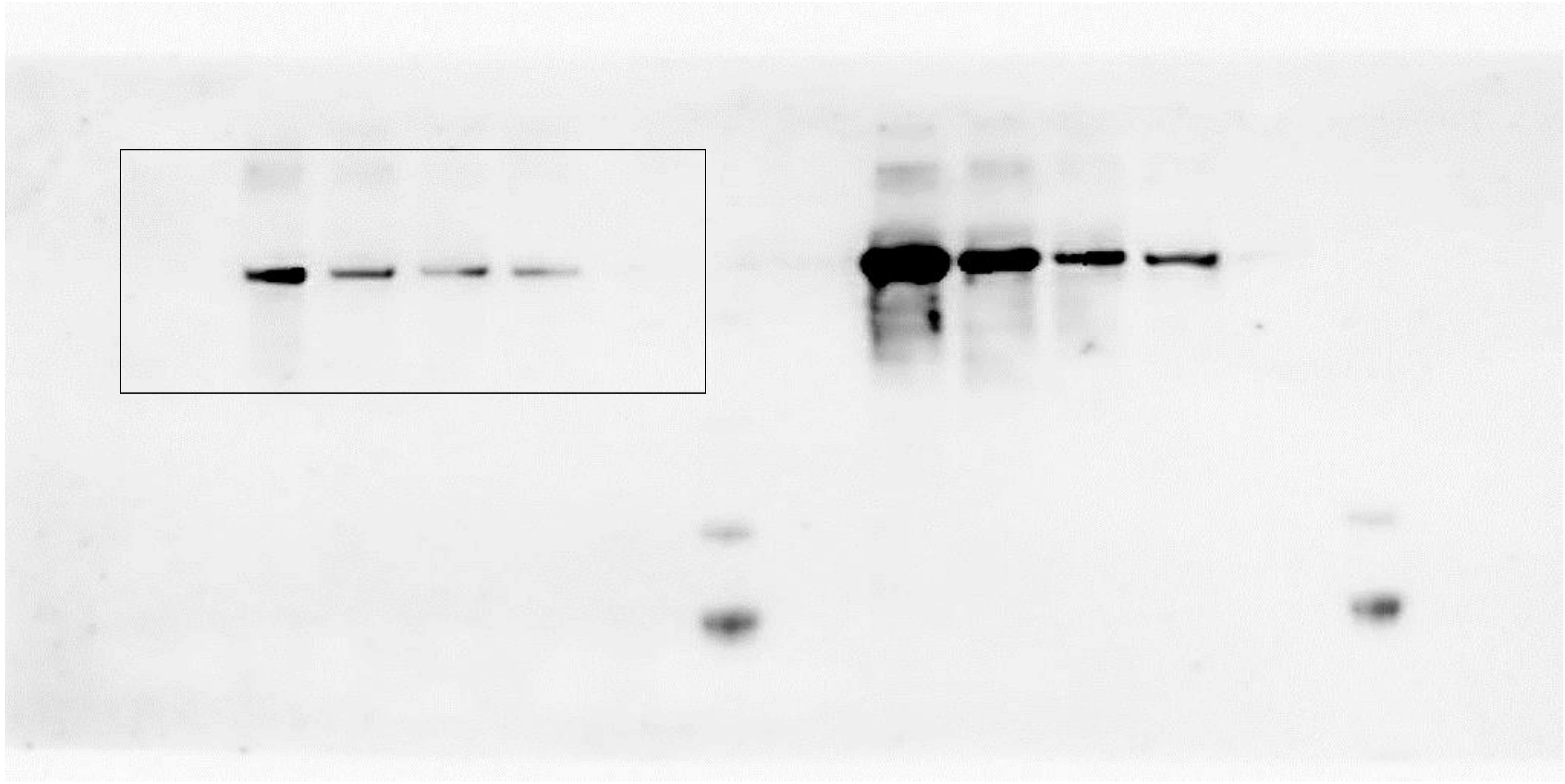

Suppl Figure 10

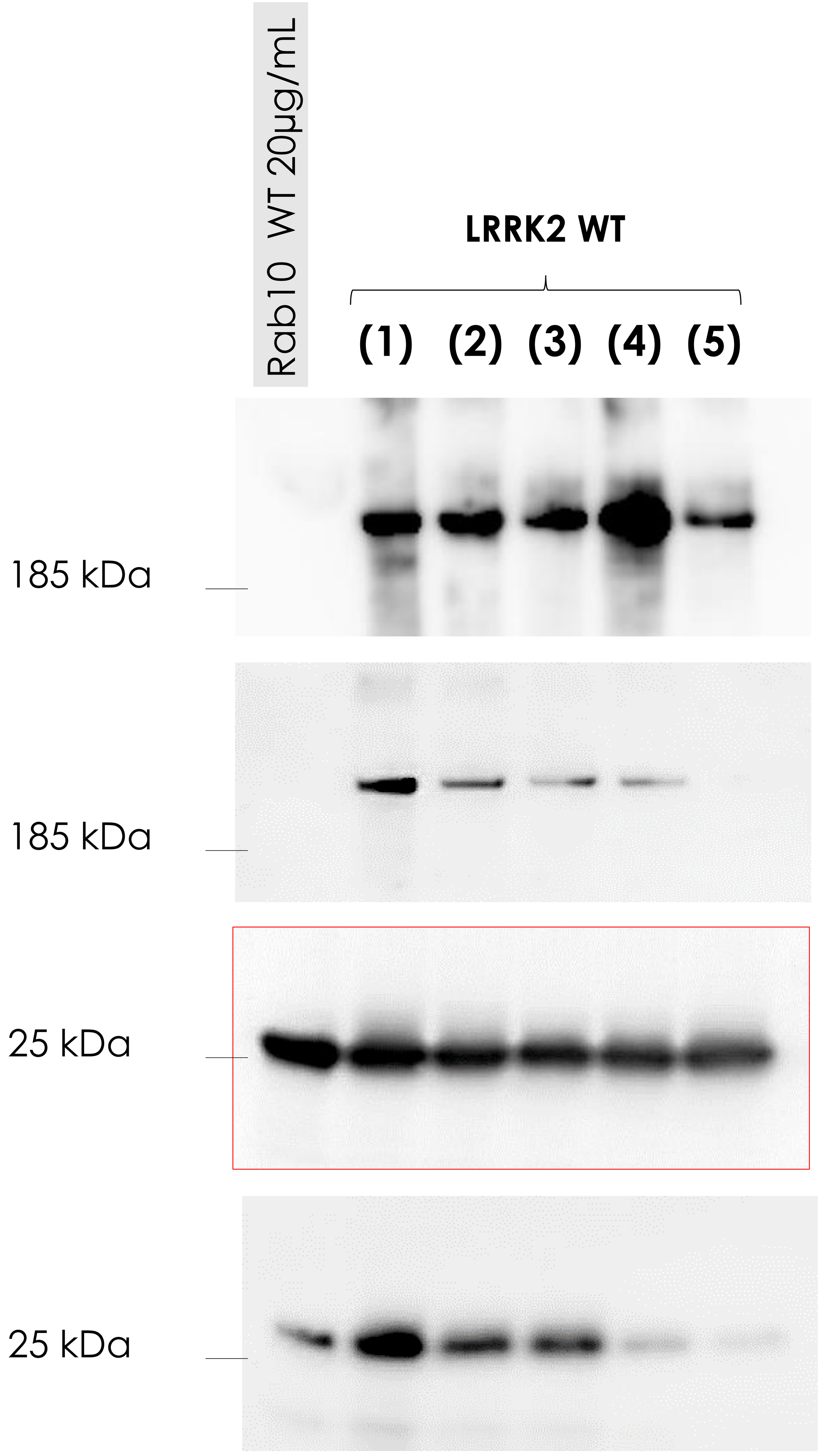

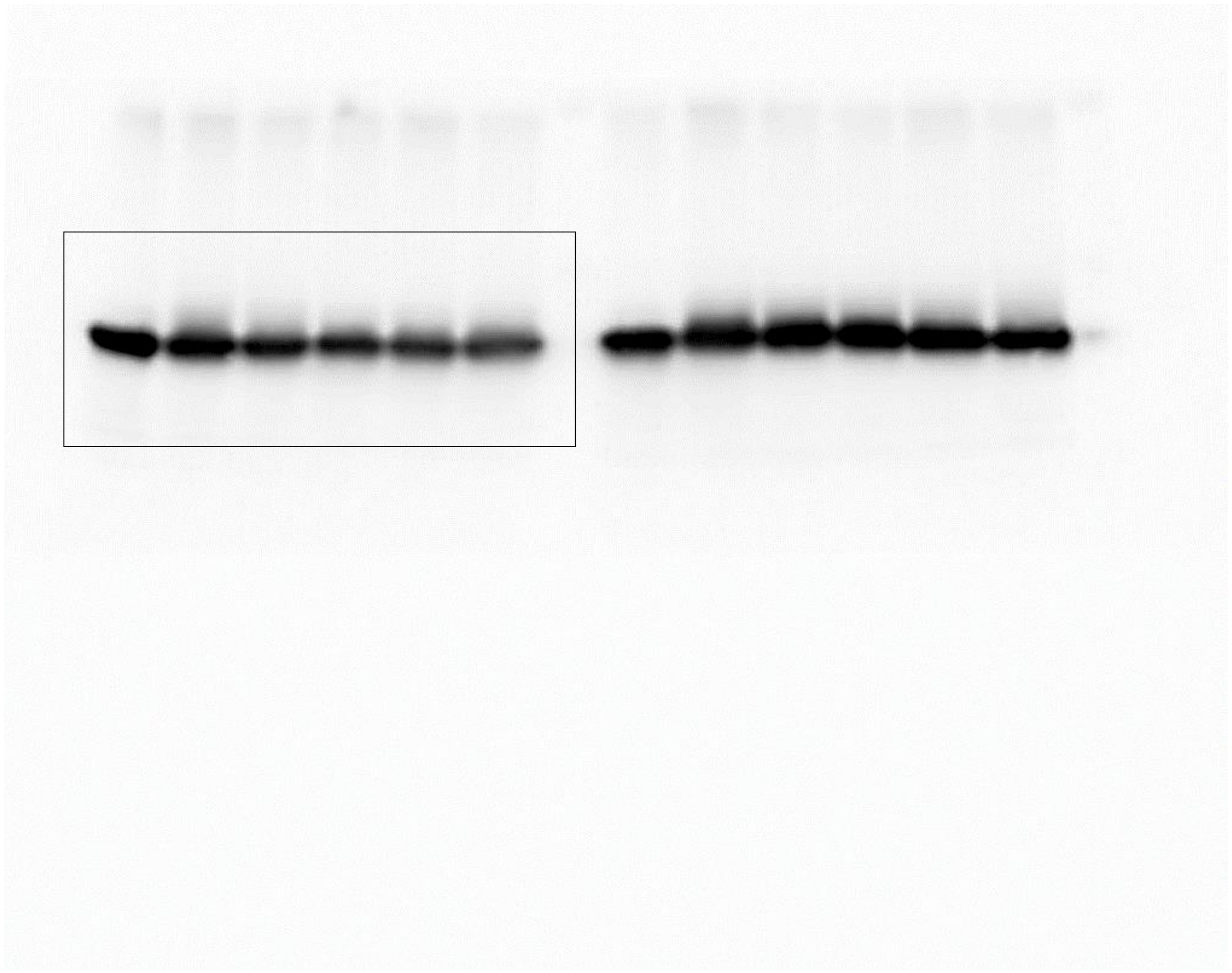

Suppl Figure 10

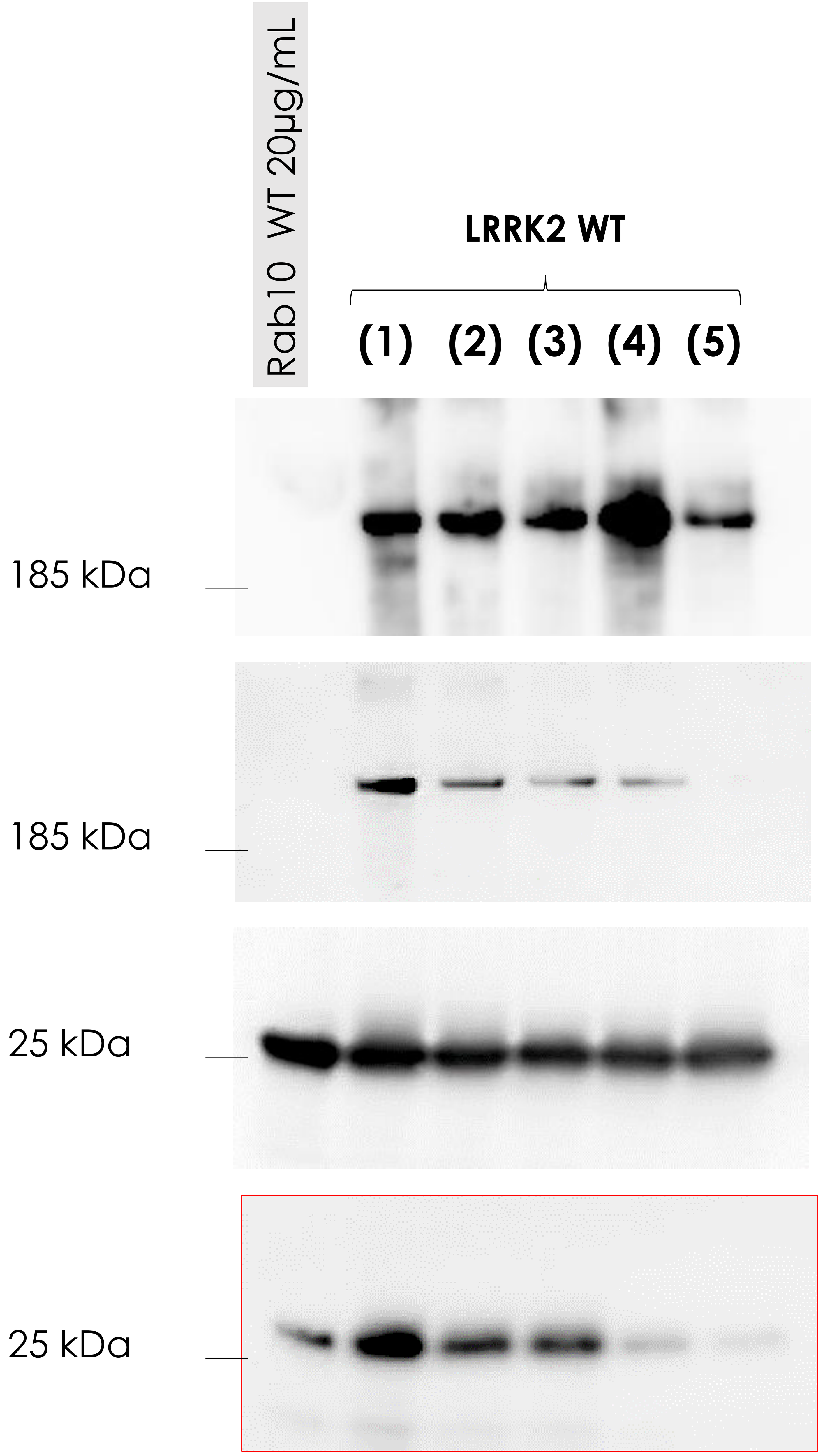

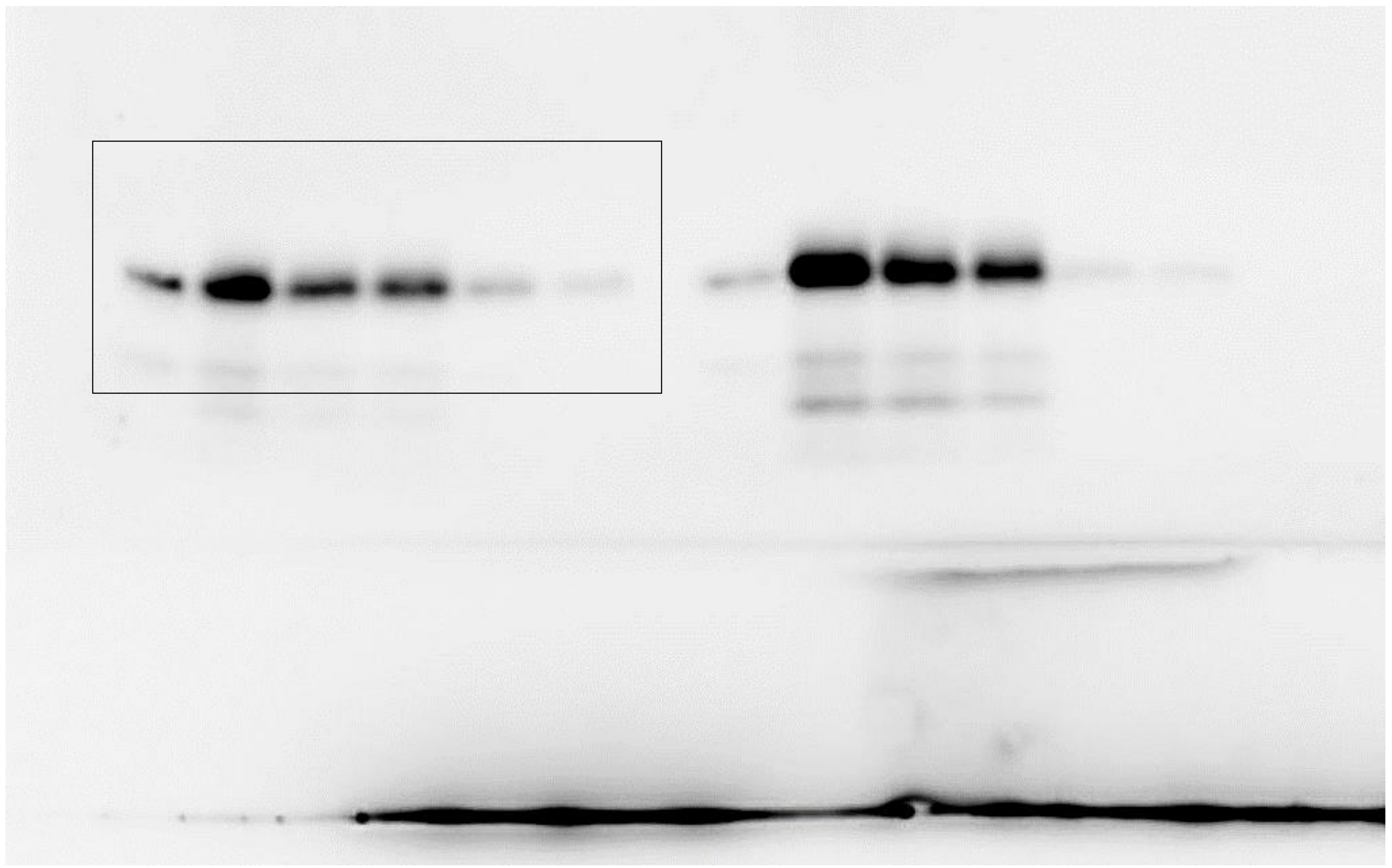

Suppl Figure 10

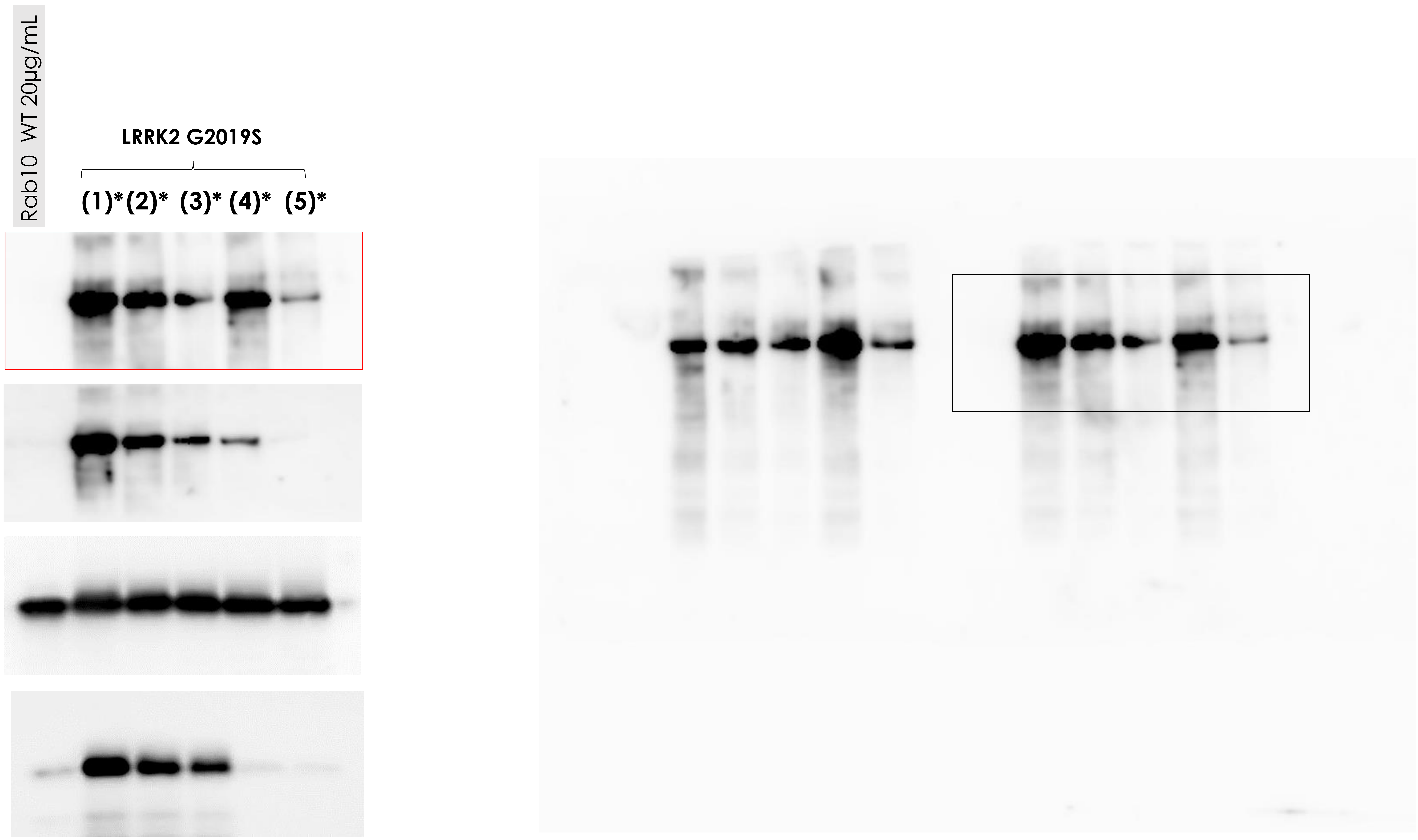

Suppl Figure 10

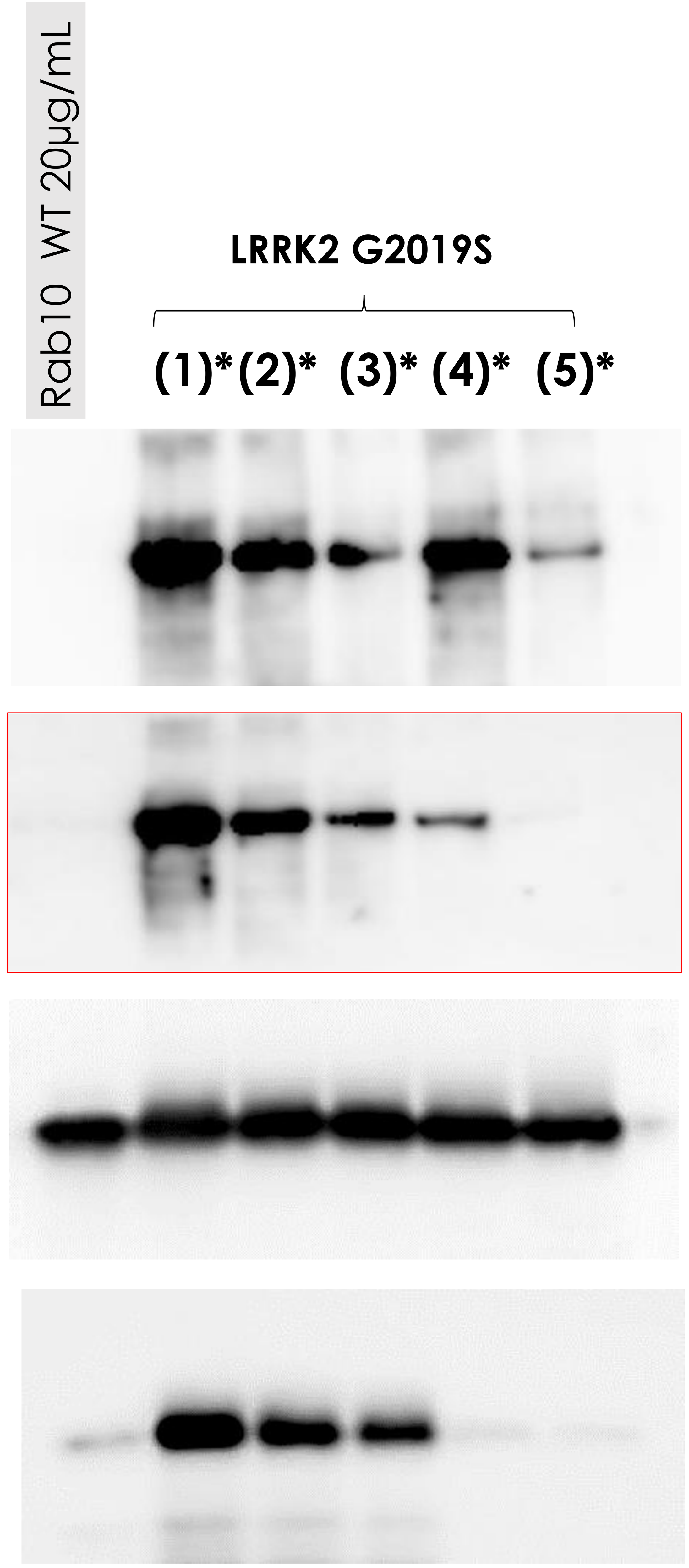

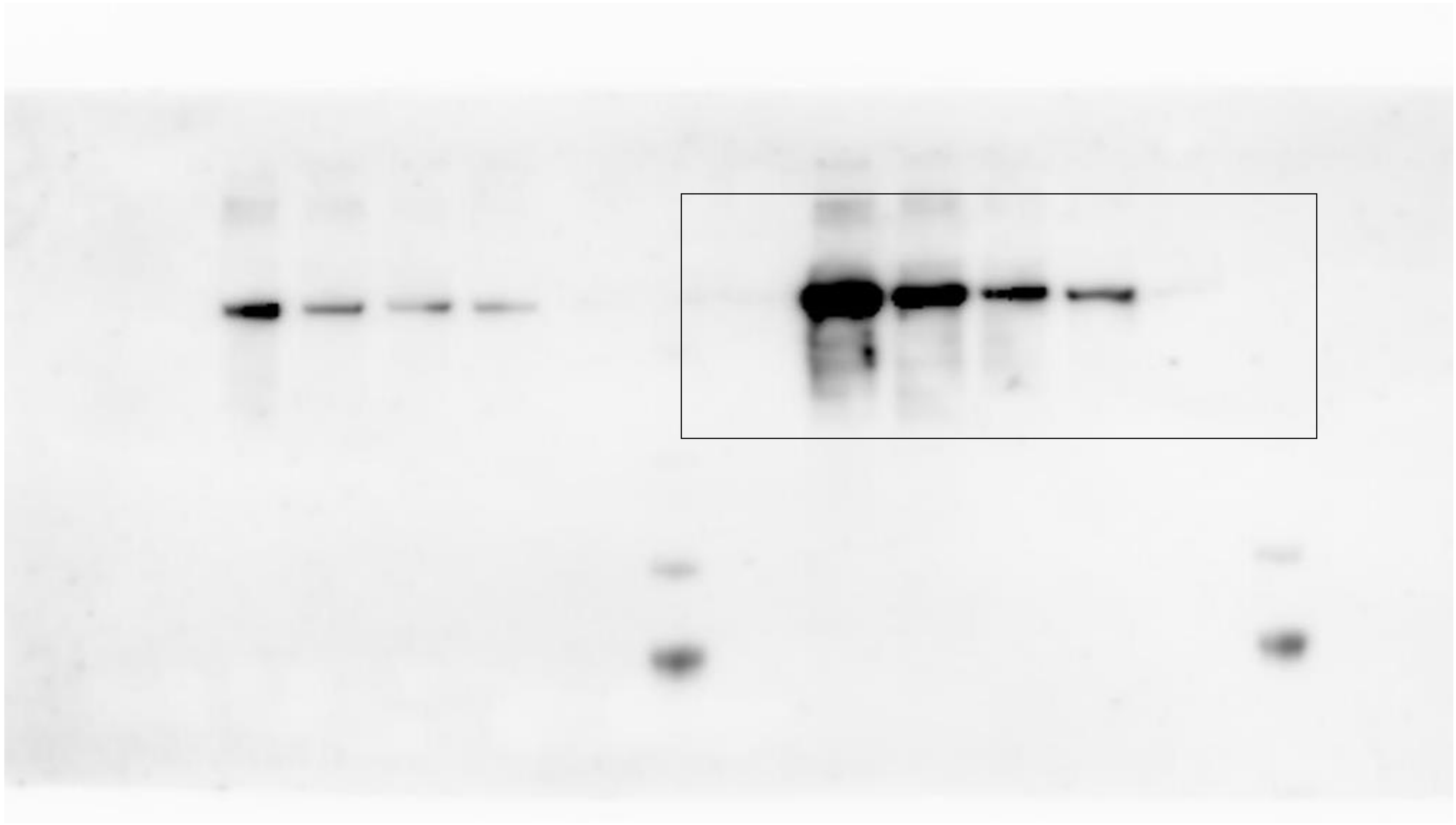

Suppl Figure 10

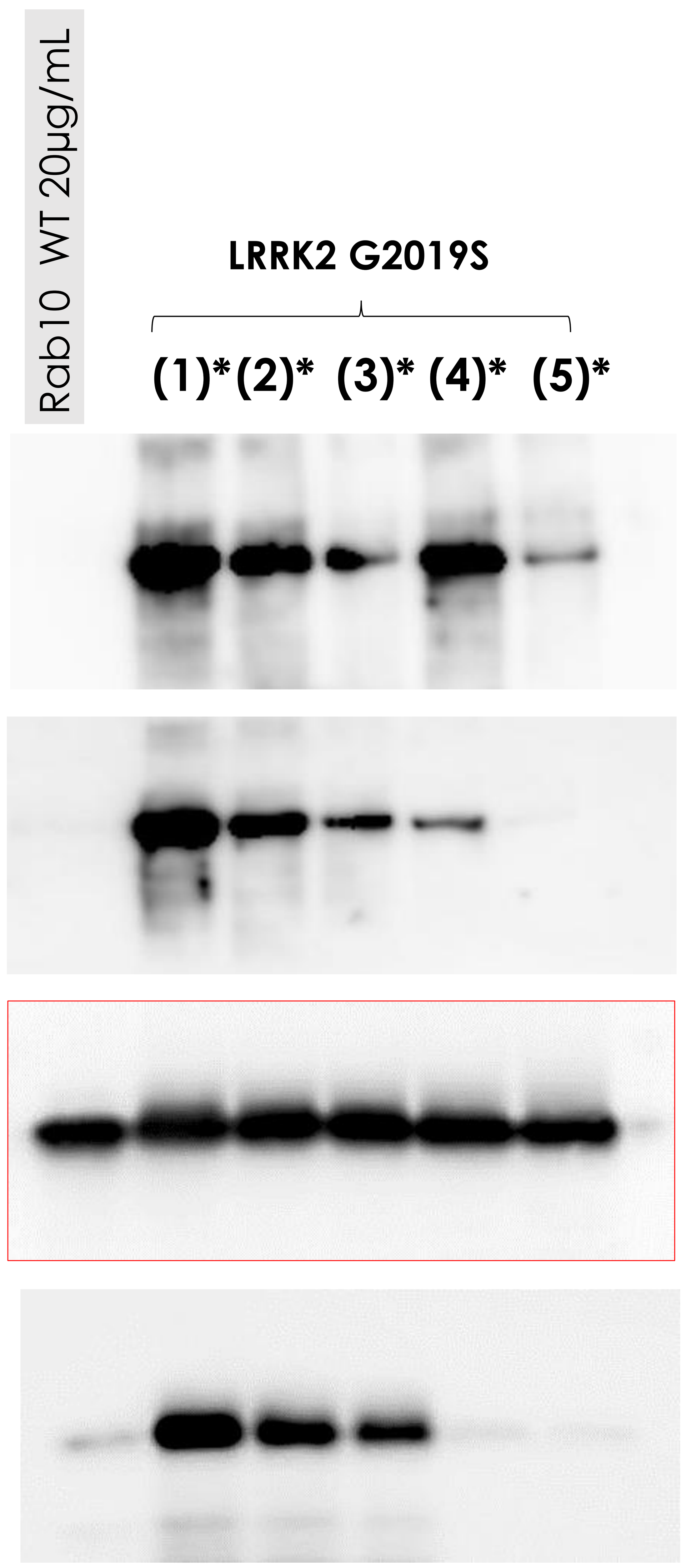

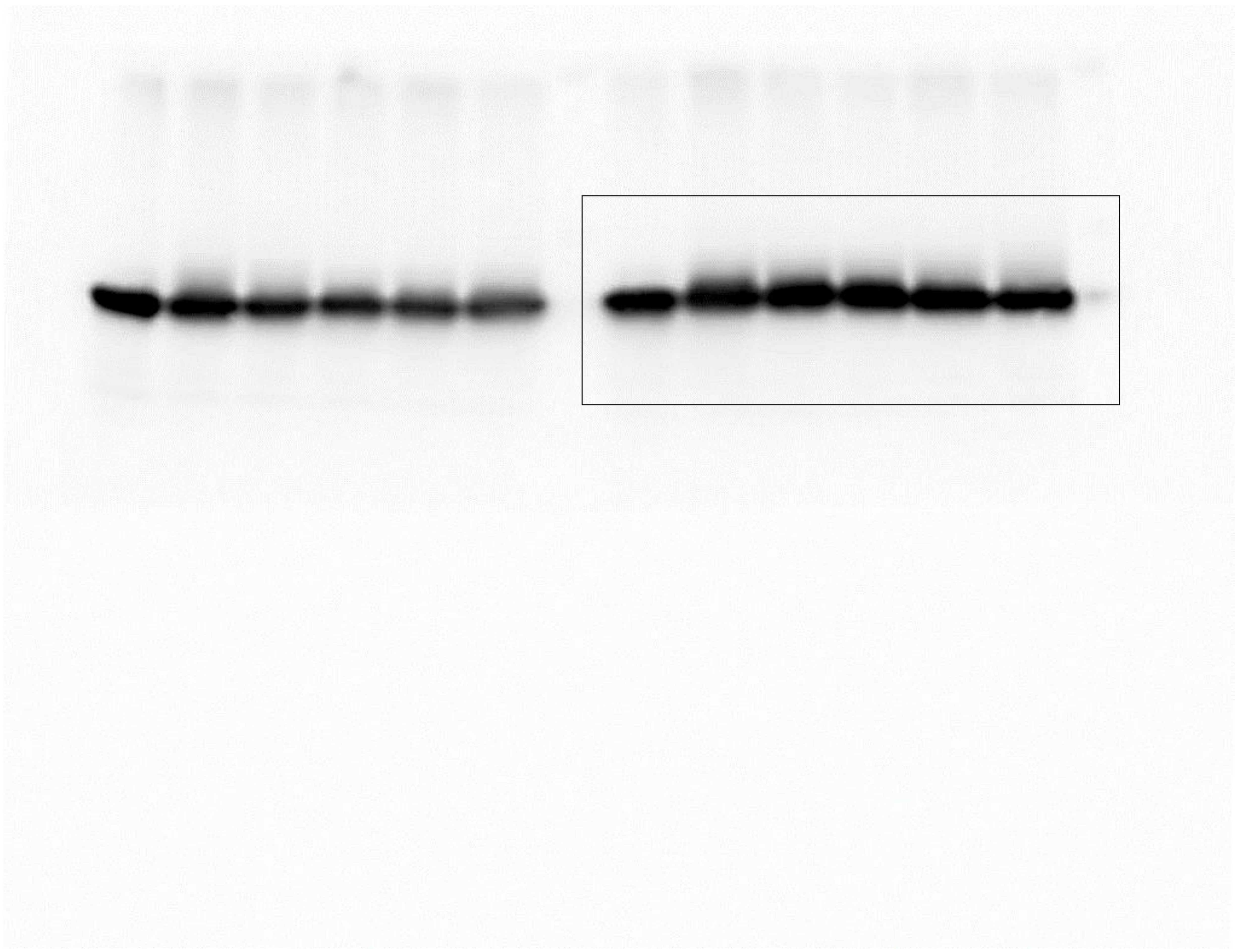

Suppl Figure 10

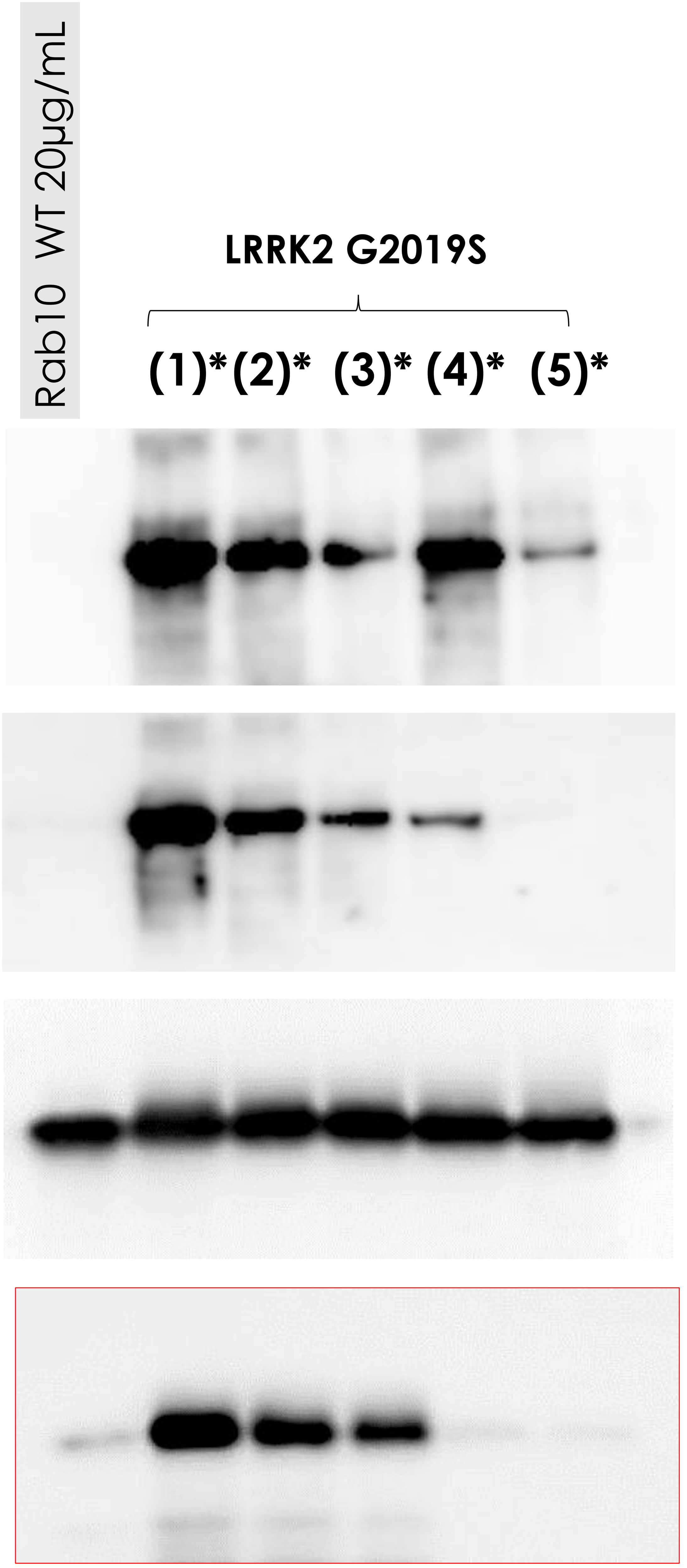

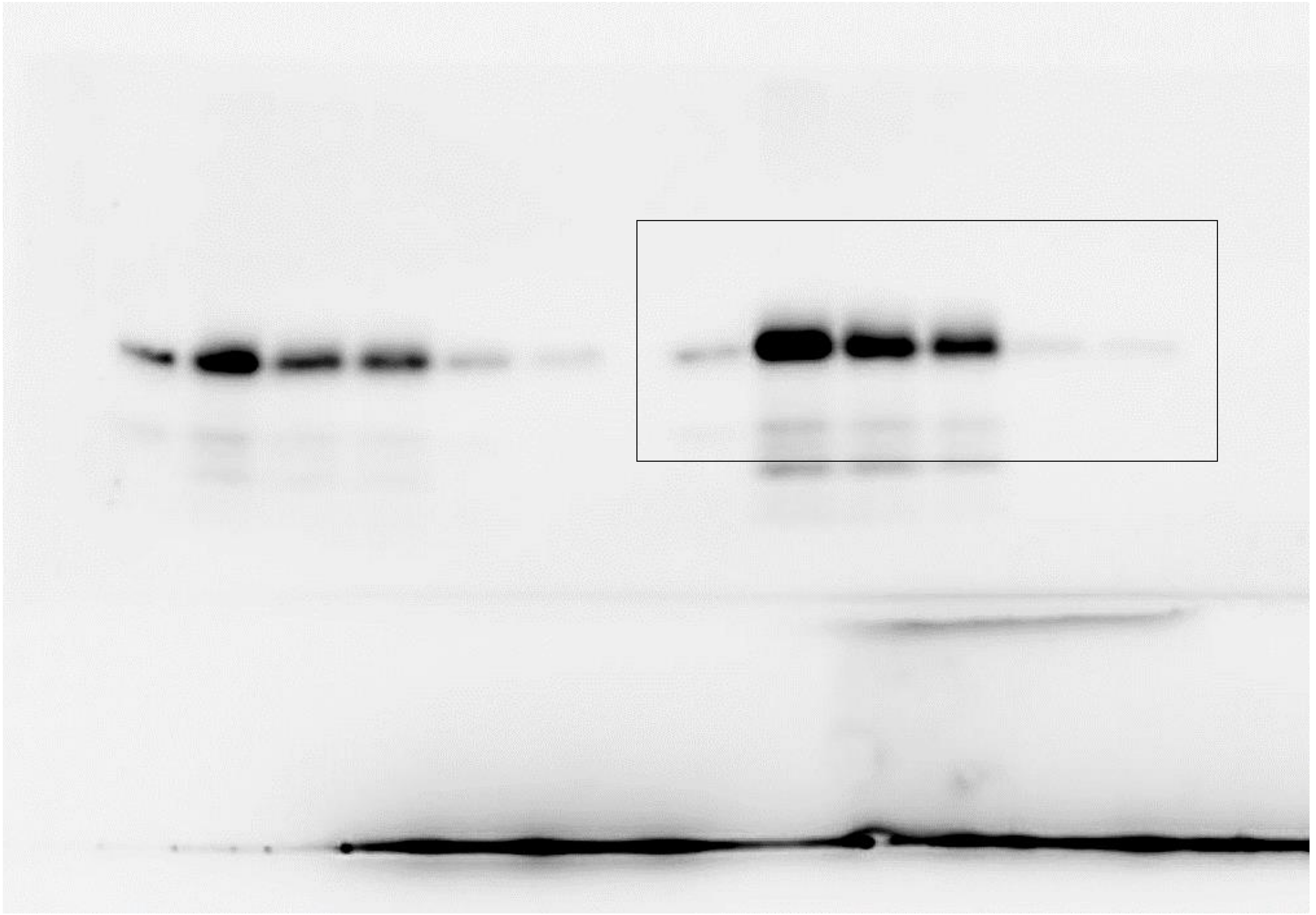

Suppl Figure S11

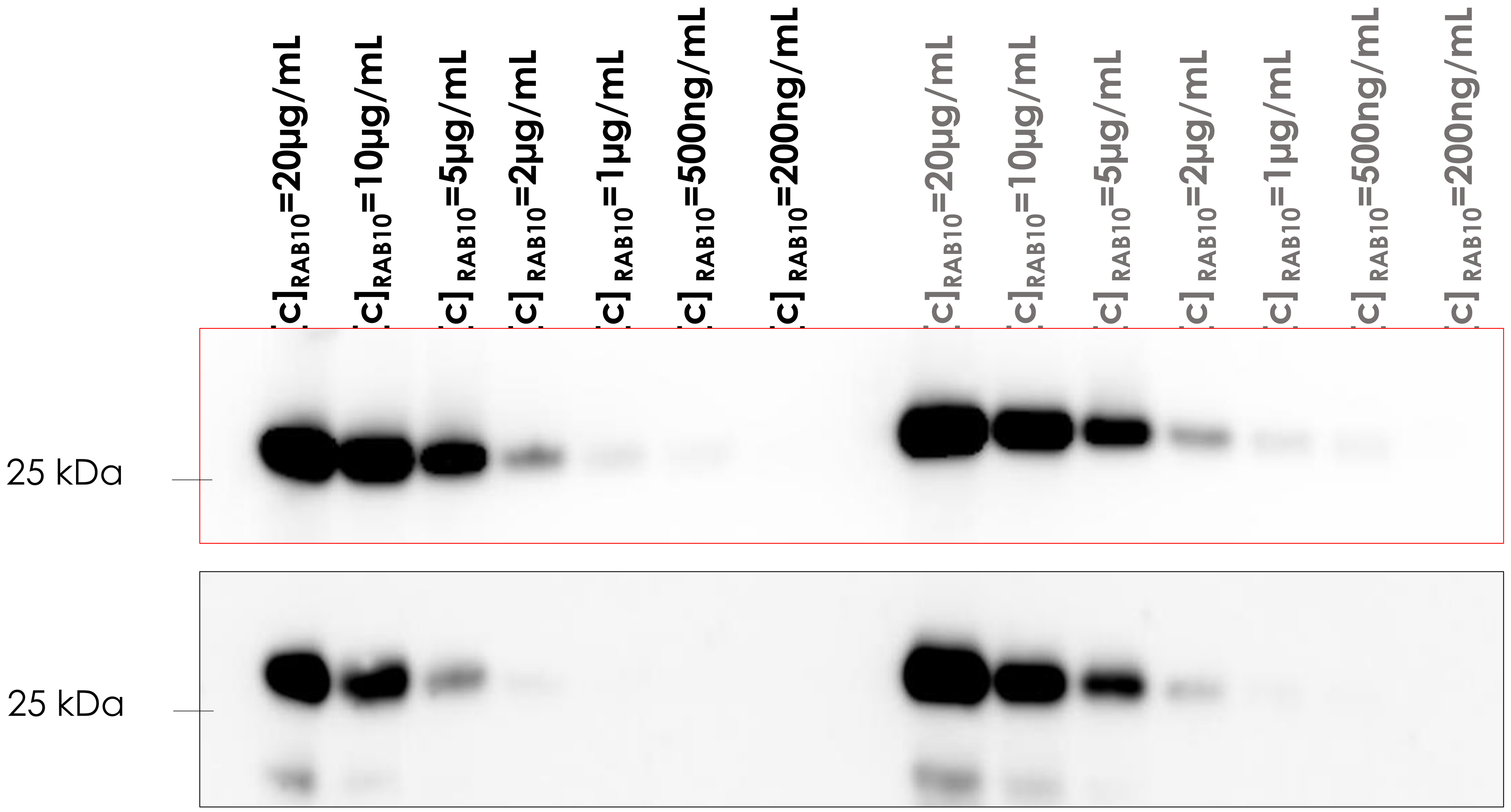

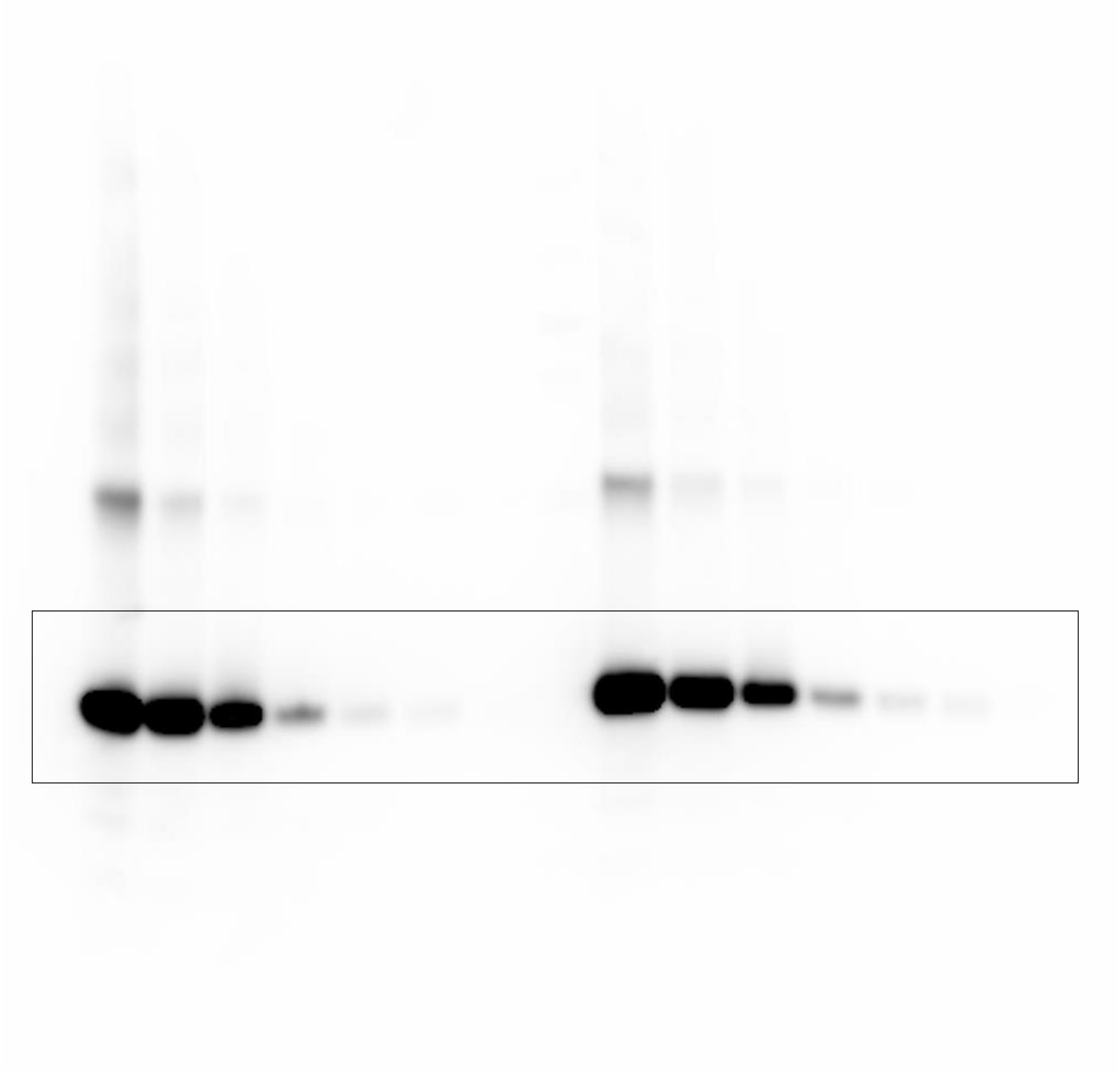

Suppl Figure S11

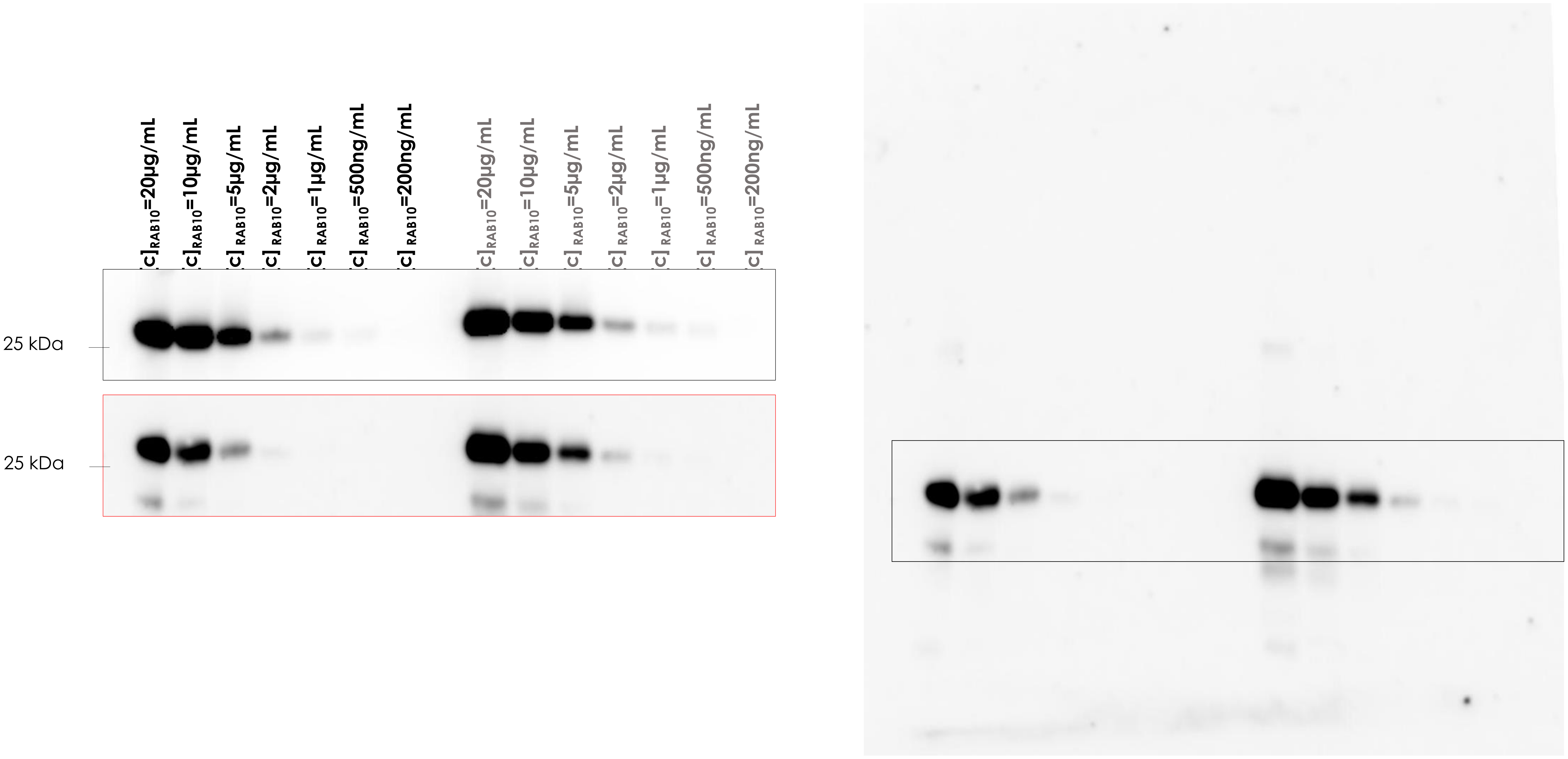

Suppl Figure 13a

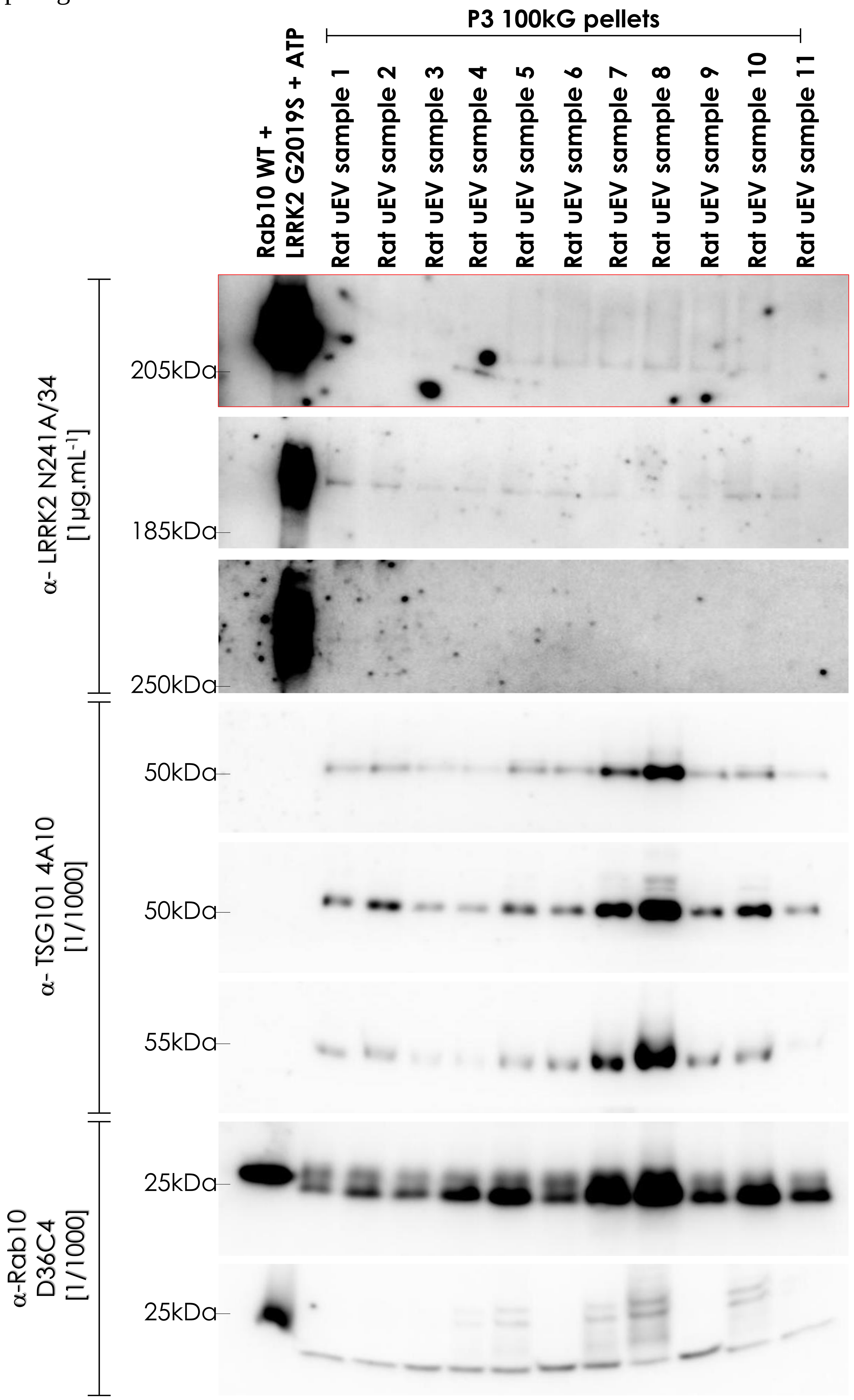

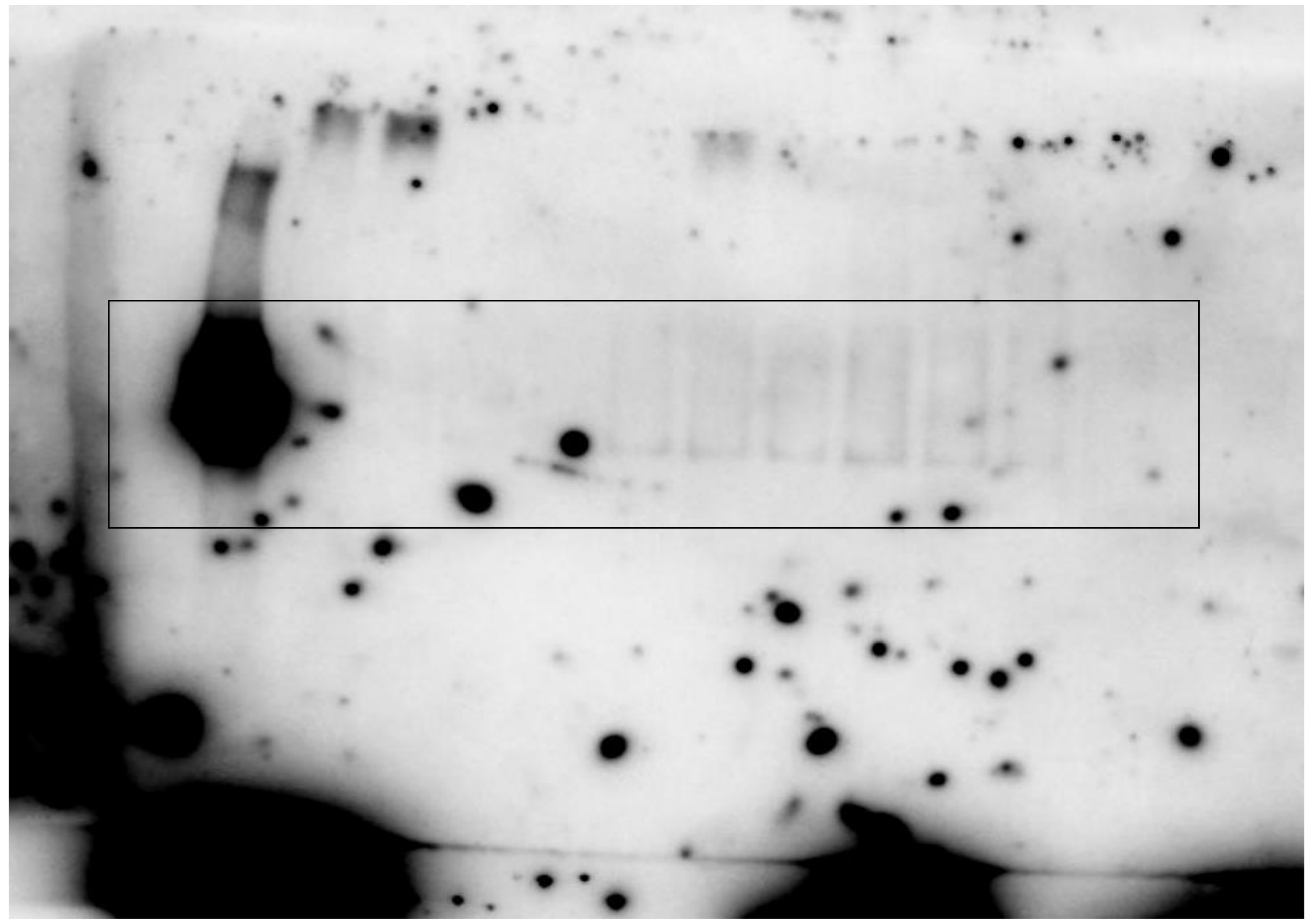

Suppl Figure 13a

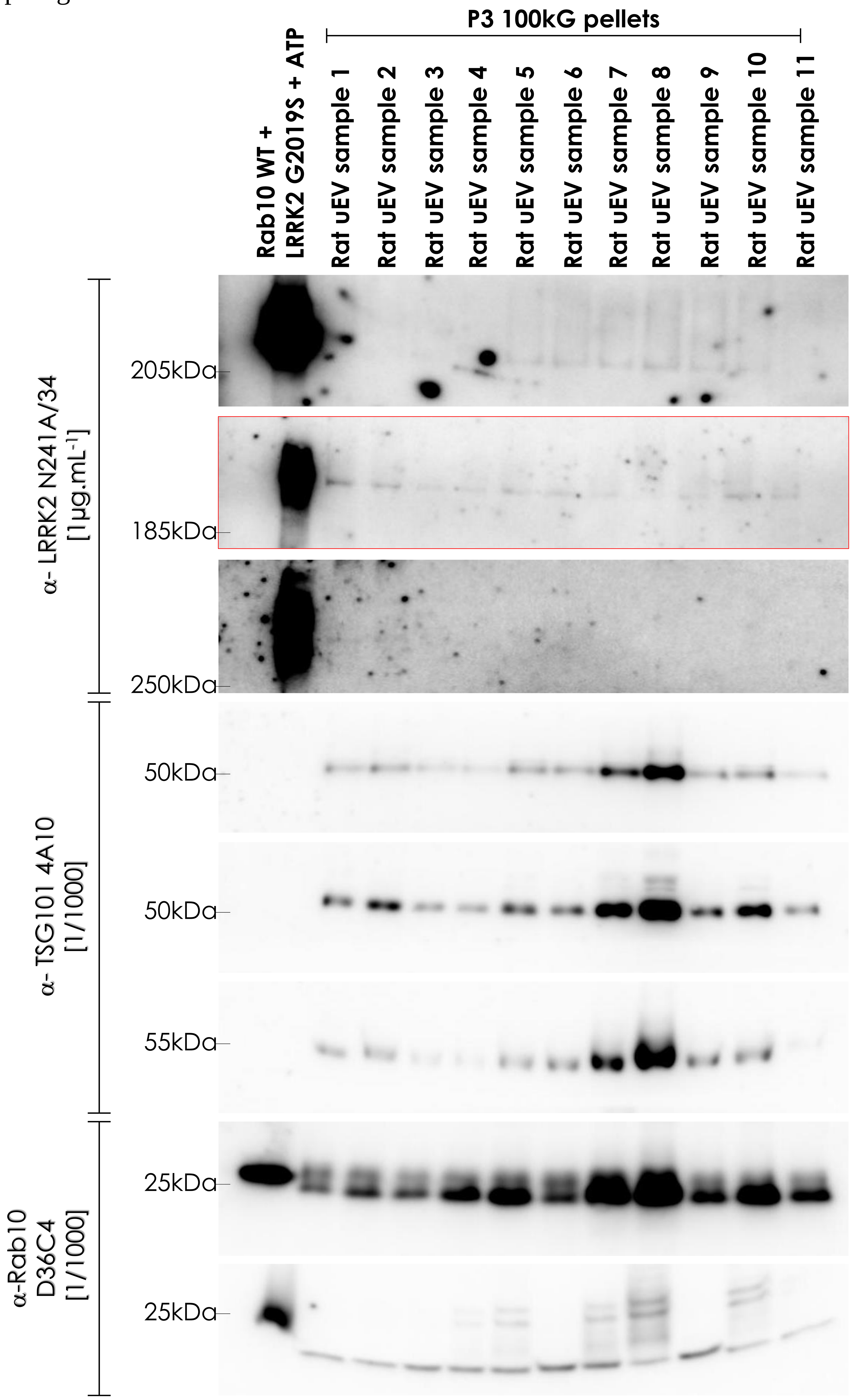

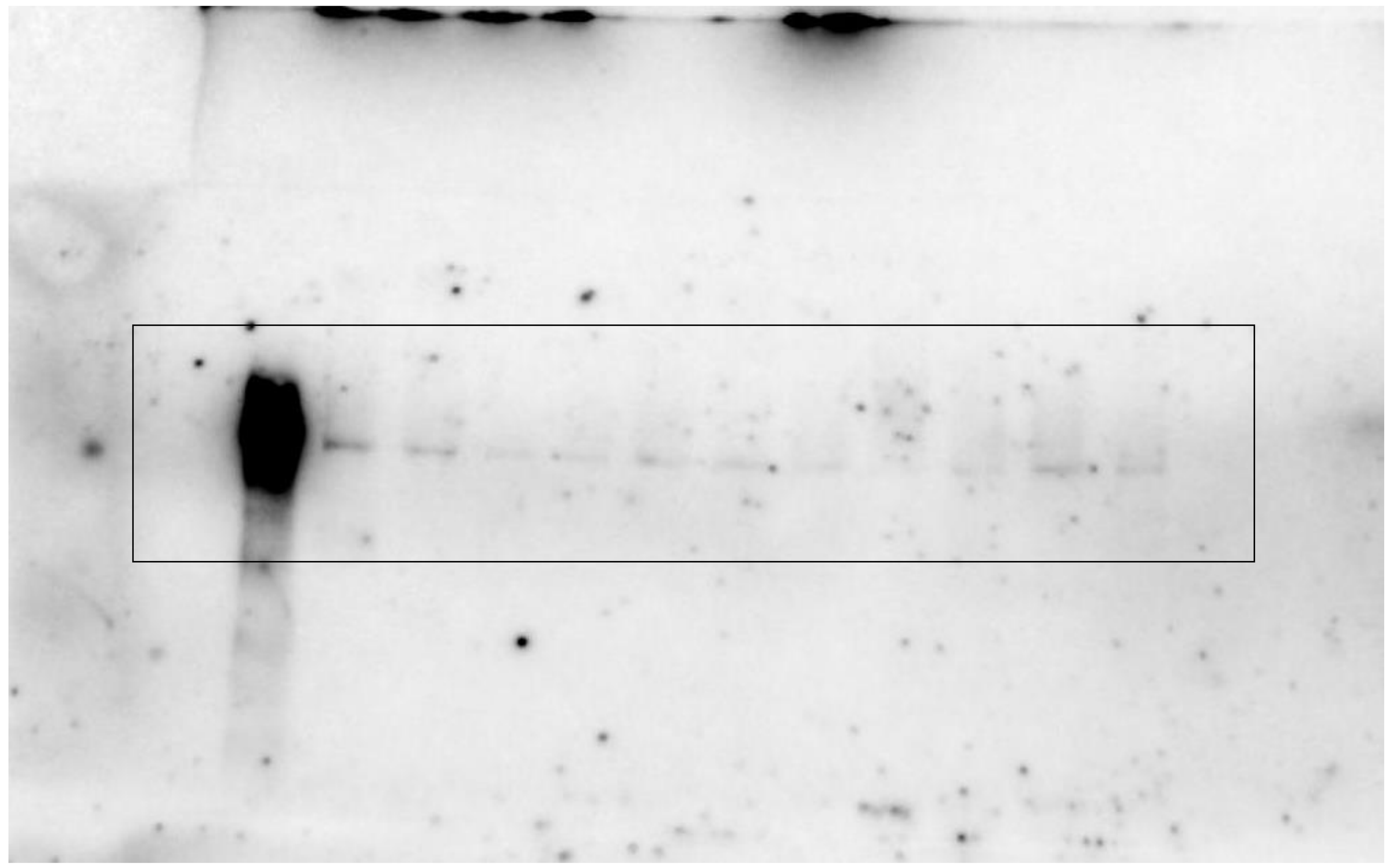

Suppl Figure 13a

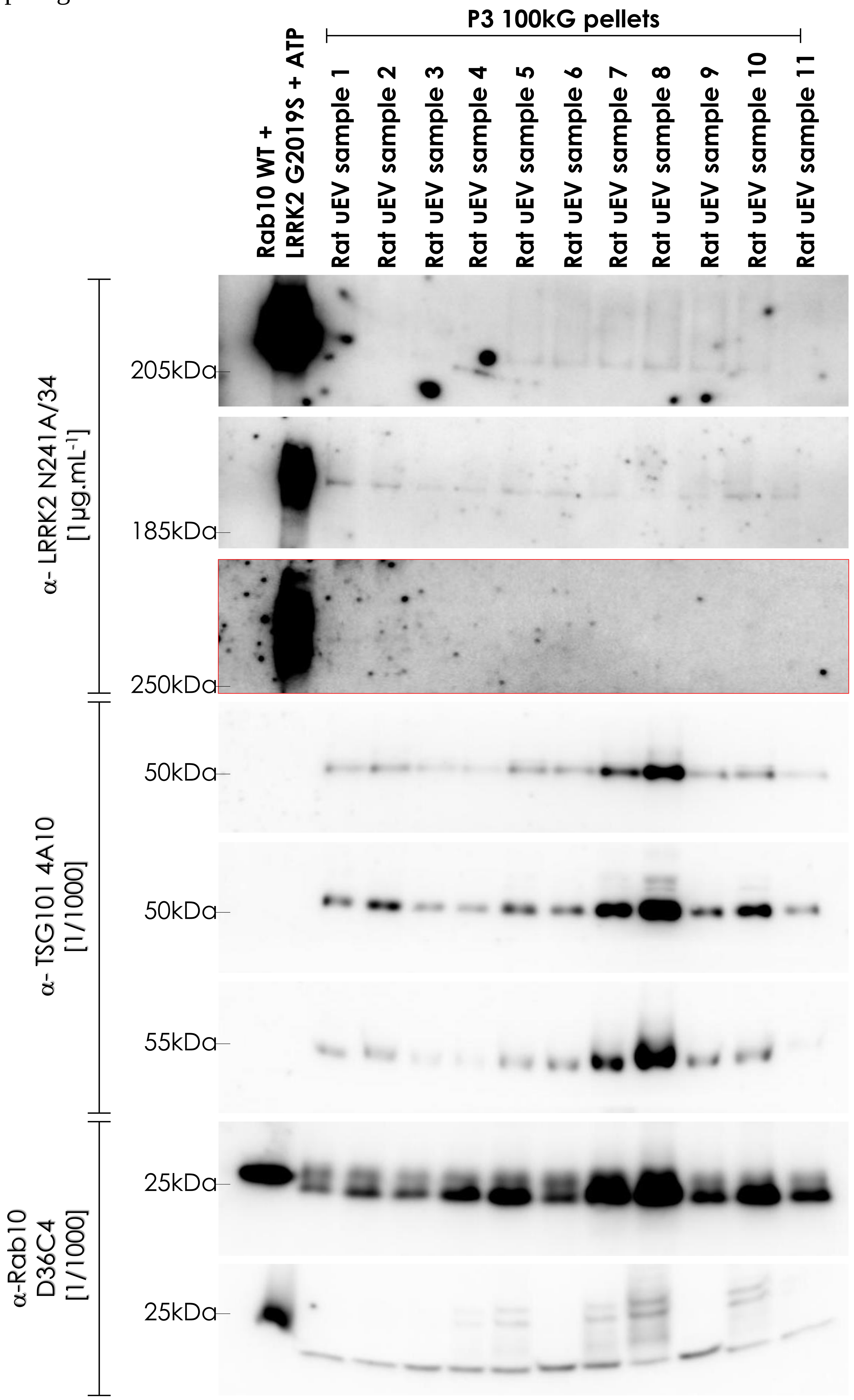

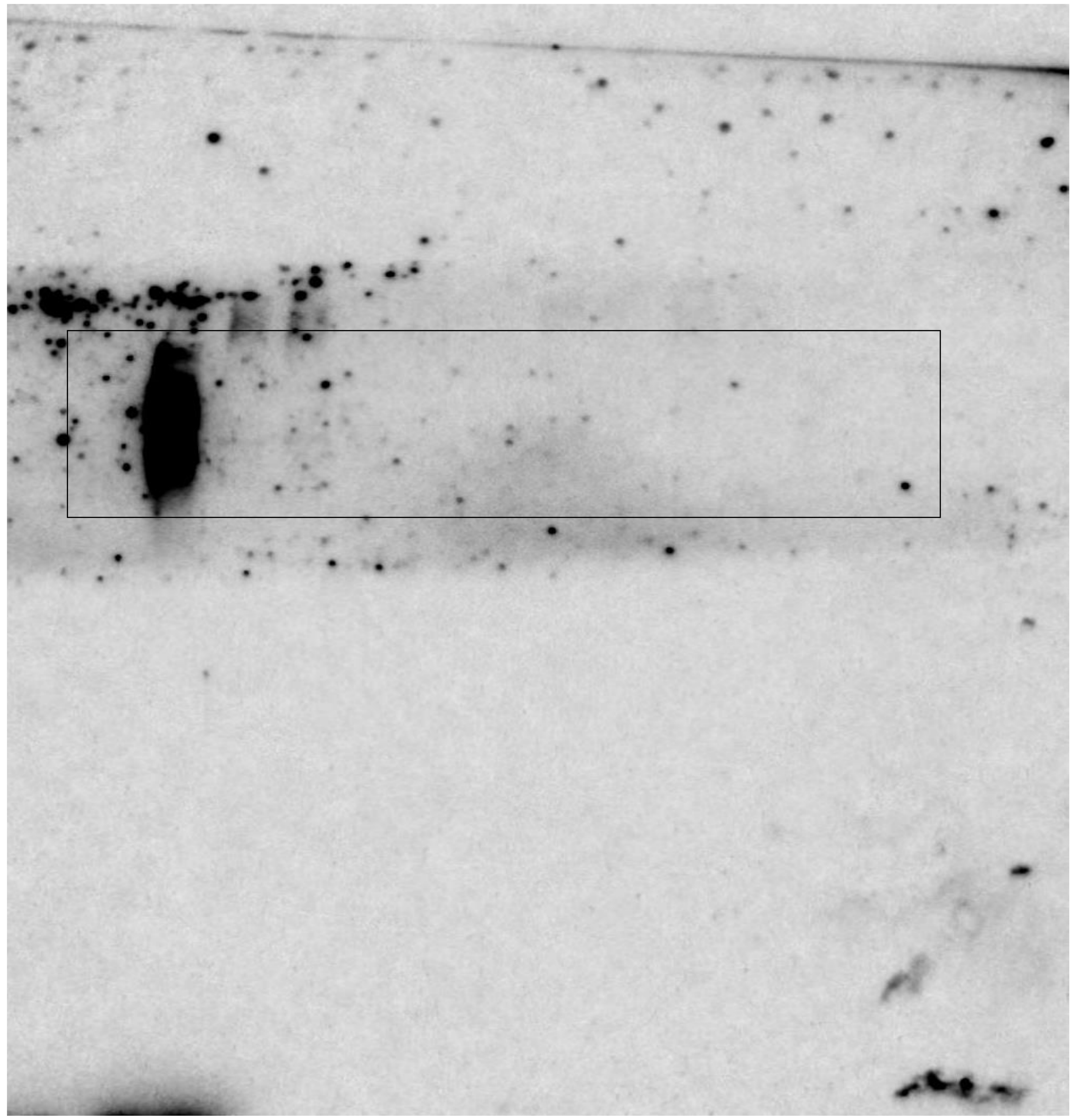

Suppl Figure 13a

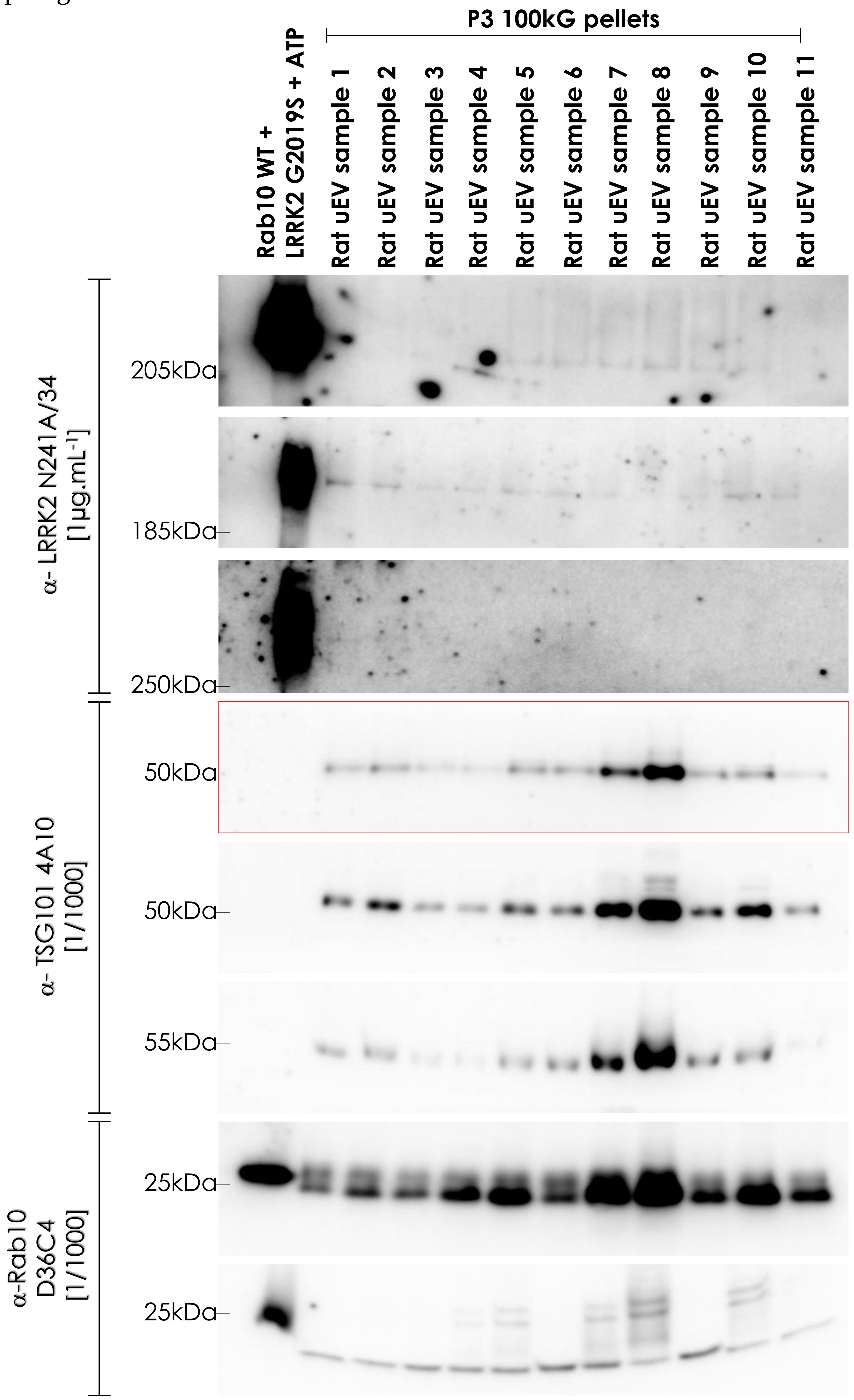

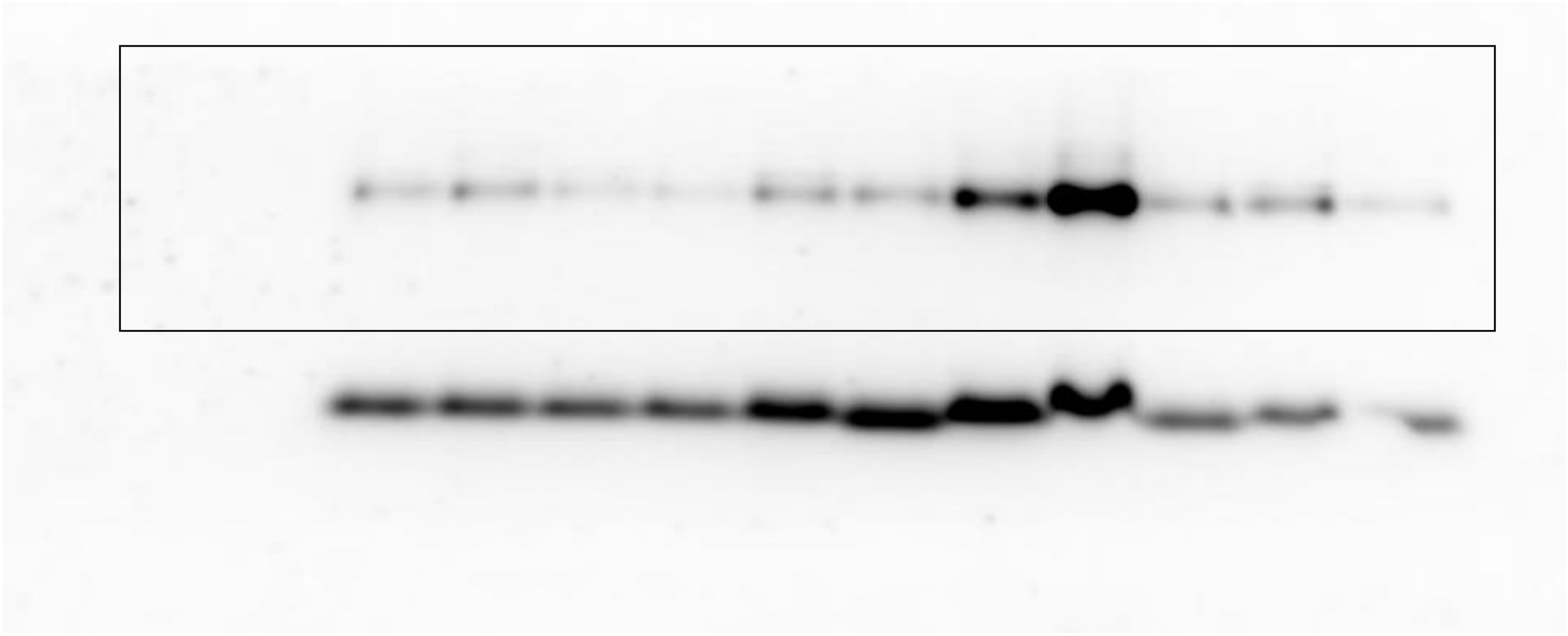

Suppl Figure 13a

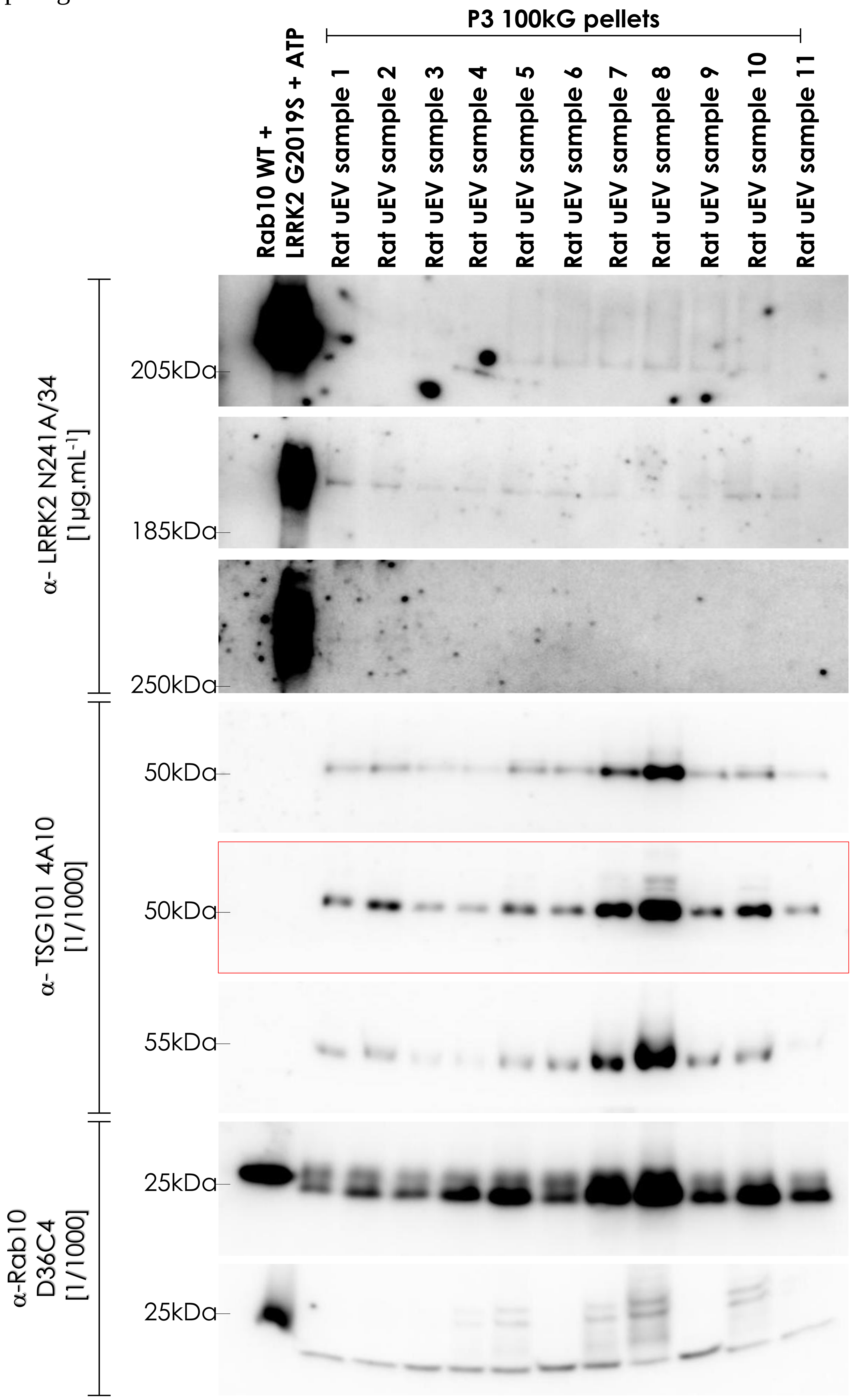

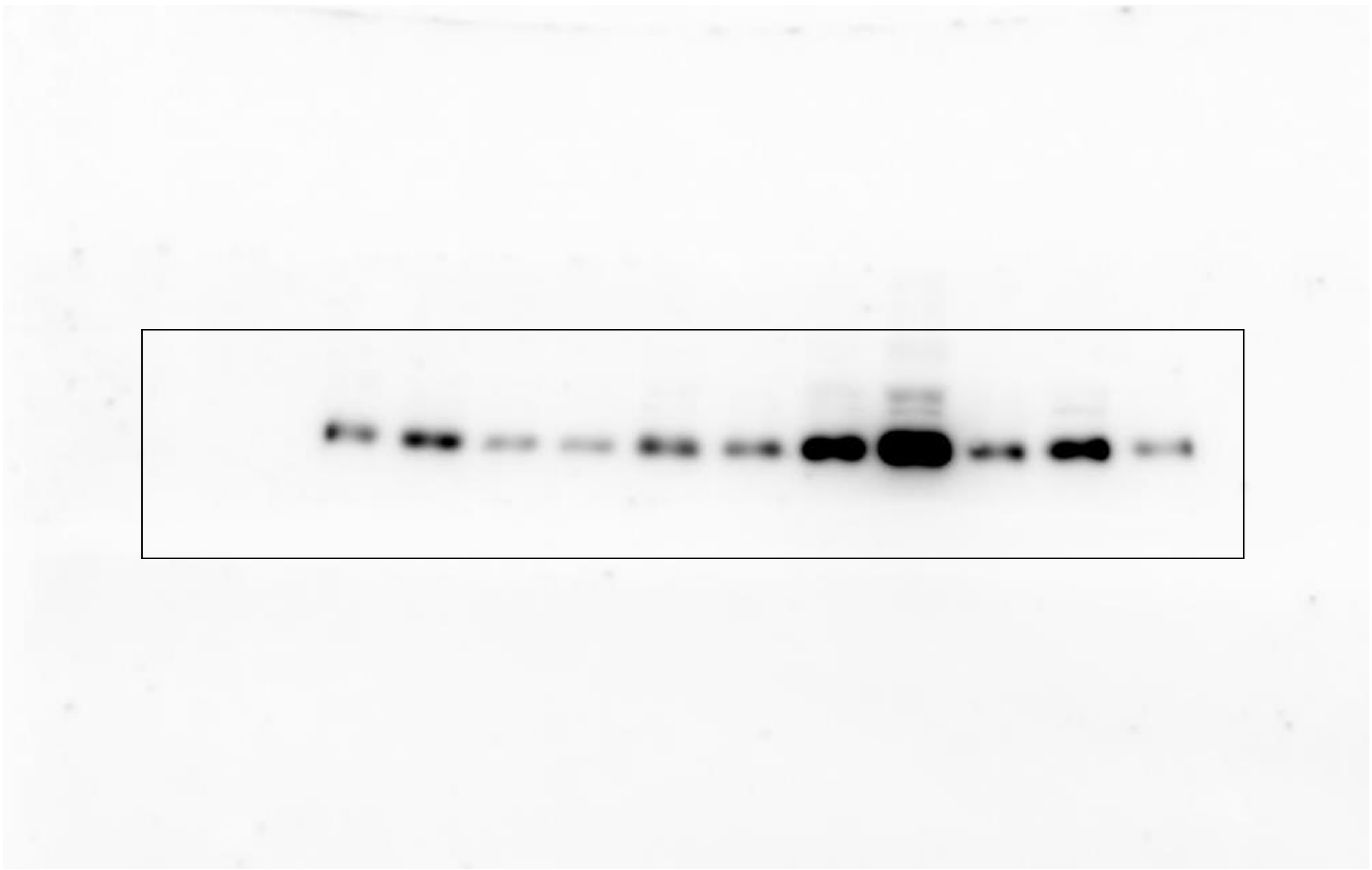

Suppl Figure 13a

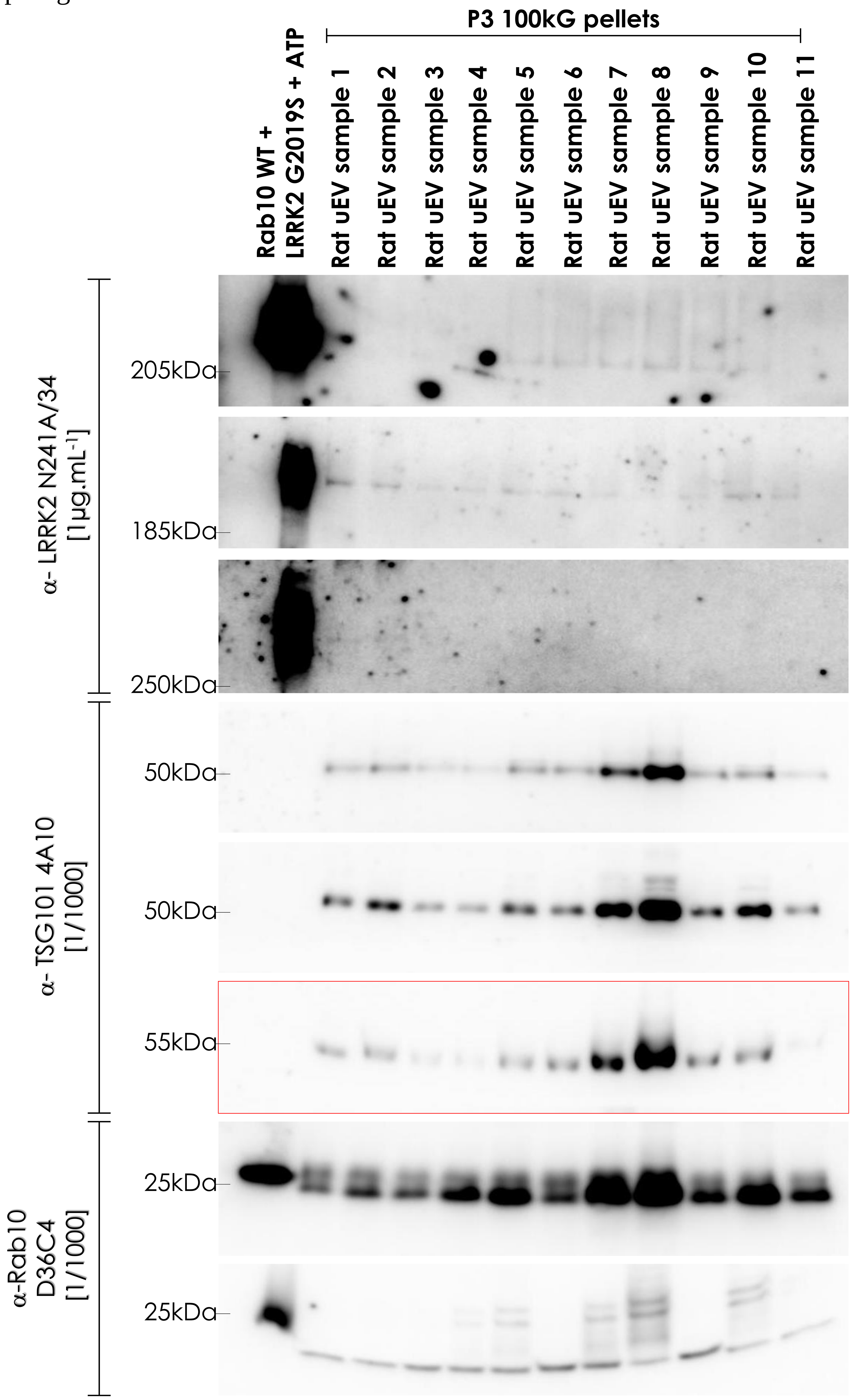

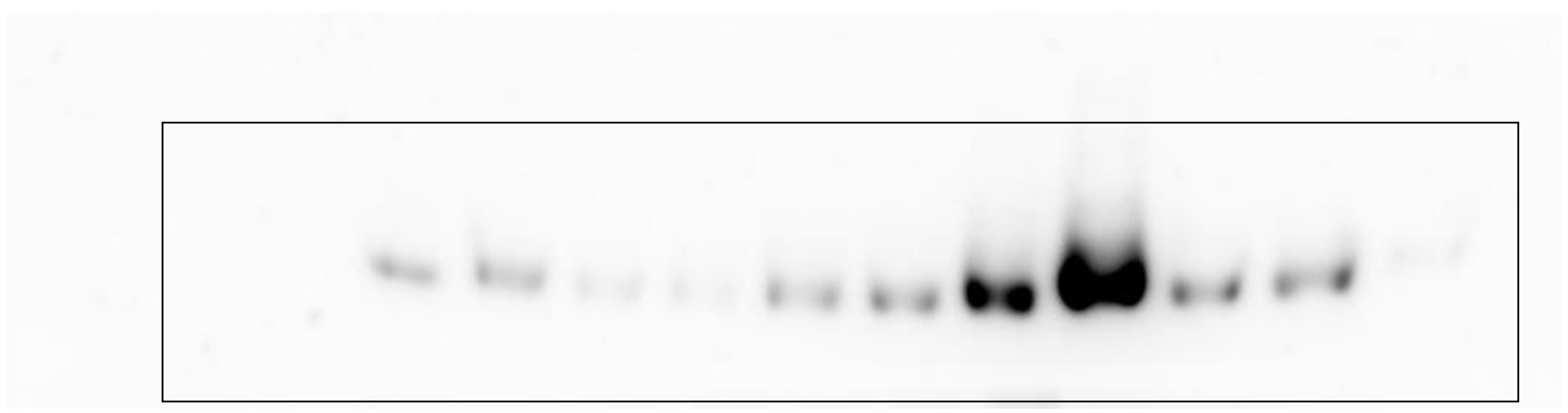

Suppl Figure 13a

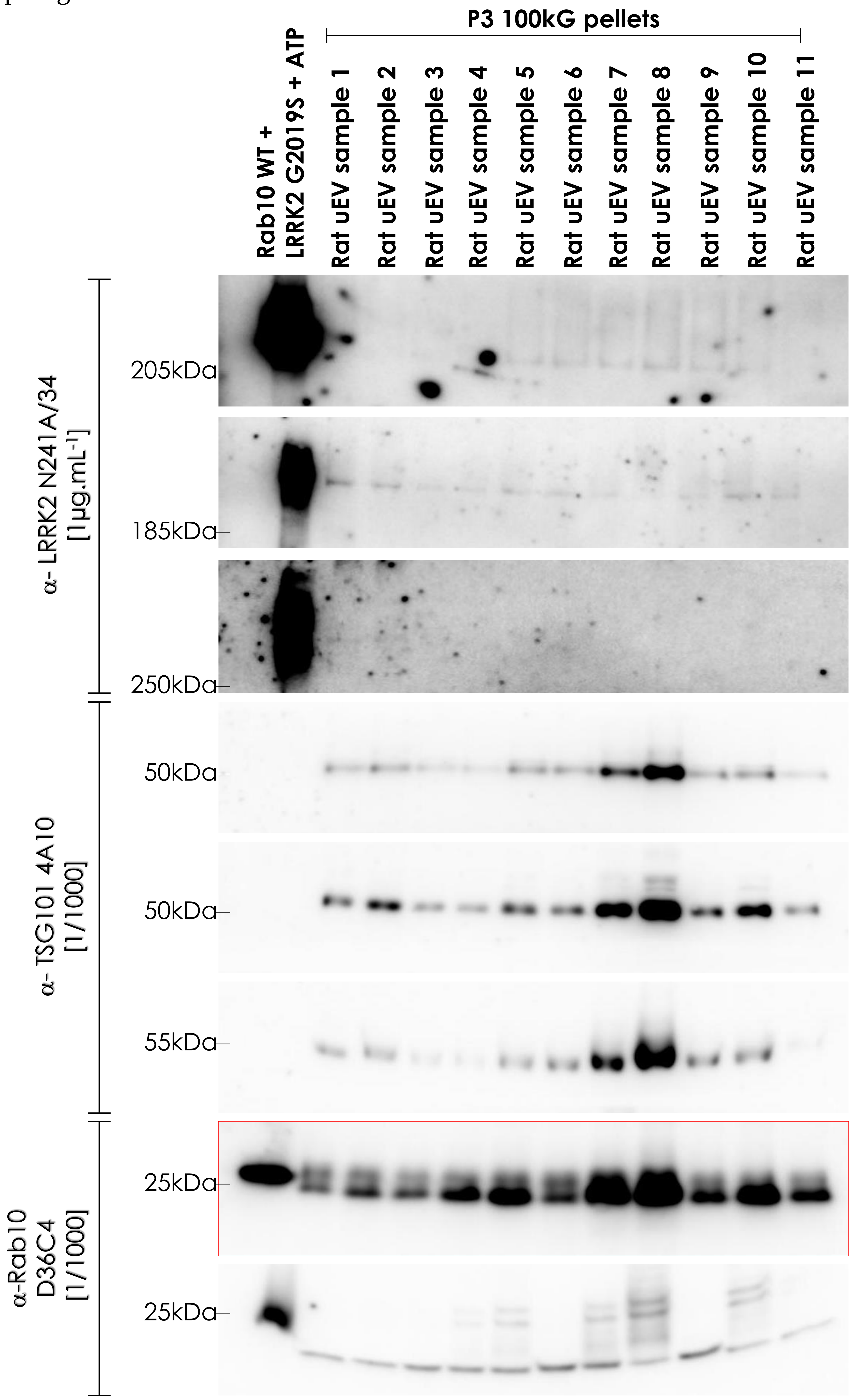

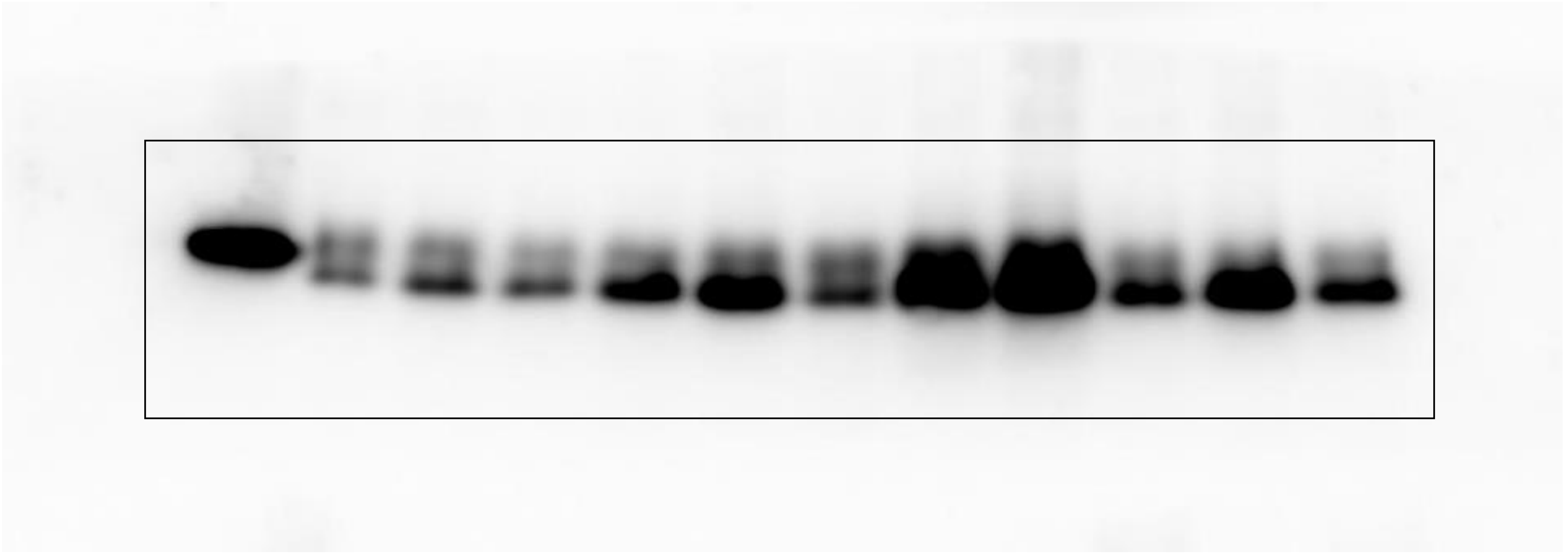

Suppl Figure 13a

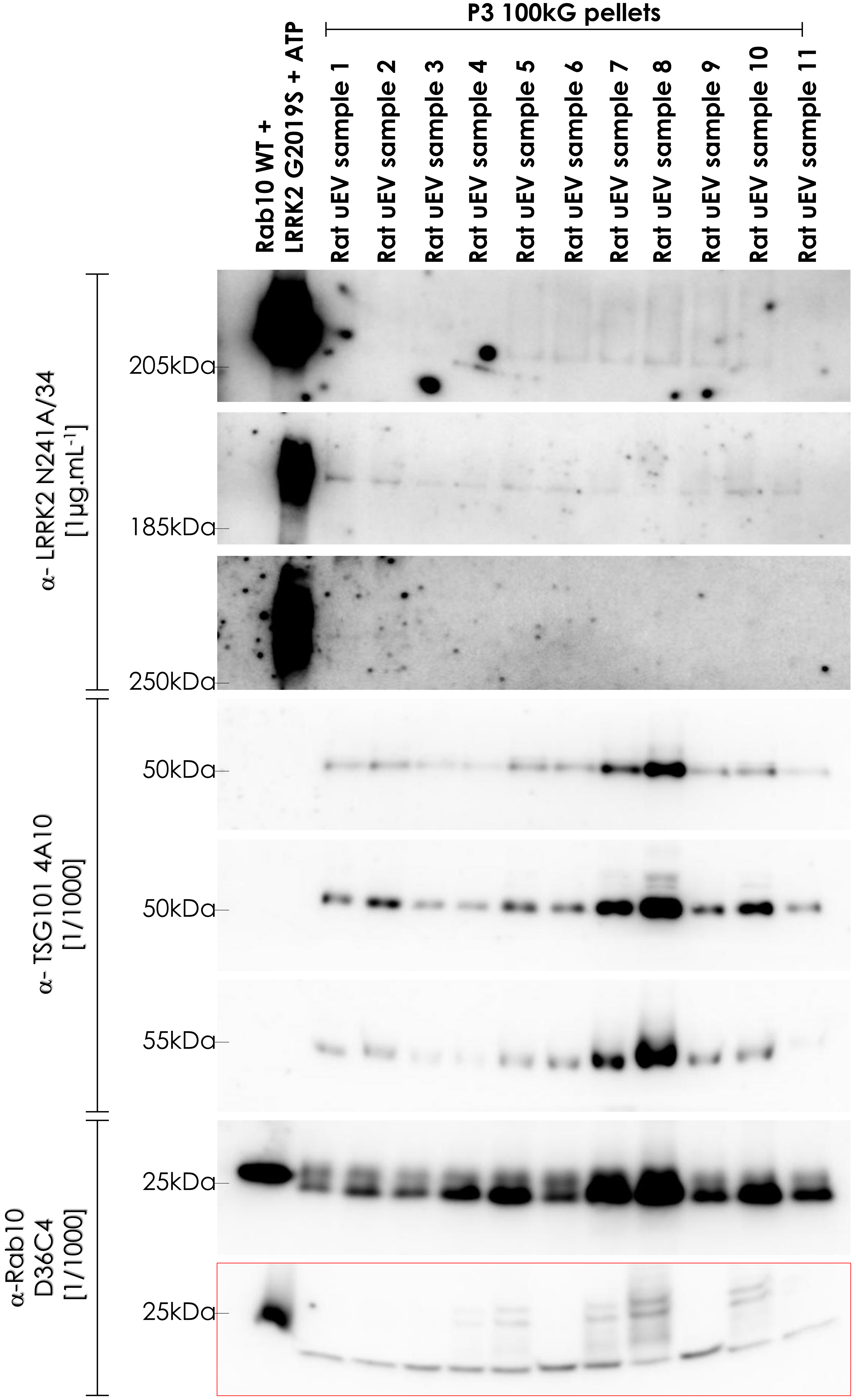

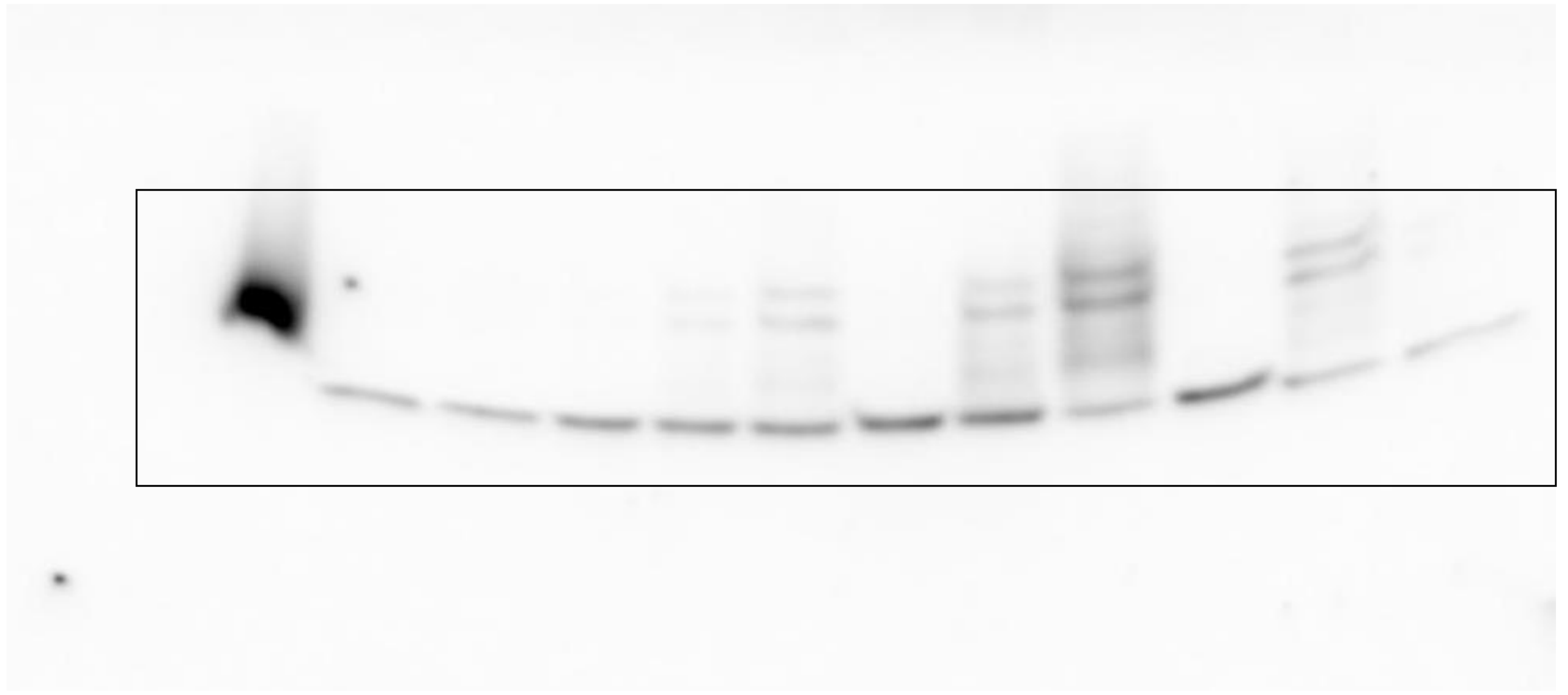

Suppl Figure 13b

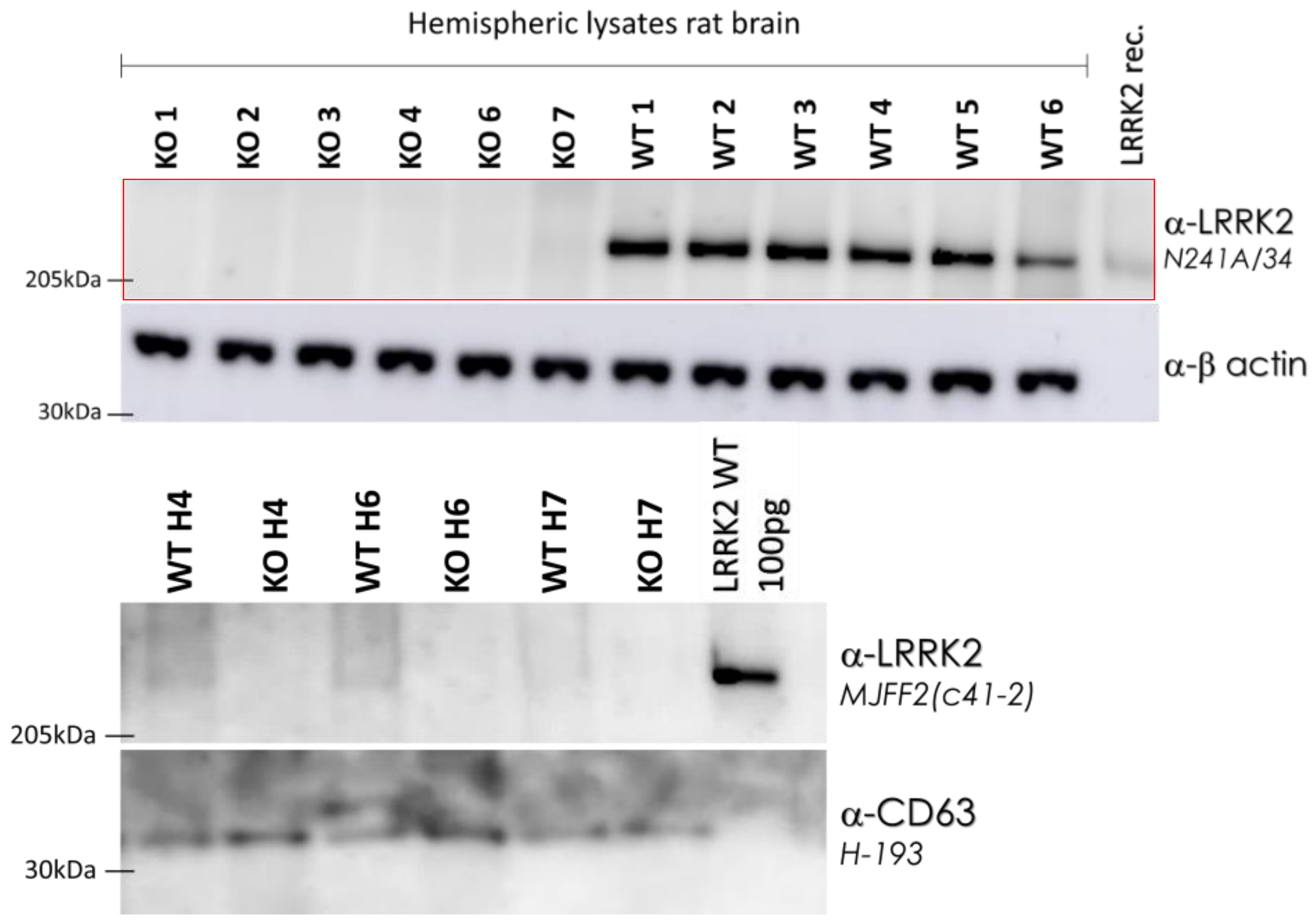

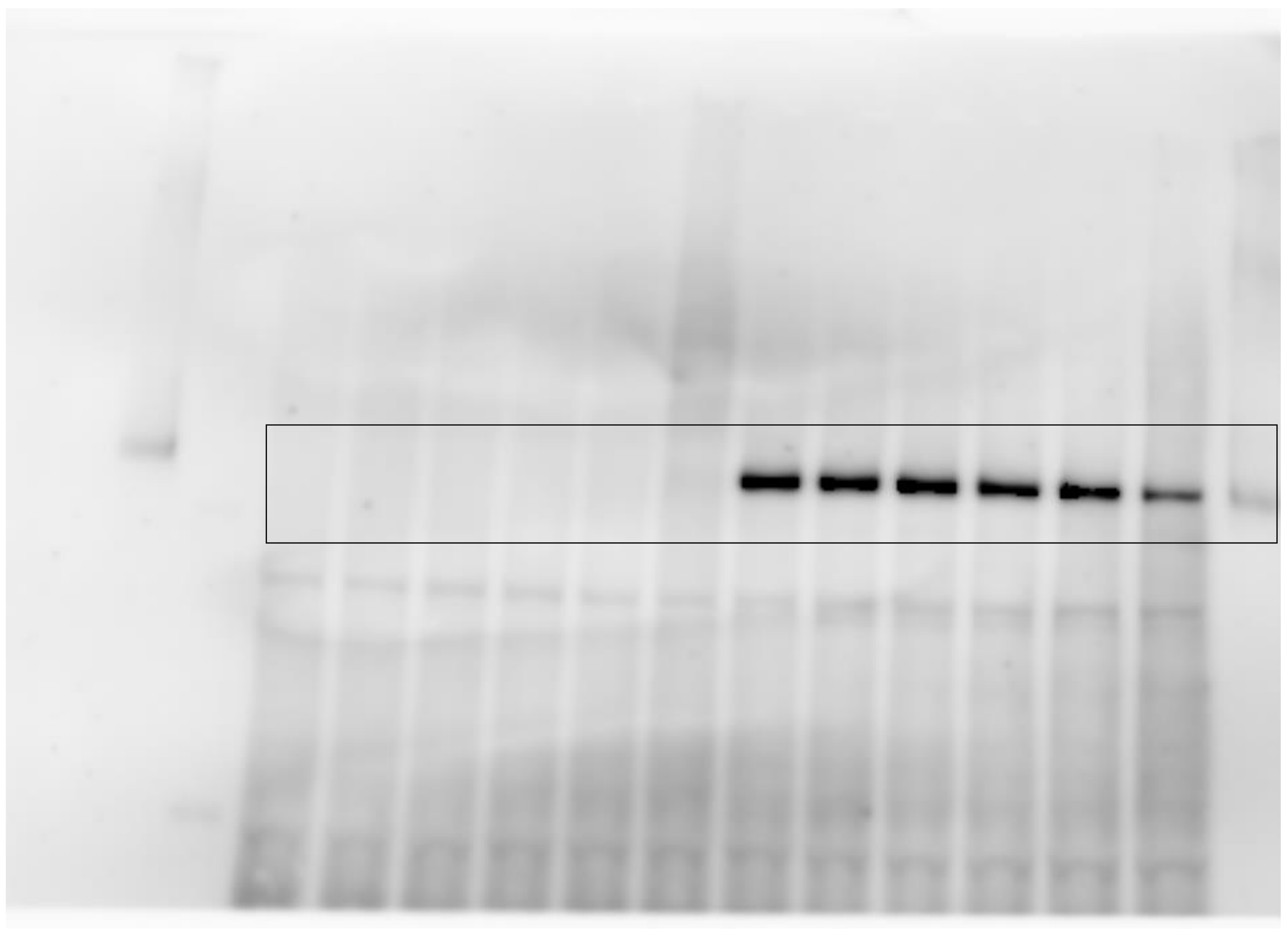

Suppl Figure 13b

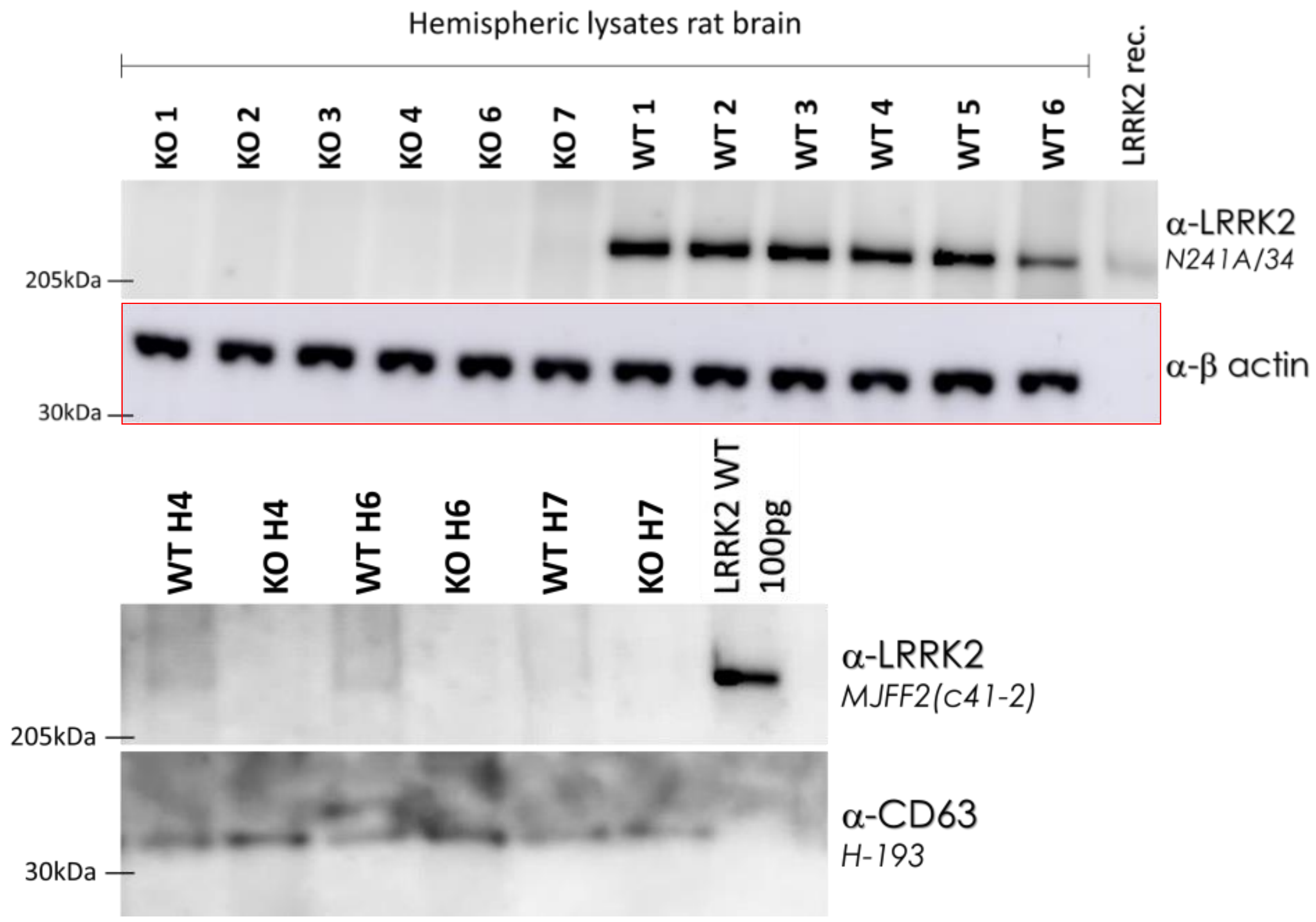

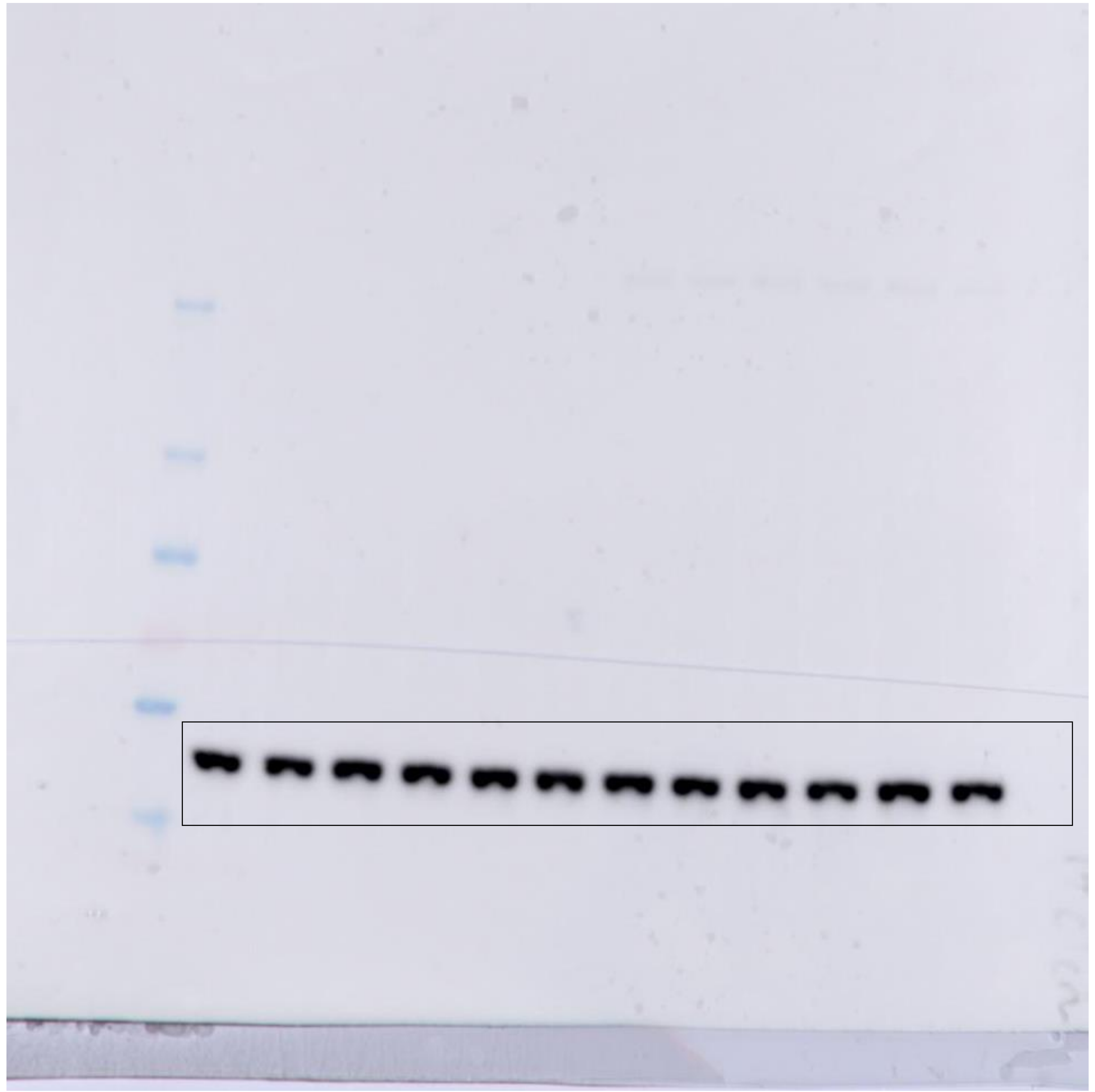

Suppl Figure 13b

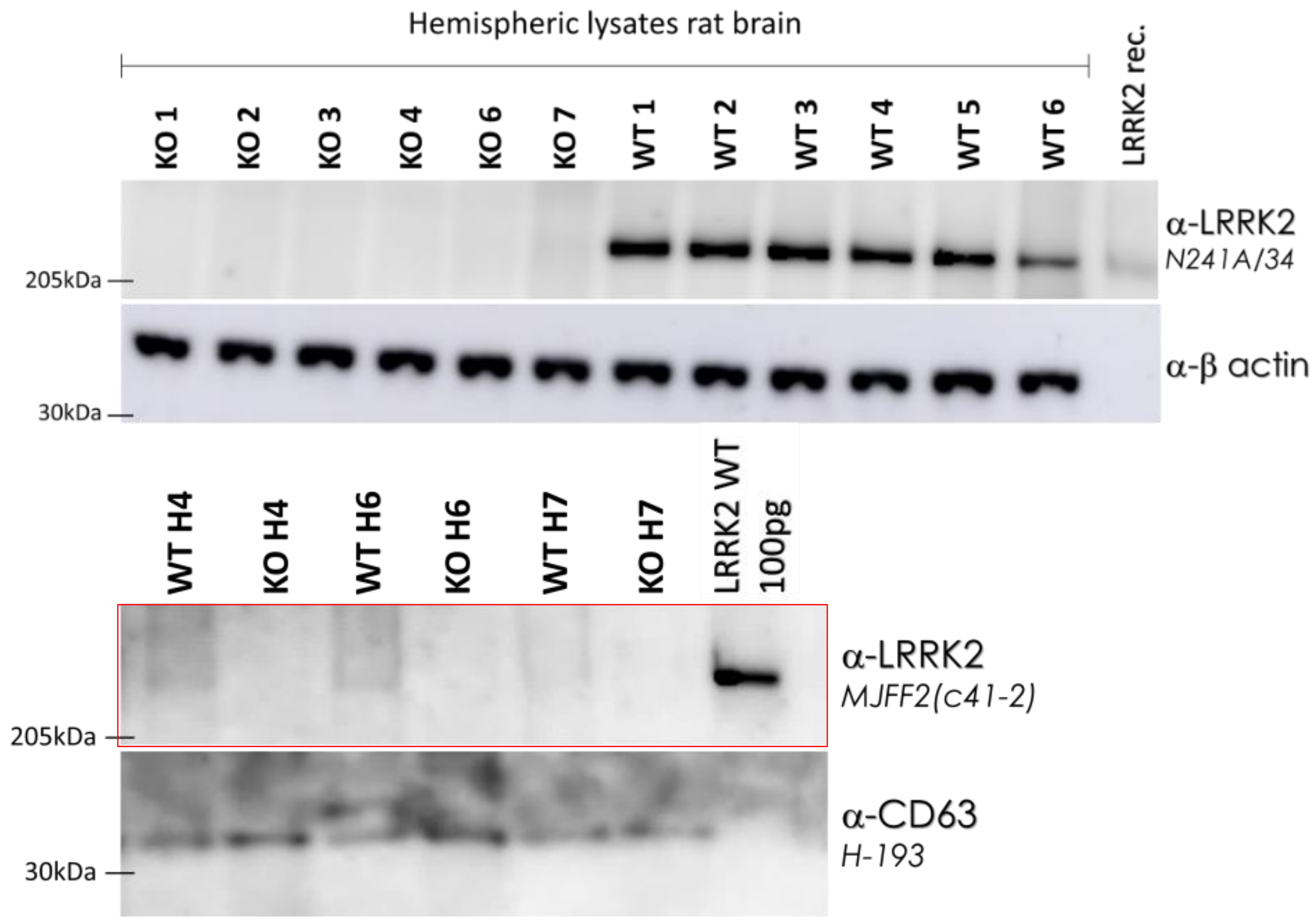

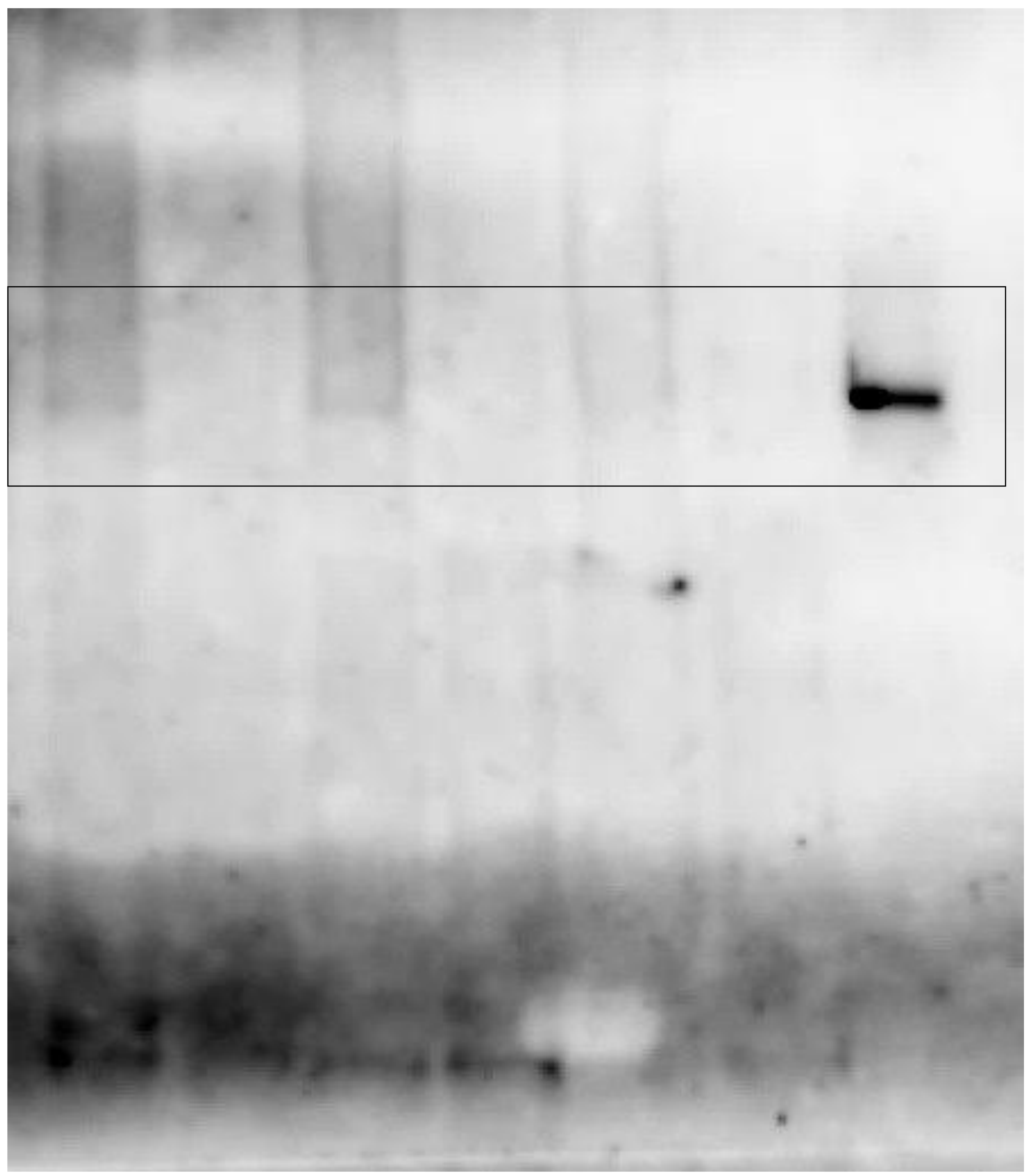

Suppl Figure 13b

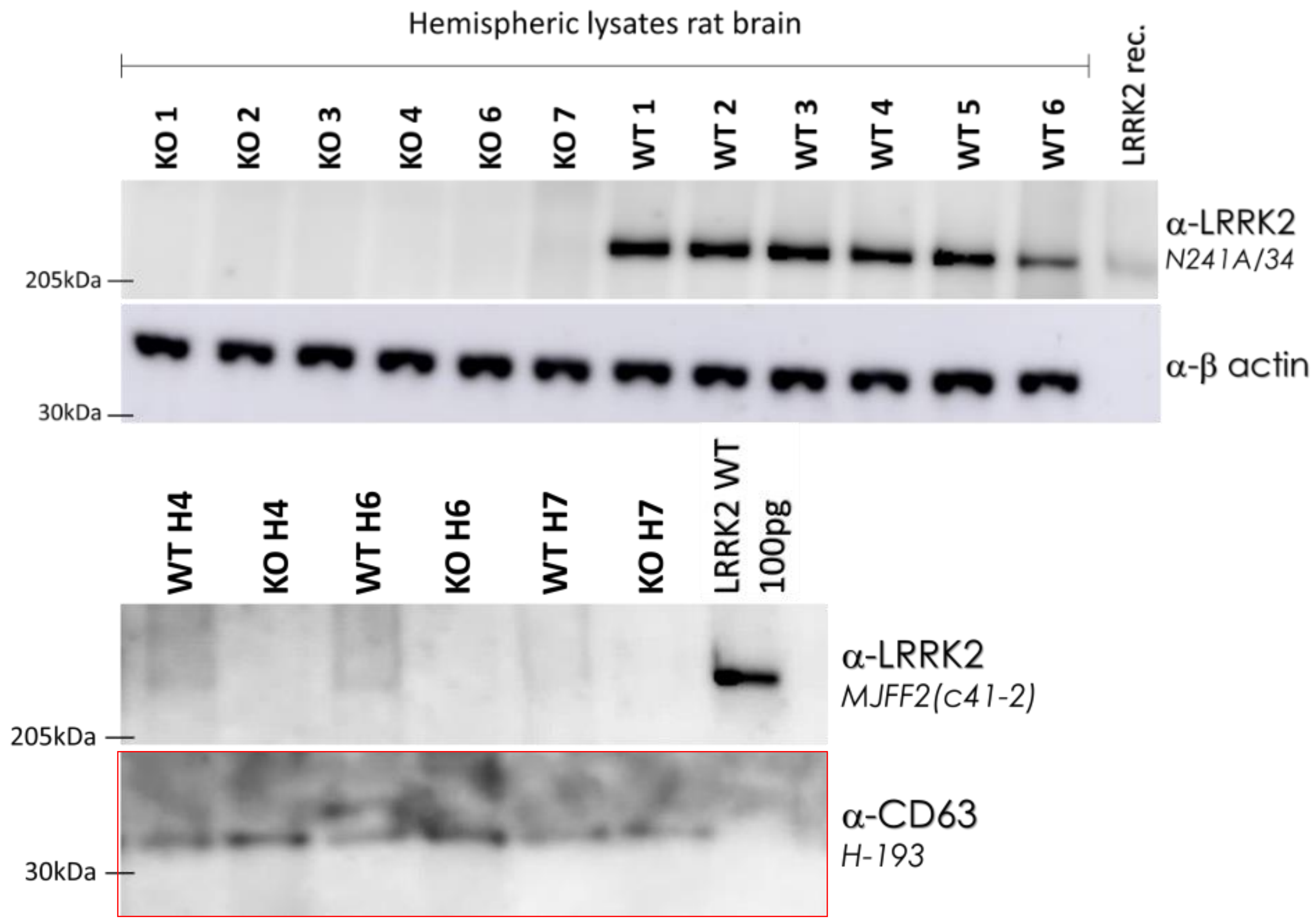

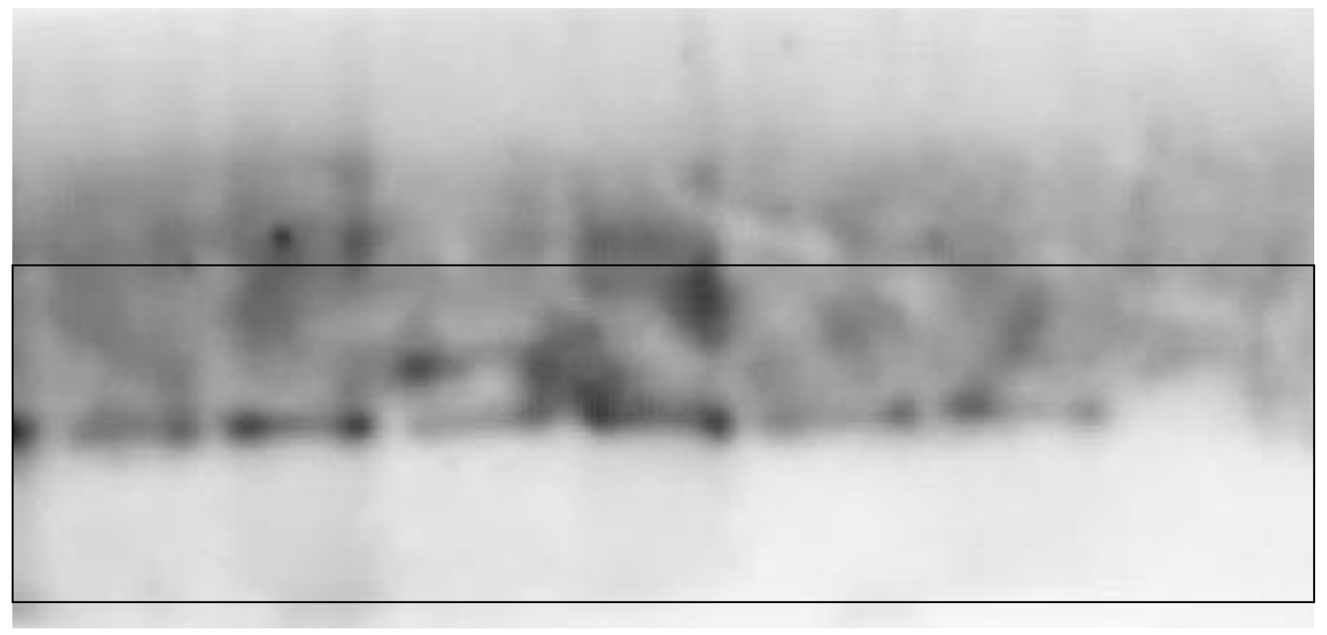

Suppl Figure S14a

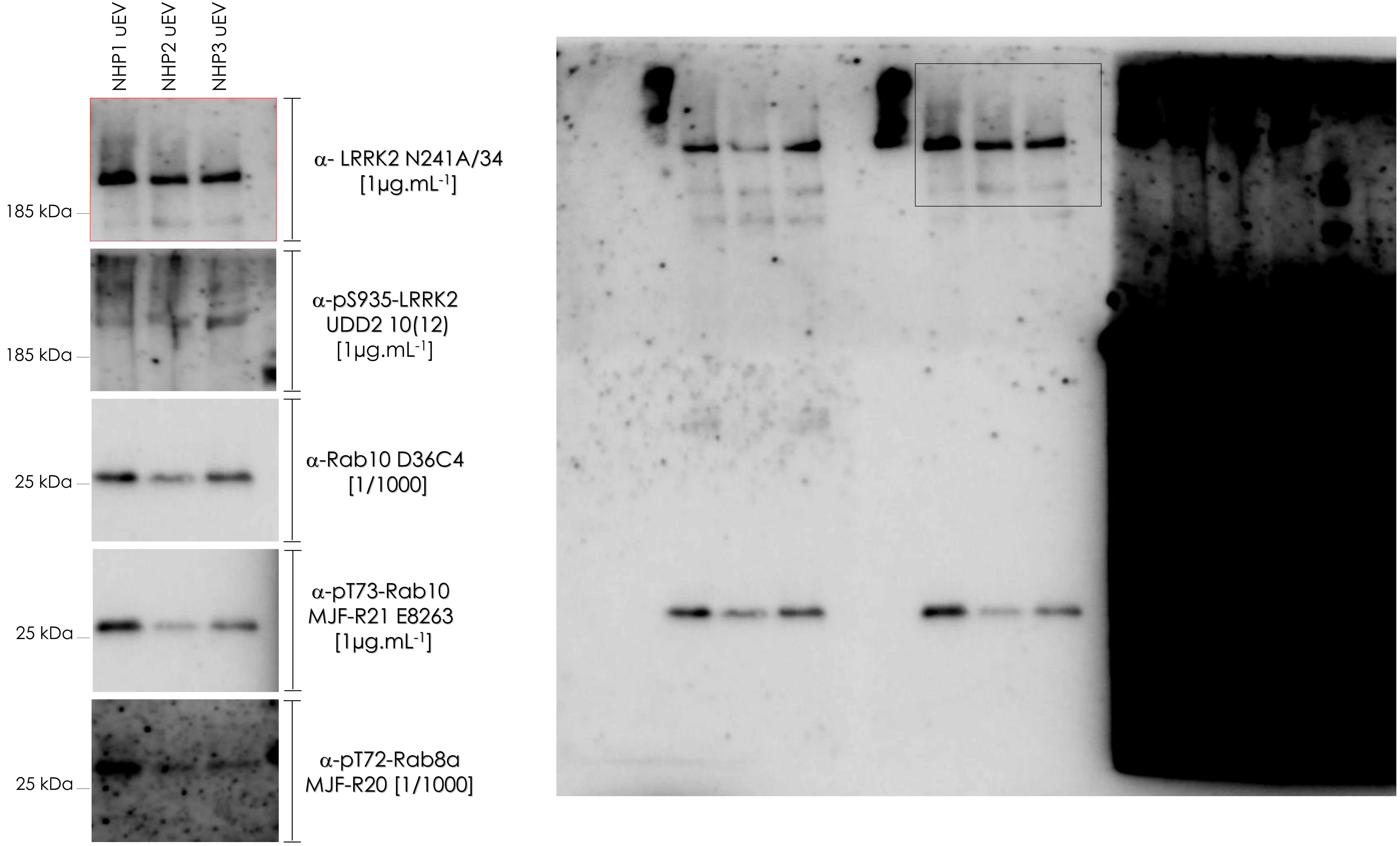

Suppl Figure S14a

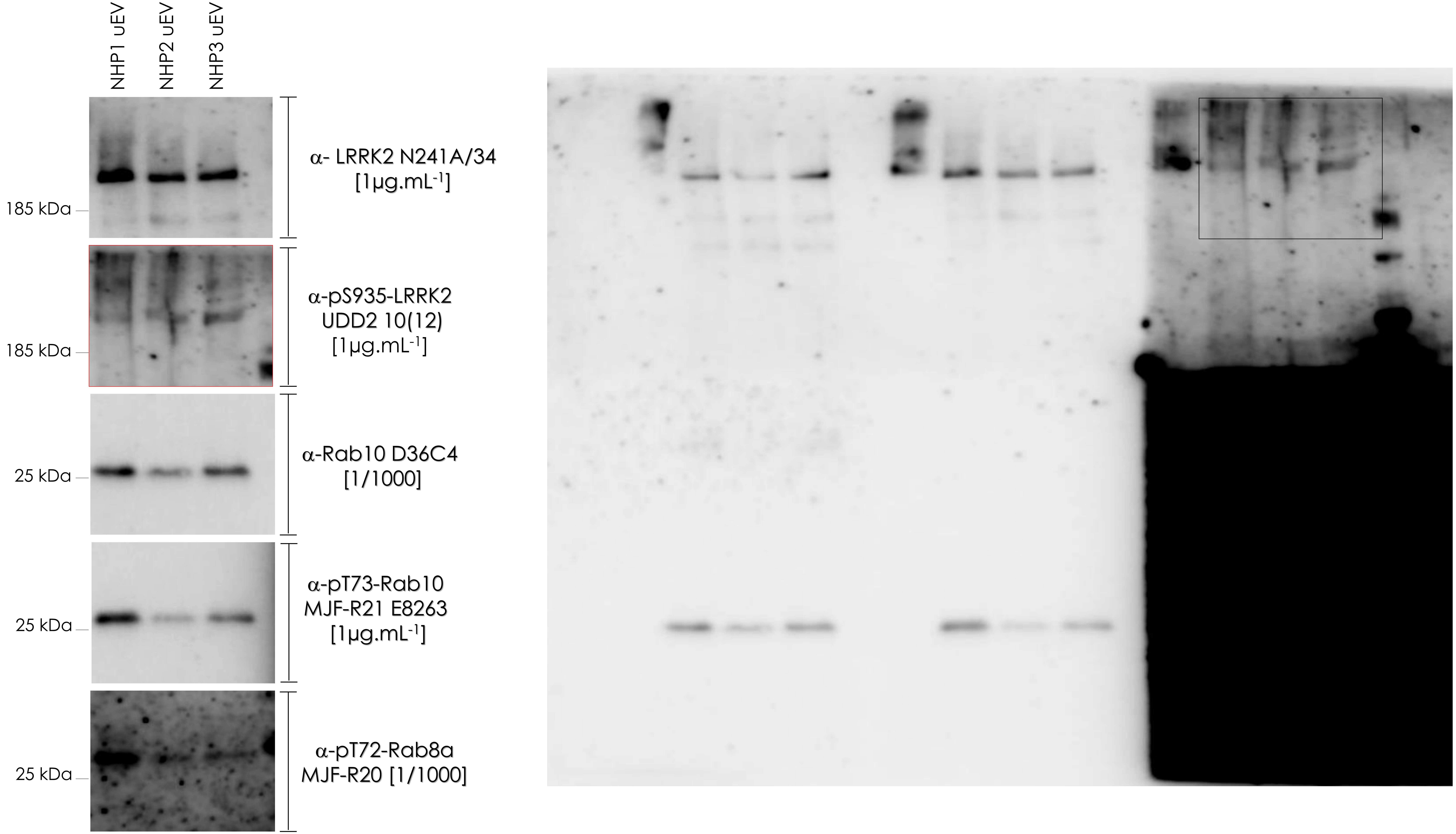

Suppl Figure S14a

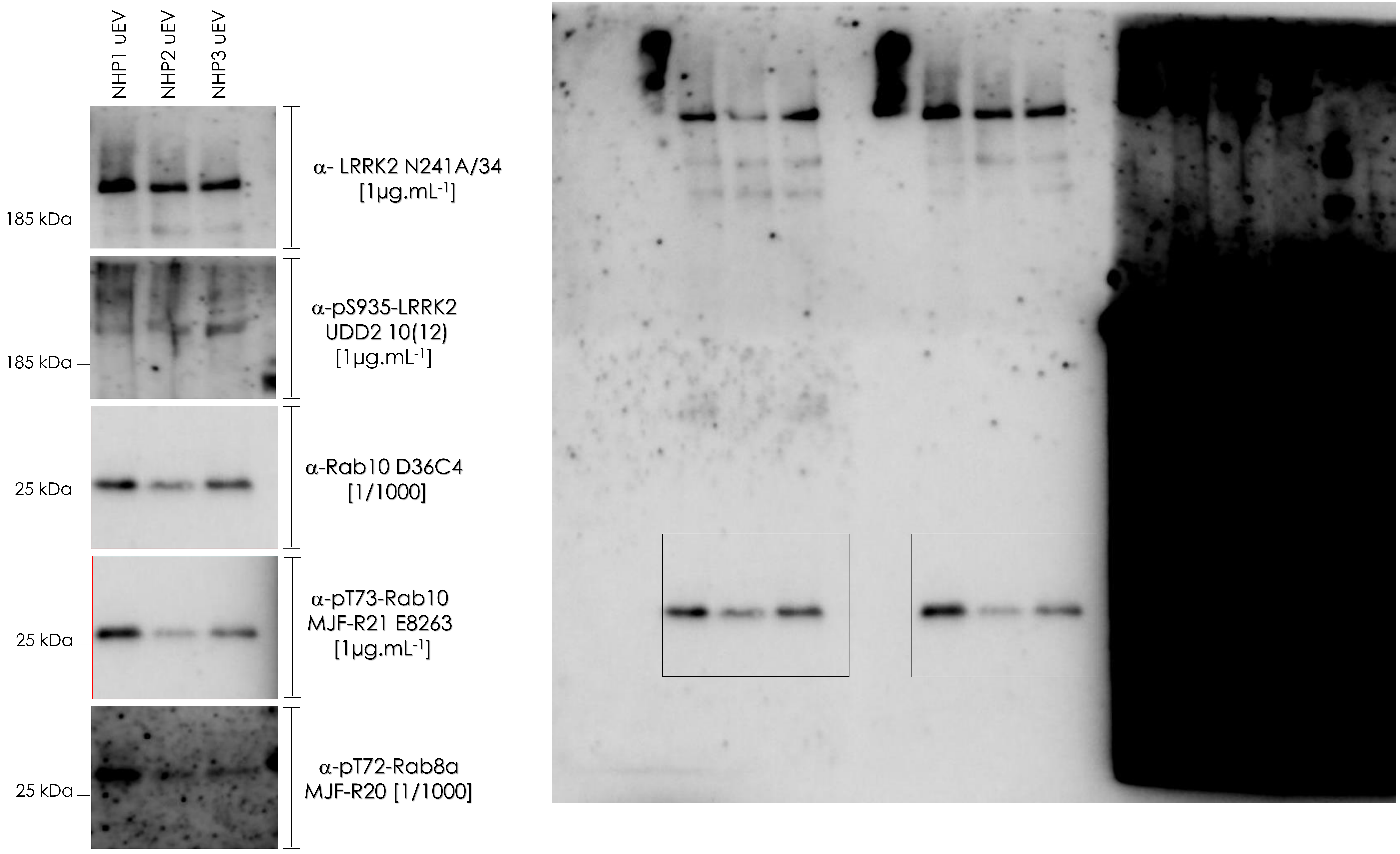

Suppl Figure S14a

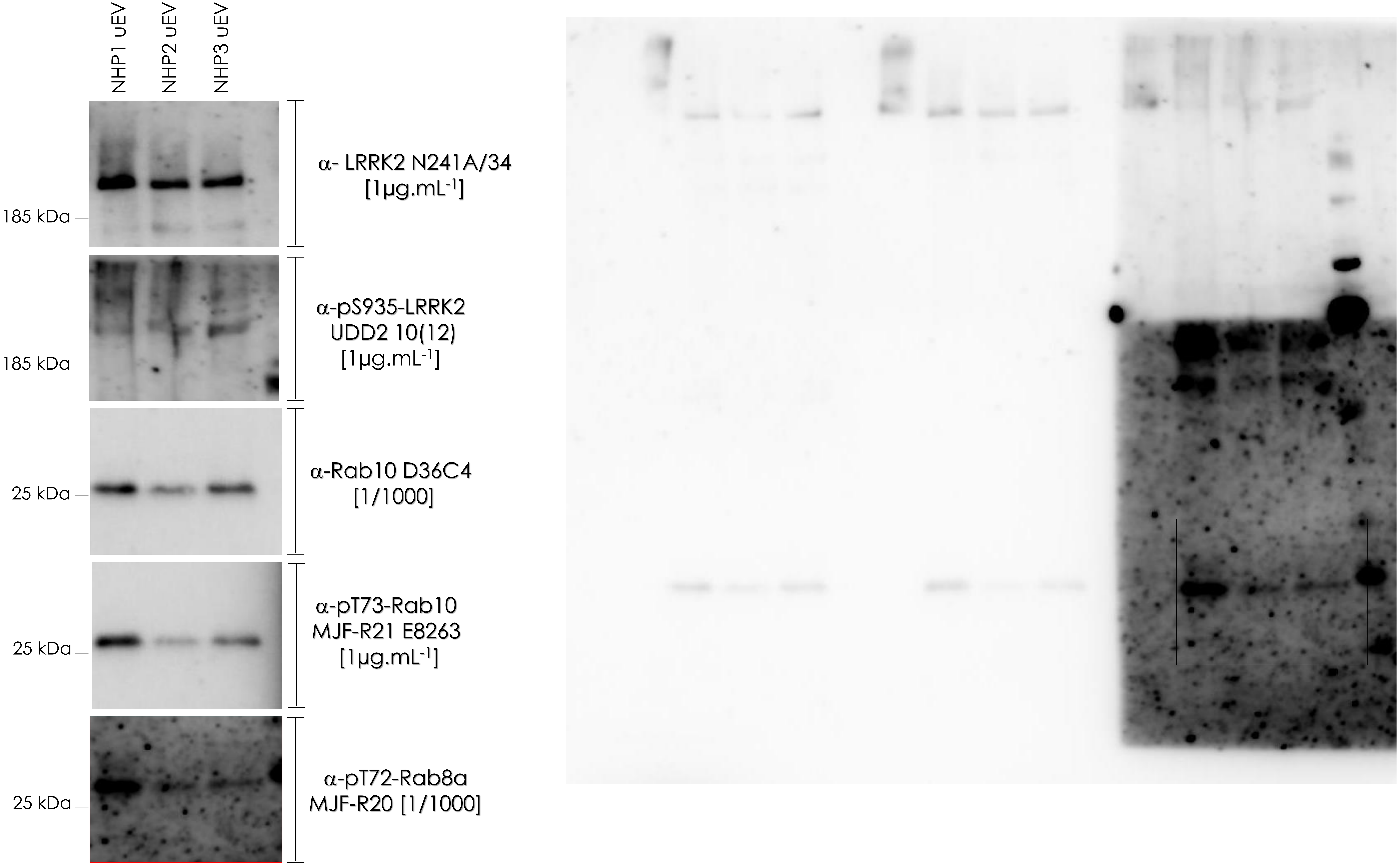

Suppl Figure 14b

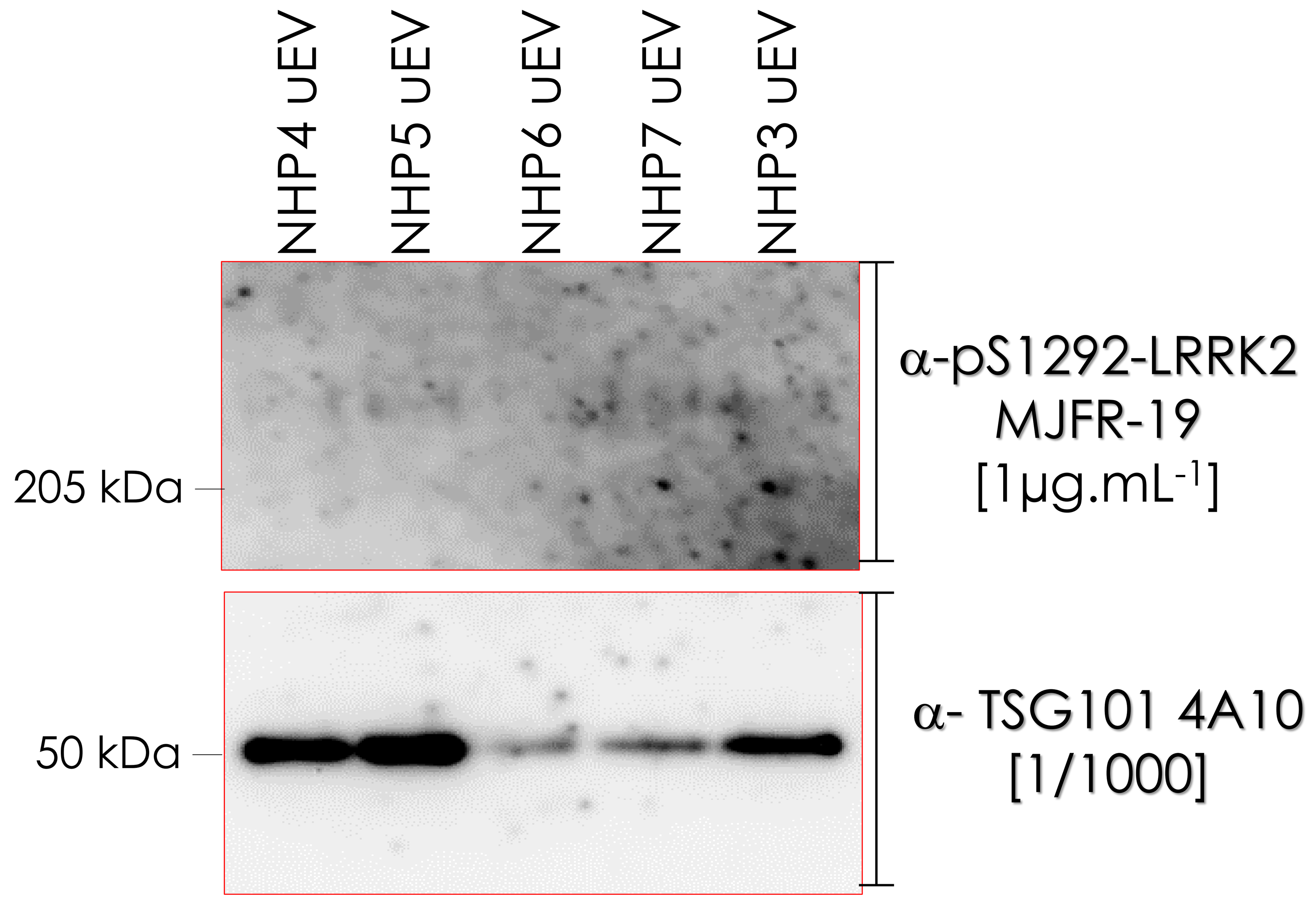

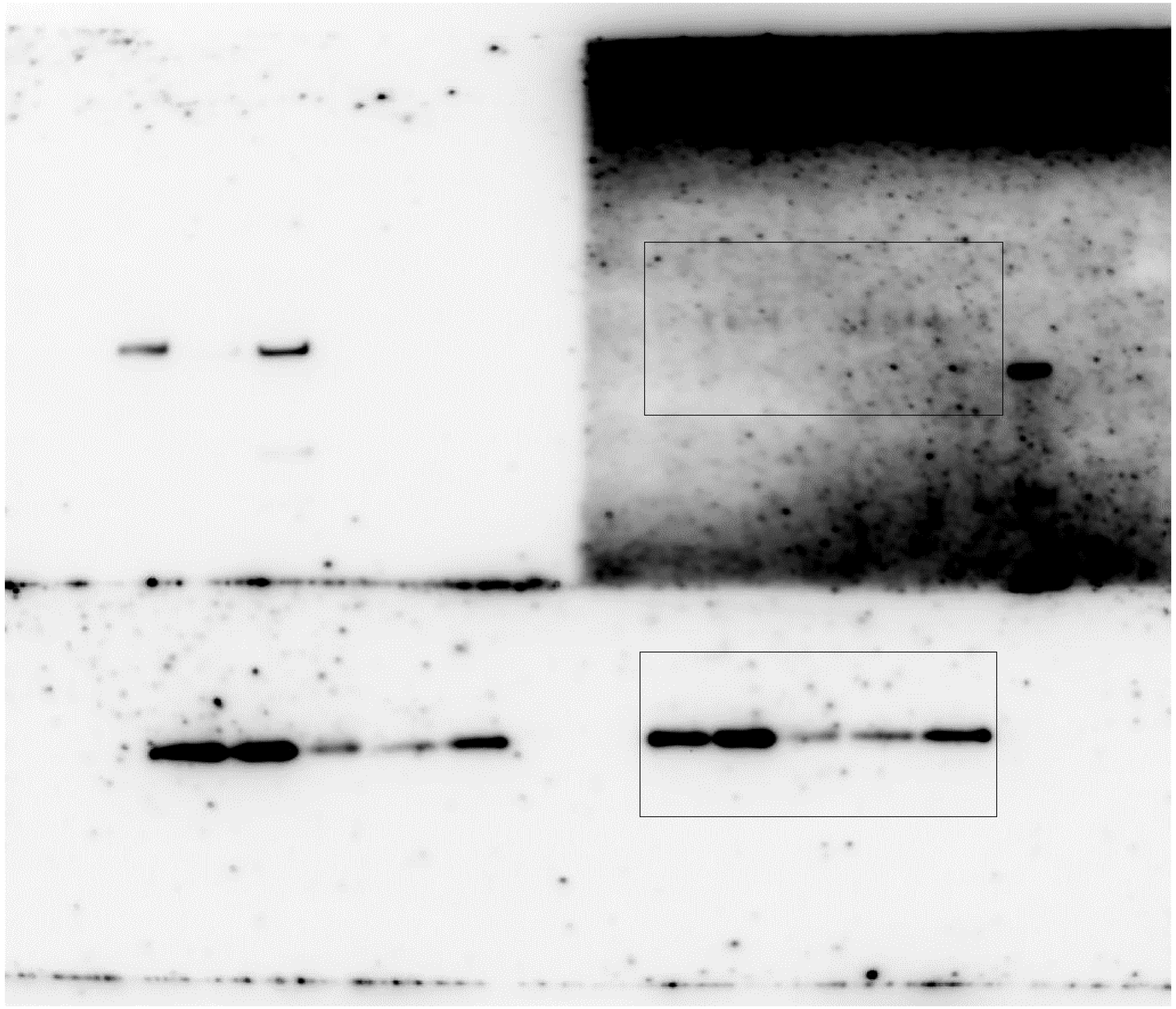

Supplement: Supplementary file 2 — Uncropped western blot images [file 41531_2023_445_MOESM2_ESM.pdf]
