# Supplementary material for: Alterations in the LRRK2-Rab pathway in urinary extracellular vesicles as Parkinson’s disease and pharmacodynamic biomarkers
Source: NPJ Parkinsons Dis. 2023 Feb 7;9:21. doi: 10.1038/s41531-023-00445-9 (PMC9905493; doi:10.1038/s41531-023-00445-9)
Supplement: Supplementary file 3 — Reporting Summary [file 41531_2023_445_MOESM3_ESM.pdf]

nature portfolio

Corresponding author(s): Jean-Marc Taymans & Marie-Christine Chartier-Harlin

Last updated by author(s): Dec 31, 2022

Reporting Summary

Nature Portfolio wishes to improve the reproducibility of the work that we publish. This form provides structure for consistency and transparency in reporting. For further information on Nature Portfolio policies, see our Editorial Policies and the Editorial Policy Checklist.

Statistics

For all statistical analyses, confirm that the following items are present in the figure legend, table legend, main text, or Methods section.

n/a	Confirmed	
☐	☒	The exact sample size (*n*) for each experimental group/condition, given as a discrete number and unit of measurement
☐	☒	A statement on whether measurements were taken from distinct samples or whether the same sample was measured repeatedly
☐	☒	The statistical test(s) used AND whether they are one- or two-sided<br>*Only common tests should be described solely by name; describe more complex techniques in the Methods section.*
☐	☒	A description of all covariates tested
☐	☒	A description of any assumptions or corrections, such as tests of normality and adjustment for multiple comparisons
☐	☒	A full description of the statistical parameters including central tendency (e.g. means) or other basic estimates (e.g. regression coefficient) AND variation (e.g. standard deviation) or associated estimates of uncertainty (e.g. confidence intervals)
☐	☒	For null hypothesis testing, the test statistic (e.g. *F*, *t*, *r*) with confidence intervals, effect sizes, degrees of freedom and *P* value noted<br>*Give P values as exact values whenever suitable.*
☒	☐	For Bayesian analysis, information on the choice of priors and Markov chain Monte Carlo settings
☒	☐	For hierarchical and complex designs, identification of the appropriate level for tests and full reporting of outcomes
☒	☐	Estimates of effect sizes (e.g. Cohen's *d*, Pearson's *r*), indicating how they were calculated

Our web collection on statistics for biologists contains articles on many of the points above.

Software and code

Policy information about availability of computer code

Data collection	ImageJ (open source), ImageQuant, Microsoft Excel
Data analysis	Microsoft Excel, GraphPad Prism

For manuscripts utilizing custom algorithms or software that are central to the research but not yet described in published literature, software must be made available to editors and reviewers. We strongly encourage code deposition in a community repository (e.g. GitHub). See the Nature Portfolio guidelines for submitting code & software for further information.

Data

Policy information about availability of data

All manuscripts must include a data availability statement. This statement should provide the following information, where applicable:

- Accession codes, unique identifiers, or web links for publicly available datasets
- A description of any restrictions on data availability
- For clinical datasets or third party data, please ensure that the statement adheres to our policy

Data used in preparation of this article were obtained from the MJFF-sponsored LRRK2 Cohort Consortium (LCC). For up-to-date information on the study, visit www.michaeljfox.org./lcc). For the data presented in graphs of this paper, excel files with individual values of each tested sample can be provided upon request.

Human research participants

Policy information about studies involving human research participants and Sex and Gender in Research.

Reporting on sex and gender	Gender characteristics have been collected in the human cohorts studied
Population characteristics	Cohorts of patients with PD and matched controls were included in the study, see recruitment. Population characteristics are presented in the manuscript, Tables 1 and 2.
Recruitment	The study of urinary EVs in PD was conducted in using samples from two different cohorts.<br>The first first cohort of samples was obtained from the Michael J. Fox Foundation (MJFF) LRRK2 Cohort Consortium (LCC) biobank. The LCC study was established in 2009, when the MJFF LCC brought together investigators from North America, Europe, North Africa, and Asia to study individuals with mutations in the LRRK2 gene. Ethical review and approval of the LCC biobank was not required for the de-identified sample analysis in accordance with the local legislation and institutional requirements. The patients/participants provided their written informed consent to participate in this study. All participants included are male: 36 G2019S LRRK2 mutation carriers (20 with PD and 16 without PD) and 39 non carriers (20 with PD and 19 without PD). These participants were selected to match with our second cohort (cfr. infra) as closely as possible<br>The second cohort followed at the Lille University Hospital Movement Disorders Department included 120 participants (67 diagnosed with PD and 53 healthy controls) followed at the Lille University Movement Disorders Department, and enrolled between June 2015 and October 2017. Each subject provided written consent to participate prior to inclusion. The protocol (reference CONVERGENCE cohort CPP 2008-A00219-42) was approved by the independent ethics committee for northwestern France (Comité de Protection des Personnes Nord-Ouest IV).
Ethics oversight	Comité de Protection des Personnes Nord-Ouest IV

Note that full information on the approval of the study protocol must also be provided in the manuscript.

Field-specific reporting

Please select the one below that is the best fit for your research. If you are not sure, read the appropriate sections before making your selection.

☒ Life sciences ☐ Behavioural & social sciences ☐ Ecological, evolutionary & environmental sciences

For a reference copy of the document with all sections, see nature.com/documents/nr-reporting-summary-flat.pdf

Life sciences study design

All studies must disclose on these points even when the disclosure is negative.

Sample size	Sample sizes were determined based on previous studies on similar measures (PMID 27297049 and 26865512), showing that N=14 is sufficient to identify differences in the epitopes studied.
Data exclusions	No data was excluded
Replication	2 different patient cohorts were tested. All samples were tested in triplicate or quadruplicate
Randomization	Samples were analyzed in a random manner and sample organization on western blots was done in a random manner
Blinding	Quantification of the data was done with staff blinded to the identity of the samples

Reporting for specific materials, systems and methods

We require information from authors about some types of materials, experimental systems and methods used in many studies. Here, indicate whether each material, system or method listed is relevant to your study. If you are not sure if a list item applies to your research, read the appropriate section before selecting a response.

Materials & experimental systems

n/a	Involved in the study
☐	☒ Antibodies
☒	☐ Eukaryotic cell lines
☒	☐ Palaeontology and archaeology
☐	☒ Animals and other organisms
☒	☐ Clinical data
☒	☐ Dual use research of concern

Methods

n/a	Involved in the study
☒	☐ ChIP-seq
☒	☐ Flow cytometry
☒	☐ MRI-based neuroimaging

Antibodies

Antibodies used

Antibodies are listed in a table in the materials and methods, also pasted below.
Detection epitope Clone Species Supplier
Total LRRK2 MJFF2 (C41-2) Rabbit Abcam (ab133474)
Total LRRK2 UDD3 30(12) Rabbit Abcam (ab133518)
Total LRRK2 N241A/34 Mouse UC Davis/NIH NeuroMab Facility (75-253)
Total LRRK2 D18E12 Rabbit Ozyme (#13046)
Total LRRK2 MC.028.83.76.242 Mouse BioLegend (SIG-39840-100)

pS910-LRRK2 UDD1 15(3) Rabbit Abcam (ab133449)
pS935-LRRK2 UDD2 10(12) Rabbit Abcam (ab133450)
pS955-LRRK2 MJF-R11 (75-1) Rabbit Abcam (ab169521)
pS973-LRRK2 MJF-R12(37-1) Rabbit Abcam (ab181364)
pS1292-LRRK2 MJF-R19-7-8 Rabbit Abcam (ab203181)
Total Rab8 D22D8 Rabbit Ozyme (#6975)

pT72-Rab8a MJF-R20 Rabbit Abcam (ab230260)
pT72-Rab8a Polyclonal Rabbit MRC PPU (E8263)
Total Rab10 D36C4 Rabbit Ozyme (#8127)
pT73-Rab10 MJF-R21 Rabbit Abcam (ab230261)
TSG101 4A10 Mouse Life Technology (MA1-23296)
Alix 3A9 Mouse Ozyme (#2171S)
CD9 EPR2949 Rabbit Abcam (ab92726)
CD63 H-193 Rabbit Santa Cruz (sc-15363)
GAPDH Polyclonal Rabbit Sigma-Aldrich (G9545)

Validation

Antibody validation is provided in several of our figures, including Figure 1, S3, S5, S6, S10, S11, S13, S14, as well as in previous publications such as Davies et al. 2013, PMID 23560750

Animals and other research organisms

Policy information about studies involving animals; ARRIVE guidelines recommended for reporting animal research, and Sex and Gender in Research

Laboratory animals

Sprague Dawley rats (Janvier labs, Le Genest-Saint-Isle, France) were used in the pharmacological treatment experiments. LRRK2 KO rats (HsdSage: LE-Lrrktm1sage)25 and wild type Long Evans hooded rats were also used in experiments to confirm LRRK2 detection in rat uEVs and were ordered from Horizon Discovery (currently, Envigo, Lafayette, CO, USA). Female cynomolgus macaques were also included in the study (Suzhou, PRC)

Wild animals

Not applicable

Reporting on sex

Male rats were used and female cynomolgous macaques were used. As these were initial experiments using treatments that had previously been tested in rats and non human primates but using this time a new sample type (the urinary extracellular vesicles), experimental groups of the same sex were preferred.

Field-collected samples

Not applicable

Ethics oversight

For rats, procedures were approved by the Ethical Committee of the French Ministry of National Education, Higher Education and Research (reference #4271-2015102712365542). For non human primates, research was conducted under an IACUC animal use protocol specific for this study and approved by Suzhou Xishan Zhongke Drug R&D Co., Ltd. (IACUC number, IP171125PD11).

Note that full information on the approval of the study protocol must also be provided in the manuscript.
